# Understanding the initiation, formation, functioning, and performing of networks to change practices – Realist evaluation of a programme to improve newborn care in Kenya

**DOI:** 10.1016/j.ssmhs.2025.100101

**Published:** 2025-12

**Authors:** Katherine Kalaris, Mike English, Geoff Wong

**Affiliations:** a Health Systems Collaborative, Centre for Global Health Research, Nuffield Department of Medicine, University of Oxford, Peter Medawar Building for Pathogen Research, 3 South Parks Road, Oxford OX1 3SY, United Kingdom; b Nuffield Department of Primary Care Health Sciences, University of Oxford, Radcliffe Primary Care Building, 32 Woodstock Road, Oxford OX2 6HT, United Kingdom

**Keywords:** Networks, Health systems, Realist evaluation, Newborn care, Quality care, Relationships

## Abstract

Networks are increasingly employed to tackle health system challenges by either explicitly or implicitly targeting the relational aspects of health systems. We undertook a Realist evaluation to test a programme theory developed during a previous Realist review on network initiation, formation, and functioning. Our aim was to further develop an understanding of the processes involved in the initiation, formation, functioning, performing, and sustaining change and impact of networks that enable changes in practice to improve quality of care and services. We undertook a multiple-methods single case study of the Newborn Essential Solutions and Technologies programme in Kenya to evolve the programme theory. The revised programme theory explains how a network evolves through three phases: Initiation & Formation, Functioning & Performing, and Sustaining Change & Impact through interrelated processes (identify a problem; collective vision; taking action to solve a problem; identity and culture), activities (knowledge and skills dissemination; cross-learning; resourcing; leadership; champions; adaptability), foundations (teamwork; psychological safe space; commitment; engaged, motivated, empowered, and confident network members; purposeful relationships, linkages, and partnerships), and cross-cutting factors (communication; trust; energy, effort, and passion). This network programme theory provides a roadmap for the relational work those employing networks should perform to promote success in changing practices to improve quality of care, service delivery, and health system functioning.

## Highlights

• Networks are increasingly employed to tackle health system challenges.
• Networks explicitly or implicitly target the relational aspects of health systems.
• Network evolution linked by processes, activities, foundations, cross-cutting factors.
• Relationships & relational work in networks are key to success in changing practices.

## 1 Introduction

### 1.1 Networks in health systems

There are an abundance of approaches to tackle the problems of health systems; one such approach with interest and momentum from ministries of health (MoHs) and global and local healthcare stakeholders are networks (Sibbald et al., 2020, Morcol and Wachhaus, 2009, Cunningham et al., 2012). We define networks as: “groups of facilities and/or healthcare affiliated stakeholders linked formally or informally, horizontally or vertically, through programs, interventions, activities, or initiatives” (Kalaris et al., 2023a) with an aim to initiate change to improve service delivery, quality of care, and/or health system functioning. Healthcare affiliated stakeholders include all cadres of healthcare providers, technicians, government officials, professional associations, non-governmental organisations, and donors at all levels of the health system, including the community (Kalaris et al., 2023b).

Networks differ from other approaches because they focus on the relationships between their members and the willingness of their members to participate (Aveling et al., 2012). Many networks form when health system actors come together in a distinct way and work together to improve service delivery, quality of care, or health system functioning. They may form organically in response to a problem or more deliberately through government or external partner initiation (Aveling et al., 2012). Network members, who may come from across levels and sectors of care, health sector entities, and geographies, see value in working with each other to address a mutual problem (Keast et al., 2004, Southon et al., 2005, Brown et al., 2016). These networks are not parallel structures to the health system nor are they geographic or vertical networks of service delivery units underlying the organisational structure of many health systems. Instead, they represent a new, primarily relational, layer within or overlying the existing health system, potentially reinforcing it.

A network approach may provide something unique. Networks may overcome problems caused by fragmented services or organisational barriers (Sheaff and Schofield, 2016) and support sustainability and functioning of network entities (Cunningham et al., 2012). Networks are claimed to be able to achieve goals or changes that may not be feasible by a single organisation or individual, leveraging collective action from network members (Aveling et al., 2012, Sheaff and Schofield, 2016, Provan and Kenis, 2007). They may effectively share knowledge and influence people’s behaviour because they develop norms and values that steer and influence decisions and actions (Aveling et al., 2012). Networks are an approach to tackle complex health system challenges because they work to identify problems and support different network members to collaborate to change practices (Manns and Wasylak, 2019).

Existing research shows clinical outcomes and quality of care can improve in networks (Kamanga et al., 2022, Fasawe et al., 2020, Broughton et al., 2016, Sloan et al., 2018, Ahmed et al., 2019). For example, a network in three states in northern Nigeria with the highest maternal mortality ratios (MMR), reduced the MMR by 37 %, the neonatal mortality rate by 43 %, the stillbirth rate by 15 %, and the perinatal mortality rate by 27 % (Sloan et al., 2018). In a network in Ecuador, the quality of maternal and newborn care improved in facilities in the network compared to comparison sites. This included meeting clinical standards for first antenatal visits (56–90 %) and newborn ambulatory visits (73–88 %) and improved clinical quality for outpatient maternal postpartum visits at health centres (68–97 %) and hospitals (0–81 %) (Broughton et al., 2016). However, there is insufficient understanding of how and why networks form and work. This is important to understand to support future and existing networks, particularly as interest in a network approach grows. The World Health Organization (WHO) recently developed implementation guidance for networks of care for maternal and newborn health (World Health Organization, 2024). In the World Bank’s recent Hospitals in Health Systems report, networking was cited as a strategy to reduce fragmentation and move towards integration to advance quality, equity, and efficiency in health systems (Chopra et al., 2024). The Ghana Health Service has adopted a network approach for primary health care (Agyekum et al., 2023). A better understanding of what to pay attention to in networks can enable a more effective use of both human and financial resources.

#### 1.1.1 Developing an understanding of networks in health systems

This Realist evaluation progressed the understanding of the initiation, formation, functioning, performing, and sustaining change and impact of networks in health systems. It builds on a scoping review on networks in low- and middle-income country (LMIC) health systems that outlined the main components of a network and the uses, purposes, and stakeholders (Kalaris et al., 2023a) and a Realist review that developed a programme theory based on the literature and substantive theory to explain how networks form and function to be able to change practices (Kalaris et al., 2023b). We used the Newborn Essential Solutions and Technologies (NEST360) programme in Kenya as a case study to further evolve our network programme theory. The complexity and urgency of newborn care and the stress that it can cause on systems within and across facilities lends it to be a good candidate for a network and to evolve our network programme theory (Carmone et al., 2020, Kawaza et al., 2020, World Health Organization, 2020).

### 1.2 NEST360

Newborn mortality remains high in many LMICs, including Kenya, in part due to poor quality of care as health facilities lack the technologies, equipment, and trained staff needed to care for small (weighing <2500 g at birth) and sick (having any medical or surgical condition) newborns (NEST360, 2021, World Health Organization, 2020). High-impact low-cost interventions could help avert 71 % of newborn deaths (Nzinga et al., 2019).

NEST360, a multi-country multi-stakeholder programme, aims to implement recommended interventions by providing hospital in-patient newborn units (NBUs) with multifaceted support, including infrastructure improvements, a bundle of medical devices (e.g. CPAP machines, radiant warmers), support for device installation and maintenance, clinical, biomedical, and instructor training, mentoring, and quality improvement (QI) activities. In phase one (2019–2023), NEST360 supported four countries, Kenya, Malawi, Nigeria, and Tanzania, working with governments, bi- and multilateral organisations, global health donors and organisations, civil society, academic institutions, and the private sector.

#### 1.2.1 NEST360 as a network example

We studied NEST360 in Kenya as an example of a network initiated, supported, and financed by external organisations. Our analysis shows how NEST360 functions as a network and why thinking of it in this way may be important for its success. NEST360 formed a network across hospitals and partner organisations in Kenya leveraging opportunities to link clinicians, biomedical engineers (biomeds), county health departments, the national MoH, and stakeholders, which facilitated training, equipment support, and capacity strengthening.

### 1.3 Evaluation objectives

Drawing and building on our prior scoping and Realist reviews we undertook a Realist evaluation of NEST360 taking the perspective that it is a network in a LMIC health system intended to change practice and improve quality of services. Additionally, we assessed whether the programme theory from our Realist review was able to explain why, in what context, and for whom changes happened based on primary data.

The objectives of our Realist evaluation were to:

1. Test (confirm, refute, or refine) the Realist programme theory (developed in our Realist review), using the NEST360 programme in Kenya as an exemplar, to further understanding of the processes involved in the initiation, formation, functioning, performing, and sustaining change and impact of networks that enable changes in practice to improve quality of care and services.
2. Use the refined programme theory to identify transferable lessons and best practices for MoHs, health system managers, clinicians, and health sector partners interested in establishing or scaling up networks, intending to change practices to improve quality of care and services.

### 1.4 Ethical approval

Ethics approval was gained as part of the study Learning to Harness Innovation in Global Health for Quality Care (HIGH-Q) approved by the Oxford Tropical Research Ethics Committee at the University of Oxford (OxTREC Reference 21–26) and the Kenya Medical Research Institute (KEMRI) Scientific and Ethical Review Unit (Protocol No. KEMRI/SERU/CGMR-C/229/4203). Written informed consent was obtained from participants.

## 2 Methods

### 2.1 Rationale

The Realist review developed the initial programme theory for this Realist evaluation on how and why networks are initiated, form, and function to be able to change practices based on the existing literature and ten organisational and sociological theories supporting analytical Context – Mechanism – Outcome configurations (CMOCs) (Kalaris et al., 2023b). A Realist approach was justified as networks are complex intervention strategies because they are composed of many interacting components and actors (individuals and entities) with collective and divergent interests, that operate within complex health systems (Greenhalgh and Papoutsi, 2018, Skivington et al., 2021). The outcomes produced through networks may therefore be dependent on contexts internal to the network or those outside of its control (Skivington et al., 2021). A Realist approach is increasingly employed to make sense of complex social interventions and programmes, and therefore, networks are a suitable candidate for a Realist evaluation.

In the literature we found examples of successful outcomes attributed to networks, however, there is a gap in understanding how and why networks are initiated, form, function, and perform to produce the documented outcomes. Our evaluation aimed to address this gap through the development and refinement of a programme theory.

### 2.2 Evaluation design

Our evaluation focused on the NEST360 programme in Kenya because it was useful to evaluate one country level programme as a specific network in keeping with our initial programme theory (Kalaris et al., 2023b). Any understanding developed could potentially be transferable to other countries with similar contexts and where the same mechanisms may also be in operation (Pawson, 2013).

Our Realist evaluation was a multiple-methods single case study to test (confirm, refute, or refine) the initial programme theory and deepen our understanding of causal processes (Kalaris et al., 2023b, Marchal et al., 2010). We considered the NEST360 programme in Kenya to be the case rather than each hospital being a separate case because we needed to examine it as a whole unit or network. Participant sampling is explained below. The data analysis confirmed, refuted, or refined CMOCs from our Realist review (Kalaris et al., 2023b) and developed new CMOCs where necessary. This evaluation took place from January to December 2023. The evaluation protocol is available in Appendix A.

### 2.3 Programme theory testing

Data collection and analysis centred around the different phases (identify a problem, developing a collective vision, taking action to solve the problem, forming purposeful relationships, linkages, and partnerships, building a network identity and culture, network leadership, and committed, engaged, and motivated network members) of the initial programme theory developed from our Realist review (Kalaris et al., 2023b).

#### 2.3.1 Data collection and sampling

Our evaluation included three methods of data collection: semi-structured Realist interviews, programme document review, and fieldnotes from non-participant observation of meetings, informal interviews, and continuous reflection. Data collection was undertaken in 2023, the fifth year of NEST360 in Kenya.

1) **Semi-structured Realist interviews:** Interview questions were based on the initial programme theory to understand how, where, when, and why the network works or does not work and drew on experiences from study participants who could provide information on programme processes and outcomes (Manzano, 2016, Pawson and Tilley, 1997). Fieldnotes and post-interview reflections were used to identify areas that seemed most relevant to explore in later interviews. The interview guide is in Appendix B.
  Prior to starting the interviews, we held informal introductory conversations with members of the NEST360 Kenya team; this helped purposively sample and select interview participants. Participants included NEST360 researchers and implementers based internationally and in Kenya, NEST360 partners, and MoH representatives. Four hospitals were selected based on the following criteria: part of NEST360 and HIGH-Q, geographic mix, mix of well-poor performing, and likely to have relevant information. At each hospital, participants were purposively sampled and included biomeds, hospital administrators, and NBU clinical and nursing staff. We conducted additional interviews with participants from three other hospitals in the NEST360 network during the Kenya Paediatric Association (KPA) annual conference.
  The number of participants was based on the concept of Information Power, which argues that the larger the information power in a sample, the fewer study participants needed in the sample and visa-versa (Malterud et al., 2016). We found this approach to sample size appropriate for our evaluation as the case focused on a specific network and all participants needed to have specific knowledge of and experiences with the network. A total of 33 formal interviews were completed.
2) **Document review:** We identified programme documents and reports from NEST360 globally and specific to Kenya, including annual NEST360 programme reports, quarterly NEST360 newsletters, and NEST360 blog posts, which provided background information on the NEST360 programme, particularly to provide an understanding of how the programme intended to work and its achievements. The list of analysed documents is in Appendix C.
3) **Fieldnotes from meetings, informal interviews, and reflections:** At the beginning of data collection, we held three informal interviews which helped to orient the formal interviews. We informally observed two NEST360 meetings, one online and one in-person. The selection of meetings was based on their relevance to the programme theory. This source of data provided insights into interactions and communication between the different network members and how they work together. Fieldwork reflections and discussions amongst the authors helped to understand how the data was potentially evolving the programme theory.

#### 2.3.2 Data analysis

Interview transcripts, programme documents, and fieldnotes were imported into NVivo QRS International for qualitative coding. Transcripts were reviewed again for familiarisation and to understand emerging patterns in the data. The data analysis process was iterative. Transcripts, programme documents, and fieldnotes were reviewed and analysed in waves, starting with data that were most likely to hold the most relevant information. Data were coded deductively with codes developed from the initial programme theory and inductively with new codes that emerged from the data relevant to our research questions. Already coded transcripts and documents were rereviewed when new codes emerged.

Coded data were reviewed and grouped with existing parts of the programme theory and CMOCs. We then considered if the data confirmed, refuted, or refined each existing CMOC. To do this, we identified parts of the data that were relevant to and helped to assess the CMOC.

The data elucidated parts of the programme theory that were not found in the literature. Relevant data that were not yet represented in the programme theory were grouped in new sections or in new sub-sections of existing parts of the programme theory to elaborate new aspects. To develop new CMOCs, the same analytic processes were followed as outlined in our Realist review (Kalaris et al., 2023b). CMOCs were iteratively revised, consolidated, and repositioned to where they made most sense in the programme theory and with the supporting data.

#### 2.3.3 Research team and positionality

This research was undertaken by KK as part of her doctoral work supervised by GW (realist methodologist) and ME (health systems expert with 25 + years’ experience working in Kenya) under a large research project, HIGH-Q, led by a consortium from the KEMRI Wellcome Trust Research Programme (KWTRP), the Kenya Paediatric Research Consortium, and the University of Oxford. The HIGH-Q project looked at the impact of workforce interventions in a set of NBUs supported by NEST360 programme.

The research team has previous work on networks; prior to and during her doctoral work, KK worked on networks of care and ME established the Clinical Information Network in Kenya. This prior experience has likely influenced our positionality towards the topic as a worthwhile approach to improve health systems functioning, quality of care, and outcomes. The interviews and analysis were led by KK, who undertook this Realist work in Kenya as an American woman and doctoral student with 10 years’ experience in global health programmes. Interview questions were posed at a level of specificity that was open enough to be able to elicit interviewee reflections, perspectives, and experiences to the greatest degree, but also specific enough to keep the interview focused on NEST360. Data collection was supported by colleagues at the KWTRP. KK kept a fieldnote journal to reflect on the interviews and document immediate perspectives and discussed regularly with GW and ME throughout the process. The interviews and the analysis were undertaken in a robust and transparent way to manage the limitations of doing Realist work as an outsider.

### 2.4 Theory consolidation

As the programme theory was iteratively refined with CMOCs revised or developed, substantive theories from our Realist review were reconsidered for applicability. We searched for and investigated additional theories to clarify the underlying mechanisms acting within the programme theory and to provide analogy, especially for new parts of the programme theory. Appendix D provides an overview of the substantiative theories used to support the refinement of the programme theory. A stakeholder group was convened to present and obtain external input on the revised programme theory to support sense-making. The stakeholders felt that the revised programme theory resonated with their knowledge of and experiences with different networks. They provided input on the place of trust in the programme theory, network sustainability, psychological safety, resourcing across network levels, and how to define the success of networks.

## 3 Results

### 3.1 Study participants

Our study included 33 individuals from NEST360 Global (4), NEST360 Kenya (6), the MoH (2), hospitals (18), and partners (3). At the four hospitals we visited, we interviewed five people at one hospital, four at the second, and three at the remaining two hospitals. The individuals interviewed at KPA (3) each came from different hospitals.

### 3.2 Refined network programme theory

**Fig. 1:**
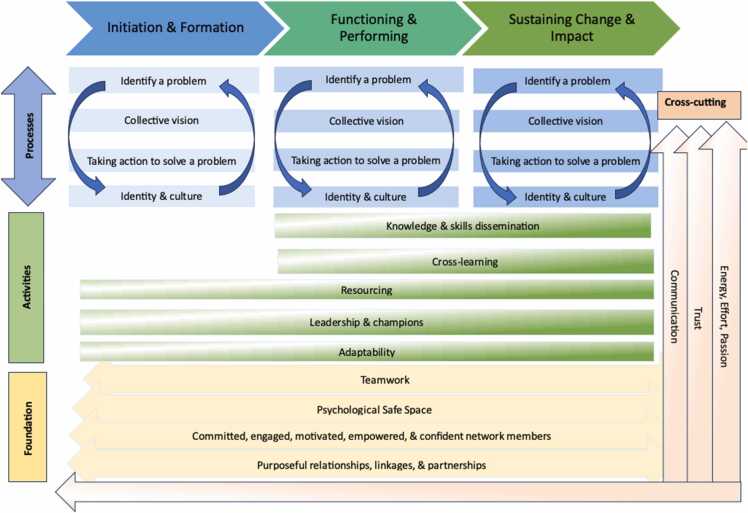
Refined network programme theory.

**Fig. 2:**
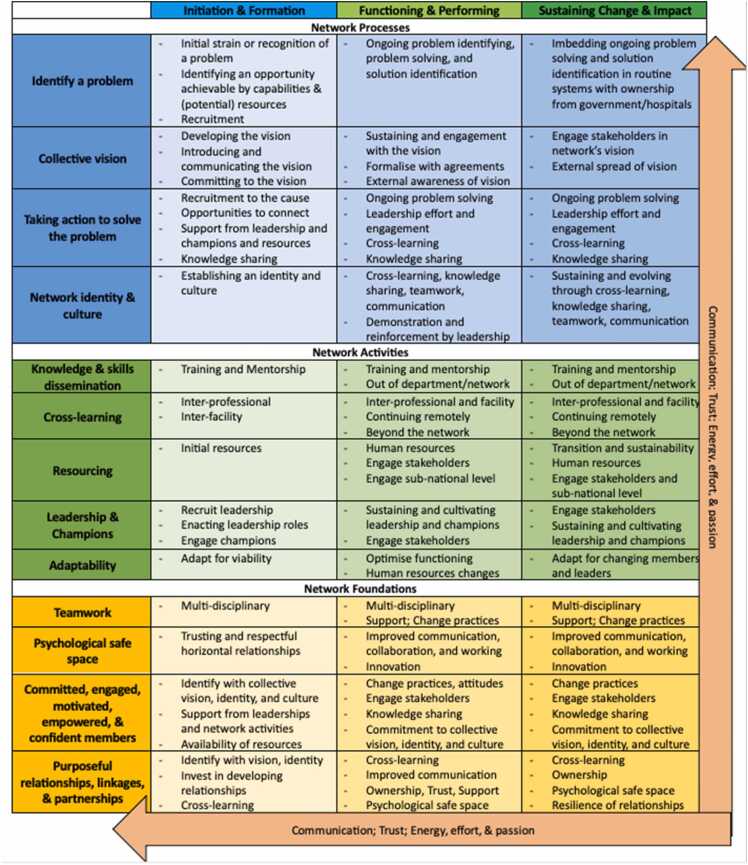
Network programme theory framework.

A network evolves through three phases: Initiation & Formation, Functioning & Performing, and Sustaining Change & Impact through a set of interdependent processes, activities, foundations, and cross-cutting factors. The network processes—identify a problem, collective vision, taking action to solve a problem, and identity and culture—are dynamic and active processes that continue throughout network evolution but manifest in different ways across the phases. Network activities, such as knowledge and skills dissemination, cross-learning, resourcing, leadership and champions, and adaptability, are undertaken throughout network evolution and may increase in intensity as the network begins to function and perform. At the foundation of networks are teamwork; a psychological safe space; committed, engaged, motivated, empowered, and confident network members; and purposeful relationships, linkages, and partnerships. Present throughout the programme theory are communication, trust, and energy, effort, and passion. Fig. 1 illustrates the revised programme theory and sets out the interrelations between the concepts and processes. Fig. 2 describes each of these processes, activities, foundations, and cross-cutting factors. Appendix E links the different parts of the programme theory to the CMOCs.

The programme theory was refined based on 143 CMOCs. Based on the primary data, 18 CMOCs were confirmed, 30 CMOCs were refined, and 95 new CMOCs (55 in existing and 40 in new parts of the programme theory) were developed based on the evolved understanding that the primary data brought to the programme theory. Data collection was undertaken while the network was operating and this helped to expand understanding of network functioning and performing and, after over 4 years of NEST360 activity, to develop an understanding of sustaining change and impact. Appendix F shows how the CMOCs from our Realist review were refined with the primary data. The following sections summarise the programme theory, the full set of refined CMOCs, which underpin the programme theory, with examples of data is in Appendix G. Bolded text in each section below highlights the key points of each section of the programme theory. One example CMOC and an illustrative piece of data are included for each section of the programme theory. Extended narrative results, which include application of the substantive theories, are in Appendix H.

#### 3.2.1 Network processes

This first section of the programme theory explains the processes that networks undertake to initiate, form, function, perform, and sustain change and impact (See Processes in Fig. 1 & 2).

**Identify a problem**: **The initiation of a network starts with** ***identifying a problem*** **coupled with network initiators seeing an opportunity and believing that they have (or can generate) the capabilities and resources to do something about the identified problem. Network initiators will then recruit other people, particularly potential leaders or champions, into the cause and collectively work to understand what they need to do to solve the** ***identified problem*****. Depending on the network, this may be people who directly feel the strain or who see the magnitude of the problem. Network initiators engage with potential network members to support forming the network and start to generate their commitment to solving the problem through the network.**

NEST360 aims to reduce high preventable newborn morbidity and mortality. The network initiators understood and saw the magnitude of the problem, but they also knew they had the capabilities and potential access to resources to do something about it. Healthcare workers, biomeds, and hospital administrators that feel the strain and frustration of the problem, lacked access to the medical devices and training to substantially act on the problem. Once in existence, well-functioning networks can be employed to identify additional problems. The NEST360 network puts in effort to continually *identify problems* linked to improving care for small and sick newborn and takes action to improve them, including QI dashboards and visits, audits, and mentorship visits; see CMOC 1 H below as an example.

CMOC 1 H:

**Table:** 

When members in an established network feel it is safe to critically examine and reflect on existing practices together (context), they are able to identify new problems and potential solutions (outcome) because they feel enabled to challenge the status quo (mechanism) and/or feel in a psychological safe space to be able to challenge the status quo as a team (mechanism)
*“And I think also the the growing recognition and appreciation of some of the problems that still continue to contribute to neonatal mortality, for instance, hypothermia management, you know, that's something that every meeting, every place we went, people recognise that hypothermia, both in inborns* *and outborns, is still a major, major challenge. And through those meetings, some of them put corrective measures. So, for instance, some hospitals started using plastic wraps that they were not using before but after, you know, discussions with our teams, they took it back but taken it as an intervention.” (FI 18)*

**Collective vision: Emerging from the identified problem, a** ***collective vision*** **is formed and introduced to potential network members.**

In the NEST360 network, the *collective vision,* was developed by external network initiators but it resonated with potential network members as newborn mortality and morbidity, a global problem, is a policy priority in Kenya. Different approaches facilitated potential NEST360 network members to commit to the *collective vision*, including, a shared understanding of challenges in small and sick newborn care, pre-existing relationships, resourcing network NBUs early on, and communicating the *collective vision* in a way that appeals to professional ideals and values of network members (see CMOC 2 I).

CMOC 2 I:

**Table:** 

If the collective vision of a network is communicated and explained by network leadership in a way that appeals to professional ideals and values shared by potential network members (context), then this will lead to commitment to the collective vision (outcome) because potential network members feel engaged (mechanism)
*“I think there was, you know, the quantification of the problem, the magnitude of the problem, for people to really understand what they're dealing with. And, you know, visualizing that, you know, just how many babies are dying and promising with a promise that, you know, at a very, you know, with a* *very low cost, cost-effective solution, you can reverse that was. Those that conversation helped a lot, because we all working together towards a common problem where we think we have an opportunity to do something.” (FI 31)*

**Taking action to solve the problem: Network members first** ***take action*** **by recruiting others to join the collective vision. In an externally initiated network, the energy and effort put in to recruit others ensures a diverse mix of skills and competencies. Network members will also seek out those with common experiences or perspectives. They then** ***take action*** **to work towards solving the identified problem supported by the existence of resources, opportunities to coordinate efforts and work within and across cadres and organisations, supportive leadership, and platforms for communication.**

In the NEST360 network, NEST360 Global recruited a diverse mix of partners with complementary skill sets

and expertise to organise the network in Kenya. Within network hospitals, NBUs recruited other hospital. staff to support their unit in working towards the collective vision. Within network hospitals, the resources (equipment, parts, and training) NEST360 provides encouraged network hospitals to *take action* because with the necessary resources, they had more capacity to improve care. The feeling of working as a team within and across hospitals, NEST360 network meetings, support from NEST360 Kenya for mentoring, QI, training, and network WhatsApp groups (see CMOC 3 I) all supported the hospitals in *taking action* towards changing their newborn care practices.

CMOC 3 I:

**Table:** 

When network members are provided with a platform for them to easily communicate (e.g. WhatsApp groups) (context), they are more able to take practical and concrete action to solve problems (outcome) because they can access and gain the knowledge and reassurance they need (mechanism)
*“There were they were a couple of WhatsApp groups that were created that were specifically for NEST that were very instrumental at that early age for people to bond to chat to say what they’re seeing, to actually say what devices are working what challenges are there. Was the same platform that was used for dissemination of doing information sharing materials, webinars, and getting people to invite people to webinars. So, I think there was a lot of that informal network, so to speak, in terms of in terms of how people interacted at that early stage.” (FI 32)*

**Identity and culture: A network’s identity and culture come from a feeling of collective identity among network members that is linked with identifying with the network’s collective vision.**

NEST360 created a *network identity and culture* that stems from network members’ passion for newborn care. Network hospitals and members feel part of a *culture* to improve newborn care and when they identify with other network members and the network’s vision, this can support the development of a network identity (see CMCO 6 C). The provision of resources, for example training, equipment, and mentoring activities, helped to create a separate *network identity,* but this may make the *identity and culture* less sustainable when these resources are no longer provided to the network hospitals. However, NEST360 creates opportunities across network hospitals for members to connect, which reinforces a sense of belonging to the network and spreads its *culture*. Findings also show that for some network hospitals, a positive pre-existing *culture* that aligns with the network’s vision can positively support the development of a *network identity and culture*.

CMOC 6 C:

**Table:** 

When network members identify with other network members and the network’s vision (context), they develop a network identity (outcome) because it gives them a sense of purpose (mechanism)
*“Let's say we feel proud to be part of NEST, we feel we refer ourselves as the NEST hospitals. And because it came and changed how we do things. It's changed how we you know, we perceive things, it's changed our outcomes. So, we are very proud to be part of it. I must say, it's like, yeah, it's like a movement sort of. So, first of all, you are always motivated to do the best you can, you're motivated to make a difference for that child. And, you know, you stop doing things just the way you were doing them before, because now you know, better. So, I think it's more than just adding the group of people but from within, they found it changed in you. And then when you come together as a group, I think it's more of a movement. We want to make a difference for the children. So, I hope that can continue.” (FI 23)*

#### 3.2.2 Network activities

Networks undertake different activities for Initiation & Forming, Functioning & Performing, and Sustaining Change & Impact (See Activities in Fig. 1 & 2). There may be other activities that networks undertake but based on the primary data and our preceding scoping and Realist reviews, (Kalaris et al., 2023a, Kalaris et al., 2023b) the ones described below are likely the most important.

**Knowledge and skills dissemination: When a network** ***disseminates knowledge and skills*** **by creating training, mentorship, and learning opportunities, this can change and improve practices related to the identified problem and improve network relationships.**

Knowledge and skills dissemination is an important aspect of the NEST360 network. Clinical pre-service and in-service training materials were updated and training materials on NEST360 supported devices were developed for the biomeds. A course to train trainers in teaching skills was created. NBUs integrated maternity and postnatal wards and referring facilities into facility trainings and continuing medical education (CME) which has improved their relationships with other departments and facilities (see CMCO 14 C).

CMOC 14 C:

**Table:** 

When network members include providers in non-network facilities in knowledge sharing opportunities (context), this supports their ability to improve practices (outcome) because they develop essential skills and knowledge (mechanism)
*“We have linkages with the lower-level facilities. We know those facilities that refer to us a lot. Even when we were doing the training, the newborn ETAT training, we incorporated them into the training. Yes, because we found that they were referring and there could be a knowledge gap. So, we actually took two from the referring facilities and trained. And whenever we get opportunities for training because I have a book where I record all the referrals, referrals, so I see the facilities that refer so much. And whenever I get a training opportunity, I usually call them yeah for training. So, we have those linkages.” (FI 22)*

**Cross-learning:** ***Cross-learning*** **within and across network hospitals helps change practices in networks and facilitates the development of purposeful relationships.**

*Cross-learning* occurs in the NEST360 network through inter-professional trainings, hospital trainings and CMEs, network review meetings, supportive supervision, mentoring, QI activities, and network webinars. Network meetings provide nurses, clinicians, and hospital administrators opportunities to share experiences, learnings, and challenges with each other (see CMOC 13 C). Cross-learning opportunities have led to better working relationships between clinical and biomedical network members, better linkages between the NBU and labour and postnatal wards, and cultivated relationships across the network. Network members use WhatsApp groups to continue learning from each other in-between more formal learning opportunities.

CMOC 13 C:

**Table:** 

When the network brings together network members through meetings (context), this helps them to better solve problems (outcome) because they get to learn from each other (mechanism)
*“One of the challenges we have had is the issue of we have not been doing very well with the CPAP uptake despite the fact that we got the machines. So, one of the meetings or in some of the meetings, we've tried to compare with other facilities, which are doing better. And so, we learned that some* *facilities were doing what they really invested in CPAP champions. So, these are people who are the ones who are running that idea. And because what we noticed is that after the initial training, over time, the confidence levels went down. But when we got now those sharings and the issue of champions,* *you know, we've tried to get one champion here. And we have seen that that has helped us quite quite a lot.” (FI 25)*

**Resourcing: Networks require tangible** ***resources*****, such as financial resources, human resources, infrastructure, medical devices, and pharmaceutical products, as well as intangible ones, such as time of network members, throughout their evolution.**

The NEST360 network has provided significant financial *resources*, infrastructure upgrades, equipment, parts, consumables, and capacity building activities (e.g. training, mentoring, QI) to hospitals. As NEST360 begins to transition direct *resource* support from existing network hospitals, there may be challenges for the health system to match the level of *resource* input provided by NEST360. Network hospitals are understaffed and so existing staff may have challenges in sustaining the changes in practice and network activities. The NEST360 Kenya team are engaging with stakeholders and county governments to help sustain network activities and the network’s reach (see CMOC 16 E).

CMOC 16 E:

**Table:** 

When the network makes efforts to engage local/sub-national government in the network and the collective vision (context), members can use their skills and resources to support network activities and sustainability (outcome) because they appreciate the value of the network (mechanism)
*“I think maybe, importantly, maybe looking, in retrospect, one of the things we realised is that we didn't have enough government involvement. And I think this has actually contributed it's one of the challenges in implementation. And I think one of the challenges we have as a programme is because of the devolution aspect as well, because when NEST came in, well the national government was quite aware, but now this sub-national level, in most places was not aware, because MoUs were actually done with hospitals themselves rather than the county government. So I think that is one thing we are working to reverse and yeah …because now, initially, it was working very well. But then you see, as time goes, even leadership in hospital keeps changing. So, when you have a new team, a new leader, see a new nurse manager, a new, and so you go there as NEST, ‘I'm not aware’ or ‘What is that?’ Or sometimes they tell you, ‘Oh, is the county aware that you're here? Don't you think you need to inform the county and that allow you to come into the hospital.’ You know, so I think one of the things, I think where we missed a mark was getting the sub-national level government that we work in getting involved. But other than that, I think we are working well, everybody's happy here.” (FI 14)*

**Leadership: Network leadership enacts various leadership capabilities to support network evolution, including, communicating and bringing people into the collection vision, providing support and feedback to network members, championing practices, and creating a supporting network environment. Leaderships needs to be identified or grown and cultivated; changes in leadership can be a challenge for a network.**

There are different layers of *leadership* in NEST360: NEST360 Global, NEST360 Kenya, and *leaders* at the hospitals (medical superintendents) and within the NBU (nurse-in-charge, paediatricians). Each of these *leaders* enact different *leadership* capabilities in different parts of the network. For example, consistently and regularly supporting and providing resources to network members, which helps network members act to achieve the network’s collective vision (see CMOC 5 I). One participant saw *leadership* as an important success factor: *“leadership to me was one one big unaccounted for factor in the success of this programme” (FI 31)*. This statement links to one of our overall findings– that there are many other factors that need to occur or be in place (that make up the network) for the central activities that NEST360 has put in place to work. Among the NEST360 Kenya *leadership*, there were several changes in partner organisations. This made implementation of network activities challenging, delayed, and frustrating for the team providing support to the hospitals, who remained mostly the same.

CMOC 5 I:

**Table:** 

When network leadership consistently and regularly supports and provides resources to network members (context), this helps network members act to achieve the network’s collective vision (outcome) because the network members feel empowered (mechanism)
*“NEST is one of the organisations that did not just apply and run away, they have actually stuck with the with the facilities like throughout the life of NEST, as long as NEST is still existing. Then I think it is a culture that was cultivated in at the onset of NEST because most of the organisations, they start a project and they run away. Even when they are still existing, probably they would have supplied the equipment and run away. Now they take care of everything. But here we are, we are stuck. And the facility biomeds are very comfortable. You should do a bit of the questions for the areas where NEST is existing, they will actually tell you, there is a very big change when it comes. There is a big change, there is a big difference between other organisations.” (FI 17)*

**Champions: Network** ***champions*** **have the skills, passion, and energy to take action and support and motivate other network members. Their passion aligns with the network’s collective vision and they put extra energy into making things happen.**

In the early stages of the network, NEST360 *network champions* were passionate, respected people with a good understanding of the context of the network hospitals, and engaged well with the hospitals. They were key in forming the initial network relationships (see CMOC 11 A). As the network began to function, additional *champions* emerged as network trainers and mentors. Some *champions* brought with them relationships that are beneficial to network functioning, for example, from the MoH. Over time, some *champions* have transitioned out and left a void. One network hospital felt a reduction in the energy and enthusiasm around network activities following a *champion’s* departure.

CMOC 11 A:

**Table:** 

When a network provides a platform for members who are passionate and believe in the collective vision (context), they are willing to be champions (outcome) because doing so enables them to initiate change (mechanism)
*“networks and and teams really depend on champions. And the success of a lot of this implementation work and and how thing take up and taken up and successful in some of the environments is who the champions are on the ground. I think part of the success for NEST to some degree is because of the champions that NEST had when it started, there were passionate people, there were people who were respected had a good knowledge of the terrain, engaged very well with people at the hospital level. And that played a very huge role in creating this collegial partnership and network of people who were willing to work together. And I think that's something maybe is as can't be quantified or can’t be it's not tangible, per se, but it's it's a software component that we might overlook that sort of played quite a role because of the champions behind CIN the champions so to come in NEST initially and created all these other and mentored other people to actually be champions and advocates for NEST.” (FI 32)*

**Adaptability: Networks exist in complex open systems and will need to adapt to form and undertake activities to work towards achieving their collective vision and sustain the change and impact they achieve.**

The NEST360 network has had to *adapt* throughout its evolution. During the network’s formation, the network had to significantly *adapt* its plans and activities because of COVID-19 related restrictions. NEST360 modified in-person trainings for clinicians and nurses on newborn care and devices to a hybrid format with three-days of virtual teaching followed by two-days of in-person practical training. Training for biomeds was conducted virtually and followed-up with mentorship during device installation and continued remote support. Meetings were moved online and tools such as Zoom and WhatsApp were leveraged to facilitate virtual trainings, meetings, and communication.

The network has had to continually *adapt* to changes among trained staff on the NBU and biomeds. In response, remaining NBU and biomedical staff and mentors train the replacement staff on-the-job to avoid loss of skills needed to work towards the collective vision (see CMCO 15 C). The NEST360 network in Kenya is organised by a consortium of organisations and over the evolution of the network, there have been organisations who have left and been replaced by other. The network has had to *adapt* to different and changing processes to execute network activities, which have been challenging to the organisation staff, who have largely remained the same, and required them to *adapt* to changing operational and financial processes to support network hospitals and activities.

CMOC: 15 C

**Table:** 

When skilled and capable network members leave network facilities (e.g. move to non-network facilities) (context), network members work to restore those same competencies within the network (outcome) because they recognise the value and importance of those skills in the network (mechanism)
*“What we try to do and what to try to hope that happens in the field, should there be such a case that some mentorship needs to be done, we hope that amongst the team in the field that already attended the skills training, at least there should be one or two that are still there, because, again, within our system, their county health system, rotations and transfers are always there. So, you'll find that in most cases, and currently, we will face to be the one that ideally all NEST, all five NEST trained biomeds, all got transferred. So, a new team was brought all of them. And the new team came in and then we were like, ‘Hey, okay, so we are starting from square one.’ So, we normally hope that amongst those that we will be transferred, at least one or two should be left behind. So that this one or two, I am able to organise for some sort of tele-mentorship that you could either do on the phone or in the during our webinars. It makes it easier for them because already they have a clue of what we'll be talking about they'd already attended the physical training the onsite training.” (FI 15)*

#### 3.2.3 Network foundations

Network processes and activities are underpinned by teamwork; a psychological safe space; committed, engaged, motivated, empowered, and confident network members; and purposeful relationships, linkages, and partnerships (See Foundations in Fig. 1 & 2).

**Teamwork: Networks can facilitate improving teamwork among network members and across network hospitals through multidisciplinary team meetings, trainings, inter- and intra-facility QI, and mentoring, which can lead to better support and increased initiative to provide care and change practices.**

The NEST360 network established and strengthened *teamwork* within and across network hospitals, among the network organisers, and between network hospitals and the network organisers through multidisciplinary meetings that included clinicians, nurses, biomeds, and hospital administrators, trainings across the network and within hospitals, and intra-and inter facility QI and mentoring activities. *Teamwork* was built and strengthened between clinical network members and the biomeds and their relationships and ability to work together is one of the noteworthy changes in practice in the network (see CMOC 12 B).

CMOC 12 B:

**Table:** 

When the training provided to network members is multidisciplinary (context), this enables interprofessional teamwork among network members (outcome) because they understand each other’s roles and capabilities (mechanism)
*“I wouldn’t call it a change, let me call it a transformation because it’s a very big change about the the nurses and the biomeds and the clinicians working together for a patient. That’s when you have maybe if they want to fix a patient, they are not very sure of the settings or how we are being, told they include you there. We want to fix this patient. We want this is not coming on the way, we want assistance, that big, big collaboration. And that one, maybe on the department we are seeing of it. But in NBU it’s a big, big transformation there. Yes. Yes. And I think maybe if the Minister or in other partners, maybe they upgrade that cooperation, it works very well, even assisting with the patient. And then machine usage. You minimize those minor breakdowns. Yes. When the nurses and the clinician are that confidence about the machine.” (FI 24)*

**Psychological safe space: Networks create and promote a psychological safe space when network members form trusting and respectful relationships, which can support network members to learn, improve, seek feedback, openly raise concerns and problems, encourage innovation, and facilitate communication and collaboration.**

In the NEST360 network, inter- and intra-facility network meetings helped to create teamwork. This has enabled network members to share openly and freely experiences and challenges and identify ways to change practices. The network has created an environment where network members do not feel a sense of fault finding but of improvement (see CMOC 9 D). Network hospitals can be innovative and share what they have learned with other hospitals in the network. Communication has also improved between network hospitals and the facilities that refer to them and between the MoH and network hospitals.

CMOC 9 D:

**Table:** 

When a network is a psychological safe space for network members (context), it enables members to openly raise concerns or problems (outcome) because they know they will be supported and there will not be negative repercussions (mechanism)
*“In the beginning, you can you can tell it's the they might not be but we really try to moderate the tone of the meeting. So that it's not fault-finding, about blaming, we just want to understand the process they went through. And we've seen, like some cases, we thought were cut and dried that this maybe it wasn't so easy. And they you realise that the staff might be trained and experienced. But what's available in terms of equipment and monitoring for them to use is…by inviting like, there's a case where we realised we didn't have the answers, even after talking to them because the person who came was an experienced midwife. And we felt if we had post-mortems, for example, for some of these babies, if it's acceptable to the family, which is a different discussion, we might have learnt a bit more about why they presented the way they did. So, we try to make the tone of the meeting one where we are meeting as colleagues to discuss our our the things that we are grappling with and what we also try to inverse it. So, when they tell us their challenges, we tell them our challenges, too. We say like see, we have issues with documentation, we also did not do this. And we asked for their feedback. Like how were you handled when you came? Did you feel victimised? So, that we can go back to the team and tell them to be careful how we handled them. Because we're one team and we need to make people comfortable talking, voicing their concerns.” (FI 03)*

**Commitment*****:*** **Commitment to a network is generated when network members identify with the network’s collective vision, identity, and culture, when their professional identity/calling aligns with the vision, when they have support from leadership or stakeholders, when the network enables members to achieve professional norms, and when network members feel emotional benefits or a sense of purpose from the network. Networks with committed members are more likely to act on the identified problem and collective vision and take action to disseminate knowledge to other members and those external to the network.**

NEST360 has worked to cultivate *commitment* from the NBUs, hospital leadership, the MoH, and county health departments. Network members feel proud and positive about being part of the NEST360 network. The support provided by the NEST360 Kenya team in terms of training, mentorship, and ad hoc support to the network members helps them feel a sense of belonging (see CMOC 7 E). With the *commitment* that network members feel towards the network, they are motivated to act on the collective vision.

CMOC 7 E:

**Table:** 

When a network member gets ‘emotional’ benefits (positive feelings) or feel a sense of purpose from being part of the network (context), they are likely to be highly committed (outcome) because it is fulfilling for them (mechanism)
*“So, I'm really happy for this programme, it has made us to be up to the task of just not doing things, you are doing things knowing that at the end of this month, I'm being monitored, what have I done right? What have I done wrong? You see and it was good to have… know when you go to meetings and you see that glucose monitoring [name of facility] you at number 10 here, but [name of facility] is number one. You want to see I'm going to [name of facility] that the nurse there but that you're doing right so that I can reach you on. NEST has opened up our minds as a programme.” (FI 11)*

**Engaged and motivated network members: Network members can become engaged and motivated when network leadership provides opportunities for network members to be supported, recognised, and learn, personal identity aligns with network identity and culture, and network change practices align with professional values.**

NEST360 has engaged and motivated network members through the types of people they have recruited, many of whom have a professional calling towards newborn care that has led them to be passionate network members. NEST360 has brought significant resources and opportunities to be supported, recognised, and learn (see CMOC 8 A) to the hospitals in the network and so there are likely some network members who are engaged and motivated because of this.

CMOC 8 A:

**Table:** 

When network leadership provides opportunities for network members to be supported, recognised, and learn through training, equipment provision and support, and facility renovations (context), this creates engaged and motivated network members (outcome) because they derive direct benefits and feel like they belong to the network (mechanism)
*“But with NEST it's narrowed down to the people that are hands on, for our case, the devices, the people that are really with the devices on a day-to-day basis. And with that, we feel we are part of the programme because you're able to contact a technical person in the programme that is readily available to assist in terms of selling as well as in terms of assisting, even troubleshooting in terms of diagnosis, in terms of repairing, and even at some point they can even be able to come and physically support. Then in terms of training again, the kinds of training that NEST has been offering is quite different from so many trainings that have been able to attend because the kind of training that NEST is offering it's not just a training to understand the equipment but they're gauging ‘Do you have the necessary skills to handle this piece of device?’ I speak that because I'm also a trainer with them. So, I'm also a trainer with them. And this was really assisted us to feel to be part of NEST. And also, we're able to give those monthly reports to them. So, every time we share the reports, they're able to gauge the weakest points we are having. And from there they are now to support.” (FI 08)*

**Empowered and confident network members: As network members become committed to and engaged with the network, their confidence in their skills may improve and they may feel more empowered to enact these skills and their role within and beyond the network. A network empowers and increases confidence of network members by selecting them to be network trainers and mentors or providing them support to help improve and expand their skill set leading them to apply their skills beyond the network, independently take action to address problems, and change attitudes towards providing care.**

The NEST360 network created *confident and empowered network members* through its relationship building and network activities, such as training and mentoring (see CMOC 10 B). The biomeds were able to take the skills they learned as part of the NEST360 network to other departments within the hospital. NEST360 helped the nurses in the NBU feel *confident and empowered* in their skills and roles, so that they were able to address problems and provide care without waiting for their more senior colleagues.

CMOC 10 B:

**Table:** 

When network members receive support (e.g. training, mentorship) to help them improve and expand their skill set (context), they become more confident in their previously learned and newly acquired skills (outcome) because they develop the capacity to perform them (mechanism)
*“Also, the use of CPAP, most of this stuff, when we started the programme, most of the clinical staff have been lacking the confidence let me say that to use CPAP. Because to them, it it has been like a complex device to use given the mortality around the use of device, of course, and it sort of brought* *about some negative attitude around using it. So, with time with the trainings with the mentorship, this builds so much on their capacity and their skills and and boosted their confidence. And speaking of hospital like Kiambu they've been doing well when it comes to use of CPAP and and then in one or two other facilities.” (FI 20)*

**Purposeful relationships, linkages, and partnerships: Relationships are an essential part of a network and the intention and strength of these relationships set them apart from traditional health system connections. Relationships can form when there is belief in the network’s collective vision; network members are open to and able to invest time in developing relationships through the network; there are strong pre-existing** ***relationships*** **between network members; there is a shared professional identity; and there is a common baseline understanding between network members and what is expected of them. Networks create opportunities for building relationships by providing resources that supports the network members ways of working and when network leadership creates a psychological safe space that enables cross-learning between network members. With established relationships, communication is improved between network members; ownership of the network may be strengthened; understanding and trust between network members is built; members are better able to provide support to work towards the identified problem and create a psychological safe space; and attitudes towards other professions in the network may change.**

The NEST360 network had the benefit of pre-existing relationships from the Clinical Information Network (NEST360 is a subset of hospitals from this network) (English et al., 2017). However, despite these pre-existing *relationships* the NEST360 Kenya team and network members still needed to put in effort to strengthen these existing *relationships* and build new ones. When there were changes within people or network organisations, this undermined network functioning because new *relationships* needed to be formed. The NEST360 network created different opportunities to develop and strengthen *relationships*, including cross-professional trainings with clinicians, nurses, and biomeds and trainings across levels of facilities. Opportunities for cross-learning at inter-hospital network meetings helped to form *relationships* amongst hospital administrators, nurses, and paediatricians at different hospitals in the network. *Relationships* in the NEST360 network have improved communication between NBU nurses and biomeds and network hospitals (see CMOC 4 J), generated a feeling of ownership of the network activities on the part of the hospitals, enabled support in changing and improving clinical and biomedical practices, and created a feeling of psychological safety within and among the NBUs in the network. While trust emerged in the *relationships* between the network organisers (NEST360 Kenya) and network hospitals, changes to the organisations that make up the NEST360 Kenya team weakened this trust.

CMOC 4 J:

**Table:** 

When network members have established purposeful relationships within and across network facilities (context), this helps to improve communication between network members (outcome) because they are familiar with each other (mechanism)
*“It is very important, I will say, prior to NEST, [name of county] County and [name of county] County are just about 50 kilometres away. But trust me, I had never even visited that newborn unit. I didn't even know whatever was happening there. Currently, it's very easy, you know, you when you have a case, you can call your colleague say, we have this business or that, have you seen this before? How are you handling this scenario? How are you handling this, this, and that? So, it's very it's very good. It's and I'm even called by the nursing team, nurses from other hospitals, you know, they'll be like, ‘Oh, Doc, we have this scenario. We've done A-B-C-D and probably, we're not getting anywhere, what would be the best thing to do?’ When it comes to consumables the various things, you know, in the lab and all that I'm able to ask my colleagues ‘Anyway, do you have this in excess? Could you give me this for sometime?,’ you know, and all that.” (FI 04)*

#### 3.2.4 Network cross-cutting strategies

The network cross-cutting factors, *communication, trust,* and *energy, effort, and passion*, (see Cross-cutting arrows in Fig. 1 & 2) are not supported by separate sections of CMOCs and data but they appear across the network processes, activities, and foundations. *Communication* is required for network initiation and can improve among network members as the network evolves. *Trust* emerges from the processes, activities, and foundations but is pre-existing to a degree for network initiation to occur. The formation of purposeful relationships, linkages, and partnerships can help to build *trust* within the network. *Trust* is important in the formation and functioning of a network, (Kalaris et al., 2023a) however it did not emerge strongly. *Energy, effort, and passion* are needed from a dedicated group of network members for the network to form and function, but as the network evolves, this becomes more distributed across the network.

## 4 Discussion

### 4.1 Summary of findings & comparison with existing literature

The primary data refined and progressed the programme theory on network initiation, formation, and functioning developed from our Realist review. This refined theory explains the key processes, activities, foundations, and cross-cutting factors necessary for network Initiation & Formation, Functioning & Performing, and Sustaining Change & Impact achieved by the network (Fig. 1 & 2). The 143 CMOCs supporting the programme theory are organised according to the specific processes, activities, foundations, and cross-cutting factors for clarity of explanation, however, they are interdependent and interrelated. With the primary data, 55 CMOCs in existing parts of the programme theory and 40 CMOCs among the new parts of the programme theory were added. These new CMOCs bring breadth and depth to the programme theory and exhibit gaps in the literature filled by the primary data collection. We were unable to assess nine CMOCs from our Realist review based on the primary data, likely because they are more relevant to networks that are formed organically or from the bottom-up, while the case is an externally initiated and supported network.

NEST360 made significant investments in the network, however, they have invested in a way that has helped create committed, engaged, motivated, empowered, and confident network members who are changing practices around the collective vision. This is necessary because NEST360 may not achieve what they aim to with the tangible investments without the investment in the network. NEST360 has a theory of change describing the programme and its main activities, however, except for leadership and champions, the network processes, activities, foundations, and cross-cutting factors are not articulated in the theory of change. These network components are important for the successful implementation of NEST360’s programme activities, impact the success of the programme, and may sustain changes in practice that improve quality of care and service delivery for small and sick newborns.

Our findings resonate with earlier studies on networks in both HIC and LMIC settings, described below, as well as networks in other disciplines, such as public administration and education, which include culture, identity, shared belief, communication, leadership, commitment, problem orientation, and building relationships (Russell et al., 2017, Mandell and Keast, 2008). However, while the programme theory touches on some similar concepts as other studies on networks, it provides a deeper explanatory understanding of how and why network initiators, leaders, organisers, and members act to establish a network, which through its functioning and performing can support them to change practices to improve service delivery and quality of care.

A collective network vision or goal is common across different types of networks (Keast et al., 2004, Sheaff and Schofield, 2016, Russell et al., 2017, Britto et al., 2018). This may be articulated in the form of agreements, such as in examples of service delivery networks in the Philippines and Indonesia (Vergara et al., 2020, Hyre et al., 2019). Our programme theory and the underlying CMOCs and data contribute to the idea of a collective vision by explaining how a collective vision is developed, introduced, and communicated in a network; how network members commit to and sustain engagement with the collective vision; and how the network’s collective vision may be spread beyond the network.

While the activities that networks undertake to enact the processes throughout its evolution may differ across networks, the activities in the programme theory seem to be most common. Cross-learning and knowledge and skills sharing is done by many networks (English et al., 2017, Russell et al., 2017, Britto et al., 2018). The roles and activities of leaders and champions resonate across different examples of networks (Russell et al., 2017, Britto et al., 2018, McInnes et al., 2012, McInnes et al., 2015, Vergara et al., 2020). Our evaluation brings important nuances with regards to leaders and champions, particularly how to cultivate and sustain the influence of these important network roles and contributors.

Relationships are the foundation of networks; without taking the time for relationships to form and strengthen, networks would struggle. The importance of relationships in networks has been recognised by other studies (Keast et al., 2004, Carmone et al., 2020, Russell et al., 2017, Mandell and Keast, 2008, McInnes et al., 2012, Shawar et al., 2024). Our findings bring further insight into how relationships form, evolve, and adapt over a network’s evolution, for example, when network members have a shared professional identity, a common understanding of what is expected of them, the network provides resources that support their way of work, or network leadership has created a psychological safe space that enables cross-learning between network members. Other studies have not explored these nuances of network relationships in this degree of depth. Networks, therefore, offer a potential solution to an important health systems challenge: “health systems are inherently relational and so many of the most critical challenges for health systems are relationship problems.” (Kalaris et al., 2023b, Gilson, 2003)

Our evaluation findings highlight the importance of the energy, effort, and passion network members put into the network that is necessary for its evolution and without it networks may fail to get off the ground, adapt in the face of challenges or change, or be sustained. This finding is an important aspect that our evaluation brings to the network literature that has not previously been identified.

Fenton et al. (2001) raise some important challenges with networks that are reflected in the programme theory. When networks have group norms and a collective identity, they can “produce a collective blindness through an overconvergence of viewpoints.” (Fenton et al., 2001) This may be what we saw reflected in the data and the highly positive perspectives from many interviewees. Networks require financial resources, as well as time, and may struggle when certain resources are no longer available; (Fenton et al., 2001) this threat is acknowledged by the CMOCs and data. However, the CMOCs and data also highlight potential mitigating strategies that networks can take to alleviate these risks.

This Realist evaluation is on one specific type of network and there could be a risk that this inhibits the transferability of findings to other types of networks. However, the methodological approach of Realist evaluation enables the analysis of the behaviour of mechanisms that make NEST360 ‘work’ under different contexts and so any understanding developed could potentially be transferable to other countries, if the same mechanisms are also in operation (Pawson, 2013).

### 4.2 Strengths, limitations, and future directions

This is the first Realist evaluation, to our knowledge, on a network in a LMIC health systems. The evaluation has several strengths. First, we were able to test the programme theory with the primary data collected and evolve the understanding of how networks are initiated, form, function, perform, and sustain change and impact. We had sufficient data to test the programme theory, as we were able to confirm or refine 49 of the 58 CMOCs developed in our Realist review. The addition of new CMOCs within existing parts of the programme theory from our Realist review and new parts of the programme theory exhibit that there are gaps in the literature around understanding networks that we have addressed. We synthesised the results from a large amount of data and CMOCs into a framework that is applicable and practical to those interested in organising, leading, and studying networks. The revised programme theory is supported by 15 substantive organisational and sociological theories. These theories supported the identification and understanding of mechanisms and provided analogy. Nine of these theories support the Realist review programme theory, however, their prominence has shifted in the refined version. This is likely because of the type of network used to refine the theory.

There are several limitations to our evaluation. In terms of data collection, the majority of the interviewees had positive perspectives and seemed to have strong buy-in to NEST360. On the one hand this may reflect how the NEST360 network has engaged network members and generated commitment to the collective vision and the network; without this level of buy-in from members, networks may be less successful in forming, functioning, performing, and sustaining change and impact. However, this may have caused interviewees to not disclose negative events, experiences, or perspectives. There are some data that explain what has not worked as well in the network but, in general, there is limited critique on what does not. This is an area for future research as it is also a gap in the literature. Research is needed on understanding what does not work in networks, and how, why, and when they fail in part or whole (VanderWeert et al., 2022, Podolny and Page, 1998). The NEST360 network is made up of 13 hospitals and we interviewed participants from seven of those hospitals and visited four of them. Ideally, we would have interviewed people from all network hospitals, but the breadth and depth of collected data have partly mitigated this issue. We had initially planned to observe NEST360 meetings but likely due to the timing of data collection, we were only able to informally observe two. By not observing NEST360 meetings, we could not observe parts of the programme theory functioning and performing ‘in action.’ However, at the two meetings we informally observed, we observed cross-learning and knowledge and skills dissemination across the network.

At the time of data collection, the network had already formed and was functioning and performing but is now only beginning to work towards sustaining the change and impact the network enabled. Therefore, there is less understanding of how the processes, activities, foundations, and cross-cutting factors play out as networks work to sustain change and impact.

There are nine CMOCs from our Realist review that could not be tested by the primary data. This is likely because these CMOCs are more applicable to networks formed organically or from the bottom-up and so are an area for future research. The supporting substantive theories have mostly been developed, refined, and applied to Western high-income countries and so need to be applied carefully to other contexts. Future research could draw on theories from societies that are more collectivist in thinking, which has relevance for networks.

The revised programme theory can be tested on different types of networks, for example, ones formed organically or from the bottom-up, in different geographies, or focused in different clinical areas. Further research is needed on understanding the cross-cutting factors. Four new CMOCs in the revised programme theory lack supporting data but made logical sense in the programme theory and should be tested in future research. Additionally, our research focused on network initiators, organisers, and members; there is a lack of research into how patients, clients, or caregivers experience change (or not) when facilities join a network and what influences their experiences. According to a recent World Bank report, Hospitals in Health Systems, and an analysis on financing networks of care, more research is needed on approaches to financing networks in LMIC health systems (Chopra et al., 2024, Villalobos-Dintrans et al., 2023).

Much of the existing network research is focused on assessing clinical outcomes and quality of care. It would be useful for stakeholders to know if a network is performing as intended. However, given their complexity, how could this be done in a way that meaningfully captures performance from differing stakeholder perspectives? Therefore, a future avenue for the programme theory is to explore and identify performance indicators to understand how networks are effective or successful in these relational areas and to make recommendations for practice.

Trust was not as prominent in the primary data to the extent anticipated. Other studies have reported that trust in networks and how it evolves needs to be better understood (Provan and Kenis, 2007). Trust may be an emergent property of the programme theory, it may be latent, or not something explicitly talked about. Furthermore, while psychological safety emerged as one of the network foundations in the programme theory, it differs from trust as it has more relevance for small groups, like a network, and the degree of interpersonal safety they experience, including a “sense of how valued and comfortable an employee feels in that work setting.” (Edmondson and Trust, 2004) Although both trust and psychological safety include perceived vulnerability, this is narrower for psychological safety than trust (Edmondson and Trust, 2004). If trust did not exist in networks, networks logically would be much less likely to achieve what they have for health system functioning, quality of care, and clinical outcomes; therefore, trust may be implicitly fostered through shared aspects of networks. Another reason why trust may not appear in the literature and primary data more prominently and explicitly is that trust in networks may be “a silent background, sustaining the unproblematic, smooth running of cooperative relations.” (Misztal, 1996) Alternatively, due to the group nature and the creation of psychological safety, trust as a concept may be less prominent in networks because the relationships exist at the organisational or group level and less between individuals, though direct interpersonal relationships exist as well.

The programme theory did not explicitly touch on power and power dynamics. Power, like trust, may be latent, and can often be subjective. The role that power plays in networks is under researched; (Addicott and Ferlie, 2007) power dynamics may be relevant for networks with regards to leadership, relationships, and creating psychological safety. A systematic review on clinical network effectiveness found that power imbalances and the limited power of network members were barriers to network success (Brown et al., 2016). Non-health care networks highlight that informal power from interpersonal relationships can be more important than formal power, signalling the importance of leadership in networks to manage power dynamics (Keast et al., 2004).

Although power did not emerge strongly in the primary data and in the programme theory, we can infer that power dynamics are likely at play within different parts of the NEST360 network. Power dynamics exist between the network organisers and the network hospitals, as well as within the network hospitals, potentially between the hospital management and NBU or the NBU and other departments; for example, because the NBU is seen receiving particular support from NEST360. One of the challenges that the NEST360 network had were changes in the partners that make up the network organisers; some of these changes may have been due to power dynamics. The different power dynamics might have come out stronger through different data collection methods, such as observations. In data collection, KK’s position as a non-native researcher may have made her less sensitive to subtle power dynamics at play. Exploring the roles of trust and power and why and how (or why not) they function in the programme theory are area for future research.

### 4.3 Conclusion and recommendations

The refined programme theory (Fig. 1 & 2) provides those interested in or working in a network with details on the processes, activities, foundations, and cross-cutting factors to consider for a network throughout its evolution. This can lead to a more considered implementation of networks, thereby improving health system functioning and performance. Forming a network takes work and a level of effort and energy will be required over network evolution. Many parts of the programme theory may be implicit knowledge to those who have started or are part of a network, but the existing literature lacks explicit explanations as to why these are rational things to do. The following recommendations provide an initial set of considerations for those interested in forming, working in, or evaluating a network.

1. *Ensure sufficient time and resources are dedicated to network formation - Forming a network takes time, effort, and energy; passion helps too.*
2. *Develop and deploy activities to develop a collective vision and to rally potential members around this - The development and buy-in to a collective vision are important and play a key role as the network evolves.*
3. *Provide suitable opportunities for relationship building for network members - Purposeful and intentional relationships, linkages, and partnerships are key, differentiating a network from the health system.*
4. *Ensure processes are in place to develop psychological safe spaces for network members - A psychological safe space may be a key facilitator to network members being able to change practices.*
5. *Develop a plan to sustain the network - A network needs to plan for sustainability, focused on the changes and impact it wishes to sustain, from the beginning.*
6. *Put in place plans and processes that enable the network to adapt to internal and external changes - Networks need to be adaptable throughout their evolution, including around their vision and goals; external forces can limit network evolution.*

## Funding

KK received funding from Kellogg College (Travel Fund and Research Support Grants), University of Oxford for this research. ME is funded by a Senior Research Fellowship from the 10.13039/100004440Wellcome Trust (#207522). GW receives funding from a range of Funders - see https://orcid.org/0000–0002–5384–4157 for a full list. ME & GW also receive salary support from the National Institute for Health Research (10.13039/100006662NIHR)(NIHR130812): Learning to Harness Innovation in Global Health for Quality Care (HIGH-Q) grant using UK aid from the UK government to support global health research. The views expressed in this publication are those of the author(s) and not necessarily those of the NIHR or the UK government. Additional financial support came from a grant to the NEST360 program from the 10.13039/100000870John D. and Catherine T. MacArthur Foundation, the 10.13039/100000865Bill & Melinda Gates Foundation, ELMA Philanthropies, and The Children’s Investment Fund Foundation UK under agreements to William Marsh Rice University with a sub-agreement to ME through the 10.13039/501100000769University of Oxford Centre for Tropical Medicine and Global Health.

## CRediT authorship contribution statement

**Geoff Wong:** Writing – review & editing, Visualization, Supervision, Methodology, Formal analysis, Conceptualization. **Katherine Kalaris:** Writing – review & editing, Writing – original draft, Visualization, Methodology, Funding acquisition, Formal analysis, Data curation, Conceptualization. **Mike English:** Writing – review & editing, Visualization, Supervision, Project administration, Funding acquisition, Formal analysis, Conceptualization.

## Declaration of Competing Interest

The authors declare the following financial interests/personal relationships which may be considered as potential competing interests: KK was a consultant for the World Health Organization on networks of care for maternal and newborn health from 2021 to 2024. This Realist evaluation was part of her doctoral work at the University of Oxford, supervised by GW and ME. ME established the Clinical Information Network in Kenya and received funding for this research platform.

## Footnotes

Appendix A Supplementary data associated with this article can be found in the online version at doi:10.1016/j.ssmhs.2025.100101.
