# Supplementary material for: Understanding the initiation, formation, functioning, and performing of networks to change practices – Realist evaluation of a programme to improve newborn care in Kenya
Source: SSM Health Syst. 2025 Dec;5:100101. doi: 10.1016/j.ssmhs.2025.100101 (PMC12678620; doi:10.1016/j.ssmhs.2025.100101)
Supplement: Supplementary file 1 — Supplementary material [file mmc1.docx]

# Appendix A. Developing an understanding of the processes involved in the formation and functioning of networks to change practices: learnings from a Realist Evaluation of the Newborn Essential Solutions and Technologies programme in Kenya: protocol

## Abstract

Networks are becoming a more frequently used approach to solve problems of poor service delivery, quality of care, clinical outcomes, and health system functioning in low-and-middle income countries. While networks may undertake standard health system strengthening activities, there are certain less tangible but very key processes and interactions that occur in networks which enable the activities to happen. The ability to articulate these less tangible elements of a network are important to understanding how and why networks form and work. A realist review developed a programme theory that explained how and why networks form and function to change practices. Following on this realist review, a realist evaluation will aim to further uncover and understand the processes involved in the formation, functioning, and performing of an internationally supported programme network in an LMIC health system that has led to changes in practice to improve quality of care and services. This realist evaluation will confirm, refute, or refine the programme theory through a multiple-methods single embedded case study of the Newborn Essential Solutions and Technologies programme in Kenya. Data collection methods will include semi-structured realist interviews, document review, and non-participant observation. The findings from this realist evaluation will enable a greater understanding of the processes involved in network formation, functioning, and performing that enable changes in practices in internationally supported programme networks. This can lead to a more considered implementation of these networks, thereby improving health system functioning and performance.

## Background

### Networks in health systems

Health systems are complex systems and their sub-optimal functioning due to increased demands, limited resources, and structural inertia^1^ may lead to poor service delivery, quality of care, and clinical outcomes. Health sector actors look to approaches to improve under-performing systems, services, and the resultant poor outcomes that are within their capabilities and resources. Networks are an increasingly frequent approach to solve these problems.^2^

A vast and diverse range of networks exist in the field of healthcare; they range from global advocacy networks to service delivery networks to community-based networks. While there are various network definitions, typologies, and frameworks in the health and non-health literature, there is “no single, consensually agreed definition of what constitutes a ‘network.’”^3^ For a scoping review on networks in low-and-middle income country (LMIC) health systems, we defined networks as “groups of facilities and/or healthcare stakeholders (including but not limited to all types of providers, technicians, government officials, professional associations, non-governmental organisations, and donors) linked formally or informally, horizontally or vertically, through programs, interventions, activities, or initiatives.” The scoping review identified five key components that make up networks (form and structure, governance and leadership, method of functioning, resources, and communication) and mapped network uses, purposes, and stakeholders.

While the activities networks undertake are often similar to other health system strengthening initiatives, there are certain less tangible but very key processes and interactions that occur in networks which enable the activities to happen. The ability to articulate these less tangible elements of a network are important to understanding how and why networks form and work. If those interested in establishing networks are able to deliberately take these considerations into account, it could support achieving the desired network outcomes. This evaluation seeks to learn transferable lessons about how networks (as defined above) form, function, and perform in order to change practices. Data will come from the Newborn Essential Solutions and Technologies (NEST) programme in Kenya (see below for more details).

### Kenya

Despite substantial declines in global child mortality over the last 30 years, significantly less progress has been made in the reduction of newborn deaths. Globally, in 2018, newborn deaths made up 47% of all under-5 deaths.^4^ In Kenya, while under-5 mortality notably reduced between 2003-2014 (from 115 to 52 deaths/1000 live births), newborn deaths only declined by one-third (from 33 to 22 deaths/1000 live births).^5^ 40% of all under-5 deaths occur during the newborn period^6^ and over 50% of sick newborns lack access to a health facility able to provide appropriate care.^7^ Improvements in the availability and quality of newborn care are essential to reduce newborn mortality as 71% of newborn deaths could be reduced with high-impact low-cost interventions.^8^

### Newborn Essential Solutions and Technologies

NEST is a multi-country multi-stakeholder programme in Kenya, Malawi, Nigeria, and Tanzania which has the goal of implementing high-quality, safe, inpatient care for small and sick newborns.^[[1]](#footnote-1)^ The programme works with governments, bi and multilateral organisations, global health donors and organisations, civil society, academic institutions, and the private sector. NEST provides in-patient newborn wards in hospitals across the four countries with a bundle of medical devices for the care of small and sick newborns (e.g., CPAP machines, radiant warmers) and supports their installation and maintenance, updates pre-service and in-service educational materials for clinicians and biomedical engineers and technicians, and implements tools and dashboards for data management and quality improvement. The programme also works globally to improve market access for these medical devices and develop global materials, such as an investment case, cost planning tools, and an Implementation Toolkit for small and sick newborn care.

In Kenya, NEST supports 13 hospitals in 11 counties. NEST is an example of a network initiated by external organisations with aims to engage and empower national and country level partners. It is a large, complex programme with international financial support to build networks. NEST takes an approach of trying to build networks at the country and facility levels amongst clinicians, biomedical engineers and technicians, the Ministry of Health (MoH), and other stakeholders. By doing this, it affords the opportunity to develop committed, engaged, and motivated network members to change practices, transfer knowledge, and sustain results.

Over the past three years of the NEST programme, in collaboration with the MoH, NEST has leveraged opportunities to develop networks between clinicians to build clinical capacity, highlighted in Table 1.

| **Activity** | **Materials** |
| --- | --- |
| **Quarterly clinical mentorship and supportive supervision:** A clinical training team trains and provides quarterly mentorship and supportive supervision to clinicians. The training includes opportunities for clinicians to practice together in simulation situations. This has created a network of newborn care champions in hospitals across the country. | https://nest360.org/kenya-holds-gic-for-pre-service-and-clinical-mentorship/ |
| **Adaptation of clinical mentorship:** During Covid-19, these trainings were moved to webinars open to all healthcare workers. To ensure accessibility to frontline healthcare workers, in particular, the clinical training team undertook outreach on using Zoom, by leveraging existing WhatsApp groups to provide instruction on how to use the platform. |  |
| **Adaptation of in-service training:** The clinical training team built on the success of these webinars to transform in-person newborn care trainings into a three-day virtual training for facilities during Covid-19. | NEST Clinical Modules  <https://nest360.org/project/clinical-modules/>  NEST Clinical Scenarios  <https://nest>360.org/project/clinical-scenarios/ |
| **Integration of NEST materials into the national curricula:** NEST clinical training integrated into the Newborn Emergency Triage Assessment and Treatment (ETAT) course |  |
| **Pre-service training and training materials for biomedical engineers and technicians:** Biomedical training and training of trainers at Kenyatta University on repair and maintenance of NEST technologies | NEST Technical Modules  <https://nest360.org/project/technical-modules/>  NEST Technical Scenarios  <https://nest>360.org/project/technical-scenarios/ |
| **Clinical – Biomedical linkages:** NEST has created linkages between clinicians and biomedical engineers and technicians, which in many cases may have not previously been that strong. |  |
| **Generic instructor courses:** Training of potential clinical and non-clinical healthcare professionals as instructors | Generic Instructor Course  https://nest360.org/project/gic/  https://nest360.org/kenya-holds-gic-for-pre-service-and-clinical-mentorship/ |

Table 1: Examples of NEST clinical and relationship building activities

A Committee of Experts, led by the Kenya MoH’s Department for Family Health and includes partners such as USAID, Unicef, and Path, guides NEST implementation and leverages learnings from NEST to inform changes to national policy. These quarterly meetings have helped facilitate integration of NEST developed educational materials into national curricula and the adoption of a standard neonatal unit layout.

NEST has a theory of change that describes the programme and its main activities across the four countries. However, processes of network formation and functioning are not articulated in the theory of change and are likely country specific. These complex processes of network formation and functioning are important for successful implementation of NEST’s programme activities, impact the success of the programme, and sustain (or not) changes in practice that improve quality of care and service delivery for small and sick newborns. This realist evaluation will attempt to articulate and understand what these processes of network formation and functioning are and how and why they facilitate NEST to achieve its goals.

While NEST is implemented across four countries, this evaluation will only focus on the NEST programme in Kenya. This is to achieve sufficient depth in the data collection and analysis that would be difficult to reach across the four countries without extensive resources and time. We will aim to achieve maximum variation in the NEST sites selected. The methodological approach of realist evaluation lends itself to analysis at the middle range theory level of abstraction and therefore transferable lessons can still be identified from data collection in one country. Additionally, there are pre-existing relationships with stakeholders and the MoH in Kenya, which will facilitate access to hospitals, interview participants, and meetings as well as logistics.

### Realist Evaluation

The realist evaluation approach seeks to understand causation in phenomena, such as social programmes, initiatives, or interventions.^9^ Realism acknowledges that actors respond differently to interventions in diverse circumstances and that programmes are not universally successful, working better in certain contexts than in others. According to the realist philosophy of science, patterns of observed outcomes are the result of underlying generative mechanisms, that may or may not function in certain contexts.^9^ The approach is regularly used to make sense of complex social programmes or interventions.^10^

All programmes and interventions have underlying theories explaining how and why they produce certain outcomes which may or may not be explicit.^11^ Realist programme theories explain what works, for whom, in what respect, to what extent, and how using the concepts of context, mechanism and outcome (see below for more details). They are a form of ‘middle range theory’ because they are specifically expressed at the level of abstraction that permits empirical testing.^12^

The realist approach to evaluation helps to develop an understanding of how and why things work and in what context and cause various outcomes in different circumstances, which can have broader implications for the implementation of social programmes, initiatives, or interventions. A realist evaluation tests (i.e., confirms, refutes or refines) programme theories with primary data collection. Data collected is interpreted to identify what mechanisms are triggered in what contexts to produce the observed outcomes; the mechanisms are key to understanding how the outcomes are produced.^13^ Mechanisms are the interaction between the resources provided by a programme or intervention and how actors interpret and act upon the resources; mechanisms are always present but their operation is context dependent.^14^ Contexts are elements of the programme or intervention environment that may or may not trigger the mechanism.^15^ Data on context, mechanisms, and outcomes are formed into Context – Mechanism – Outcome Configurations (CMOCs) to show which mechanisms are triggered by which context to produce certain outcomes within the programme theory.

### Rationale

This realist evaluation builds on a scoping review on networks in LMIC health systems and a realist review that developed a programme theory on how networks are initiated, form, and change practices. The realist review drew on studies on networks in LMIC health systems and a selection of relevant High-Income Country (HIC) literature and substantive theories to develop and refine the CMOCs contained within the initial programme theory. This literature-based programme theory, which looked across many different types of networks is therefore relatively generic. The realist evaluation will test the programme theory from the realist review to confirm, refine, or refute it using primary data for an internationally supported programme network exemplified by NEST. From the refined programme theory recommendations will be drawn for developing and implementing these types of networks in LMIC health systems.

Health systems are complex systems “composed of many interdependent, heterogenous parts that self-organize and co-evolve.”^16^ Networks, implemented in a complex system, can be considered complex interventions because they are made up of many interacting components.^17^ In the Science of Evaluation (2013), Pawson describes the realist perspective of complexity through seven concepts: volition, implementation, context, time, outcome, rivalry, and emergence (i.e. VICTORE).^18^ Therefore, as the realist approach is often employed to make sense of complex interventions and programmes, networks are a suitable candidate for the realist approach to research. Table 2 applies these concepts to networks in health systems to exemplify how they meet Pawson’s criteria of complex interventions.

| **Concept** | **Explanation** | **Application to networks in health systems** |
| --- | --- | --- |
| Volition | People make choices | Networks are composed of stakeholders who make decisions on what, how, and when to implement activities to set-up and run a network. Stakeholders can be an individual, institution, or organisation. In a network a stakeholder may be represented individually as well as with an institution or organisation. |
| Implementation | Programmes have long implementation chains | There may be many different activities, relationships, and steps involved to implement and run a network, and they may occur over time. |
| Context | Pre-existing from micro to macro levels, influences outcomes | Networks involve all levels and sectors of the health system, and these have different contexts which can affect the network in different ways. |
| Time | Programmes and interventions come from somewhere | Networks are not a new intervention in health systems and there is relevant existing research that can be drawn on to understand them. Networks are often developed from or linked to prior relationships or existing structures. |
| Outcome | Desired, undesired, predictable, unpredictable, intermediate | Networks can support the production of clinical outcomes, health system outcomes, and network specific outcomes. These outcomes may be intended or an unintentional biproduct of the network. |
| Rivalry | Interactions with other programmes or interventions | A multitude of interventions and programmes occur in health systems; networks are not implemented in isolation. |
| Emergence | Things change | Health systems and networks are dynamic and subject to change. |

Table 2: Explanation of networks in health systems as complex interventions, using Pawson’s VICTORE framework

### Study aims and objectives

#### Study aim

The aim of this study is to uncover and understand the processes involved in the formation, functioning, and performing of internationally supported programme networks in LMIC health systems that have led to changes in practice to improve quality of care and services. This study will undertake a multiple-methods case study of the NEST programme in Kenya to explore if there have been changes in practice, what they are, what has enabled the changes in practices and whether the programme theory (from the realist review) is able to explain why, in what context and for whom these happened. Where necessary modifications to the programme theory will be made based on the data collected.

#### Study objectives

1. Test (confirm, refute, or refine) the realist programme theory (developed in preceding realist review) on the NEST programme in Kenya to develop an understanding of the processes involved in the formation, functioning, and performing of the programme’s network that enable changes in practice to improve quality of care and services
2. Use the refined programme theory to identify transferable lessons and best practices for ministries of health, health system managers, clinicians, and health sector partners interested in establishing or scaling up networks, highlighting how networks can change practices to improve quality of care and services

#### Research questions

Using NEST in Kenya as a case study,

1. What are the changes in practice in NEST’s programme network?
2. What are the processes involved in the formation, functioning, and performing of the programme’s network that enable changes in practices to improve quality of care and services?
3. How do the processes involved in the formation, functioning, and performing of the programme’s network enable changes in practices to improve quality of care and services?
4. Why do the processes involved in the formation, functioning, and performing of the programme’s network enable changes in practices to improve quality of care and services?
5. What are the mechanisms in the processes involved in the formation, functioning, and performing of the programme’s network that enables changes in practices to improve quality of care and services?
6. What contexts trigger the mechanisms in the processes involved in the formation, functioning, and performing of the programme’s network that enables changes in practices to improve quality of care and services?

## Study Design and Methods

### Study design and setting

This realist evaluation will be a multiple-methods single embedded unit case study to confirm, refute, or refine the programme theory. The evaluation will take place in and around Nairobi County, Kenya. A case study is an in-depth exploration of a complex phenomena in its environment.^19^ The case study approach in a realist evaluation allows for refining the programme theory across contexts and deepening an understanding of causal processes.^20^ The NEST programme in Kenya is considered the case and NEST stakeholder organisations and 2-4 hospitals as embedded units. Data collection methods will consist of semi-structured realist interviews, programme document review, and non-participant observation. Analysis of the data will confirm, refute, or refine CMOCs from a previous realist review or develop new ones and the programme theory. The evaluation will run from January - December 2023.

### Phase I: Initial programme theory development

A programme theory was developed through the realist review “Developing an understanding of networks in LMIC health systems: how and why clinical and programmatic networks improve and provide high-quality healthcare and services: a realist review.” This review developed and refined a programme theory that explains how and why networks in health systems are initiated and form in a way that sets them up to be able to change practices, leading to improved quality of care and service delivery. The review selected 32 pieces of peer-reviewed and grey literature and nine substantive theories to develop and refine 58 CMOCs and refine the programme theory.

The realist review programme theory is presented in Figure 1; the programme theory provides an explanation of how and why networks are initiated in a way that sets them up to be able to change practices. The formation of a network starts with the identification of a problem by health system actors. This problem, in service delivery or health system functioning, causes a strain among health system actors that triggers an energy to work towards improving the identified problem. Health systems actors realise that there are others who feel the same strain and that they can pool their energy to collectively take action toward solving the problem. With similar perspectives and experiences, the potential network members are able to coalesce around a collective vision. Network members will form or strengthen existing purposeful relationships, linkages, and partnerships. These relationships, linkages, and partnerships differentiates the network from the health system. Network leadership emerges early in the formation of the network and evolves as the network matures. Overtime, a network identity and culture begin to emerge. Network members become committed, engaged, and motivated to the network’s collective vision, identity, and culture and the network becomes a psychological safe space.

The combination of the network, as a psychological safe space, and the network members as committed, engaged, and motivated enables them to act with accountability and trust allowing for improved teamwork, communication, coordination, and collaboration. This facilitates changes in practices to improve quality of care, service delivery, and health system functioning and ultimately clinical outcomes. However, there are many outside factors that may prevent changes in practices, the provision of care, or outcomes that are beyond the network members’ control.


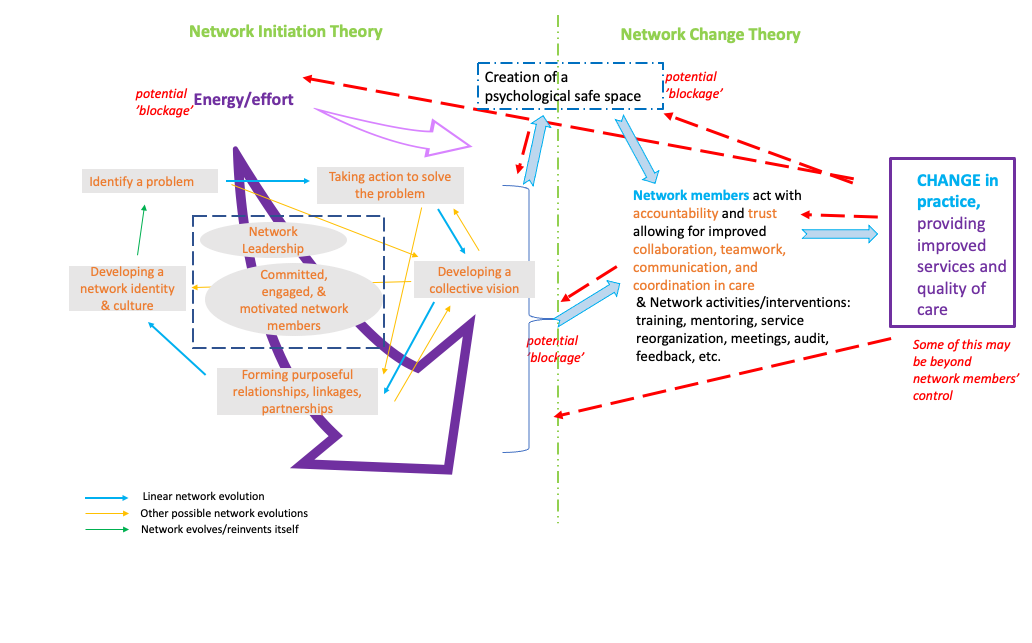


### Figure 1: Realist Review Programme theory

### Phase II: Programme theory testing

The process of testing the programme theory will aim to uncover and understand the processes involved in the formation, functioning, and performing of NEST as a network. Data collection will centre around the different phases of the programme theory, providing data to further support, refine, or refute CMOCs developed during the realist review and the programme theory. During data collection, it may become apparent that certain parts of the programme theory are particularly important, and the evaluation will progressively focus on those areas. It may as well become apparent that there are certain phases of the programme theory that are not happening, and it will be important to understand why.

#### Data collection and sampling

This study will include three methods of data collection: semi-structured realist interviews, programme document review, and non-participant observations. A fieldwork journal will be kept to document reflections and to take into account positionality and potential biases. This is particularly important in realist research as, unlike with qualitative constructivist interviews, the interviewer abandons neutral territory in order to engage with participants.^21^ Data collection will be done in batches to provide time for preliminary analysis and reflection, which may progressively focus the evaluation and target subsequent interviews and observations until theoretical saturation is reached.

1. **Semi-structured realist interviews:** The anticipated main source of data will be collected using semi-structured realist interviews. Questions for realist interviews are based on the programme theory to understand how, where, when, and why the programme is and is not effective. Researchers draw on the experiences from study participants because they can provide information on programme processes and outcomes. Realist interviews are structured in a ‘teacher-learner cycle’ in which participants respond to the programme theory or part of to either confirm, refine, or refute the theory.

An interview guide will be developed and will include the following types of questions: 1) participant’s role in, experiences with, and views of the programme; 2) examples of participant’s specific experiences of the programme that targets different aspects of the programme theory; and 3) test specific elements of the programme theory. Early interviews will be more exploratory and become more specific throughout the data collection process; possibly reinterviewing participants interviewed earlier on in the data collection process. Interview questions will evolve as we understand the case in greater depth and fieldnotes and post-interview reflections will be used to identify areas that seem most relevant to explore in later interviews.

Participants will be purposively sampled and will include NEST researchers and implementers based internationally and in Kenya, programme stakeholders, Kenya MoH representatives, biomedical engineers and technicians affiliated with NEST, hospital administrators, and Newborn Unit clinical and non-clinical staff. Purposively sampling enables the selection of information rich cases which best provide insights relevant to the research questions.^22^ Interviews will be organised in waves starting with NEST researchers and implementers based internationally and in Kenya, followed by programme stakeholders, then the Kenya MoH, and then clinical, managerial, and administrative staff and biomedical engineers and technicians in NEST sites. As we begin to understand the case to a greater degree, the sampling will be informed by parts of the programme theory to be able to test it. Individuals will be targeted for recruitment with guidance from the project managers, investigators, and research officers on the Learning to Harness Innovation in Global Health for Quality Care (HIGH-Q) project at the KEMRI Wellcome Trust Research Programme (KWTRP), implementing the study Evaluating the effects of technology and workforce enhancement to support neonatal hospital care in Kenya (HIGH-Q Kenya team). As much variation as possible will be sought from the types of interviewees to provide the contrasts necessary to draw out learnings. Hospital sites will be selected based on pre-defined criteria, included in Table 3.

| **Criteria** | **Rationale** |
| --- | --- |
| Part of NEST and HIGH-Q (non-workforce intervention hospitals) | NEST/HIGH-Q programme documents |
| Urban – rural | Kenya MoH classification |
| Well – poor performing | NEST health facility assessments |
| Likely to have relevant information | Guidance from HIGH-Q Kenya team |

Table 3: Pre-defined selection criteria for hospitals

Interview participants will hopefully be able to provide relevant information in respect to different aspects of the programme theory. The number of participants will be based on the concept of Information Power – the more relevant information in a sample, the few participants needed.^23^ It is anticipated up to 40 interviews will be undertaken at hospitals and 10 with NEST researchers and implementers based internationally and in Kenya, programme stakeholders, and Kenya MoH representatives. Interviews will be recorded and transcribed. Researcher’s reflections will be captured in a fieldwork journal and transcribed. This source of data will try to uncover the processes behind the formation, functioning, and performing of NEST as a network and how participants understand and see the network working.

1. **Document review:** Programme documents and reports will be identified from NEST globally and specific to Kenya as well as relevant strategic and operational documents from the Kenya MoH. Programme documents and reports will include hospital quality improvement reports, annual NEST programme reports, and quarterly NEST newsletters. Meeting minutes from NEST Kenya meetings will also be sought for inclusion. This source of data will provide background information on the NEST programme, particularly to provide an understanding of how the programme was planned to work, as well as important context on the Kenya health system to understand how the programme fits into the health system, shining some light on its complexity.
2. **Non-participant observations:** Non-participant observations will be undertaken at 5-7 NEST meetings online and in-person. These may include quarterly national multi-stakeholder group newborn meetings, quality improvement meetings with facilities, and other NEST multiple stakeholder meetings. The selection of meetings will be purposeful and based on potential relevance to the programme theory. Meetings will be identified and selected with guidance from the HIGH-Q Kenya team. This source of data will provide insights into interactions and communication between the different network actors and how they work together; looking particularly for information that relates to aspects of the programme theory or new insights not identified from the realist review. Observer notes will be made by hand and transcribed following the meetings.

#### Data analysis

Interview transcripts, programme documents, and notes from interviews and non-participant observations will be imported into NVivo QRS International, a data management software tool for qualitative analysis. The data analysis process will be iterative. Interview transcripts, programme documents, and notes from interviews and non-participant observations will be reviewed and analysed in waves, starting with data that is most likely to hold the most relevant information. Data will be coded deductively with codes developed from the programme theory and CMOCs from the realist review and inductively coded with new codes that emerge from the data that are relevant to the research questions. Coded data will be reviewed and organised according to parts of the programme theory and CMOCs developed during the realist review. Data will be used to further support, refine, or refute the previously developed CMOCs or to develop new CMOCs, employing retroductive reasoning. Retroduction, which falls between inductive and deductive reasoning, is the process of identifying the mechanisms behind the observed pattern of outcomes.^24^

### Phase III: Theory consolidation

The programme theory will be iteratively refined with CMOCs that have been revised or developed based on interpretations from the primary data collected. Substantive theories supporting the programme theory in the realist review will be reconsidered for relevance to the refined programme theory and additional substantive theories will be considered if necessary. Furthermore, based on the findings from the evaluation, modifications to the substantive theories may be suggested.

### Ethics and research governance

This research falls under the ethics approval of the study Evaluating the effects of technology and workforce enhancement to support neonatal hospital care in Kenya. The study has ethical approval from the Oxford Tropical Research Ethics Committee at the University of Oxford (OxTREC Reference 26-21) and the KEMRI Scientific and Ethical Review Unit (Protocol No. KEMRI/SERU/CGMR-C/229/4203). Written informed consent will be obtained from all participants interviewed and from participants in the observed meetings. Interview and observation data will be anonymised, all identifiers removed, and participants will have unique study codes.

### Data Management

Interviews will be audio recorded with an encrypted digital voice recorder and will be uploaded to a secure and encrypted file space at KWTRP or University of Oxford and deleted from the recorder. Recordings will be sent to a transcription service in batches to be transcribed verbatim. Recordings will be archived and destroyed after the study has ended.

Field notes from interviews and observation notes will be anonymised. A separate document containing any identifiers will be kept in a separate KWTRP or University of Oxford secure location. Notes will be typed or scanned into an electronic format and stored on secure KWTPR or University of Oxford servers; paper documents will subsequently be shredded.

All files will be stored on secure servers at the KWTRP or the University of Oxford accessible only to specific researchers with password-protected access. This evaluation will follow the data management plan of the HIGH-Q project.

### Communication and dissemination of results

The findings from this realist evaluation will be written-up according to the Realist and Meta-narrative Evidence Syntheses (RAMESES) for Realist Evaluation and submitted for publication in a peer-reviewed journal. In addition, a brief on the key findings and transferable lessons and recommendations from the final programme theory or other types of materials may be developed. Dissemination of the results will be targeted at stakeholders in Kenya and to the broader NEST programme, as well as other government and organisation stakeholders who have implemented or are interested in implementing a network in their health system context. This may be done through presentations, small group or individual discussions, or sharing of materials (e.g., brief, summary slide deck, publication).

## Discussion

### Importance of the research

Networks are an increasingly employed approach to improve health systems functioning and performance, service delivery, and quality of care, particularly in LMIC health systems. They potentially offer a solution to an important health systems problem: “Health systems are inherently relational and so many of the most critical challenges for health systems are relationship problems.”^25^ The findings from this realist evaluation will enable a greater understanding of the processes involved in network formation, functioning, and performing that enables changes in practices in internationally supported programme networks. This can lead to a more considered implementation of these networks, thereby improving health system functioning and performance. Furthermore, this evaluation will contribute to the growing literature on networks in LMIC health systems and is the first realist evaluation, to our knowledge, specifically focused on a network in a LMIC health system. The refined programme theory provides an opportunity for additional research to test the theory in other contexts and on other types of networks.

### Limitations and risks

In undertaking this realist evaluation, there are methodological limitations and challenges that need to be considered. In terms of data collection, there may be challenges with identifying a sufficient number of available and willing participants for interviews. This will be mitigated by relying on the guidance of the HIGH-Q Kenya team and taking a broad approach to identifying people who would fit the different profiles of interviewees. It will also require flexibility and adaptability on the part of the interviewer. Secondly, there may be a risk that meeting organisers and participants are reluctant to have meetings observed. Furthermore, the presence of an observer may have a stifling effect on the discussion causing some of the unarticulated aspects of processes that we are looking for to be altered or not appear as easily. In an effort to mitigate concerns of meeting organisers and participants, the observed meetings will not be recorded and observation notes will be anonymised. Prior to data collection familiarisations visits will be undertaken at the hospitals which will also assist with understanding of the context. Lastly, this study is only able to look at one of the four NEST countries, which may limit transferability of recommendations to other settings.

Reflections from Gilmore (2019) on the challenges of undertaking a realist evaluation as a foreign researcher identified four main concerns that merit taking into consideration: 1) power imbalances between researchers and participants during data collection; 2) working through translators and across languages; 3) being ‘engaged’ with limited contextual familiarity; and 4) bringing in appropriate substantive theories.^26^ Recommendations to counter these challenges potentially relevant to this evaluation are summarised in Table 4. Interviews are planned to be conducted in English and observed meetings will likely be as well, so translation (challenge #2) is not anticipated to be necessary.

| **Challenge** | **Recommendations** |
| --- | --- |
| 1) Power imbalances between researchers and participants during data collection | - Employ standard high-quality qualitative interview techniques - Avoid overly technical language and consider how the structure of the interview is presented - Include open interviews before theory testing interviews - Be reflexive about the interview process and consider how power might have played a role and bring this into the analysis (fieldwork journal) |
| 2) Working through translators and across languages | - Document decision-making processes and reflexivity (fieldwork journal) - Iteration is very important - Make sure that meaning and explanations are not lost during translation - Share findings to translators or research assistants - Interview translators as research participants following data collection |
| 3) Being ‘engaged’ with limited contextual familiarity | - Spend as much time as possible in the research setting before and during the study - Review relevant literature from other disciplines - Draw on insights into the context of the study from local collaborators - Actively engage with the surroundings |
| 4) Bringing in appropriate substantive theories | - Iterative theory development - Avoid bringing in theories based on the study of western, educated, industrialised, rich, democratic people early in the research process but use them to help explain findings and place within the wider literature |

Table 4: Adapted from Challenges and recommendations for undertaking a realist evaluation as a foreign researcher, Gilmore (2019)

## References

1. Rossiter M, Verma J, Denis J, et al. Governing Collaborative Healthcare Improvement: Lessons from an

Atlantic Canadian Case. *International Journal of Health Policy and Management* 2017;6(12):691-94. doi: 10.15171/ijhpm.2017.60.

2. Sibbald S, Schouten K, Sedig K, et al. Key characteristics and critical junctures for successful Interprofessional

networks in healthcare - a case study. *BMC Health Services Research* 2020;20 doi: 10.1186/s12913-020-05565-z

3. Aveling E, Martin G, Armstrong N, et al. Quality improvement through clinical communities: eight lessons for practice. *Journal of Health Organization and Management* 2012;26(2)

4. Global Health Observatory: World Health Organization; [Available from: <https://www.who.int/gho/child_health/mortality/neonatal/en/>.

5. Kenya Demographic and Health Survey Kenya National Bureau of Statistics, 2014.

6. Aluvaala J, Nyamai R, Were F, et al. Assessment of neonatal care in clinical training facilities in Kenya. *Arch Dis Child* 2015;100(1):42-47. doi: 10.1136/archdischild-2014-306423

7. Murphy G, Gathara D, Abuya N, et al. What capacity exists to provide essential inpatient care to small and sick newborns in a high mortality urban setting? - A cross-sectional study in Nairobi City County, Kenya. *PLoS One* 2018;13(4) doi: 10.1371/journal.pone.0196585

8. Nzinga J, McKnight J, Jepkosgei J, et al. Exploring the space for task shifting to support nursing on neonatal wards in Kenya public hospitals. *Human Resources for Health* 2019;17(1) doi: 10.1186/s12960-019-0352-x

9. Greenhalgh T, Pawson R, Wong G, et al. Realist evaluation, realist synthesis, realist research - what’s in a name? The RAMESES II Project, 2017.

10. Pawson R, Greenhalgh T, Harvey G, et al. Realist review - a new method of systematic review designed for complex policy interventions. *Journal of Health Services Research & Policy* 2005;10:21-34.

11. Pawson R. Evidence-based policy: a realist perspective Sage 2006.

12. Greenhalgh T, Pawson R, Wong G, et al. “Theory” in realist evaluation. The RAMESES II Project, 2017.

13. Pawson R, Tilley N. Realistic Evaluation Sage 1997.

14. Greenhalgh T, Pawson R, Wong G, et al. What is a mechanism? What is a programme mechanism? The RAMESES II Project, 2017.

15. Greenhalgh T, Pawson R, Wong G, et al. What realists mean by context; or, Why nothing works everywhere or for everyone. The RAMESES II Project, 2017.

16. Lanham HJ, Leykum LK, Taylor BS, et al. How complexity science can inform scale-up and

spread in health care: understanding the role of self-organization in variation across local contexts. *Soc Sci Med* 2013;93:194-202. doi: 10.1016/j.socscimed.2012.05.040 22819737

17. Greenhalgh T, Papoutsi C. Studying complexity in health services research: desperately seeking an overdue paradigm shift. *BMC Med* 2018;16 doi: 10.1186/s12916-018-1089-4 29921272

18. Pawson R. The Science of Evaluation: A realist manifesto: Sage 2013.

19. Creswell J. Qualitative Inquiry and Research Design: Choosing Among Five Approaches. Third Edition ed: Sage Publications 2013.

20. Marchal B, Dedzo M, Kegels G. A realist evaluation of the management of a well-performing regional hospital in Ghana. *BMC Health Services Research* 2010;10 doi: 10.1186/1472-6963-10-24

21. Manzano A. The craft of interviewing in realist evaluation. *Evaluation* 2016;22(3):342-60. doi: 10.1177/1356389016638615

22. Emmel N. Sampling and Choosing Cases in Qualitative Research: A Realist Approach London: Sage Publications Ltd 2014.

23. Malterud K, Siersma V, Guassora A. Sample size in qualitative interview studies: guided by information power. *Qualitative Health Research* 2016;26(13):1753-60. doi: 10.1177/1049732315617444

24. Greenhalgh T, Pawson R, Wong G, et al. Retroduction in realist evaluation. The RAMESES II Project, 2017.

25. Gilson L. Trust and the development of health care as a social institution. *Soc Sci Med* 2003;56:1453-68.

26. Gilmore B. Realists evaluations in low-and-middle income countries: reflections and recommendations from the experiences of a foreign researcher. *BMJ Global Health* 2019;4 doi: 10.1136/ bmjgh-2019-001638

1. www.nest360.org [↑](#footnote-ref-1)
