# Supplementary material for: Understanding the initiation, formation, functioning, and performing of networks to change practices – Realist evaluation of a programme to improve newborn care in Kenya
Source: SSM Health Syst. 2025 Dec;5:100101. doi: 10.1016/j.ssmhs.2025.100101 (PMC12678620; doi:10.1016/j.ssmhs.2025.100101)
Supplement: Supplementary file 2 — Supplementary material [file mmc2.docx]

# Appendix B. Interview Guide

## **Interview Guide**

Semi-structured realist interview guide

| Interviewee Code |  |
| --- | --- |
| Place and date |  |
| Period worked on NBU/hospital/organisation |  |

*Introduction:*

- *Introduce myself: My name is Katherine Kalaris. I am a 3^rd^ year DPhil student at the University of Oxford. I am in Kenya for a few months as part of my DPhil project. My research looks at understanding how clinical and programme networks of people, organisations, facilities, etc. form, function, and perform. I am looking at the NEST programme in Kenya as a case study of a programme network.*
- *Explain purpose of the interview and how it is structured: The purpose of this interview is to speak with you about how you see NEST working as a network. I will start with a few general questions about your experiences and interactions with NEST and then go into more detail on how you see things working as a network and any changes you have experienced.*
- *If it’s ok with you I would like to record this interview. Everything will be anonymous and we can stop at any time. Seek consent*

1. Please describe your professional/clinical role?
2. For hospital base staffed (clinical and non-clinical), who do you interact with in your role? For NEST/MoH/stakeholders who do you interact with in respect to the NEST programme?
3. When did you start engaging with NEST? / How long have NEST activities been going on in your facility? / How did you get involved with NEST activities?
4. Could you explain to me what NEST is? How does it work?

- In hospitals, how do you distinguish it from CIN activities?

1. Thinking of NEST, how do you feel part of a programme or network or initiative? (probe do you feel part of something specific from your standard activities)
2. What have your experiences been with NEST? Could you give some examples? What have these experiences enabled you to do?

For example: Meetings (What is it like going to a NEST meeting and connecting with others?), trainings, newborn clinical protocol updates, quality improvement activities/QI visits, mentoring and supportive supervision visits, virtual connections, etc.

Would you be able to tell me more about…….

(*focus on exploring specific elements of the programme theory based on interviewee or responses above*)

1. How has NEST changed in your facility overtime? / How have your interactions with NEST changed over the course of the programme? Could you give any examples?
2. From your perspective, could you describe any changes in practice on NBUs since the start of NEST? Or in the larger network? Could you give any examples?
3. What do you think has enabled (each of) those changes?

*Probe to see what the deeper causes may be and map to parts of the programme theory*

Would you be able to tell me more about….

*(focus on exploring specific elements of the programme theory based on interviewee or responses above)*

1. Are there things that don’t work well in terms of NEST being a network? Could you give me some examples? How does it compare to other (multi-organisation) programmes that you have worked with?
2. Is there anything else you would like to add?

*Close:*

- *Thank the interviewee for their time*
- *Inform them that they will be made aware of findings*

*Map any responses to parts of the programme theory and probe deeper*

- Identifying a problem
- Taking action to solve a problem
- Developing a collective vision
- Forming purposeful relationships, linkages, and partnerships (Through what activities have relationships, linkages, and partnerships evolved?)
- Network Leadership
- Developing a network identity and culture
- Commitment
- Engaged and motivated network members
- Creation of a psychological safe space
