# Supplementary material for: Understanding the initiation, formation, functioning, and performing of networks to change practices – Realist evaluation of a programme to improve newborn care in Kenya
Source: SSM Health Syst. 2025 Dec;5:100101. doi: 10.1016/j.ssmhs.2025.100101 (PMC12678620; doi:10.1016/j.ssmhs.2025.100101)
Supplement: Supplementary file 3 — Supplementary material [file mmc3.docx]

# Appendix C. List of analysed documents

**Newsletters**

NEST360 Newsletter Q1 (2021)

NEST360 Newsletter Q2 (2021)

NEST360 Newsletter Q3 (2021)

NEST360 Newsletter Q4 (January 2022)

NEST360 Newsletter Q1 (April 2022)

NEST360 Newsletter Q2 (July 2022)

NEST360 Newsletter Q3 & Q4 (December 2022)

**Annual reports**

NEST360 Annual Impact Report 2020

NEST360 Highlights 2021

NEST360 Highlights 2022

**Stories**

Opportunity in adversity (March 8, 2021)

A look into biomedical engineers and technicians training (May 2, 2022)

Kenya holds GIC for pre-service and clinical mentorship (August 2, 2022)

World Prematurity Day Celebrations (November 30, 2022)

Partners updates – an overview of the past few months (November 29, 2022)

Fellow spotlight: Natalie Nichell, Celsi Warmer Study Kenya (February 3, 2023)

**Personal communications**

NEST360 Annual Progress Report 2020

NEST360 Annual Progress Report 2021

NEST360 Annual Progress Report 2022

NEST360 Annual Progress Report 2023
