# Supplementary material for: Understanding the initiation, formation, functioning, and performing of networks to change practices – Realist evaluation of a programme to improve newborn care in Kenya
Source: SSM Health Syst. 2025 Dec;5:100101. doi: 10.1016/j.ssmhs.2025.100101 (PMC12678620; doi:10.1016/j.ssmhs.2025.100101)
Supplement: Supplementary file 4 — Supplementary material [file mmc4.docx]

## Appendix D. Substantive theories

| **Theory/Concept** | **Key Author** | **Overview** |
| --- | --- | --- |
| Theory of collective behaviour | Smesler  1962 | - Explanation for the formation of social movements - Social movements start with the experience of a strain, a generalisable belief rises identifying the source of the strain, a precipitating event occurs, leadership gives the movement direction, social control mechanisms can affect the direction of the movement |
| Collective identity approach from New Social Movement Theory | Melucci  1995 | - Shared identity is developed through common experiences and emotions and the development of a common perspective or understanding |
| Leadership in social movements | Ganz 2010 | - “accepting responsibility to enable others to achieve shared purpose in the face of uncertainty” - Five leadership practices: relationship building, storytelling, devising strategy, structuring social movements, and catalysing action |
| Theory of planned behaviour | Ajzen  1985 | - When the behaviour is not under self-control, an individual has greater perceived control over the behaviour if they think they have the resources and opportunities to perform the behaviour; they may have lower intentions to perform the behaviour if they lack resources and opportunities, even if they have a favourable attitude toward performing the behaviour |
| Self-efficacy theory | Bandura  1977 | - Individual’s belief in their ability to enact behaviours is needed to arrive at a certain outcome |
| Small group development theory | Tuckman  1965 | - Model that conceptualises changes in the behaviour of groups - The model has four stages: forming (coming together, developing relationships), storming (resistance to the newly formed group), norming (developing cohesiveness, new standards), and performing (the group is functional and executes tasks) |
| Organisational culture theory | Schein  1990;  Schein, Bennis, Blake  1965 | - “Set of shared mental assumptions that guide interpretation and action in organisations by defining appropriate behaviour for various situations” |
| Organisational commitment theory | Porter  1974;  Meyer, Allen  1991 | - “Attachment to the organisation, characterised by an intention to remain in it; an identification with the values and goals of the organisation; and a willingness to exert extra effort on its behalf” - Types of commitment: affective, continuance, normative commitment |
| Collective intelligence theory  *(Realist review only)* | Malone, Bernstein  2015;  Malone, Woolley  2020 | - “Groups of individuals acting collectively in ways that seem intelligent” - Explains extrinsic and intrinsic motivations |
| Psychological safe space | Edmondson  2004 | - “Individuals’ perceptions about the consequences of interpersonal risks in their work environment” - “A climate in which the focus can be on productive discussion that enables early prevention of problems and accomplishment of shared goals, because people are less likely to focus on self-protection” |
| Empowerment Theory | Zimmerman 2000 | - Individual: Beliefs about self-competence, efforts to enact control, understanding of the environment; developed from engagement in activities and organisations; self-efficacy - Organisational: provide members opportunities for skill building and to take on additional and meaningful roles; shared responsibilities; support from peers and a social identity; shared leadership and decision-making - Community: skills, desires and resources to improve the community, including identifying needs and strategies to address them and taking action to address them |
| Culture Typology | Quinn & Cameron 2011 | - Four types of culture in organisations - Clan: collaboration, high engagement and commitment from members, values relationships and teamwork, promotes shared values and goals, empowers members to facilitate commitment and loyalty to the organisation - Hierarchy: procedural, structured, controlling - Market: results and external orientated, competitive, goal-focused - Adhocracy: entrepreneurial, dynamic, risk-taking leaders, innovative |
| Relational Coordination Theory | Bolton, Logan, Gittell 2021 | - Stakeholders coordinate work through processes of communicating and relating across roles to achieve desired outcomes - Organisational structures may or may not support these processes - Relational and work process interventions |
| Relational Systems & Organisational Resilience | Bartel & Rockmann 2024 | - Better relationships in an organisation can better handle situations and enact resilient responses - Strong relationships support members’ attention in crises - Positive attention to relationships in an organisation; the value of interpersonal relationships to the organisation is recognised and resources are allocated to cultivate relationships - Communication styles shape relationships |
| Teamwork | LaFasto & Larson 2001 | - Components that increase team effectiveness - Members, relationships, problem-solving, leadership, and organisation environment |
| Action Theory | Bowey 1972 | - Different types of relationships between organisation members and the organisation’s goals - Commitment to the goals - Acceptance of the goals as part of the organisation with an obligation to contribute to goals, however they have other aims which they desire to achieve by being part of the organisation - Aims are unclear or flexible and members may engage in discussing their direction - Disagreement with the goals but must remain organisation members |

### References

Bandura A. Self-efficacy: Towards a unifying theory of behavioral change *Psychological Review* 1977;84(2):191-215

Bartel CA, Rockmann K. The disease of indifference: How relational systems provide the attentional infrastructure for organizational resilience. *Strategic Organization* 2024;22(1) doi: 10.1177/14761270231183441

Bolton R, Logan C, Gittell JH. Revisiting Relational Coordination: A systematic review. *The Journal of Applied Behavioral Science* 2021;57(3):290-322. doi: 10.1177/0021886321991597

Bowey AM. Approaches to organisation theory. *Soc Sci Inform* 1972;11(6):109-28

Cameron KS, Quinn RE. The Competing Values Framework. Diagnosing and Changing Organizational Culture San Francisco: Jossey-Bass 2011

Edmondson A. Psychological Safety, Trust, and Learning in Organizations: A Group-Level Lens. In: Kramer RM CK, ed. Trust and Distrust in Organizations: Dilemmas and Approaches. New York: Russell Sage Foundation 2004

Engles B, Muller M. Northern theories southern movements?: contentious politics in Africa through the lens of social movement theory *Journal of Contemporary African Studies* 2019;37(1):72-92. doi: 10.1080/02589001.2019.1607967

Fishbein M, Ajzen I. Belief, attitude, intention, and behavior: an introduction to theory and research. Reading, Mass; London: Addison-Wesley 1975

Fominaya CF. Collective Identity in Social Movements: Central concepts and debates. *Sociology Compass* 2010;4/6(393-404) doi: 10.1111/j.1751-9020.2010.00287.x

Ganz M. Leading Change: Leadership, Organization, and Social Movements. In: Nohria N, Khurana R, eds. Handbook of Leadership Theory and Practice: A Harvard Business School Centennial Colloquium. Boston, MA Harvard Business Press 2010:527-68

Ganz M, McKenna E. Bringing Leadership Back In In: Snow DA, Soule SA, Kriesi H, et al., eds. The Wiley Blackwell Companion to Social Movements Second ed: John Wiley & Sons Ltd 2019

LaFasto F, Larson C. When teams work best: Sage Publications 2001

Malone TW, Wolley AW. Collective Intelligence. In: Sternberg, R.J. (Ed), The Cambridge Handbook of Intelligence. 2ed. Cambridge, UK. Cambridge University Press. 2020

Meyer J, Allen N. A three-component conceptualization of organizational commitment *Human Resources Management Review* 1991;1(1):61-89

Polletta F, Jasper J. Collective Identity and Social Movements. *Annual Review of Sociology* 2001;27:283-305

Porter L, Steers R, Mowday R, et al. Organizational commitment, job satisfaction, and turnover among psychiatric technicians. *Journal of Applied Psychology* 1974;59(5):603-09

Schein EH. Organizational Culture. *American Psychologist* 1990;45(2):109-19

Tuckman B. Developmental sequence in small groups. *Psychological Bulletin* 1965;63(6):384-99

Weeber S, Rodeheaver D. Militias at the Millennium: A test of Smelser’s Theory of Collective Behavior. *The Sociological Quarterly* 2003;44(2):181-204. doi: 10.1111/j.1533-8525.2003.tb00554.x

Zimmerman MA. Empowerment Theory: Psychological, Organizational and Community Levels of Analysis. In: Rappaport J, Seidman E, eds. Handbook of Community Psychology. New York: Kluwer Academic/Plenum Publishers 2000
