# Supplementary material for: Understanding the initiation, formation, functioning, and performing of networks to change practices – Realist evaluation of a programme to improve newborn care in Kenya
Source: SSM Health Syst. 2025 Dec;5:100101. doi: 10.1016/j.ssmhs.2025.100101 (PMC12678620; doi:10.1016/j.ssmhs.2025.100101)
Supplement: Supplementary file 5 — Supplementary material [file mmc5.docx]

# Appendix E. Programme theory framework with CMOC references

Communication; Trust; Energy, effort, & passion

|  | **Initiation & Formation** | **Functioning & Performing** | **Sustaining Change & Impact** |
| --- | --- | --- | --- |
| **Network Processes** | | | |
| **Identify a problem** | - Initial strain or recognition of a problem (1A, 1B) - Identifying an opportunity achievable by their capabilities & (potential) resources (1C) - Recruitment (1D, 1F, 1G) | - Ongoing problem identifying, problem solving, and solution identification (1E, 1H-1J) | - Imbedding ongoing problem solving and solution identification in routine systems with ownership from government/hospitals (1H, 1J) |
| **Collective vision** | - Developing the vision (2A, 2B, 2C) - Introducing and communicating the vision (2D, 2H, 2I) - Committing to the vision (2D-2J) | - Sustaining and engagement with the vision (2K, 2M) - Formalise the vision with agreements (2L) - External awareness of the vision (2N, 2O) | - Engage stakeholders in the network’s vision (2O, 2P) - External spread of the vision (2N, 2P) |
| **Taking action to solve the problem** | - Recruitment to the cause (3A-3C) - Opportunities for network members to connect (3E, 3F) - Support from leadership and champions and resources (3D, 3G) - Knowledge sharing (3H.1, 3H.2) | - Ongoing problem solving (3H.3) - Leadership effort and engagement (3J.1-3K) - Cross-learning (3I) - Knowledge sharing (3H.1, 3H.2, 3J) | - Ongoing problem solving (3H.3) - Leadership effort and engagement (3J.1-3K) - Cross-learning (3I) - Knowledge sharing (3H.1, 3H.2) |
| **Network identity & culture** | - Establishing an identity and culture (6A-6D) | - Sustaining through cross- learning, knowledge sharing, teamwork, communication (6E, 6G, 6H) - Demonstration and reinforcement by leadership (6F) | - Sustaining and evolving through cross-learning, knowledge sharing, teamwork, communication (6E, 6G, 6H) |
| **Network Activities** | | | |
| **Knowledge & skills dissemination** | - Training and Mentorship (14A-14B.2) | - Training and mentorship (14A-14B.2) - Out of department/network (14C, 14D) | - Training and mentorship (14A- 14B.2) - Out of department/network (14C, 14D) |
| **Cross-learning** | - Inter-professional (13A) - Inter-facility (13B.1-13C) | - Inter-professional (13A) - Inter-facility (13B.1-13C - Continuing remotely (13D) - Beyond the network (13E) | - Inter-professional (13A) - Inter-facility (13B.1-13C) - Continuing remotely (13D) - Beyond the network (13E) |
| **Resourcing** | - Initial resources (2J, 3D, 6E, 8F, 4H) | - Human resources (16C) - Engage stakeholders (16D) - Engage sub-national level (16E) | - Transition and sustainability (16A, 16B) - Human resources (16C) - Engage stakeholders (16D) - Engage sub-national level (16E) |
| **Leadership & Champions** | - Recruit leadership (5A) - Enacting leadership roles (5B- 5H, 5J, 5Q) - Communication (5B, 5C) - Provide support (5D, 5E, 5H- 5K, 11C-11E) - Engage champions (11A-11D) | - Sustaining and cultivating leadership and champions (5L, 5O.3, 5R, 5S, 11F, 11G) - Engage stakeholders (5P, 5T) - Enacting leadership roles (5B- 5H, 5J, 5Q) - Provide support (5D, 5E, 5H- 5K, 11C-11E) | - Engage stakeholders (5P, 5T) - Sustaining and cultivating leadership and champions (5L, 5O.3, 5R, 5S, 11F, 11G) |
| **Adaptability** | - Adapt for viability (15A) | - Adapt to optimise functioning (15B) - Human resource changes (15C-15E) | - Adapt for changing members and leaders (15C-15E) |
| **Network Foundations** | | | |
| **Teamwork** | - Multi-disciplinary (12A, 12B) | - Multi-disciplinary (12A, 12B) - Provide support (12C, 12F) - Change practices (12D, 12E) | - Multi-disciplinary (12A, 12B) - Provide support (12C) - Change practices (12D, 12E) |
| **Psychological safe space** | - Trusting and respectful horizontal relationships (9A, 9B) | - Improved communication, collaboration, and working (9C, 9D, 9F) - Innovation (9E) | - Improved communication, collaboration, and working (9C, 9D, 9F) - Innovation (9E) |
| **Committed, engaged, motivated, empowered, & confident members** | - Identify with collective vision, identity, and culture (7A, 7B, 7E, 8B, 8C, 8E, 8G) - Support from leadership and network activities (7C, 8A, 10B, 10C) - Availability of resources (8F) | - Change practices (7D, 7F, 7H, 8C, 8D, 10E) - Engage stakeholders (7F, 7G, 7J, 7K, 8I) - Knowledge sharing (7I, 8H, 10B-10D) - Continued commitment to collective vision, identity, and culture (8G) - Champions emerge (10A) - Change in attitude (10F) | - Change practices (7D, 7F, 7H, 8C, 8D, 10E) - Engage stakeholders (7F, 7G, 7J, 7K, 8I) - Knowledge sharing (7I, 8H, 10B-10D) - Continued commitment to collective vision, identity, and culture (8G) |
| **Purposeful relationships, linkages, & partnerships** | - Identify with collective vision and identity (4A, 4D) - Invest in developing relationships (4B, 4C, 4G) - Understanding of expectations (4E, 4F) - Availability of resources (4H) - Cross-learning (4I) | - Cross-learning (4I) - Improved communication (4J) - Ownership (4K) - Trust (4L) - Psychological safe space (4M) - Provide support (4N) - Change attitudes (4P) | - Cross-learning (4I) - Ownership (4K) - Psychological safe space (4M) - Resilience of relationships (4L, 4M, 4O, 4P) |

Communication; Trust; Energy, effort, & passion
