# Supplementary material for: Understanding the initiation, formation, functioning, and performing of networks to change practices – Realist evaluation of a programme to improve newborn care in Kenya
Source: SSM Health Syst. 2025 Dec;5:100101. doi: 10.1016/j.ssmhs.2025.100101 (PMC12678620; doi:10.1016/j.ssmhs.2025.100101)
Supplement: Supplementary file 6 — Supplementary material [file mmc6.docx]

# Appendix F. Realist Review and Evaluation CMOC comparison table

This table shows how the Context – Mechanism – Outcome Configurations (CMOCs) from our Realist Review were confirm, refined, or if we did not find supporting data from the primary data in our Realist Evaluation. The parts of the programme theory are arranged in line with the revised programme theory from our Realist Evaluation. We have not included the CMOCs that belong to new sections of the programme theory developed through the Realist Evaluation. The red text shows how CMOCs have been refined.

### Identify a problem

|  | **Realist Review CMOC** |  | **Realist Evaluation CMOC** | **Result** |
| --- | --- | --- | --- | --- |
| 1A | When potential network members feel a sense of dissatisfaction (misaligned with expectations or values) with an issue (e.g. in clinical care, service delivery organisation, or health system management) (context), they will have the energy, excitement, and motivation to do something about it (outcome) because of their frustration (mechanism) | 1A |  | *No supporting data* |
|  |  | 1B | When potential network members feel a sense of dissatisfaction (misaligned with expectations or values) with an issue (e.g. in clinical care, service delivery organisation, or health system management) (context), they will get annoyed/angry/fed up (outcome) because of their frustration (mechanism) | *New* |
|  |  | 1C | In the formation of an externally initiated network, when future network members/leaders/initiators are helped to believe that improvement/change with an issue (e.g. newborn care, morbidity, and mortality) is achievable (context), they will have the energy, excitement, and motivation to do something about it (outcome) because they feel doing so is a worthwhile use of their time and energy (mechanism) | *New* |
| 1B | When a network is in the initial stages and potential network members reflect together on problems (context), they will recruit others to the cause (outcome) because there is a realisation that they can be part of the solution to some degree (mechanism) | 1D | When a network is in its formation stage and potential network members reflect together on problems (context), they will recruit others to the cause (outcome) because there is a realisation that they can be part of the solution ~~to some degree~~ (mechanism) | *Refined* |
| 1C | When potential network members share and discuss their collective experiences, emotions, understanding, or perspectives for a commonly felt problem (context), they are better able to understand what needs to be done and find solutions (outcome) because they have more knowledge to draw on (mechanism) | 1E |  | *Confirmed* |
|  |  | 1F | In a setting with change potential, when network initiators meet and select others who share and amplify their concerns about the identified problem (context), this helps form ties that support network formation (outcome) because the shared concerns about the identified problem are mutually reinforced (mechanism) | *New* |
|  |  | 1G | When the network initiators get input from potential network members on the magnitude and resources needed to begin solving the identified problem (context), this starts to generate commitment from potential network members (outcome) because the potential network members feel their perspective and experiences are respected and valued (mechanism) | *New* |
| 1D | When members in an established network feel it is safe to critically examine and reflect on existing practices (context), they are able to identify new problems and potential solutions (outcome) because they feel enabled to challenge the status quo or feel in a psychological safe space to be able to challenge the status quo (mechanism) | 1H | When members in an established network feel it is safe to critically examine and reflect on existing practices together (context), they are able to identify new problems and potential solutions (outcome) because they feel enabled to challenge the status quo (mechanism) and/or feel in a psychological safe space to be able to challenge the status quo as a team (mechanism) | *Refined* |
|  |  | 1I | In an externally initiated network, when there is support and partnership from network initiators and organisers (e.g. through quality improvement and mentorship activities) (context), this can help to continue to identify problems that are within the network member’s capacity to change and determine appropriate solutions (outcome) because the network members feel in a psychological safe space to be able to challenge the status quo as a team (mechanism) | *New* |
|  |  | 1J | When the network enables members to regularly identify problems they can work together to address (context), this supports network functioning (outcome) because they can appreciate the value of the network (mechanism) | *New* |

### Developing a collective vision

|  | **Realist Review CMOC** |  | **Realist Evaluation CMOC** | **Result** |
| --- | --- | --- | --- | --- |
| 2A | When potential network members engage in an open process of collective sense-making around a problem (context), they can identify what they share in common (outcome) because they learn about each other’s experiences, emotions, understandings, or perspectives (mechanism) | 2A | When potential network members or network initiators engage in an open process of collective sense-making around a problem (context), they can identify what they share in common (outcome) because they learn about each other’s experiences, emotions, understandings, or perspectives (mechanism) | *Refined* |
| 2B | If potential network members identify and articulate commonalities among each other (context), then this enables the development of a collective network vision (outcome) because they understand each other’s perspectives (mechanism) | 2B | If potential network members or network initiators identify and articulate commonalities among each other (context), then this enables the development of a collective network vision (outcome) because they understand each other’s perspectives (mechanism) | *Refined* |
| 2C | When network members have common professional or vocational identities or calling (context), this facilitates the development of a collective network vision (outcome) because they are more likely to have common professional ideals or identities (mechanism) | 2C | When potential network members and/or network initiators have common professional or vocational identities or calling (context), this facilitates the development of a collective network vision (outcome) because they are more likely to have common professional perspectives and values (mechanism) | *Refined* |
|  |  | 2D | When network initiators introduce the idea/intention of the network to potential members when the network is forming and recruiting network members (context), this will generate early commitment to the collective vision (outcome) because potential network members understand the network’s purpose (mechanism) | *New* |
| 2D | If the collective vision of a network is based on shared experiences, emotions, perspective, and understanding among the network members (a similar specific way in which reality is perceived) (context), then this will lead to commitment to the collective vision (outcome) because network members feel represented (mechanism) | 2E | If a network’s collective vision is based on shared experiences, emotions, perspective, and understanding among the potential network members and network initiators (a similar specific way in which reality is perceived) (context), then this will lead to commitment to the collective vision (outcome) because potential network members feel represented (mechanism) | *Refined* |
|  |  | 2F | If potential network members know each other prior to network formation (context), this can help generate commitment to the collective vision (outcome) because there is existing trust between the potential network members (mechanism) | *New* |
|  |  | 2G | When potential network members engage in an open process of collective sense-making around a problem (context), this generates a sense of ownership around the collective vision (outcome) because potential network members feel heard/represented (mechanism) | *New* |
|  |  | 2H | When network leaders/champions consult and engage hospital/facility leadership (context), this generates commitment to the collective vision from the administrative hierarchy even if they have no leadership role within the network (outcome) because they understand its value (mechanism) | *New* |
| 2E | If the collective vision of a network is communicated and explained by network leadership in a way that appeals to professional ideals and values shared by network members (context), then this will lead to commitment to the collective vision (outcome) because network members feel engaged (mechanism) | 2I | If the collective vision of a network is communicated and explained by network leadership in a way that appeals to professional ideals and values shared by potential network members (context), then this will lead to commitment to the collective vision (outcome) because potential network members feel engaged (mechanism) | *Refined* |
|  |  | 2J | When the network provides resources to support making changes to the physical infrastructure of facilities within the network (context), this encourages potential network members to commit to the collective vision (outcome) because potential network members feel that the network is doing something for them (mechanism) | *New* |
| 2F | When formal agreements have been negotiated and agreed between network members (context), then the network members will be more likely to follow a collective vision (outcome) because this helps network members to understand their roles and responsibilities (mechanism) | 2L | When formal agreements have been negotiated and agreed between network members (context), then the network members will feel solidified in a network and be more likely to follow a collective vision (outcome) because this helps network members to understand their roles and responsibilities (mechanism) | *Refine* |
| 2G | When potential network members establish and agree to a collective vision (context), this purposefully links them into a network (outcome) because they have common understanding and perspectives on what the goals of the network are (mechanism) | 2K | When potential network members agree to an existing collective vision (context), they will be more willing to take on activities to enact the collective vision (outcome) because they have an understanding of what is expected of them (mechanism) | *Refined* |
| 2H | If there is no collective vision or if network members do not follow the network’s collective vision (context), then they will be less successful in implementing network activities (outcome) because network members do not feel committed to the network (mechanism) | 2M |  | *No supporting data* |
|  |  | 2N | When the network makes an effort to disseminate its vision to solve the identified problem beyond the network (context), then non-network stakeholders (e.g. partners, donors) will be more aware of the network (outcome) because network members have advocated for its purpose (mechanism) | *New* |
|  |  | 2O | When the network engages with government officials and takes their inputs into consideration (context), greater support is generated for the network and network activities (outcome) because there is closer alignment of vision between the network and the government and government officials feel valued and respected (mechanism) | *New* |
|  |  | 2P | When network leadership/organisers are engaged in government meetings (e.g. technical working groups) (context), this can improve the enabling environment for the identified problem/collective vision (outcome) because more stakeholders are aware and engage with the problem/vision (mechanism) | *New* |

### Taking action to solve a problem

|  | **Realist Review CMOC** |  | **Realist Evaluation CMOC** | **Result** |
| --- | --- | --- | --- | --- |
| 3A | In a bottom-up network when potential network members realise they alone are unlikely to be able to solve a problem (context), they will recruit likeminded colleagues for their cause (outcome) because they believe collective action is needed (mechanism) | 3A | In a network formed from the bottom-up when ~~potential~~ network members realise they alone are unlikely to be able to solve a problem (context), they will recruit likeminded colleagues for their cause (outcome) because they believe collective action is needed (mechanism) | *Refined* |
| 3B | While in a top-down/more directed network when health system administrators or managers realise that they need diverse health system actors to be able to solve the problem/meet the target (context), they will recruit/mandate/encourage colleagues they perceive to be capable to their cause (outcome) because they believe that collective effort/action is needed (mechanism) | 3B | In an externally initiated network when ~~health systems administrators or managers or~~ network initiators and organisers realise that they need diverse health system actors to be able to solve the problem/meet the target (context), they will recruit/mandate/encourage colleagues they perceive to be capable of helping their cause (outcome) because they believe a collective approach is needed (mechanism) | *Refined* |
| 3C | When potential network stakeholders think collective action is needed to solve the identified problem (context), they will seek out other individuals with common experience or perspectives (outcome) because they believe such people may be willing to help them (mechanism) | 3C | When ~~potential~~ network members think collective action is needed to solve the identified problem (context), they will seek out other individuals with common experience or perspectives (outcome) because they believe such people may be willing to help them (mechanism) | *Refined* |
| 3D | When members in an established network have access to resources that will help them solve the problem they have identified (context), they may be more prepared/more likely to intend to take action (outcome) because they believe they have a more realistic chance of success (mechanism) | 3D |  | *Confirmed* |
|  |  | 3E | When an externally initiated network is forming and opportunities are provided to network members to meet, discuss, and find solutions to problems (context), this will support network members to take action and shape an identity and a sense of belonging (outcome) because they are able to coordinate their work and work together | *New* |
|  |  | 3F | When network members of different roles/cadres/units/organisations work together as a functioning team (context), this enables them to take collective action towards solving the problem (outcome) because they have a common understanding of what needs to be done (mechanism) | *New* |
|  |  | 3G | When network leadership or organisers are supportive of network members taking action (context), network members will be more likely to take practical and concrete action to solve problems (outcome) because they feel their efforts are valued and worthwhile (mechanism) | *New* |
|  |  | 3H.1 | When trained network members are provided with support (e.g. quality improvement and mentoring) that is relevant to the problem they are addressing (context), they are more able to take practical and concrete action to solve problems (outcome) because they feel empowered (mechanism) | *New* |
|  |  | 3H.2 | When information that makes challenges visible emerges from supportive activities (e.g. quality improvement and mentoring) (context), network members feel a greater need to address the problem (outcome) because they feel accountable to network leaders/organisers (e.g. mentors, quality improvement team) | *New* |
|  |  | 3H.3 | When network members take practical action as a team (context), this mobilises the network members efforts and resources (outcome) because they feel internal accountability towards other network members (mechanism) | *New* |
|  |  | 3I | When network members are provided with a platform for them to easily communicate (e.g. WhatsApp groups) (context), they are more able to take practical and concrete action to solve problems (outcome) because they can access and gain the knowledge and reassurance they need (mechanism) | *New* |
|  |  | 3J.1 | If network leaders/organisers continue to put effort and work into solving the identified problem (context), this supports network members to make progress towards changes in practice/improvements (outcome) because they feel encouraged to do so (mechanism) | *New* |
|  |  | 3J.2 | When efforts are made to provide consistent support to network members from network leadership/organisers (e.g. through quality improvement and mentorship) (context), it promotes network members to take action (outcome) because network members develop a bond with those providing support (feeling part of a larger team) and feel empowered by and accountability to them (mechanism) | *New* |
|  |  | 3K | If, despite the support provided, network members fail to act or make progress (context), this starts to undermine the relationships between network members and those providing support (outcome) because they feel the network members do not value the effort (mechanism) | *New* |

### Developing a network identity and culture

|  | **Realist Review CMOC** |  | **Realist Evaluation CMOC** | **Result** |
| --- | --- | --- | --- | --- |
| 6A | When network members with a collective identity come together to solve a shared problem (context), this makes them feel fulfilled (outcome) because of a sense of shared purpose (mechanism) | 6A |  | *Confirmed* |
| 6B | When network members feel fulfilled from working with likeminded people in a network (context), they want to belong to the network (outcome) because they feel it is worthwhile (mechanism) | 6B |  | *No supporting data* |
| 6C | When network members identify with other network members and the network’s vision (context), they develop a network identity (outcome) because it gives them a sense of purpose (mechanism) | 6C |  | *Confirmed* |
|  |  | 6D | When a network celebrates the contributions made by previously unrecognized/ unacknowledged hospital unit/facility leaders (context), this gives hospital unit/facility leaders an identity in the network as important network members (and in the hospital/facility) (outcome) because their expertise and role is now more valued by others (mechanism) | *New* |
|  |  | 6E | When the identity of a network is linked with network partners/initiators providing resources to the network (context), the network has a separate (not integrated into the health system) identity that may make it less sustainable (outcome) because the network partners/initiators have not put in sufficient effort to integrate the network in the health system (mechanism) | *New* |
|  |  | 6F | When network leadership and members consistently and regularly demonstrate and reinforce the network identity (context), this helps other network members adopt the network identity (outcome) because of role modelling (mechanism) | *New* |
| 6D | When a network creates opportunities for members to connect, share experiences, and learn from each other in an open and safe environment (context), this creates a positive network culture and a feeling of belonging to the network (outcome) because they feel respected and valued (mechanism) | 6G | When a network creates opportunities for members to connect, share experiences, and learn from each other in an open and safe environment (context), this creates a positive network culture and a feeling of belonging to the network (outcome) because they feel respected, valued, and acknowledged (mechanism) | *Refined* |
| 6E | When network leadership and members identify that the network’s culture is not aligned to existing cultures (context), then they may attempt to change the pre-existing cultures and instil a culture (outcome) because they do not identify with the existing ones (mechanism) | 6H | When local network leadership and members recognise that the network’s culture is not aligned to existing cultures in their environment (context), then they may attempt to change the pre-existing cultures to make room for the network culture and practices (outcome) because they no longer identify with the pre-existing culture and see value in the network culture (mechanism) | *Refined* |

### Network leadership

|  | **Realist Review CMOC** |  | **Realist Evaluation CMOC** | **Result** |
| --- | --- | --- | --- | --- |
|  |  | 5A | Networks, initiated and supported by outside organisations/partners (context), will recruit organisations or individuals into leadership positions that they think are competent for network formation and functioning (outcome) because they believe these organisations or individuals understand what the network needs to succeed (mechanism) | *New* |
| 5A | When networks have a member(s) that clearly takes on a leadership role and focuses on building linkages between potential network members and stakeholders and creates communication channels (context), this helps the network to form (outcome) because it brings people together (mechanism) | 5B |  | *No supporting data* |
| 5B | When networks have a member(s) that clearly takes on a leadership role and communicates the network’s vision and helps to set collective goals (context), this helps to bring network members around the collective vision and to move forward in the same direction (outcome) because they have the same understanding (mechanism) | 5C | When networks have a member(s) that enacts leadership capabilities, communicates the network’s vision, and helps to set collective goals (context), this helps to bring network members around the collective vision and to move forward in the same direction (outcome) because they have the same understanding of the network’s value (mechanism) | *Refined* |
| 5C | When networks have a member(s) that clearly takes on a leadership role and actively coordinates network members (context), this helps the network to form (outcome) because there is a central figure bringing people together (mechanism) | 5D |  | *No supporting data* |
| 5D | When networks have a member(s) that clearly takes on a leadership role and provides support and feedback to network members (context), this can help the network function and take more appropriate action towards the identified problem (outcome) because network members are engaged and see what they need to do to help achieve the vision (mechanism) | 5E | When networks have a member(s) that enacts leadership capabilities and provides support and feedback to network members (context), this can help the network function and take more appropriate action towards the identified problem (outcome) because network members feel empowered to take action to work towards achieving the vision (mechanism) | *Refined* |
| 5E | When networks have a member(s) that takes on a leadership role and gets things done (context), network members develop greater commitment, engagement, and/or motivation (outcome) because they believe belonging to the network is worthwhile (mechanism) | 5F | When networks have a member(s) that enacts leadership capabilities and gets things done (context), network members develop greater commitment, engagement, and/or motivation (outcome) because they believe belonging to the network is worthwhile (mechanism) | *Refined* |
| 5F | When networks have a member(s) that takes on a leadership role and dedicates time to setting up and the functioning of the network (context), network members develop greater commitment, engagement, and/or motivation (outcome) because they believe belonging to the network is worthwhile (mechanism) | 5G |  | *No supporting data* |
| 5G | When networks have a member(s) that clearly takes on a leadership role and actively engages network members (context), network members develop greater commitment, engagement, and/or motivation (outcome) because network members feel part of the network (mechanism) | 5H |  | *No supporting data* |
| 5H | When network leadership consistently and regularly supports and provides resources to network members (context), this helps network members act to achieve the network’s collective vision (outcome) because the network members feel empowered (mechanism) | 5I |  | *Confirmed* |
| 5I | When network leadership consistently and regularly promotes, encourages, or champions practices or influences network members to take up certain practices (context), this helps network members to adopt these practices (outcome) because it allows network members to fulfil a latent desire to practice better (mechanism) | 5J | When network leadership and members consistently and regularly promote, encourage, or champion practices or influence network members to take up certain practices (context), this helps other network members to adopt these practices and for them to be sustained in the network/unit (outcome) because of role modelling (mechanism) | *Refined* |
| 5J | When network leadership creates a welcoming and inclusive environment within the network (context), members feel more able to critically discuss issues and suggest possible solutions (outcome) because network members feel that they are in a psychologically safe environment (mechanism) | 5K | When network leadership creates a welcoming, supportive, and inclusive environment within the network (context), members feel more able to seek support to improve knowledge, skills, or manner of working (outcome) because network members feel that they are in a psychologically safe environment (mechanism) | *Refined* |
| 5K | When networks have processes in place to identify and enable those with the necessary skills, motivations, or attitudes to take on leadership roles (context), this may support network functioning and may lead to changes happening (outcome), because the network can draw on a wider pool of engaged resources (mechanism) | 5L | When networks have processes in place to identify and enable those with the necessary skills, motivations, or attitudes to take on leadership roles from a wider pool of engaged human resources (context), this may support network functioning and may lead to changes happening (outcome), because the network is able to create a distributed form of leadership across the network (mechanism) | *Refined* |
| 5L | If a network is highly dependent on a few people to implement network activities (context), then it is at risk of being unsustainable (outcome) because of burnout and/or loss in those tasked with implementation (mechanism) | 5M | If a network is highly dependent on a few people or leaders to implement network activities or leaders that support their implementation (context), then it is at risk of being unsustainable or functioning poorly (outcome) because the leadership structure is fragile (mechanism) | *Refined* |
|  |  | 5N | When there is misalignment of priorities between network leaders/partner organisations (context), then one leader/partner organisation may feel they need to leave the network (outcome) because they do not feel they are moving in the same direction (mechanism) | *New* |
|  |  | 5O.1 | When there is a leadership/partner organisation change within the network and a new leader/partner organisation comes in (context), this can jeopardise maintaining the network’s collective vision, existing relationships, and functioning (outcome) because the new leader/partner organisation is external and has not assimilated into the network (mechanism) | *New* |
|  |  | 5O.2 | When a leadership change within the network introduces new working practices in the network (context), this can disrupt network activity implementation and functioning (outcome) because network members need to learn about new practices (mechanism) | *New* |
|  |  | 5O.3 | When a leadership change within the network introduces new working practices in a network without first discussing and negotiating these with network members (context), network members can become frustrated (outcome) because their experience and expertise in the network is not valued (mechanism) | *New* |
|  |  | 5P | When non-network health system leaders, whose buy-in/commitment to the network is important for network functioning, change (context), this can disrupt network activity implementation and functioning (outcome) because it puts at risk relationships essential for network functioning (mechanism) | *New* |
| 5M | When network leadership is not able to get the network to develop and agree on a shared vision (context), then it is at risk of not performing well and becoming unsustainable (outcome) because members are unclear as to what the network is for (mechanism) | 5Q |  | *No supporting data* |
|  |  | 5R | If network members take appropriate action to mitigate any disruption leadership changes might bring (context), then the network is more likely to keep functioning (outcome) because the impact of any disruptions are minimised (mechanism) | *New* |
|  |  | 5S | When there is a change of leadership within the network and new network leadership puts in efforts to maintain network functioning (context), network members feel that it is still worthwhile to be part of the network (outcome) because they feel the leaders value the network (mechanism) | *New* |
|  |  | 5T | When hospital/facility leadership sees the effort that the network is making and the changes that have resulted (context), this encourages them to take action to work towards the collective vision (outcome) because they understand the value of the work (mechanism) | *New* |

### Psychological safe space

|  | **Realist Review CMOC** |  | **Realist Evaluation CMOC** | **Result** |
| --- | --- | --- | --- | --- |
| 9A | When network leadership is available and approachable, invites input and feedback, and models openness, fallibility, and non-judgmental behaviour (context), then this promotes a network’s psychological safety (outcome) because network members feel empowered and not threatened when they speak up or make a mistake (mechanism) | 9A | When network leadership/organisers are available and approachable, invites input and feedback, and models openness, fallibility, and non-judgmental behaviour (context), then this promotes a network’s psychological safety (outcome) because network members feel empowered and not threatened when they speak up or make a mistake (mechanism) | *Refined* |
| 9B | When network members form trusting and respectful horizontal relationships (leading to flattened hierarchy) (context), then this promotes a network’s psychological safety (outcome) because network members feel equal (mechanism) | 9B |  | *Confirmed* |
| 9C | When a network has a shared network identity and culture that promotes a psychologically safe space (context), then network members are more likely able to learn, improve, and seek feedback (outcome) because they feel empowered and feel a reduced fear of negative consequences (mechanism) | 9C |  | *Confirmed* |
| 9D | When a network is a psychological safe space for network members (context), it enables members to openly raise concerns or problems (outcome) because they know they will be supported and there will not be negative repercussions (mechanism) | 9D |  | *Confirmed* |
| 9E | When a network is a psychological safe space for network members (context), it encourages innovation and innovative behaviour (outcome) because they know they will be supported and there won’t be negative repercussions (mechanism) | 9E |  | *Confirmed* |
| 9F | When a network creates a psychological safe space for network members (context), it enables them to more easily collaborate across the network’s facilities, levels, and sectors of care (outcome) because they already have a common ground and understanding (mechanism) | 9F | When a network creates a psychological safe space for network members (context), it enables them to more easily communicate and collaborate across the network’s facilities, levels, and sectors of care (outcome) because they already have a common ground and understanding (mechanism) | *Refined* |

### Commitment

|  | **Realist Review CMOC** |  | **Realist Evaluation CMOC** | **Result** |
| --- | --- | --- | --- | --- |
| 7A | When networks members identify with the network’s collective vision, identity, and culture (context), they are more likely to be committed to the network (outcome) because they believe in and value the vision, identity, and culture (mechanism) | 7A |  | *Confirmed* |
|  |  | 7B | When network members’ professional identity/calling align with the network vision (context), they are more likely to be committed to the network and enact affective commitment (outcome) because they professionally value/find importance in the network vision (mechanism) | *New* |
| 7B | When networks members have support from leadership/administration/stakeholders (context), they are more likely to be committed to the network (outcome) because they feel valued (mechanism) | 7C | When networks members have support from network leadership/~~administration/stakeholders/~~ organisers (context), they are more likely to be committed to the network (outcome) because they feel valued (mechanism) | *Refined* |
|  |  | 7D | When networks enable members to achieve professional norms (that are part of their professional identity) (context), they will be committed to the network (outcome) because of affective commitment (i.e. the alignment between their professional and the network’s norms) (mechanism) | *New* |
| 8B | When a network member gets ‘emotional’ benefits (positive feelings) or feel a sense of purpose from being part of the network (context), they are likely to be highly committed (outcome) because it is fulfilling for them (mechanism) | 7E |  | *Confirmed, moved from ‘Engaged and motivated network members to ‘Commitment’* |
|  |  | 7F | When a network shows network hospital/facility leadership it can improve care practices (context), this can generate commitment from them (outcome) because they see its value (mechanism) | *New* |
|  |  | 7G | In an externally initiated network, when the network initiators promote the efforts and accomplishments of the network to donors and the interested global community (context), this increases network member commitment to the network (outcome) because network members feel their efforts are acknowledged and valued (mechanism) | *New* |
| 7C | If a network has committed members (context), then they are more likely to act on their identified problem and collective vision (outcome) because members are willing to put in the energy and effort (mechanism) | 7H | If a network has committed and proud members (context), then they are more likely to act on the identified problem and collective vision (outcome) because members are willing to put in the energy, effort, and passion (mechanism) | *Refined* |
|  |  | 7I | When committed network members take action to disseminate knowledge to network members and others outside the network (context), this extends network benefits to network members and others outside the network (outcome) because of a greater availability of knowledge and skills (mechanism) | *New* |
| 7D | When influential outside stakeholders are actively engaged in network processes (context), then they are more likely to be committed to the network (outcome) because they can be helped to understand the collective vision of the network (mechanism) | 7J |  | *Confirmed* |
| 7E | When influential outside stakeholders are committed to the network and actively engaged in participating (context), then it may be easier for the network to achieve its aims (outcome) because network members feel empowered (mechanism) | 7K | When influential outside stakeholders (often specific individuals within an organisation) are committed to the network and actively engaged in participating (context), then it may be easier for the network to achieve its aims (outcome) because network members feel empowered (mechanism) | *Refined* |

### Engaged and motivated network members

|  | **Realist Review CMOC** |  | **Realist Evaluation CMOC** | **Result** |
| --- | --- | --- | --- | --- |
| 8A | When network leadership provides opportunities for network members to be supported, recognised, and learn (context), this creates engaged and motivated network members (outcome) because they derive direct benefits (mechanism) | 8A | When network leadership provides opportunities for network members to be supported, recognised, and learn, through training, equipment provision and support, and facility renovations (context), this creates engaged and motivated network members (outcome) because they derive direct benefits and feel like they belong to the network (mechanism) | *Refined* |
| 8B | When a network member gets ‘emotional’ benefits (positive feelings) or feel a sense of purpose from being part of the network (context), they are likely to be highly committed (outcome) because it is fulfilling for them (mechanism) |  |  | *Confirmed as 7E* |
| 8C | When network members’ personal identity strongly aligns with a network’s identity and culture (context), then this will result in engaged and motivated network members (outcome) because they feel they belong (mechanism) | 8B |  | *Confirmed* |
| 8D | When network members actively participate in network change practices that align with the professional values they live by (context), this creates engaged and motivated network members (outcome) because it helps them to fulfil their moral obligation or vocational calling (mechanism) | 8C |  | *Confirmed* |
| 8E | When a network can show its members that it can affect some change (context), members are more likely to continue their engagement with it (outcome) because of they can see its value (mechanism) | 8D |  | *Confirmed* |
|  |  | 8E | If a network can leverage the affective or normative professional commitment of its members to work towards achieving network goals (context), then they can become more engaged with the network (outcome) because of a sense of shared purpose (mechanism) | *New* |
| 8F | When engaged and motivated network members are provided with the resources and opportunity to act (context), then they will attempt to enact changes in practices (outcome) because they feel empowered to change practice/or work towards solving the problem (mechanism) | 8F |  | *Confirmed* |
|  |  | 8G | When network members are committed to the collective vision, that is strongly associated with their professional identity, are faced with issues that are beyond their control (context), they will not be deterred from continuing to engage with the network (outcome) because they believe what they are doing is worthwhile and feel accountable to other network members (mechanism) | *New* |
|  |  | 8H | When network members are transferred to facilities out of the network or out of the network unit (context), this leaves a gap in trained, engaged, and motivated network members (outcome) because their skills and importance are lost (mechanism) | *New* |
|  |  | 8I | When networks engage with non-network stakeholders in network activities (e.g. quality improvement) and deliberately seek out feedback and advice (context), this can improve network activity implementation (outcome) because they provide a broader understanding of relevant issues (mechanism) | *New* |

### Purposeful relationships, linkages, and partnerships

|  | **Realist Review CMOC** |  | **Realist Evaluation CMOC** | **Result** |
| --- | --- | --- | --- | --- |
| 4A | When network members believe in the network’s collective vision (context), then this can help ensure that network members have purposeful and co-operative working relationships (outcome) because they are more willing to work with and value working with people whom they identify as being likeminded (mechanism) | 4A |  | *No supporting data* |
| 4B | When network members are open to and able to invest time in developing relationships and linkages through the network (context), then this can help ensure that network members have purposeful and co-operative working relationships (outcome) because they have a better understanding of each other (mechanism) | 4B |  | *Confirmed* |
| 4C | If network members have strong pre-existing relationships (context), then this helps ensure that network members have purposeful and co-operative working relationships (outcome) because they are already familiar with each other (mechanism) | 4C |  | *Confirmed* |
|  |  | 4D | When network members share a professional identity (context), this supports forming purposeful and co-operative working relationships (outcome) because they share common socialised perspectives (mechanism) | *New* |
| 4D | If there are artefacts in place that outline the roles and responsibilities of network members (context), then this can help ensure that network members have purposeful and co-operative working relationships (outcome) because there is a common understanding among network members about what they are expected to do (mechanism) | 4E |  | *Confirmed* |
|  |  | 4F | When a common baseline understanding is created among network members of what is expected of them (context), this helps to develop purposeful relationships (outcome) because the network members have the same level of understanding about each other and their roles and responsibilities (mechanism) | *New* |
| 4E | When a network creates opportunities for relationship building between network members (context), this may help in the creation of purposeful and co-operative working relationships and improve existing working relationships (outcome) because they get to know each other’s personality, skills, ways of working, and motivations better (mechanism) | 4G | When a network creates opportunities for relationship building between network members within and across facilities (context), this may help in the creation of purposeful and co-operative working relationships and improve existing working relationships (outcome) because they get to know each other’s personality, skills, ways of working, and motivations better (mechanism) | *Refined* |
|  |  | 4H | When the network provides resources that supports the network members’ ways of working (context), this may help in the creation of purposeful and co-operative working relationships and improve existing working relationships across professions (outcome) because they are better able to collaborate and do their job (mechanism) | *New* |
|  |  | 4I | When network leadership creates a psychological safe space that enables cross-learning between network members (context), this may help in the creation of purposeful and co-operative working relationships and improve existing working relationships (outcome) because network members are able to openly share their experiences (mechanism) | *New* |
| 4F | When network members have established purposeful relationships (context), this helps to improve communication between network members (outcome) because they are familiar with each other (mechanism) | 4J | When network members have established purposeful relationships within and across network facilities (context), this helps to improve communication between network members (outcome) because they are familiar with each other (mechanism) | *Refined* |
|  |  | 4K | When purposeful linkages and strong working relationships are established between network members (context), this improves network members ownership of the network (outcome) because they feel connected to each other and to the vision (mechanism) | *New* |
|  |  | 4L | When purposeful linkages and strong working relationships are established between network members (context), this helps to build trust among network members (outcome) because network members have confidence in each other (mechanism) | *New* |
|  |  | 4M | When purposeful linkages and strong working relationships are established between network members (context), this supports the creation of a psychological safe space (outcome) because network members feel they can speak freely without fear of negative consequences (mechanism) | *New* |
|  |  | 4N | When purposeful linkages and strong working relationships are established between network members/organisers (context), this enables network members/organisers to provide support in enacting the collective vision (outcome) because they can develop a mutual understanding of the identified problem and potential solutions (mechanism) | *New* |
| 4G | When purposeful linkages and strong working relationships are established between network members or between network members and external stakeholders (context), this bridges gaps between network members or network members and stakeholders (outcome) because of mutual understanding (mechanism) | 4O | When purposeful linkages and strong working relationships are established between network members ~~or between network members and external stakeholders~~ (context), this helps them to be in agreement (outcome) because they can more easily develop a shared conceptualisation of the identified problem and potential solutions (mechanism) | *Refined* |
|  |  | 4P | When different professions within the network (e.g. clinical and technical) develop strong working relationships (context), their attitudes toward the other profession changes (outcome) because they understand each other’s value (mechanism) | *New* |
