# Supplementary material for: Understanding the initiation, formation, functioning, and performing of networks to change practices – Realist evaluation of a programme to improve newborn care in Kenya
Source: SSM Health Syst. 2025 Dec;5:100101. doi: 10.1016/j.ssmhs.2025.100101 (PMC12678620; doi:10.1016/j.ssmhs.2025.100101)
Supplement: Supplementary file 7 — Supplementary material [file mmc7.docx]

# Appendix G. Realist Evaluation CMOCs with illustrative supporting data

### Identify a problem

| **1A** | When potential network members feel a sense of dissatisfaction (misaligned with expectations or values) with an issue (e.g. in clinical care, service delivery organisation, or health system management) (context), they will have the energy, excitement, and motivation to do something about it (outcome) because of their frustration (mechanism) |
| --- | --- |
|  | *No supporting data* |
| **1B** | When potential network members feel a sense of dissatisfaction (misaligned with expectations or values) with an issue (e.g. in clinical care, service delivery organisation, or health system management) (context), they will get annoyed/angry/fed up (outcome) because of their frustration (mechanism) |
|  | *No supporting data* |
| **1C** | In the formation of an externally initiated network, when future network members/leaders/initiators are helped to believe that improvement/change with an issue (e.g. newborn care, morbidity, and mortality) is achievable (context), they will have the energy, excitement, and motivation to do something about it (outcome) because they feel doing so is a worthwhile use of their time and energy (mechanism) |
|  | *“Well, it started long before NEST in about 2006. I think, Rebecca and Maria, who are the biomeds, who've been the initiators of all this came to Malawi, primarily to visit the new Baylor HIV unit that had been was just being opened in Lilongwe. And I was at that opening. And they, I just happened to have tea with them, you know, and they, they said, Is there anything their students could do? And I was, I've worked with government all my life, I feel that's extremely important. And so, I was a bit irritated with all the money that was pouring into Baylor. So, I said, if you want to see proper medicine, you should come down to Blantyre and see. So, to my surprise, they came and asked what their students could do. And they were obviously taken with the neonatal unit. And so, I suggested CPAP, which we didn't have a robust sort of CPAP. And also a that at that stage phototherapy, I think, and I think they were quite interested, because I'd already had some sort of DIY type of hot cots made. So, they went away, and I didn't think they'd come back, because lots of people are fired up when they come, but not so fired up when they return. And, yeah, they did, they came with the phototherapy that worked very well and cost something like $25 instead of several hundreds. And so from that, developed the CPAP, which was made, and then we decided to put that throughout the country. And it was clear when you actually went to all these hospitals that that was that, that they actually didn't have basic neonatal training. And so even if they could make the baby breathe, you know, if you don't keep the baby warm and fed, and everything else, you're in trouble. So it became clear that more was needed. And from that develop this concept of giving a more holistic approach. And then because they're biomedics, they were, they could see the importance indeed, we all can of keeping your equipment working, and also training people in country to innovate, and make things that are created for themselves.” (FI 01)*  *“So now, when we came to the next year's conference, the issue of these equipments came in the NEST, the NEST bundles and all that things. So, when I was talking to with Aluvaala, he was like Nakuru is a very busy facility, it is well organized, we know the leadership is positive. So, do you think we can do this and I was like, I really need that. So, that is how we started engaging now, how they could get the facility and how then they could be included into the NEST project programme.” (FI 05)* |
| **1D** | When a network is in its formation stage and potential network members reflect together on problems (context), they will recruit others to the cause (outcome) because there is a realisation that they can be part of the solution (mechanism) |
|  | *No supporting data* |
| **1E** | When potential network members share and discuss their collective experiences, emotions, understanding, or perspectives for a commonly felt problem (context), they are better able to understand what needs to be done and find solutions (outcome) because they have more knowledge to draw on (mechanism) |
|  | *“They came with the phototherapy that worked very well and cost something like $25 Instead of several hundreds. And so from that, develop the CPAP, which was made, and then we decided to put that throughout the country. And it was clear when you actually went to all these hospitals that that was that, that they actually didn’t have basic neonatal training. And so even if they could make the baby breathe, you know, if you don’t keep the baby warm and fed, and everything else, you’re in trouble. So, it became clear that more was needed. And from that develop this concept of giving a more holistic approach. And then because they’re biomedics, they were, they could see the importance indeed, we all can of keeping your equipment working, and also training people in country to innovate, and make things that are created for themselves.” (FI 01)*  *“So for Kenya, when we began oh, my God, it was it was quite intense, because we did not have level two. When they say Level 2, I mean special newborn care guidelines. We did not have. So, we didn't have anything to base our programme on. But we knew that we need to because NEST gives equipment. We didn't just want to do user training. Oh, this is a CPAP machine using like this, because I think that had been done before, it wasn't working. So myself and Prof. Grace. We thought it would be important to get the devices the best practices, marry the two and help the country to create guidelines on how to use how to really take care of a baby in a level two newborn unit. So, if they have hypoglycaemia, what is the standard? How do you identify a baby with hypoglycaemia? What do you exactly do? Which devices do you use? How do you monitor? How do you know you're doing well?” (FI 29)* |
| **1F** | In a setting with change potential, when network initiators meet and select others who share and amplify their concerns about the identified problem (context), this helps form ties that support network formation (outcome) because the shared concerns about the identified problem are mutually reinforced (mechanism) |
|  | *“The model that NEST has used across all these facilities which is very, very peculiar, very, very, it is a unique way of, of getting into these facilities. Because they didn’t just get in. Others, other organisations will just get in, identify, you know a health facility and get in there. NEST has, you know, this health facility, I was talking about, health facility checklist that’s I was talking about, health assessment checklist that’s I was talking about. It is a very helpful tool because it gives you the rationale of why you are choosing a particular health facility. Unlike other organizations where they will choose any facility for the sake of choosing and you know you take an equipment there is no unit operating, there is no neonatal person. There is no nurse, there is no biomed. Then who is going to use that equipment? Those are the cases where by you realise two years down the line, and the equipment’s are still in a box, packed nicely and kept in a store somewhere. Because it was with a very good intention but the person who was giving out these equipments, or donating the way NEST do, did not consider whether this, the readiness, the facility readiness to uptake this project.” (FI 17)*  *“I think it was back in 2019. We’re told about the NEST idea and we’re told that they want to come to see how they can make a difference for a newborn unit. And first, they wanted to see which equipment, if they were improved and added to the unit, where they would make a difference. Some other time some two gentlemen came. And basically, they wanted to get information from us from which kind of equipment we would like to have, which improvement we like to have. And I think we had a discussion for like two hours, just looking at if there’s a suction, which one would you prefer, why that one, and if a radiant warmer, where would you want and what you want, where we would want the probe, how would you understand that to be like this kind of thing. And that it will say that there will be a study called NEST which will come give us equipment, then after some time we were told, the study is becoming reality. So, they came and I were informed about this study, it was just all of us in the newborn unit here and hospital, generally, the hospital management, about the study. And it was supposed to come first do a baseline study to see what we have how our newborn unit is how busy it is, and then see what equipment they can give us to make a difference for our babies because we’re trying to lower the the neonatal mortality rate so that it can also help lower the infant mortality rate.” (FI 23)* |
| **1G** | When the network initiators get input from potential network members on the magnitude and resources needed to begin solving the identified problem (context), this starts to generate commitment from potential network members (outcome) because the potential network members feel their perspective and experiences are respected and valued (mechanism) |
|  | *“When they started, it’s not that they started and went away. Okay, when they introduced this programme to this facility. Yeah. Many times they come and monitor the progress. They come do assessments, and we are working together. It’s not like we are left to work on our own. After the assessment, we are assisting together, we plan together the interventions, what is it that we need to do first of all to identify the gaps? What is it we have implemented this? What are the gaps? And what are the possible solutions? And the good thing that they actually do is that they look at the things that we are able to manage the solutions that we are able to do not that not the external solutions. Like now we’d say fans, we don’t have fans. So what but they’re looking at what is it within our our reach that we can be able to use to be able to bridge that gap? Yeah, and you see now we are working together with them. They are pulling us and we’re moving together. So, we feel really involved and starting from the management to the last person who is providing the service.” (FI 09)*  *“I think when they came in there was an assessment of the facility, what we had, and also our outcomes, in terms of the neonatal outcomes. And there was a suggestion of providing an intervention, which was, which seems to be attractive to us. We agreed to sign up for the programme, because after the baseline survey was done, we’re given a feedback, we’re also informed of some form of interventions that will help us to improve the neonatal outcomes, and neonatal survival. So that is how I came to know about the programme. I think it was there was an elaborate entry in terms of what the programme wanted to achieve, what we where we were, and what are our targets in terms of improving neonatal outcomes and reducing neonatal mortality.” (FI 12)* |
| **1H** | When members in an established network feel it is safe to critically examine and reflect on existing practices together (context), they are able to identify new problems and potential solutions (outcome) because they feel enabled to challenge the status quo (mechanism) and/or feel in a psychological safe space to be able to challenge the status quo as a team (mechanism) |
|  | *“The QI visits have also been happening again to just to improve the quality. I think we'll be having them every quarter. So, and during the QI visits, basically again, informed by our performance in as far as those documentation is concerned through the neonatal CIN, we're able now to identify just look at the data and then we are able to see where we are not doing well, where we're doing well and where we are doing well and we are able to maintain where the gaps. We come together even as we discussed with the QI team and even ourselves come up with ways of improving. And example is most of our QI visits and even documentation has shown that we have challenges with hypothermia so still working on that grouping in the maternity labour ward identify what are the issues we are putting forth to ensure heaters are provided in critical areas. So, we are hoping to see a drop in something like hypothermia which has been a major issue in this facility.” (FI 25)*  *“And I think also the the growing recognition and appreciation of some of the problems that still continue to contribute to neonatal mortality, for instance, hypothermia management, you know, that's something that every meeting, every place we went, people recognise that hypothermia, both in inborns and outborns, is still a major, major challenge. And through those meetings, some of them put corrective measures. So, for instance, some hospitals started using plastic wraps that they were not using before but after, you know, discussions with our teams, they took it back but taken it as an intervention.” (FI 18)* |
| **1I** | In an externally initiated network, when there is support and partnership from network initiators and organisers (e.g. through quality improvement and mentorship activities) (context), this can help to continue to identify problems that are within the network member’s capacity to change and determine appropriate solutions (outcome) because the network members feel in a psychological safe space to be able to challenge the status quo as a team (mechanism) |
|  | *“The QI visits are very important, they're there even to make us put on more effort, whenever the lapses are noted on our sides, through QI. The QI visits have also impacted the… empowered us even to be more productive in our work, because without our Q..., okay, on that particularly, you see it gives us a to do on toes, knowing very well there's no room for lapses. For us to improve on the quality and the quality because in the event maybe we did notice some some lapses they come in to tell us on how to go about it to get a problem solved. For example, if you have a challenge, maybe during those visits they normally even engage us to tell some of the challenges we normally face, then how to after out of that QI assessment, they are able to pick some of those problems, then they then they are advise on how to tackle them they give some advice based on the priority what is supposed what is supposed to come first. For example, recently we had one of the problems we had in the in the unit they want to do with a baby incubator and infant that we have failed to get a probe. Yes. So to do that QI visit you are able to advise as to go for to get in the room for maybe to go for a replacement to buy just another another equipment and they give us some time like like by the time they give us some timeline when we are supposed to perfect so it gives us keeps us a reminder to us and even even gives us that motivation to work hard towards maybe beating that timeline. Because they suggested that we do a replacement of equipment because so far efforts to get a replacement for the temperature probe has been futile.” (FI 13)*  *“However, there are six monthly mentorship I'm talking about is now where you have these mentors have an opportunity to go to another hospital, still, unless the implementing hospital where now they meet up with the mentors of that site. And before they go, they usually will have will have identified the mentorship needs. Okay, so there's some times we have a mentorship need that is across for all sites, like for now I know we are struggling with hypothermia. So, during this last mentorship, of course, we said, as much as we are doing so many other things, and hypothermia should really be a focus. So, every hospital will have identified their mentorship needs, maybe based on their day-to-day challenges. Sometimes we identify those needs from our data. So, looking at the data, you can see what people are struggling with. And then others, you just actually just see the need when you go there, the hospital might not have told you but as a visiting mentor, you can actually identify a problem and you'd want to just have the team deal with it or just support them in … that.” (FI 14)* |
| **1J** | When the network enables members to regularly identify problems they can work together to address (context), this supports network functioning (outcome) because they can appreciate the value of the network (mechanism) |
|  | *“When I need a spare I could talk to the biomed from the NEST side. I could first of all identify the problem. Then when I if it comes to a need for a spare I could either call in direct and also WhatsApp in what I what I need. Then within a very short time the spare brought here sometimes you find the to repair the equipment might be a bit tricky. We could they could also tell us to forward the equipment back to them so that they may also repair from their side then the equipment is brought back again.” (FI 21)* |

### Collective vision

| **2A** | When potential network members or network initiators engage in an open process of collective sense-making around a problem (context), they can identify what they share in common (outcome) because they learn about each other’s experiences, emotions, understandings, or perspectives (mechanism) |
| --- | --- |
|  | *No supporting data* |
| **2B** | If potential network members or network initiators identify and articulate commonalities among each other (context), then this enables the development of a collective network vision (outcome) because they understand each other’s perspectives (mechanism) |
|  | *No supporting data* |
| **2C** | When potential network members and/or network initiators have common professional or vocational identities or calling (context), this facilitates the development of a collective network vision (outcome) because they are more likely to have common professional perspectives and values (mechanism) |
|  | *“These people trusted us because maybe they knew us before but they also trusted that the content we're giving them what's recent. And then we have experts in Kenya. Like all the neonatologists were on board, and they're all speaking the same language. And they're all wanting to input and say, ‘Oh, no, in Canada, this happened, or in a SA this happened, maybe we should try this,’ you know. So, Everybody owned the whole thing. I feel that is what was probably the link. That helped, everybody was always constantly informed. What was happening.” (FI 29)* |
| **2D** | When network initiators introduce the idea/intention of the network to potential members when the network is forming and recruiting network members (context), this will generate early commitment to the collective vision (outcome) because potential network members understand the network’s purpose (mechanism) |
|  | *“On the biomedical side, I think we were introduced to NEST at a time when there was needed. And the I came to learn that the main, the main reason why NEST was established, they wanted to reduce the mortality rate, neonate newborn mortality rate by the 20 was 20 2050 or 30. They want to reduce it by a half. Yeah. Yes. So we were, we were introduced to NEST, they told us the intention of the program to do to do away with the neonatal death. Because they, particularly in Africa, it has been on the rise. So, the target was to have it reduced by half when it comes to by the by 20 2050. I'm not sure of the 2030? 2030, maybe, Do you think so? 2030 Yeah, yes. Yes. So, they introduced us, they told us about the intention, then apart from that, they went as far as far as training us, on the packages they're going to give to assist the newborn. So, for us to realise the goal, the vision they have for in the first-year arrangement, we were trained on how to handle the equipment, on how to handle to know more about the the working phase for the equipment.” (FI 13)*  *“I know the hospital, the hospitals that were selected to join the NEST implementation are hospitals that were in the CIN network. So of course, Kakamega was a CIN site, then of course, there are a lot of factors, I think they looked into the workload, in terms of deliveries, do you have a newborn space and all that. So, for NEST to come in, I know, of course, the there was an introduction to the hospital. And then now there was an introductory meeting, where the NEST leadership at that time met up with the hospital management of the hospital. So of course, they introduced what the programme was intending to achieve, and how they were planning to do it. And everything they had planned in terms of implementation, then after that, an MoU was signed, between NEST and the hospital. And so, I think that is where the journey started.” (FI 14)* |
| **2E** | If a network’s collective vision is based on shared experiences, emotions, perspective, and understanding among the potential network members and network initiators (a similar specific way in which reality is perceived) (context), then this will lead to commitment to the collective vision (outcome) because potential network members feel represented (mechanism) |
|  | *“Then the staff on the ground also being willing to take up those those changes as they come. So, you have to believe in what is coming also.” (FI 03)*  *“More like I'm part of this movement. Like, we can do better. I think people feel that way because you will find everybody was kind of inspired because we would always think that mechanical ventilation is a solution. But really it's the small things it's how you assess your baby when you go into the ward. It's how you talk to the mother. It's how you do infection prevention. It's how you, you know, small things. It's how you communicate to the person you're handing over to. So, I think that when we change people's mindset, they were able to feel like part of this movement.” (FI 29)* |
| **2F** | If potential network members know each other prior to network formation (context), this can help generate commitment to the collective vision (outcome) because there is existing trust between the potential network members (mechanism) |
|  | *“Some of these people we knew them. Because either Grace taught them or I taught them. So, they already like trusted us……These people trusted us because maybe they knew us before but they also trusted that the content we're giving them what's recent. And then we have experts in Kenya. Like all the neonatologists were on board, and they're all speaking the same language. And they're all wanting to input and say, ‘Oh, no, in Canada, this happened, or in a SA this happened, maybe we should try this,’ you know. So, everybody owned the whole thing. I feel that is what was probably the link that helped, everybody was always constantly informed what was happening.” (FI 29)*  *“Number two is that there is already an informal relationship that has formed within the CIN hospitals, professionals. So, when they meet, when they show up in a meeting, almost some of these 90% of the room or 80% of the room knows each other in some way or form.” (FI 32)* |
| **2G** | When potential network members engage in an open process of collective sense-making around a problem (context), this generates a sense of ownership around the collective vision (outcome) because potential network members feel heard/represented (mechanism) |
|  | *“Some of these people we knew them because either Grace taught them or I taught them, so they already like trusted us. What we're doing is useful and but we needed, we were very open. And we spoke of what we saw, what needs to be done. And we listened a lot. We really listened, we we created opportunities for people to, to feel like. Me and Grace didn't want to be the ones to create these things. We wanted them to feel like this is ours. Right? And I think that is still there even now. Because you would hear people to say during the validation meeting, ‘no, you can see when you remember, we tested this, this cannot work we have to do.’ So, we involved them from the very beginning. Those meetings we used to have the monthly meetings. I think those were very good, because we people would ask questions and clarify. And we would agree. This is the best way to to go forward with this. And we were involved everybody. And everybody owned the idea.” (FI 29)*  *“These people trusted us because maybe they knew us before but they also trusted that the content we're giving them what's recent. And then we have experts in Kenya. Like all the neonatologists were on board, and they're all speaking the same language. And they're all wanting to input and say, ‘Oh, no, in Canada, this happened, or in a SA this happened, maybe we should try this,’ you know. So, everybody owned the whole thing. I feel that is what was probably the link that helped, everybody was always constantly informed what was happening.” (FI 29)* |
| **2H** | When network leaders/champions consult and engage hospital/facility leadership (context), this generates commitment to the collective vision from the administrative hierarchy even if they have no leadership role within the network (outcome) because they understand its value (mechanism) |
|  | *“In fact, at that time, I was not the medical superintendent. I was just the in-charge of the department. And the hospital management team, upon sharing with them the objectives of NEST and how useful it was going to impact the outcome of the neonates it was accepted. Yes, they bought into it. We've got the initial support. In fact, the initial meetings involving the medical superintendent and and others that were held in Nairobi, from Kakamega came with that team. And they were very happy. We have received support throughout. Yeah, from the time NEST came on board.” (FI 04)*  *“Was receptive because, you know, critical care for newborns is one of the neglected areas in our setting. And it's an area that has very high mortality if you go into these hospital. So, an intervention that promises to cut that back, I think is quite attractive to hospital managers and Ministry of Health officials, so it was quite well received. In fact, you see, government purchasing quite a number of the NEST equipment even beyond what was donated. So that's normally my test of acceptability. Can they put their own money into it? And we've seen that coming up.” (FI 31)* |
| **2I** | If the collective vision of a network is communicated and explained by network leadership in a way that appeals to professional ideals and values shared by potential network members (context), then this will lead to commitment to the collective vision (outcome) because potential network members feel engaged (mechanism) |
|  | *“But I feel that open communication, trust, constant, updating, letting people give you input, I think that really helped. For us, it really helped us. And I thank God in Kenya, we have good internet, that was also very helpful, that we can we can be able to reach people with a click of a button, right? Yeah, that was very, very useful. And also, I think we had a very good foundation on what we wanted to achieve. We were very clear on what we want to do. And we're both very passionate. So, any opportunity, we got to talk about it, we talked about it and everybody was everybody was onboard.” (FI 29)*  *“I think there was, you know, the quantification of the problem, the magnitude of the problem, for people to really understand what they're dealing with. And, you know, visualizing that, you know, just how many babies are dying and promising with a promise that, you know, at a very, you know, with a very low cost, cost-effective solution, you can reverse that was. Those that conversation helped a lot, because we all working together towards a common problem where we think we have an opportunity to do something.” (FI 31)* |
| **2J** | When the network provides resources to support making changes to the physical infrastructure of facilities within the network (context), this encourages potential network members to commit to the collective vision (outcome) because potential network members feel that the network is doing something for them (mechanism) |
|  | *“A lot of changes, a lot of changes. I didn't even say initially that they actually renovated the unit. Yes, soon it was renovated. It initially it used to have few power points, but then after the renovations, there* *were power points everywhere. We used to really suffer because we didn't know when you had several gadgets to be put on to power. There was a challenge. But when NEST came in and it renovated this unit, it put power points in every room, several of them. So that's the those renovations were good. They actually put in when washing hand, washing points like this one you see outside there was not there, it was by NEST. And see when mothers come in even before they do anything else. They wash your hands. Yes. Yeah. So those renovations also really helped.” (FI 22)*  *“See, we were able to able to renovate the newborn unit and repainting. We're proud of that. Okay, it is been NEST who did for us…When you're working in a clean environment in a beautiful place, it motivates you to work... in a clean environment, beautiful place it motivates you to work.” (FI 26)* |
| **2K** | When potential network members agree to an existing collective vision (context), they will be more willing to take on activities to enact the collective vision (outcome) because they have an understanding of what is expected of them (mechanism) |
|  | *“We really felt great because the next training we figured, oh look at us we could do three sites. Because by when we had enough trainers, we had enough paediatricians, I think we had almost 100 people who we had taken through GIC both paediatricians, nurse, neonatal nurses, specifically neonatal nurses who work in the newborn unit, who have experience with these devices. And the neonatologists we trained. So, we had a pool of almost 100 of those. So, we thought let's do three sites at the same time. So, we did that. So, we had a team of five in Machakos, neonatologist, two paediatricians, two neonatal nurses. Same team Kerugoya, same team in Thika, all everybody was taught online and we had people on the ground. So, we covered three sites. So, by the time we finish the year, we had done eight okay eight sites. Yes, so which was quite some impact created a very good communication network. So, by time we're coming to the New Year, COVID rules had kind of become easy. Now we did, we still did the online for introduction to the hospital HMT, the hospital management team, we still did Zoom, but the trainings were on on site. So, we in the end, we had a huge network of people who had bought into the vision who knew about NEST who knew about the protocols.” (FI 29)* |
| **2L** | When formal agreements have been negotiated and agreed between network members (context), then the network members will feel solidified in a network and be more likely to follow a collective vision (outcome) because this helps network members to understand their roles and responsibilities (mechanism) |
|  | *“Working very closely with the Ministry of Health. I know when the programme was introduced, there was very little engagement of county teams. So the way the programme was introduced, because CIN was already existing, and CIN already had MoUs with hospitals. There was no engagement of the county leadership. So they went straight to the hospitals, had meetings with the hospital management teams, signed MoUs. And that is how the programme operated until last year, which has its limitations, because now as we interact with the county people, some of them don't know about NEST, because also the leadership changes. And what we are trying to do now is to have a meeting with the county leadership to tell them about NEST and change those MoUs from being hospital specific to county specific because as we transition some of our programmes, we want counties to model hospitals to take ownership, for instance, procurement of supplies and consumables. They need a budget for that, at the moment, hospitals rely on county budgets. So, most of them don't have their facility improvement funds. And so their hands are tied, you're telling them to procure things that they cannot purchase. And the counties don't know about the programme because some of the devices require very specific consumables that are not even available within KEMSA at the moment. Yeah, so that's going to change and we already having those engagement meetings.” (FI 18)*  *“Then, I think after that, at some point, we signed a memorandum of understanding with the facilities, all these facilities, because it's very key in ensuring that it's just a binding document, because you see that NEST programme is also a programme that is on a research basis. Yeah. So, involving them and all that there has to be a binding document for this.” (FI 20)* |
| **2M** | If there is no collective vision or if network members do not follow the network’s collective vision (context) then they will be less successful in implementing network activities (outcome) because network members do not feel committed to the network (mechanism) |
|  | *No supporting data* |
| **2N** | When the network makes an effort to disseminate its vision to solve the identified problem beyond the network (context), then non-network stakeholders (e.g. partners, donors) will be more aware of the network (outcome) because network members have advocated for its purpose (mechanism) |
|  | *“NEST has come to revolutionize how we deliver newborn care,” Edith says, “Now everyone is talking about newborn care, because we have created an environment where there is continuous conversation about improvement, change, and collaboration.” (document - NEST360 blog- Opportunity in Adversity)*  *“It has also now made the government think about all this space is small and now we need to expand. So, it's they have been talked about a neonate as also as a person to be taken care of not just as a blanket or their paediatrician yet, paediatric children or whatever we need to talk about. So now we know that we need to invest what newborn care.” (FI 12)* |
| **2O** | When the network engages with government officials and takes their inputs into consideration (context), greater support is generated for the network and network activities (outcome) because there is closer alignment of vision between the network and the government and government officials feel valued and respected (mechanism) |
|  | *“Anytime we have these changes, it's just important for for these officials, the Minister of Health officials to be put to speed that A-B-C-D because as I said earlier, each and every person who sits in an office will always come up with their own system of working of doing things. So even this Ministry of Health, that is what happens. So, whenever they come we had already established a momentum on how we're doing things with the previous officials now you know. So now, of course, the NEST Kenya leadership has to always approach them and ensure that the collaboration still exists. And that whenever we have to involve these people in any activities, we we just have their support as Ministry of Health throughout to be able. And all this can only happen through constant involvement of the new people who have sat in their offices who are now currently sitting in their offices. And as long as they are well versed and with the programme, then everything else will always you know, smooth will seamlessly take less.” (FI 20)*  *“And the Ministry was very supportive because when we did invitations for training, they were under the Ministry. So, people also trusted that this is not a KEMRI project right its being run from KEMRI. But this is Ministry work. So that also I think really gave us huge mileage. And one thing I liked about everything is the things we did, the things we talked about, actually helped people. So, people would try these things we were talking about. And then they would see change. They were like, ‘Oh, we can actually save these very small babies. Oh, if we do phototherapy.’ That for me, one of the biggest ones is the phototherapy work. What when we presented the LED light and we talked about it and taught people how to use it and people stopped doing exchange transfusion because the baby was within one day you don't need to do blood transfusion. Right? So, the interventions were working we had the Ministry.” (FI 29)* |
| **2P** | When network leadership/organisers are engaged in government meetings (e.g. technical working groups) (context), this can improve the enabling environment for the identified problem/collective vision (outcome) because more stakeholders are aware and engage with the problem/vision (mechanism) |
|  | *“And maybe some assistance in advocating higher because it has to cascade from like government level down and it would translate for them into like the health allocation. I think NEST would be like a strong partner to talk about how those, you know, just what they have done is feeding back into the health system and saving money in terms of length of stay morbidity, mortality. If they could advocate at that level, I think it would be good also so that ministry can come in and it can cascade down.” (FI 03)*  *“And that’s because we we had a meeting yesterday. So, the new the new head of the division, all the stakeholders working on maternal child health had meeting together sharing their plans and saying what they’re doing. And what I realised from that from the presentation is that not there are not many people or organisations that are focusing on newborns. So, there are people working on early childhood there are people that are targeting the older children, but very few that are working in that newborn. And it’s, it could be the way that the meeting was mobilised. Maybe there are people doing newborn that have not just been invited and for examples, I was asking them, ‘why is Jacaranda Health not at this meeting?’ And they are they are the people leading on the service delivery redesign project in Kakamega. And so so I think what we want to do when we have a meeting with the Ministry on the 31^st^ of this month with the new leadership is to discuss exactly that, apart from the general TWG. Well, that includes all these people working on, you know, the entire space. Can we have a newborn specific technical working group, that includes, you know, the partners, the organisations implementing this, but also the the technical people – the neonatologists, the University of Nairobi, for instance, are coming together and discussing more country specific policies and regulations that we need to put in place. Like ENAP remains a gap in Kenya, can we discuss, go into more details in those discussions, because again, the general TWG, we really don't have enough time and room to go into the in-depth discussions round the systems and policies that we need to put in place to organise or to govern this space for the country. So that’s something that we are definitely going to work with the Ministry, support the Ministry to strengthen. Its forming a core component of phase two proposal, making sure that that enabling environment that allows allows or enables the country to, you know, move closer to the ENAP target.” (FI 18)* |

### Taking action to solve a problem

| **3A** | In a network formed from the bottom-up when network members realise they alone are unlikely to be able to solve a problem (context), they will recruit likeminded colleagues for their cause (outcome) because they believe collective action is needed (mechanism) |
| --- | --- |
|  | *No supporting data* |
| **3B** | In an externally initiated network when network initiators and organisers realise that they need diverse health system actors to be able to solve the problem/meet the target (context), they will recruit/mandate/encourage colleagues they perceive to be capable of helping their cause (outcome) because they believe a collective approach is needed (mechanism) |
|  | *“And so, I had the responsibility of introducing NEST in the country. It was a new, a new thing that we did, I did that in 2019. And that’s the time when I also engaged Edith so that she could work with me. And I had a choice of choosing many people for that position, who would assist me, but I looked at myself, and saw the gap I have. I’m a paediatrician, I didn’t need a paediatrician. I’m hands-on myself. So, I didn’t need another paediatrician. What I needed was a nurse because I don’t know what nurses do, because I’m not one. So that’s how Edith came on board that we can complement one another. Actually, it was a struggle convincing why not a paediatrician. But I'm happy that we chose Edith. Because she brought something which I didn’t have. Yeah. Yeah. So, we were able to introduce NEST in the in the country.”* *(FI 30)*  *“I think, as happened, there’s always a reason why things happen. And it’s about the networks that you have. So it happens that one of the partners that CPHD had worked with before. We came in quite late into the NEST programme. Okay, the programme had pretty much been approved. Before we were included. And we came in because one of the funders, of NEST Elma philanthropies, was already funding CPHD for us, nurse anaesthesia work. And they felt that this would be a good fit. To have CPHD, Mediquip, as part of the solution, largely for two reasons one we were the only local, truly local partner in the NEST programme. And we had demonstrated core competencies that in their view would be relevant for the sustainability of the NEST programme. So, donor driven in some way we were donor introduced.”* *(FI 31)* |
| **3C** | When network members think collective action is needed to solve the identified problem (context), they will seek out other individuals with common experience or perspectives (outcome), because they believe such people may be willing to help them (mechanism) |
|  | *“It is the the same I know even the other year our unit was get the cleanest and the best organized and it's amazing because one you go to the other units you find the doctor will just write and go away. But here there is a lot of teamwork. For all of us the cleaners, the nurses, the nutritionist, because actually one of the other things that is...being that teamwork has really helped us to identify the problem and be able to ask yourself, who should solve this problem? How can we solve as a team? So, like one of the problem, one time we discovered was our babies were not gaining weight and they were taking very long in the unit. We did a a small study. And the next question was, who should we include in this? So, when we sat down with our team, we discovered we only have two nurses in the unit per shift. Sometime this shift has 75 babies. So, you've got imagine if there are 75, you may be having 20 or 15 that are critical in CPAP or…so when we looked at it, we were like, no, we need more staff. So, then we asked ourselves with… with a team we have a WhatsApp group now for the newborn unit. So, in the WhatsApp, we asked ourselves, who do you think we can add in this group, and we decided we need a nutritionist. So, we work with the administration, and we are given a nutritionist, just for the newborn who has been very instrumental in helping mothers helping calculate feeds, observing babies who are not feeding, gaining weight well, which has also really helped us.”* *(FI 05)*  *“Then we shifted gears a little bit when COVID hit. Cause we were like, well, okay, we can’t do trainings, we can’t go to the ward, what do we do? we stop? So, there’s like two or three weeks, me and Grace, were like, okay, we need to be creative and the priority now is COVID. What do we do? And so, the ETAT+ trainers at University of Nairobi also are now not going to school. So, what do we do? And Grace had a brilliant idea. We should train people about COVID. Yeah. So, what do we do there is something called Zoom. We should use that. So, some of the postgraduate students came up with they read about COVID, and they prepared material about COVID. And then we called a Zoom meeting, we all had to learn what is Zoom. So, we learned about this, how do you use it? How do you download it? How do what happens when you’re in a Zoom meeting. So it was definitely chaotic at the beginning. And what we found during the because we did like three or four sessions on COVID, we realized nurses don’t attend these things. They’re not attending what’s going on. It’s only doctors who come and we’d have like 50 between 50 and 100 people attending. But they were all doctors. So, it’s so we so that was a concern, but we continued doing it and then we decided we need to get the nurses move involved. How do we go about it? And we realise nurses don't know about Zoom. Nurses are overworked with new patients in the ward things have changed, people don’t know what’s going on. So, what do we do so we decided we must do like a rapid intervention. Let’s get nurses on board so for two weeks, I did nothing but teach people how to get onto Zoom.” (FI 29)* |
| **3D** | When members in an established network have access to resources that will help them solve the problem they have identified (context), they may be more prepared/more likely to intend to take action (outcome) because they believe they have a more realistic chance of success (mechanism) |
|  | *“When I need a spare, I could talk to the biomed from the NEST side. I could first of all identify the problem then when I if it comes to a need for a spare I could either call in direct and also WhatsApp in what I what I need then within a very short time the spare brought here sometimes you find the to repair the equipment might be a bit tricky. We could they could also tell us to forward the equipment back to them so that they may also repair from their side then the equipment is brought back again. Yes. So, I do not have challenge when it comes to getting the spares and also repairing the equipment.” (FI 21)*  However, if the network does not have the resources to take action, network members will be less likely to intend to take action because they feel the problem is out of their control.  *“For biomed I think it has been good, it’s one of the best tracks I think. But again now, you find you might solve issues, then what is picked is beyond NEST. Because, we have spare parts because I mean, if you look at the track itself, what are the issues that would affect? It is availability of spare parts, training, which we have done, so they now start picking small issues like, they, how do I call it? They can easily be solved unlike previously which means it is work in progress, I mean they are working. So, you find those, the ones that are remaining are systemic issues, things to do with facility equipment which we sometimes may not have control, or staff rotation but again we can use as programme, we can see what’s the bare minimum that we can be able to, for clinical there is. And then just to mention, there is also very quite improvement in terms of the clinical, the biomed track in terms of data completeness from the facilitators themselves. They are really doing well I would say. In terms of governance we still have a long way to go. I think we still have a long long way to. First in terms of, even the people doing these tracks, it is a bit messy. We have fired a few, we are yet to fire others. Then the people also doing who are representing these tracks at the facility level, they will pick anything, no action point is worked on.”* *(FI 16)* |
| **3E** | When an externally initiated network is forming and opportunities are provided to network members to meet, discuss, and find solutions to problems (context), this will support network members to take action and shape an identity and a sense of belonging (outcome) because they are able to coordinate their work and work together (mechanism) |
|  | *“We have very regular calls for the Kenyan team and the global team to discuss project progress and activities, the scheduled governance meetings and management structures, the steering committee, so there was quite a bit of that in place to ensure that the teams were coordinating the global teams, of course, also visited Kenya, but often to just track progress. Yes, but as you rightly put, it was a big team, very complex team, a very complex project to manage just given the breadth of partners that were involved.”* *(FI 31)*  *“In terms of the network's I would say that initially, we did have lots of meetings within country with the cross-country teams. Almost so within country, we had like a weekly meeting every Monday for one hour, keep track on what's happening with the facility what devices need support, what hospitals need support. In what things need to be addressed, these were relatively new devices, so which hospitals probably are finding it difficult to use a certain device, what do they need, do they need a clinical mentor, do they need a biomed person to go and fix it? What devices are not functioning optimally? So, there is a broken probe? What is the cost of that? How do we fix it? What are the what are the repairs needed for that, so there was a lot of activities happening there.”* *(FI 32)* |
| **3F** | When network members of different roles/cadres/units/organisations work together as a functioning team (context), this enables them to take collective action towards solving the problem (outcome) because they have a common understanding of what needs to be done (mechanism) |
|  | *“It is the same, it is the the same I know even the other year our unit was get the cleanest and the best organised and it's amazing because one you go to the other units you find the doctor will just write and go away. But here there is a lot of teamwork. For all of us the cleaners, the nurses, the nutritionist, because actually one of the other things that is...being that teamwork has really helped us to identify the problem and be able to ask yourself, who should solve this problem? How can we solve as a team? So, like one of the problem, one time we discovered was our babies were not gaining weight and they were taking very long in the unit we did a a small study. And the next question was, who should we include in this? So, when we sat down with our team, we discovered we only have two nurses in the unit per shift. Sometime this shift has 75 babies. So, you've got imagine if there are 75, you may be having 20 or 15 that are critical in CPAP or…So when we looked at it, we were like, no, we need more staff. So, then we asked ourselves, with... with a team. We have a WhatsApp group now for the newborn unit. So, in the WhatsApp, we asked ourselves, who do you think we can add in this group, and we decided we need a nutritionist. So, we work with the administration, and we are given a nutritionist, just for the newborn who has been very instrumental in helping mothers helping calculate feeds, observing babies who are not feeding, gaining weight well, which has also really helped us.”* *(FI 05)*  *“It made us feel yes, we are part of this team, part of that programme. And we are moving on well, another thing when they come to do the supervisions, assessments, yeah, we are doing it together. It's not that they're coming to do their assessments on their own and give us feedback without our knowledge, we are moving with them able to see from the data, there is a very good report, you know, they analyse how we've been performing, we move together, we are able to identify our gaps. And you see now the solutions. Again, they're coming from us. They're not saying that go and do 1,2,3. From all this, what are the possible solutions, we can come up with to bridge this gap. So it is, again, as a team coming up with a solution, something that we can be able to handle at our level. So, I feel they have actually tried as much as they can to involve us in service delivery in that newborn unit.”* *(FI 09)* |
| **3G** | When network leadership or organisers are supportive of network members taking action (context), network members will be more likely to take practical and concrete action to solve problems (outcome) because they feel their efforts are valued and worthwhile (mechanism) |
|  | *“Something else, so that I feel have impacted on the changes is the goodwill of the management. Yeah, because the NEST program, we able to track our performance every quarter, because they're able to generate are reports, every quarter. And through these reports, we are able to tell where our challenges are. And once this is shared with the management, the management now becomes supportive, because we are able to make decisions based on data. That now decision is quite informative. And we feel like if there is a problem with the usage of CPAP, do you need more training is the equipment the problem, and we're able now to address those specific gaps. So, I feel the main use of data is quite quite important, and has really impacted on some of these changes that you're seeing within the facility.*  *And also the QI visits, I feel they are quite quite important as well, because they can means they're about to bring onboard are the four tracks. Those are the health information, the management, that's governance, the clinical and the biomedical and just gently have a discussion and see, where do you have a problem? How can we be able to address this? Is the management ready to support address so that, that as well has really supported in in helping and impacting and bringing about some of these positive changes that you're seeing in the unit.”* *(FI 08)*  *“They always come for the QI visits every quarter. And I must say it's, it's a good thing, they help us understand our problems. Some you have not even realized some we already know, but you don't know how to get to the root cause of it. So, the QI have been have been quite important in helping us improve and become better. Initially, I remember, when they came our biggest challenge was our babies were coming to the unit having hypothermia from the labour ward. And, you know, we saw that we were noticing. But we know when you see the data and you see how bad it is you realise how badly you are performing. And because the QI brings together the clinical team, the management team, at least even though the management team are able to see what we have been saying that we have an issue with hypothermia. I'm not saying it's fully sorted, but you can see they're making some strides towards trying to sort out the issue. Yeah, so the QI have been quite important. And they help us improve, and everyone they want to become better to notice what we are not doing well then improve.”* *(FI 23)* |
| **3H** | 1. When trained network members are provided with support (e.g. quality improvement and mentoring) that is relevant to the problem they are addressing (context), they are more able to take practical and concrete action to solve problems (outcome) because they feel empowered (mechanism) 2. When information that makes challenges visible emerges from supportive activities (e.g. quality improvement and mentoring) (context), network members feel a greater need to address the problem (outcome) because they feel accountable to network leaders/organisers (e.g. mentors, quality improvement team) (mechanism) 3. When network members take practical action as a team (context), this mobilises the network members efforts and resources (outcome) because they feel internal accountability towards other network members (mechanism) |
|  | *“Of course, how can I leave an action plan that I was told in March to leave it up to December it can't happen. So, for them when they come you just put on toes to add on whatever you are given. And it has really brought change. You know at first when we were told of the QI visits, we were we were thinking of it was something like fault-finding. Yeah, but when we when we get to where when I got used to them, I've just seen that the something that just brings change, it's not about about faults. It's how you actually it's just, it helps you to understand how things are done and how they're done based not just doing them.”* *(FI 11)*  *“The mentorship has been largely focusing on some simple things, like skills, for example, in creating and maintaining keep up of phototherapy getting it the right, the right normograms correctly. Those are some of the issues we've had. But also the other mentorship has also come in handy in areas of just documentation. What we have identified as a main gap is documentation...people have done, but they have not documented so the mandate is to tell you or to show you that yes, you are saying you have done but we cannot see it documented. And we have also tried through mentorship, get what we call task shifting. So, get some clerical people to do a bit of work, but NEST also does a bit of support on that one. So those are some of the mentorship areas that we're seeing help us a lot. And just building the confidence of the people who are on the ground.”* *(FI 25)* |
| **3I** | When network members are provided with a platform for them to easily communicate (e.g. WhatsApp groups) (context), they are more able to take practical and concrete action to solve problems (outcome) because they can access and gain the knowledge and reassurance they need (mechanism) |
|  | *“But by that time, we had a technical working group, we created a WhatsApp group. And if if anybody was having an issue in Nyeri, oh how do we deal with this, then people would respond immediately. Maybe you should try this, like live issues are happening. I would put them there people would respond and support immediately.”* (FI 29)  *“There were they were a couple of WhatsApp groups that were created that were specifically for NEST that were very instrumental at that early age for people to bond to chat to say what they’re seeing, to actually say what devices are working what challenges are there. Was the same platform that was used for dissemination of doing information sharing materials, webinars, and getting people to invite people to webinars. So, I think there was a lot of that informal network, so to speak, in terms of in terms of how people interacted at that early stage.”* *(FI 32)* |
| **3J** | 1. If network leaders/organisers continue to put effort and work into solving the identified problem (context), this supports network members to make progress towards changes in practice/improvements (outcome) because they feel encouraged to do so (mechanism) 2. When efforts are made to provide consistent support to network members from network leadership/organisers (e.g. through quality improvement and mentorship) (context), it promotes network members to take action (outcome) because network members develop a bond with those providing support (feeling part of a larger team) and feel empowered by and accountability to them (mechanism) |
|  | *“Yeah, once we’ve identified and we intervene, so our role is are we able to are we able to to maintain it for quite some time for quite some time. And the role of a manager in that department is just to see to encourage, to encourage your staff to maintain that practice, just to maintain that practice. And still, we usually also have our our QI, our QI team from the county. Yeah we should have our QI credits from the county usually visit the department monthly two months. So those are the things they also look at those other things they usually look at because our our workplan plan. After I’ve submitted the work plan to our nursing officer in-charge, she may also she may also see the need of submitting the same to higher level. So that during the QI QI meetings QI assessment in the department, those are the things that are going to base to base on them, yeah. This was your problem, yeah. How far have you gone with it? Yeah. Is there any change from that time to this place? So, I think that is how we do something.”* (FI 07)  *“After training, they didn’t go away and they still are still with us. They come back to do the assessments. You’re trying to find out how are we doing? Where are the challenges? They are able to look at the performance. Where are the challenges? We set targets with them. Yeah, why didn’t we achieve this target? What could be the problems? And the solutions? Again, they tell us we work as a team, they are the solutions that are within us. Yeah. So that involvement, that partnership. To me, I feel very good in that where you feel this one can’t work, they are there to give us a way out. But working as a team, we again come up with a way forward.”* (FI 09) |
| **3K** | If, despite the support provided, network members fail to act or make progress (context), this starts to undermine the relationships between network members and those providing support (outcome) because they feel the network members do not value the effort (mechanism) |
|  | *“The other gap is the monitoring, so between March and maybe the next time we will have, okay let us compare even between February and March, when there was those two QIs, we don’t know what happens in between here, what challenges the facilities experience. Because you know in as much as I want to know, did you achieve your previous action plan?, yes or no. That’s not enough, yes, why were you able to do it? No, why weren’t you able to do it? These are maybe two facilities in Nairobi County so why were you not able and the other facility. You know, just trying to understand the dynamics of this facility. So, I don’t think we have a monitory tool on our side also, or a follow-up, let me call it a follow-up on our end, we don’t have that structure. So, bringing the county onboard and the QI facilitators will help fill that gap. Because as they do their routine supervisions, we will ensure that they give us feedback that, I went to Nakuru and as I was doing support supervision. So that we also don’t handle the financial part of transport, I went to the newborn unit and they were able, they have been able to do this far. However, these are the challenges.”* (FI 16)  *“But in fact some of them call you to ask you “what was my joint action plan? Can you share it with me?” You get? Maybe a week, two, if you call the facilities and tell them, next week you are having QI. Send me the joint action plan for this facility, so that they see where they are. If they are not there, they will so you can’t tell me you will achieve within two weeks. And you see even with these three months you should be monitoring and changing what you are doing in case you are not able to achieve the joint action plan. Like if you read about the QI, the successful QI initiatives, there is always a monitoring in between. Like if I have said I will reduce hypothermia by supplying plastic wraps and it is not working, I change the technique as long as I want to achieve the reduction of hypothermia. So, then that means our facilities don’t monitor. They wait. NEST communicate there is QI next week and then now we start working two weeks to the time. Then we are there, did you achieve? Yes. What was it? Making sticking target it doesn’t make sense what we are doing.”* (FI 16) |

### Identity and culture

| **6A** | When network members with a collective identity come together to solve a shared problem (context), this makes them feel fulfilled (outcome) because of a sense of shared purpose (mechanism) |
| --- | --- |
|  | *“And you see, you are part of that NBUs. It's NBUs this machine is not working, you see in you you are part and parcel of that. And when everything goes well, you know that everything is okay, today we are okay. You feel good. Because you know that newborn that premature baby will be there maybe for one week, two weeks, and if they are, okay. Now with that you see now, in your profession, you are something you are proud proud of.” (FI 24)* |
| **6B** | When network members feel fulfilled from working with likeminded people in a network (context), they want to belong to the network (outcome) because they feel it is worthwhile (mechanism) |
|  | *No supporting data* |
| **6C** | When network members identify with other network members and the network’s vision (context), they develop a network identity (outcome) because it gives them a sense of purpose (mechanism) |
|  | *“Let's say we feel proud to be part of NEST, we feel we refer ourselves as the NEST hospitals. And because it came and changed how we do things. It's changed how we you know, we perceive things, it's changed our outcomes. So, we are very proud to be part of it. I must say, it's like, yeah, it's like a movement sort of. So, first of all, you are always motivated to do the best you can, you're motivated to make a difference for that child. And, you know, you stop doing things just the way you were doing them before, because now you know, better. So, I think it's more than just adding the group of people but from within, they found it changed in you. And then when you come together as a group, I think it's more of a movement. We want to make a difference for the children. So, I hope that can continue.” (FI 23)* |
| **6D** | When a network celebrates the contributions made by previously unrecognized/unacknowledged hospital unit/facility leaders (context), this gives hospital unit/facility leaders an identity in the network as important network members (and in the hospital/facility) (outcome) because their expertise and role is now more valued by others (mechanism) |
|  | *“They became very respected people, even up to now. People know the newborn units are led by nurses and they know what they're doing. Before people think hadn't recognized that nurses are knowledgeable. But now I think that it's something something we achieved to give the nurses an identity and they're very proud. Even when they come to train we look for them for trainings in the area. And that area even that Professor of Paediatrics cannot neonatology cannot doubt it because I mean you have nothing to add because that is what they're doing and that is and maybe you're also not an expert in.” (FI 30)* |
| **6E** | When the identity of a network is linked with network partners/initiators providing resources to the network (context), the network has a separate (not integrated into the health system) identity that may make it less sustainable (outcome) because the network partners/initiators have not put in sufficient effort to integrate the network in the health system (mechanism) |
|  | *“All activities are trying to align with the fact that we cannot continue operating the same model we had in phase 1. Maybe phase 1 was about proving a point that this can work and teaching the MoHs and the governments that if you bring in these changes this will work. So, it was instilling a culture of you know IPC, education all that. They have done a really wonderful job, the education team training nurses training clinicians University of Nairobi, University of Kenyatta, University … Malawi so they have done a lot of work in terms of education, and even the technical universities for the biomedical engineers in terms of* *also distribution. So it’s all in line with trying to make whatever was done now sustainable and the way to do that is strengthening the local health systems may it be distribution part of it, so we will not use the same approach” (FI 19)*  *“But I think the one that is top of my head that we haven't really done a good job at is transitioning NEST to hospitals. Even when you go to hospitals, people still call them NEST devices. If it's broken, people call us, which is not a great thing. Because NEST will not be there forever. So, I think the biggest benefit is this relationships and networks we've created and functional within hospitals. But at the start, I think we ought to have communicated better on what NEST actually is going to do and how much support is going to give and what we expect. So, with that conversation was not had early, early enough. And I think it's something that should be a priority... is still very clear that, you know, by the second year of phase two there should be a lot of efforts to get things happening within the country teams, be it data, be it evaluation, be it reports, dashboards and all that. But I think that's the one thing I would say would need better better thinking, communicating, including you know if we possibly heading towards the five-year mark a lot of those devices that were put in five years ago probably have lived their lifetime. Right? So, they need a new device, for instance. So how do they procure that? Should they call NEST, right? Should they call up the county? And how does that work? So, I think that's something that needs to be to be prioritised. If if we were to transition NEST to something sustainable beyond NEST, I think that would be a priority for me, and also thinking about the degree of support and wherever that goes. So how much of the international teams to what proportion of support from international teams should be be given to countries? And how much autonomy and independence should we be giving country teams to actually take up a lot of these activities?” (FI 32)* |
| **6F** | When network leadership and members consistently and regularly demonstrate and reinforce the network identity (context), this helps other network members adopt the network identity (outcome) because of role modelling (mechanism) |
|  | *“Continue to like, when you have new people inducting them into this is how the unit runs. This is what NEST is, this is the training, this is why you need it. So that continuity can go on even when you don't have to have specific key people to train like a core number of people so that that concept can continue going on and not drop off and data, your monthly data coming back. So, it keeps you on the ball and because you have to keep doing the simple things perfectly. Hypothermia is not just a one-off thing. If it fluctuates downwards, to go back to the upstream, and so data also helps you to to keep up with your improvements show you where you need to keep working.” (FI 03)*  *“Once we've identified and we intervene, so our role is, are we able to are we able to to maintain it for quite some time, for quite some time. And the role of a manager in that department is just to see to encourage, to encourage your staff to maintain that practice, just to maintain that practice.” (FI 07)* |
| **6G** | When a network creates opportunities for members to connect, share experiences, and learn from each other in an open and safe environment (context), this creates a positive network culture and a feeling of belonging to the network (outcome) because they feel respected, valued, and acknowledged (mechanism) |
|  | *“I do and I think they they go out of their way to also make you feel like that, like with the meetings they they regularly call us for where they I asked us to present or present like discuss our points of view, and they bring different centres together. And you realise that what I'm grappling with in my centre somebody else in another part of the country is grappling with the same thing or they have approached it in a different way that has worked. So, I feel so, I feel like we are part of a group and just listening to what other hospitals are saying that they they can see it and they want to join NEST also. So, yeah, so I think we do not just because of the innovations, but also the meetings that make you try and think about some of the interventions and how to get better.” (FI 03)*  When a network calls members for meeting for facilitates network activities, this amplifies their feeling as belonging to a network.  *“In most places, if you don't if you if you don't show up at the hospital, I think people a lot of people see themselves as CIN, it's when we call them for meetings that people differentiate what they're being called for. And the reason for that is one is nothing fundamentally changed in terms of the Clinical Information Network. So, the only issue, the only difference is that hospitals implementing NEST, they got an extra bag of interventions, right. But there is nothing that where there are no activities that were being done as part of the Clinical Information Network that this, these hospitals were told, you can't have this because it's CIN and you can only have this because it's NEST. So, a lot of people tend to differentiate when you call them for meetings, when you tend to go to the hospitals and show up and then say, ‘Oh, I'm here for NEST,’ or ‘I'm here for HIGH-Q,’ because you know, it's the same people who will show up in those places. So, it's when you invite a paediatrician, because those paediatricians, wear very many hats. And CIN is also running a couple of studies. So, so it's when you invite them that you're very explicit that you're inviting them for NEST meeting, inviting them for a CINEMAR meeting, which is a study running in their hospitals.” (FI 32)* |
| **6H** | When local network leadership and members recognise that the network’s culture is not aligned to existing cultures in their environment (context), then they may attempt to change the pre-existing cultures to make room for the network culture and practices (outcome) because they no longer identify with the pre-existing culture and see value in the network culture (mechanism) |
|  | *“Nyeri doesn't have very many deliveries. But we chose Nyeri because of the it has got a good culture of uptake uptake of of of interventions. So, when you're bring an intervention, you at least need to be sure at least in one or two, you're going to succeed. So, it turns a good culture. So that's why we fit it in. We have a team there, although it was not, doesn't have very many deliveries. Yeah, the other facilities, they were there because of the number of deliveries they have.” (FI 30)* |

### Knowledge and skills dissemination

| **14A** | When the network adequately shares knowledge and skills throughout the network by creating training opportunities (context), this changes/improves practices around the identified problem (outcome) because network members are capacitated (mechanism) |
| --- | --- |
|  | *“And then the educational piece, which is also very heavy, I think it's it's unique in a way. Because the first one we do is to provide a course that brings together the clinicians and the biomeds and that allows cross collaborative learning. And what we've seen happen with that is at the facility then there's a lot of collaboration between the nurses and the biomeds and so the nurses telling us that sometimes they're able to see when a device is not working and they can call the biomeds. So that's something that was not happening before.” (FI 18)*  *“We had the programme, they started, it had a fabulous training, we had programmes going on, we are trained on the new equipment, on how to relate with them, and how we can assist the patient. Because they were new and we have many nurses in the department. So, we had to train even the other biomeds, how to assist the the patient now with the new equipment. And we have from the time we had the the other equipment and the added equipment from the NEST. We have a lot of improvement both in the workforce. Because if you have the right equipment, it's easier even for the patient. Maybe she stays maybe three, four days. And the other time maybe we call to you, see now there are good progress, even with the patient now.” (FI 24)* |
| **14B** | 1. When network members disseminate knowledge and skills through mentorship activities (context), network members feel supported and capable (outcome) because they develop the knowledge and skills needed to do their job well (mechanism) 2. When network members regularly disseminate knowledge and skills through mentorship activities (context), network members are more likely to adopt new practices (outcome) because the mentors continuously reinforce the practices (mechanism) |
|  | *“The mentorship, for example, if I'm from Bungoma, there is list that I will take. Like the other time I was in Kisumu, then April I've been in Kakamega. So, you get Kakamega, the normal shift that is reported 730 and they leave at 5. Yeah, you see what they are doing, maybe it's if it's drug administration, you join them and see what they're doing wrong and do what you want to right. Then if it's, then we also teach them on how resuscitation is done, we do recap on warm chain, we do recap on use of failure of warmers and phototherapies. So, you know, when the training and changeovers people might be leaving and the new ones are coming in. So, we do that rotational, rotational mentorship so that even if there is a new staff, they understand what equipments are in the NBU and how are these patients handled.” (FI 11)*  *“Apart from the trainings and equipment, and also I was privileged enough to be chosen to the in the mentorship program. So, I have taken part as the host mentor here in Machakos, where visiting mentors come. And so, we go through in the department seeing what is good, what is the done well, or what needs to be improved teaching. So, I've also participated in going to other hospitals as the visiting mentor I've visited hospitals like Kenyatta National Hospital, have gone to Pumwani, I've gone to Mama Lucy, just during the mentorship programmes, teaching, or even correcting where we find that there is a problem or, okay, not a problem really, maybe there is a gap. Yeah, correcting them or even teaching them, participating even in teaching sessions in the areas where we find maybe they need to improve and those visits have really helped even here in Machakos.” (FI 22)* |
| **14C** | When network members include colleagues in other hospital/facility departments in knowledge sharing opportunities (context), this improves linkages between network members and other departments (outcome) because they respect and value the help provided (mechanism) |
|  | *“But another thing is now the reproductive health team because the labour ward is an extension of my newborn unit, and we work quite closely, we work. It dawned on us that the outcome of this baby's depends on what has been done in the labour ward. And depends on what has been done during the antenatal period. So, we go all the way. And that linkage has really been strengthened by NEST. And in fact, when we were doing the ETAT training, ETAT+ training, and all that, I picked some of the nurses from the labour ward, and even the postnatal ward, so that they know when you talk of newborn resuscitation this is what we mean by immediate care of a newborn. This is what we mean. You know, so they have that knowledge. So more often than not, I would have a joint CMEs to discuss this with obstetricians and all that. So that's really opened our interaction.” (FI 04)*  *“And by you do you doing so you will spot like, either, there is one person who is either quiet all through or afraid, doesn't want to mingle with the others, seemingly suppressed like I don't know when you try to inquire further and you actually come to a conclusion, like he or she is either outshined by the rest, simply because they are either new to the field recently posted or maybe from a different department now they've joined this department and they're like, they have no clue. So that kind of knowledge transfer. Cross team is still something that people tend to grab on it. Some are I'll say, okay, some are jealous to share. Some either do not have the time. Some want to share but the people to be given that knowledge, maybe have no interest, for example…They are avoiding future calls that will replace to them simply because somebody would have known Oh, Vincent was taught. What does that translate to? I want to be going to that department, that department cannot be assigned under me.” (FI 15)* |
| **14D** | When network members include providers in non-network facilities in knowledge sharing opportunities (context), this supports their ability to improve practices (outcome) because they develop essential skills and knowledge (mechanism) |
|  | *“And I think for me, one of the other thing I have been trying. I'm lucky to have been the one of the officials of Kenya Paediatric Association in that region is also to think of not just the county government facility, even those private facilities are killing babies. So how do you improve those facilities? So, with the knowledge some of us have from NEST, how can we disseminate now even to the private facilities? So that is one of the areas we really I have really to think. So, one of the thing is is creating a forum where you if it's a CME being offered by NEST, I disseminate to them. So, I created a forum for the nurse-in-charges from the facilities, most of the facility in Nakuru County, I believe it's even beyond Nakuru County, because they even have in-charges from Yandarwa, which is not in Nakuru County. I also have a forum for clinical officers. So now with this forum of nurses, forum of clinical officer, a forum of paediatrician, a forum of nutritionist so if there is a CME being offered from whoever, even if it is NEST or wherever, then I'm able to put it there for and then I'll ask them whether they will listen to the to the meeting…So at the end of the day, the NEST what we have learned is not only for us, it is even for the people surrounding us, can we bring change. So, I think for me that the parting shot we need to do our best and improve care where we are and in our surroundings not just limiting ourselves to where we are.” (FI 05)*  *“We have linkages with the lower-level facilities. We know those facilities that refer to us a lot. Even when we were doing the training, the newborn ETAT training, we incorporated them into the training. Yes, because we found that they were referring and there could be a knowledge gap. So, we actually took two from the referring facilities and trained. And whenever we get opportunities for training because I have a book where I record all the referrals, referrals, so I see the facilities that refer so much. And whenever I get a training opportunity, I usually call them yeah for training. So, we have those linkages.” (FI 22)* |

### Cross-learning

| **13A** | When the network brings network members together from different professions within the same facility to share their experiences on problems and solutions (context), this facilitates intra-facility cross-professional learning (outcome) because they understand the value of different roles in achieving the shared desirable change/outcomes and feel their challenges are acknowledged and inspired by different approaches from their peers (mechanism) |
| --- | --- |
|  | *“I think what NEST strengthened within the hospital was biomedical engineering. Like before, I forgot to mention them as well, but I feel since NEST that interaction has been strengthened because it was a very technical kind of interaction we used to have before, like when machines have completely broken down. But I feel that has been… since because we do a lot of they do pre-emptive maintenance. And it's now more of a discussion, not just us telling them it's broken down, but also them feeding back how we're using equipment and whether we are using it the right way.” (FI 03)*  *“So, the biomed role has been improved. Biomeds never used to go to newborn units. Now they go. And I like what one of them said that they know if this machine is not working a baby could die. So, they make it a priority to pass through the newborn unit and make sure all the devices are working the way they should. Then another biomed from JOOTRH said we took these concepts we learn about newborn technology and we are doing the same thing in ICU, in medicine. So, the other departments in the hospital are benefiting from the things because the biomed takes care of the whole hospital, right? There are not many, right? So, one or two are located in the newborn unit but they also take care of. So, what we are learning here, they are taking to, they have a path, so that for me, it reassures me that we won't have a graveyard. Because they will ask questions, they will try and look for a solution.” (FI 29)* |
| **13B** | 1. When the network makes an effort to connect network members from different facilities in the network (context), this facilitates inter-facility learning (outcome) because they feel their challenges are acknowledged and inspired by different approaches from their peers (mechanism) 2. When the network makes an effort to connect network members from different facilities in the network (context), this helps them learn new approaches to solve problems better (outcome) because network members can share their experiences and knowledge and see their own work/setting in a new light (mechanism) |
|  | *“Prior to NEST, Kakamega County and Kisumu County are just about 50 kilometres away. But trust me, I had never even visited that newborn unit. I didn't even know whatever was happening there. Currently, it's very easy, you know, you when you have a case, you can call your colleague say, ‘We have this business or that, have you seen this before? How are you handling this scenario? How are you handling this, this, and that?’ So, it's very, it's very good. It's and I'm even called by the nursing team, nurses from other hospitals, you know, they'll be like, ‘Oh, Doc, we have this scenario. We've done A, B, C, D. And probably, we're not getting anywhere, what would be the best thing to do?’ When it comes to consumables, the various things, you know, in the lab, and all that I’m able to ask my colleagues, ‘Anyway, do you have this in excess? Could you give me this for some time?’ You know, and all that.” (FI 04)*  *“Actually for me, I feel is even more advantageous because I get the to visit other facilities. And just apart from conducting the QI as as per the checklist, under the QI facilitator, I am also able to learn what they are doing there, that I can also be able to bring here, and also share to them what I've been doing from my facility that they can also be able to pick out. So, on that track, I've seen one, it helps the biomedical engineers in the facility to feel they're being supported, supported in terms of whatever they are doing, there is somebody externally, that is just coming to see the progress, and also to support in terms of where they have in the weak points. And with that advice, the management level to be able even to support the biomedical within the facility. So, to me that has been working so well, the facilities that have visited and even our own facility because every time I'm out there supporting that facility, there's somebody else coming here to track what I've been doing in my facility.” (FI 08)* |
| **13C** | When the network brings together network members through meetings (context), this helps them to solve problems better (outcome) because they get to learn from each other (mechanism) |
|  | *“I think they they go out of their way to also make you feel like that, like with the meetings they they regularly call us for where they I asked us to present or present, like discuss our points of view and they bring different centres together, and you realise that what I'm grappling with in my centre somebody else in another part of the country is grappling with the same thing or they have approached it in a different way that has worked. So, I feel so, I feel like we are part of a group and just listening to what other hospitals are saying that they they can see it and they want to join NEST also. So, yeah, so I think we do not just because of the innovations but also the meetings that make you try and think about some of the interventions and how to get better.” (FI 03)*  *“One of the challenges we have had is the issue of we have not been doing very well with the CPAP uptake despite the fact that we got the machines. So, one of the meetings or in some of the meetings, we've tried to compare with other facilities, which are doing better. And so, we learned that some facilities were doing what they really invested in CPAP champions. So, these are people who are the ones who are running that idea. And because what we noticed is that after the initial training, over time, the confidence levels went down. But when we got now those sharings and the issue of champions, you know, we've tried to get one champion here. And we have seen that that has helped us quite quite a lot.” (FI 25)* |
| **13D** | When network members have created purposeful relationships through network meetings and activities (context), they are able to continue their cross-learning remotely (e.g. WhatsApp) (outcome) because they have an already established bond (mechanism) |
|  | *“This was part of the initial plans and I'm happy that it got it kicked off well. After the training, in fact it usually starts before the training, so that we're able to do communication early enough to enable them to prepare well. After the training, we do have a WhatsApp group for ease of quick communication. Sometimes they even learn from themselves in those WhatsApp groups. And the good thing is that we do have biomedical WhatsApp groups, clinical team also has theirs. And then we have common shared groups of the focal the focal persons. These are the these are the to go to people that link the programme team that normally and Dolphine and even the other directors and the facility teams. So, they're the people who if we needed to get any updates, quickest possible, they're the people that we go to. So, we have also another common WhatsApp group for such kind of teams. So, this has both biomeds and clinical paediatricians. It's a mix of all those people. Then separate from that we have biomedical team aside, clinical team WhatsApp group aside, within the NEST much larger team. We also have cross-country. I mean, we have a common group for the cross-country teams. Like the training managers, both clinical and technical, we have one that is common for all the four countries. And of course led by the country liaison team that's now Edith and Millicent again separate from that they do have clinical aside for the four countries. And then we have another technical one for the NEST engineers for the four countries together with Hatch team in that composition. So that’s how we we are able to address issues quick as possible. Because sometimes, you will find in some other cases, like for example, let me give you an example of a device we have a device the Billidx Bilirubinometer machine. So far, we've experienced two errors that have been encountered in the field, Tanzania, Nigeria, Kenya, Malawi is yet to report. And those two errors do not exist within the user manual. They are not there. So, we whoever experiences it is free to post on WhatsApp and we try to discuss it there. If it's something that somebody already encountered and was able to manoeuvre around you can share how that you were able to do that.” (FI 15)*  *“For the networking part, they're even WhatsApp groups, like the NEST nurse mentors, the senior nurses, senior newborn unit nurses. So, the interaction is on not only during the meetings, even during the WhatsApp groups, you can ask questions, maybe if there is something that is actually bothering you can posted it in the WhatsApp group and you will get answers from them. Like, for example, a good example yesterday, we had a challenge with our HemoCue. The one that we do the HB the bedside bilirubin bilirubin haemoglobin levels, it wasn't working. And so, I put it in the group. And as the having a challenge with my HemoCue what could be wrong because it's giving me this error? And then I got answers from there I was answered. I after doing what I had been instructed to do. It's it worked. So even those words are very good. Yeah, not only the physical meeting.” (FI 22)* |
| **13E** | When the network makes an effort to connect network members with networks in other geographies (within or across countries) (context), this helps them to solve problems better (outcome) because network members can share their experiences and knowledge (mechanism) |
|  | *“And I think an important thing has been having the four different countries and learning from each other. You know, some are very good at advocacy, others are very good at negotiating their government, or their* *university. And I think everybody's learned from that and also valued the friendships that have been made. East and West Africa don't get together very often. So, it's nice that they do.” (FI 01)*  *“I think that country team interactions with other country teams also played a huge role in reassuring people that challenges are the same and ways to engage with others, government or others to network work well, so so the clinical team would work we in Kenya would work with their counterparts in the other three countries to develop training materials that were relevant and context appropriate for the countries. So, from that perspective, I think that also wider network played a role in in some ways mentoring these national level implementers. And in how to navigate some of the … within their countries based on experiences of others.” (FI 32)* |

### Resourcing networks

| **16A** | When the health system is insufficiently resourced (context), transition and sustainability of the network and network activities may be challenging (outcome) because there is limited capacity to sustain the network’s impact (mechanism) |
| --- | --- |
|  | *“Another thing that has been a hindrance a bit is the space. The space, I only have a six-bed capacity Kangaroo Mother Care, the KMC. So, you find a babies who are stable on this side, just waiting to gain weight that is still in the acute space. Yeah, acute rooms. It is such should be moved. Yeah, so space has been a problem. But that is a capital intense, you know? Yeah. Investment. Yeah, that has been an issue. The other challenge is the finances. Now, the budgetary allocation, Katherine, affects the availability of the lab reagents, the drugs that we need to manage these babies, and all that. And even the non-pharms, you know, things like gloves, things like granulars, you know those things? Yeah, so finances have been an issue. And we are just concluding the something called FIF, Facility Improvement Fund. What that means is that if we collect 10 million, the same same 10 million is sent back to us by the county. Previously, all the money collected at the county level was deposited in one common account and then redistributed at that level. What that means is that if I collect 25 million, I don’t get the 25 million, probably 15 is taken somewhere else, so I’m only given 10. So, I’m unable to sustain the supplies. Right. Right. Right. You see that, so that you’re getting. Yeah, yeah, yeah, but an actual an act has been passed at the county assembly. So, from first of July, the next financial year, all the money will be retained, that will really facilitate the day to day running of the hospital, in general, but the newborn unit in particular. Yeah. So those frequent stockouts can really affect the way we offer services. Yeah, it feels bad to ask a mother to go and buy granulars, you know, especially when this baby comes at night at 9pm. Most of the chemists are closed in town, where will they buy this? So that has been a little bit of a challenge that as I started with my disclaimer is that it is not within the mandate of NEST per se to do this. The team, it’s the county that you would have weighed in a bit heavily to support so that we complement each other. So that one was a bit. It’s delayed a bit but I know we were likely to sort out this. But other is on the side of the NEST, we have said we need more frequent trainings, probably even monthly meetings to reassess we don’t want something to, you know, build up for like three months or so before we move in. Yeah. So, for me, I think NEST has done pretty well.” (FI 04)*  *“They have got their own challenges, things which are, you know, are within their control, and they can do other things which are beyond their control, or very willing and supportive or their teams within the health facility. But remember, at the moment, most of those facilities are dependent on resources provided by their county governments, the funds that they collect, monies, which are available, they go to the county treasury. Then they have to request that process is very inefficient. So, they’re starved for finances, minor things that could be readily sorted out if they had their own budgets, which were reasonably well funded, then you’d be able to sort them out. So, at times, we found that there are very minor things were very frustrating to the hospital administration themselves. They say as you’ve been hearing the cries of the needs and the importance, we understand because we are clinical in the newborn unit, but you have returned to the county government, we are waiting, we also as frustrated as you are.” (FI 28)* |
| **16B** | When an externally initiated network is heavily dependent on external resources and not sufficiently integrated into the health system (context), this will make transition and sustainability of the network and network activities challenging (outcome) because the health system and stakeholders may not be able to prioritise absorbing the required investment (mechanism) |
|  | *“We have a programme that is not integrated into the routing system because if you look at our QI approaches its NEST, and you can hear me say, the NEST QI modules. It's really, it’s programme driven the data system, it's not already, we've not embedded that into the routine system, as much as we are using the CIN platform. If you look at what is the devices at the moment, they have not been. So, most of most of our activities at the moment are very much driven by us. But in recognition of that, if you look at our phase two proposal, there's a very strong emphasis on embedding these things we do into routine systems, and also institutionalisation, making sure that these things are gradually taken over by the Ministry, by the county government, or by hospitals. And we've started doing that already. So, the QI model, the QI that we are doing this year, we want them to be led by the county QI focal person. So, in January, we invited them to a training and then sent them out to go with a QI team. So, for the first time, none of the NEST team members here went out to supervise QI, that was entirely supervised by the QI focal county QI focal persons. Now, the same thing we've done with the biomed. So, for a long time, maintenance of the devices was done by Vincent, our biomed. But we've transitioned that after the transition training, and providing them with the equipment last year, we've entirely transitioned that to county bio...the hospital biomeds. And they're now taking charge of that. And what we do is we provide technical support only. So, we are not running around fixing every broken equipment. We only do that if they're stuck. And they've tried all the options that have been given. And they're not able to handle these, yeah trainings. Yeah. We supported the establishment of the newborn, the the curricula, the protocol that was launched last year, the newborn care protocol. And this year what we are doing is gradually starting to work on integrating the content of that curriculum of the of the protocol into national curriculum, there is someone that's going to lead that process. So that these things are taught as part of normal medical, normal in the medical school, and we don't have to wait for people to get out of college. And then we conduct all these in-service training. So that's also happening. And then, of course, because of institutionalisation. This is the reason why we're even thinking about how do we build the capacity of the Ministry to be able to lead this. So we want going forward into phase two, we don't want to be the ones that are inviting counties to come for a meeting. We want to support the Ministry, if we need a meeting, one to discuss newborn we want that meeting to be called, people to be invited to the meeting by the Ministry of Health because it’s their mandate. They provide the policy and the regulatory framework and NEST when it comes to support with the logistics and if they need technical support. And how do we provide them with the technical support to successfully all that, that's going to be our approach next year. So, we are very much aware that the past three or four years, the programme has been implemented as any other programme that comes and goes. But we don't want that to be the case with NEST, we want NEST to be embedded into routine systems. And even when we step out in 2030, then we can still see NEST going.” (FI 18)*  *“Reflecting on what's happening in the other countries and what's happening in Kenya I would say looking for the right word I would say that a better engagement with Ministry would would because the idea is to if you're transitioning, you're transitioning to hospitals and government so to what degrees government actually involved in NEST, besides them coming to meetings, to what degree are they actually involved in implementation, for instance, and in the stewardship of the next steps. For NEST for instance in some of the other countries, the Ministry is quite involved, they’re saying, ‘This is what we want, this is the places we want you to go to, this is what is going to work for us.’ In Kenya, I don't think we we have engaged Ministry to give some insights on that. But I don't think they're the ones saying, sorry, you can't go there, I think this is where as a Minister within we need more investments.” (FI 32)* |
| **16C** | When the network does not have sufficient human resources required to change practices and sustain the changes (context), this will make transition and sustainability of the network and network activities challenging (outcome) because the network has limited capacity to institutionalise its impact (mechanism) |
|  | *“I would say, NEST has done us well, by and large. One thing that has been not so good, but why why I’m speaking slowly, is that it's not the work of NEST. It's been the human resource. NEST has trained the few human resource, we have quite motivated, but as I've told you, those ratios, yeah. Yeah. So, I have a nurse who is really equipped with a lot of knowledge but he's one person against 25 or 30 neonates like for real, we might not get somewhere with that. So, in fact, the last meeting we had between the NEST team and the county because now we we brought in the county executive was we need to employ, we need to recruit more nurses. For me, I cry for the nurses because, you know, these are the people on the ground. Yes. We need to recruit more nurses, we need the numbers. The good thing is that the county has stepped in and taken two general nurses to go and do neonatal nursing. So, after one year, we expect two newborn neonatal nurses being brought onboard. But the county has also promised in the next financial year to recruit more nurses, so that the numbers improve. The numbers, if you can only do so much, Katherine, you can't attend to everyone. Yeah, yes. Yes. Yes. So that has been a challenge. Because when you give me equipment, you train the few workforce I have and all that you expect some improvement, you know, so these are doing so well. And if you ask Prof Irimu, she will tell you and she usually consults us because she looks and you know, interestingly, when NEST came onboard, and the overall outcome improvement, the numbers just increased.” (FI 04)*  *“Well, personal opinion and observation is, I think, for CPAP, CPAP is one of those very unique devices. Because with CPAP, you need very good if you're giving CPAP, there has to be very close monitoring. But then again, when you go back to the hospitals, our staffing ratios have been bad, so that it's one baby to 13 or 14, we've looked at our data, a lot of the times when just on on the spot checks, our ratios are one to more than 20. But now, when you look at what is involved in actually just setting up CPAP and monitoring CPAP it improves a lot. So, if you're one person with 25, 30 children, you might not be able to have that time to set up. Or if you have that time to set up, you might not have time to monitor. So, in some conversations, I've had things like, ‘No, we don't like CPAP, it kills babies.’ Because a baby was put on CPAP, you see, unfortunately, if you're not able to monitor and change settings then it could be fatal. So, babies have died. So sometimes, you're there you started CPAP, one baby, two babies died. Ha, ‘No. we're not going to do this a third time.’ But it's because somebody has the challenge of actually monitoring this. So, some people just tend to shy away and just not start CPAP. So, for me, I think mostly it's the staffing issues, more than anything else.” (FI 14)* |
| **16D** | When the network shares the collective vision with stakeholders (e.g. partner organisations) outside the network (context), this can help with sustainability and extending the network’s reach (outcome) because they can understand the value of the network (mechanism) |
|  | *“The other thing that I didn't mention is that in the next phase of NEST, we also emphasising a lot on co- investment with other partners. So, this is why we want to have this forum where you know it's the county governments but also other partners in the newborn space. So, if, let's say USAID supplies Mombasa County with CPAP machines, can they get another partner who is working with them to get them warmers. So, it's also going to be about NEST working together with the Ministry to make sure that all the stakeholders in the working in newborn health with this same synergy, and we don't work in silos so that we don’t end up providing 100 radiant warmers to a hospital and they don't have CPAP machines.” (FI 18)*  *“So, this is Ministry of Health and then the other partners that we involved are now partners like UNICEF, during technical working group, yeah. At least I can say we've had an engagement with them whereby they became interested in these technologies, partners like USAID. Let me say, partners in the newborn space, because there are many. Yeah. The partners in the newborn space health partners, yeah. Aside from that I'm trying to look at other collaborations we've had all with the schools. So like, universities that we involved here. So, we've had the Dean for say School of Engineering, the head of department for School of Nursing in Kenya Medical Training College that we have that NEST is targeting.” (FI 20)* |
| **16E** | When the network makes efforts to engage local/sub-national government in the network and the collective vision (context), they can use their skills and resources to support network activities and sustainability (outcome) because they appreciate the value of the network (mechanism) |
|  | *“I think maybe, importantly, maybe looking, in retrospect, one of the things we realised is that we didn't have enough government involvement. And I think this has actually contributed it's one of the challenges in implementation. And I think one of the challenges we have as a programme is because of the devolution aspect as well, because when NEST came in, well the national government was quite aware, but now this sub-national level, in most places was not aware, because MoUs were actually done with hospitals themselves rather than the county government. So I think that is one thing we are working to reverse and yeah …because now, initially, it was working very well. But then you see, as time goes, even leadership in hospital keeps changing. So, when you have a new team, a new leader, see a new nurse manager, a new, and so you go there as NEST, ‘I'm not aware’ or ‘What is that?’ Or sometimes they tell you, ‘Oh, is the county aware that you're here? Don't you think you need to inform the county and that allow you to come into the hospital.’ You know, so I think one of the things, I think where we missed a mark was getting the sub-national level government that we work in getting involved. But other than that, I think we are working well, everybody's happy here.” (FI 14)*  *“This was varying from county to county, it's not all the counties that are the same when we entered into this facility. So, there are some counties where the Director the for Health is very supportive. Like I remember in Kerugoya, we had a very supportive Director for Health. And then in another county, you find that the Chief Officer of Health, like in Embu, was quite involving. So, this varied from one county to another, but these are the people were involved yeah. I can't say that involved all the Chief Officers of Health and all the Directors for Health in these counties. But yes, it just varied from a county one county to another where these hospitals are located.” (FI 20)* |

### Leadership

| **5A** | Networks, initiated and supported by outside organisations/partners (context), will recruit organisations or individuals into leadership positions that they think are competent for network formation and functioning (outcome) because they believe these organisations or individuals understand what the network needs to succeed (mechanism) |
| --- | --- |
|  | *“I think, as happened, there's always a reason why things happen and it's about the networks that you have. So it happens that one of the partners that CPHD had worked with before, we came in quite late into the NEST programme, okay, the programme had pretty much been approved before we were included. And we came in because one of the funders of NEST, Elma Philanthropies, was already funding CPHD for us nurse anaesthesia work. And they felt that this would be a good fit to have CPHD Mediquip, as part of the solution. Largely for two reasons, one we were the only local, truly local partner in the NEST programme and we had demonstrated core competencies that in their view would be relevant for the sustainability of the NEST programme. So, donor driven in some way we were donor introduced.” (FI 31)* |
| **5B** | When networks have a member(s) that clearly takes on a leadership role and focuses on building linkages between potential network members and stakeholders and creates communication channels (context), this helps the network to form (outcome) because it brings people together (mechanism) |
|  | *No supporting data* |
| **5C** | When networks have a member(s) that enacts leadership capabilities, communicates the network’s vision, and helps to set collective goals (context), this helps to bring network members around the collective vision and to move forward in the same direction (outcome) because they have the same understanding of the network’s value (mechanism) |
|  | *“In fact, at that time, I was not the medical superintendent. I was just the in-charge of the department and the hospital management team. Upon sharing with them the objectives of NEST and how useful it was going to impact the outcome of the neonates, it was accepted. Yes, they bought into it. We've got the initial support. In fact, the initial meetings involving the medical superintendent and and others that were held in Nairobi from Kakamega came with that team. And they were very happy. We have received support throughout, yeah, from the time NEST came on board.” (FI 04)* |
| **5D** | When networks have a member(s) that clearly takes on a leadership role and actively coordinates network members (context), this helps the network to form (outcome) because there is a central figure bringing people together (mechanism) |
|  | *No supporting data* |
| **5E** | When networks have a member(s) that enacts leadership capabilities and provides support and feedback to network members (context), this can help the network function and take more appropriate action towards the identified problem (outcome) because network members feel empowered to take action to work towards achieving the vision (mechanism) |
|  | *“I'm always proud of my Med Sup, so, who is the team leader. Yeah, when you go there, it's like, he's mentor, he's a friend. No, in certain areas, staff, you may find staff who are not very, very free with their in-charge, especially the Med Sup. But this one, so long as you are focused, you are, you know, you you are focused to quality to delivering quality services. Yeah, and you you need you want to learn is actually a friend, and he is ready to teach. He impresses teamwork. Yeah, and when there is, if the unit is able to identify one who's not maybe doing very well or has knowledge gap or skills gap. Yeah, they're able to bring her on board. There are some some, yes, we had some who had very poor attitude, they could shout at the mothers, such a person. Slowly, they're brought in and mentored and coached. And you know, somebody by the end of the day understands why is she or he is in that particular team. And with that, I'm seeing great improvement.” (FI 09)*  *“It was a mixed bag. The good there is one ingredient that we don't talk about often, which is the leadership. So, in hospitals, where we deployed NEST, and we had a very strong leader, paediatrician that wanted to see things work, you could see massive improvement in a very short time. In some areas, you had mixed results. There wasn't leadership. Just deploying equipment didn't change much.” (FI 31)* |
| **5F** | When networks have a member(s) that enacts leadership capabilities and gets things done (context), network members develop greater commitment, engagement, and/or motivation (outcome) because they believe belonging to the network is worthwhile (mechanism) |
|  | *“So, in terms of giving the feedback, it is also tricky sometimes because it is around twelve, twelve thirty…exactly, people have finished their shifts they are running elsewhere for locum or something. Then you you may want to miss the biomed the here is where you need the leadership of the facility. But you will rarely get them unless they are very passionate about the programme itself and the newborn. It is very rare to get a full quorum like the one you got in the morning, it is usually very rare. So, what happens is, they fill the joint action plan, they leave it to the they leave a copy in the facility. Then the leadership is supposed to go through it and I don’t think they do that. It’s, I don’t think like, even if you are went to newborn, you know they are supposed to be somewhere. They don’t have, in fact some disappear immediately we leave the facility, and they call back.” (FI 16)* |
| **5G** | When networks have a member(s) that takes on a leadership role and dedicates time to setting up and the functioning of the network (context), network members develop greater commitment, engagement, and/or motivation (outcome) because they believe belonging to the network is worthwhile (mechanism) |
|  | *No supporting data* |
| **5H** | When networks have a member(s) that clearly takes on a leadership role and actively engages network members (context), network members develop greater commitment, engagement, and/or motivation (outcome) because network members feel part of the network (mechanism) |
|  | *No supporting data* |
| **5I** | When network leadership consistently and regularly supports and provides resources to network members (context), this helps network members act to achieve the network’s collective vision (outcome) because the network members feel empowered (mechanism) |
|  | *“NEST is one of the organisations that did not just apply and run away, they have actually stuck with the with the facilities like throughout the life of NEST, as long as NEST is still existing. Then I think it is a culture that was cultivated in at the onset of NEST because most of the organisations, they start a project and they run away. Even when they are still existing, probably they would have supplied the equipment and run away. Now they take care of everything. But here we are, we are stuck. And the facility biomeds are very comfortable. You should do a bit of the questions for the areas where NEST is existing, they will actually tell you, there is a very big change when it comes. There is a big change, there is a big difference between other organisations.” (FI 17)* |
| **5J** | When network leadership and members consistently and regularly promote, encourage, or champion practices or influence network members to take up certain practices (context), this helps other network members to adopt these practices and for them to be sustained in the network/unit (outcome) because of role modelling (mechanism) |
|  | *“Continue to like, when you have new people inducting them into this is how the unit runs, this is what NEST is, this is the training, this is why you need it. So that continuity can go on even when you don't have to have specific key people to train like a core number of people so that that concept can continue going on and not drop off and data, your monthly data coming back. So, it keeps you on the ball and because you have to keep doing the simple things perfectly. Hypothermia is not just a one-off thing. If it fluctuates downwards to go back to the upstream and so data also helps you to to keep up with your improvements, show you where you need to keep working.” (FI 03)*  *“Once we've identified and we intervene, so our role is are we able to are we able to to maintain it for quite some time for quite some time. And the role of a manager in that department is just to see to encourage to encourage your staff to maintain that practice, just to maintain that practice.” (FI 07)* |
| **5K** | When network leadership creates a welcoming, supportive, and inclusive environment within the network (context), members feel more able to seek support to improve knowledge, skills, or manner of working (outcome) because network members feel that they are in a psychologically safe environment (mechanism) |
|  | *“I'm always proud of my Med Sup, so, who is the team leader. Yeah, when you go there, it's like, he's mentor, he's a friend. No, in certain areas, staff, you may find staff who are not very, very free with their in-charge, especially the Med Sup. But this one, so long as you are focused, you are, you know, you you are focused to quality to delivering quality services. Yeah, and you you need you want to learn is actually a friend and he is ready to teach. He impresses teamwork. Yeah. And when there is if the unit is able to identify one who's not maybe doing very well or has knowledge gap or skills gap. Yeah, they're able to bring her on board. There are some some, yes, we had some who had very poor attitude, they could shout at the mothers, such a person. Slowly, they're brought in and mentored and coached. And you know, somebody by the end of the day understands why is she or he is in that particular team. And with that, I'm seeing great improvement. You know, when I look at that, that nursing, the nurses, the nurses that I have, it's like that maternity team, especially NBU, that like a role model, the way they talk, the way they handle stuff and all that.” (FI 09)* |
| **5L** | When networks have processes in place to identify and enable those with the necessary skills, motivations, or attitudes to take on leadership roles from a wider pool of engaged human resources (context), this may support network functioning and may lead to changes happening (outcome), because the network is able to create a distributed form of leadership across the network (mechanism) |
|  | *“The approach was that you see Kakamega County Referral Hospital has been had been in another programme that’s and the Clinical Information Network. So, as the hospital paediatrician, I had been involved in this data issues and all that. So, I was the point person. So, at that point, Professor Irimu reached out to me. And she said that there was this new programme that was coming on board, whose main objectives was to empower the healthcare workers in terms of knowledge and also on the use of equipment or other technology to save or to improve the outcome of neonates in various hospitals. So, at that point, when she requested me to extend that leadership role, and be the point person for the hospital, and also get a nurse, preferably the in-charge of the newborn unit, to also be a focal person.” (FI 04)*  The network may also do this to identify key people in network activity implementation, such as mentors.  *“And then maybe just to mention from from from the training, that is where the team was able to identify mentors. And, of course, we had the focal persons, the in-charges of the board and all that. But now, for somebody who will be a mentor, we had to identify someone from the training someone who looked enthusiastic, and they were willing, of course, somebody had to be willing to be a mentor. I think that is now, that is where I was identified as a mentor myself. So, somebody who is enthusiastic about learning gets the content, clinical credibility, can actually teach, all that. So, mentors were identified. And then now the journey began so the devices got into hospital, then now there was that continuous support with mentorship, from then clinical training manager. And, yeah, the journey continues.” (FI 14)* |
| **5M** | If a network is highly dependent on a few people or leaders to implement network activities or leaders that support their implementation (context), then it is at risk of being unsustainable or functioning poorly (outcome) because the leadership structure is fragile (mechanism) |
|  | *“When when Prof Irimu was handling this, I think she was more how do you call it? She was she was more hands on, which she was more involved. And so, it's made you feel better. And I think when when her influenced stopped, I don't know what exactly happened, I don't so now, it's a bit like the touch somehow, the energy, like it went away, don't necessarily know what happened when the energy went away something from so now I think the activities are just been done without even then used to have I think the clinical services manager that is Dolphine will come around.” (FI 10)*  *“So, the major changes are staff turnover, which is a very negative, has a very negative impact to the programme. I think the other day the Med Sup for Nakuru was moved, I think it was last week we have Machakos who has been moved. So, every time, and I think this is a gap we have like, we have to call but I assume the previous Med Sup is the one who is there. Then you are calling and then they are like ‘no I think that Med Sup went,’ and you are also shocked.” (FI 16)* |
| **5N** | When there is misalignment of priorities between network leaders/partner organisations (context), then one leader/partner organisation may feel they need to leave the network (outcome) because they do not feel they are moving in the same direction (mechanism) |
|  | *“Yeah, we're no longer part of the NEST programme but we're still doing a lot of newborn solutions. I think just the nature of our partnership, at some point we had to just separate because of of programme development issues that were not fully aligned that basically made our continued participation there untenable. So, every contract has an exit clause, we chose we agreed mutually to separate between us, but we're still friends, who still do the distribution of some of the equipment, okay, through our Mediquip global. We are not deeply part of the programme.” (FI 31)*  *“I would first of all, discuss the ones related to the partnerships, management of partnership was one area, I think the priorities of different partners are quite different. We come from an operations we're keen on improving clinical services, in hospitals, not so much from research work done. We have partners who are very much interested in the fidelity of the research and exactly how you're collecting data. So, the level of effort you put depends on on what exactly your outcomes are. So, I think this was a bit of a challenge, because we mixed all these players without clearly separating where they go with the different teams of how if I was to do it, again, for people who want very clean data, I would pick a small subset of hospitals where thorough research activities will be implemented. And not try to go into an operation with a with a research lens that that to me, I saw a lot of push and pull between your core clinical support system and fidelity of research.” (FI 31)* |
| **5O** | 1. When there is a leadership/partner organisation change within the network and a new leader/partner organisation comes in (context), this can jeopardise maintaining the network’s collective vision, existing relationships, and functioning (outcome) because the new leader/partner organisation is external and has not assimilated into the network (mechanism) 2. When a leadership change within the network introduces new working practices in the network (context), this can disrupt network activity implementation and functioning (outcome) because network members need to learn about new practices (mechanism) 3. When a leadership change within the network introduces new working practices in a network without first discussing and negotiating these with network members (context), network members can become frustrated (outcome) because their experience and expertise in the network is not valued (mechanism) |
|  | *“I'll honestly say that it has impacted it has okay let me say it has had an impact on the programme as such that the implementation slowed down at some point, yeah. As a programme manager, I think I can say for any programme manager something that can be frustrating is when you lack… to do something to to implement, to be able to implement, you know. So like, for example, when we move from CPHD, of course, there is a way there was a system on how, you know, you are running a programme, and then move to the next let me say stage where we are out of CPHD, and we have to continuously maintain the programme. And then just as when we are getting the momentum and coming up with systems to be able to implement smoothly, then another transitioning comes on board where we moved now to, I don't know Strathmore University. And then of course, then they have their own policies, they have, you know. Everywhere you go with a partner, they have their policies and system on how they're doing things. So, when you have a programme that is running that has to continue running and on the other side, you have to start abiding afresh, to the other partners, policies and system of how they're doing things you're used to doing. You know, it's not an emergency programme, yes. But it's that kind of a programme where we are managing technologies and if a technology breaks down today, we have to wait for its up time. Yeah, from down time. So, you see like* *maybe, initially, maybe, let me say now after CPHD, we are transition to our own kind of a system with Rice, where we were doing... And then now once we had established that, and we were having a momentum, it's when now we moved to Strathmore. So, you realise, when when you're having that momentum, where there's flexibility on how you're implementing something, you have just established that there is a downtime of equipment. And the biomed engineer, the NEST biomed engineer needs to rush the facility and ensure that because he has received a call from the hospital biomed engineers that are then unable to repair the equipment. So, within that 24 hours, at least, we have to send out the biomed, the NEST biomed engineer, to go, because remember, these babies are relying on these technologies for their life support. So, and ours is to ensure the survival and for these babies to also try as a programme. So, you realise, we have established a mechanism where and that flexibility where the NEST biomed can quickly rush to the facility and ensure the uptime. But now, when you move when we moved to, I'm just giving specific examples, this journey just to show how transition can actually affect a programme. Yeah, so then when you when you move, when you move now to Strathmore, they have their own system of how they do things. So, if someone had has to travel, you have to give a notice, have about 10 days for that to be planned and that to happen. So, it becomes frustrating, because now we can't give 10 days in the baby cannot wait for 10 days, I mean, that's a mortality yeah. For you to be able to give a go ahead and organise transport for biomedical engineer to travel and, you know, prepare the equipment. So those are the kinds of frustrations that were coming along with implementation of this programme. So, as a programme manager, I need to coordinate, I have a work plan in place with the team and the country director. And we know very well, from the work plan in the month of say January, we have these four activities that needs to happen. So in between the activities, if anything comes up with any programme implementation there has to the flexibility. So, you realise when something comes in between the planned activities in that particular month, now, having moved to having joined another partnership here, it becomes difficult to manage anything else that comes in between. So that was quite frustrating, especially the one that you're given restriction when it comes to timelines, you cannot do this at this time, we are time bound we cannot organize a training. In for instance, a training that maybe we could have organised within a span of a week and well-coordinated. No, I'm not talking about planning in terms of I'm talking about now executing the training. Planning of course has to happen probably a month back, but now you want to execute it and because you've moved to the new partnership, and they feel like no we are time bound we are not able to book a conference space for you, we are not able to organise transport for you, I mean it becomes frustrating. Then just as we are settling again, another transitioning happens now from Strathmore to now having to partner with a consultant, you know, to support us with the finance systems, the operation system, basically when it comes to logistics and you know finance. And then just as we are also picking up that momentum, now we have to transition again. Now we are currently in Aga Khan. I mean, it can be frustrating for any programme implementation because even now at Aga Khan they now have, of course, even that consultant had his own system and ways of doing things. This affects a lot the flexibility on how they programme activities can be implemented. And of course, now getting into Aga Khan now fully transitioning to Aga Khan, where now finance has to be, of course, operated by them, logistics, and when you want something to be to be done, there's a lot of bureaucracy of how things are going to be improved, approved. Sorry. And so that for that particular activity to take place, I mean, it's when you start settling down, you have to start over again. I mean, it's that kind of a cycle. So, we've been oscillating around transitioning more than how focusing on you know, programming implementation and ensuring quality of what we desire to achieve. Yeah, so I think that has also affected a little bit of the quality, but even so with that I can say, I can tap our backs, I mean, as the NEST Kenya team that we've actually managed to push through a lot of activities. I mean, just just becoming flexible ourselves, putting the frustrations aside, and, you know, trying to keep that focus and and yeah, moving on. And now I think we actually transitioned fully including the staff like, you know, now settling in under Aga Khan completely. So, I think after we settled in Aga Khan just a few months, I went on my maternity leave so. Yeah, now that I'm back. I'm sure the staff might my fellow, I* *mean, Kenyan team, I think, then they've now been able to, they're sort of getting a momentum. Rather, they're settling down in Aga Khan, and be able to continue with this implementation of the programme. Yeah. So basically, the transitioning has had an effect big an impact big time on programme implementation. Yeah, and it can be very frustrating.” (FI 20)*  *“So, the honest truth is it has had I'm looking for a bet... so I don't want to say it's significant, because you know, NEST is still functional still goes on, but it actually did have impacts on implementation. Mainly, because transitioning, so each organisation had its roles and responsibilities, and there was very little duplication across those organisations. So, then moving from one organisation to another creates like a vacuum, sort of impact stream of work. So, if see CPHD was responsible for looking after the biomedical side of things, then transitioning that to another organisation or figuring how that will work in the absence of CPHD, then that means a lot and the institutional arrangements, therefore, of how, say, colleagues in KEMRI, would reach out to CPHD to get devices for training, then that has now to go to another partner who has to understand why should we give you devices for training? How does that work? How do we loan them? How can we trust that you take care of them? Because the also at an institutional level, there is some amount of time that takes before you can you can establish like a working relationship trust and all that. And that's not just at the beginning, because we transitioned from CPHD, then, I think, had a gap where the team in Kenya tried to run the show, then it was quickly realised that there is a lot of logistics that CPHD would run in terms of coordinating trainings and couriering things to sites that needed an institutional base so we then transition to another institution, which also took a bit of time. And then each institution has its own administrative processes, which now people have to learn how to engage with they need a lead time of, for instance, 14 days to action on anything. And at that point, the implementation of NEST didn't have a lot of we'd already lost time by this transition. So, a 14-day lead time to organise a training. And it's a long time. And things can easily quickly change. So that that I can confidently say that there was some delays in getting some hospitals of the ground, and some of them activities running, as per expected. So, we would have challenges in, for example, having quarterly meetings in good time, or getting the QI activities up and running. And as frequently as they should be, because of those institutional changes, but also leadership changes as well, because we also had, so we had an institutional change, then a couple of months, I think maybe a year later, we had a change in senior leadership, then a couple of months after that we had another institutional change, then another leadership change. So, and I think in all these changes, the hospitals also now have also to adjust to who, which organisation, now are we dealing in terms of when things are sent to them? Where are they coming from? Why are they coming from this other organization? As opposed to this? What happened to the other organization? What does that mean? So, I think, to some degree, yes, that that created some bit of challenges, also, working within the implementation teams. Also, there was a bit of hiccup on how to who to go for what and because each institution, will will get some sort of the understanding of what their role is, which might be different from what the previous role of the previous organisation was. And there are some few things falling through the cracks on who now takes responsibility of this.” (FI 32)* |
| **5P** | When non-network health system leaders, whose buy-in/commitment to the network is important for network functioning, change (context), this can disrupt network activity implementation and functioning (outcome) because it puts at risk relationships essential for network functioning (mechanism) |
|  | *“And the latest one is that the head of the division just changed, three or four weeks ago, of course, that’s a setback, because you’ve been engaging this person in the purpose of, I'll give you an example, phase two. So, we were engaging the previous head, inviting her to our meetings and sharing with her our plans for phase two, and then she's moved. And then someone completely new is brought. And you are at the tail end of your writing a proposal. So, what does that mean, you have to start re-engaging. So, we have a meeting in Malawi, where we are finalising on the proposal. But mean, you have to bring someone new, that means you really have to do a lot of re-orientation of this is what NEST is about that people have also their own interests, they have their own priorities. So so that that then becomes a major problem. Same with the counties, you know. Yeah, but we've not engaged them very well.” (FI 18)*  *“You know, in every country, there is the political will, as to that affects, in addition, either positively or negatively. So, like I can say, when it comes to governance, like as we speak, I forgot to mention this, thank you for reminding me. For governance, you realise that there are changes that are happening even in the Ministry of Health. So just last year, by December, we had, so they like the entire year, last year, by December, we already had changes with the head of, you know, DFH. And and and the unit, the DNCH, so as you began the year, with the new elections, things have also changed again, we have a totally new head, for DFH, we have a totally new person when it comes to the head of the DNCH. So, there has been changes in those with the of the government officials in their offices. So of course, this impacts on the programme, because you see, every time there is a change, as a programme, we have to go and put them to speed. You know, this is NEST programme. And remember some of these people, are not even conversant with reproductive health or newborn health, you realise this is someone who has been put in that seat and has come from a TB departments, from you know, the other side of Ministry of Health or even monitoring. So, this is someone you have to start putting them to speed, this is NEST, this is what NEST stands for, it's implemented here, and here and here. So, you know, definitely, it sort of makes us lag behind a bit.” (FI 20)* |
| **5Q** | When network leadership is not able to get the network to develop and agree on a shared vision (context), then it is at risk of not performing well and becoming unsustainable (outcome) because members are unclear as to what the network is for (mechanism) |
|  | *No supporting data* |
| **5R** | If network members take appropriate action to mitigate any disruption leadership changes might bring (context), then the network is more likely to keep functioning (outcome) because the impact of any disruptions are minimised (mechanism) |
|  | *“In these hospitals, there has been changes when it comes to Med Sups, that turnover when it comes just the hospital management generally. And even with the nurses, there has been those turnovers but at least of course, at the newborn unit, we've been able to, you know, once we've had this job aids and all that. So, the knowledge is transferred within the unit. But of course, of course, with the management, we have to go to each and every facility. Whenever there's a change, you have to put these people to speed and remind them that there is an MoU and you know, so that whenever a study's happened whereas as you continue with the study and something comes up, we don't have to start from square one that how did you even enter in this hospital? Blah, blah, blah. Yeah, so of course, there's that challenge. And maybe the support need here is, of course from the NEST leadership in Kenya.” (FI 20)*  *“I think it's what I said that at the hospital level, because of, you know, this teamwork that had started, you know, way before NEST came in, at the hospital level, there was less of disruptions, it's more at a more senior, much more higher administrative logistics issue to get things done. But having said that, there's the, I would say, the boots on the ground didn't change in a big way. So even when we had a lot of administrative changes, the guys who were responsible for making things run, remained relatively the same. So, you the learning curve was not issue because these people knew what needed to be done. And had, you know, were part of the original NEST plans, were consistently updated through them, meetings, both national and international meeting. So, they were pretty familiar with the implementation processes. So, I would say its the administrative logistic hiccups that would end up slowing things down, rather than the this team, this middle level team that knew what they needed to do. That changed. And I think that played a very huge role to make to still get NEST to where it is. And there was a lot of dedication and commitment from that team. That you know you cannot take away that I, I it's a pretty slim team, looking at the amount of work that needed to be done. And they did a fantastic job. But it's important to add that there was a lot of effort to build capacity beyond the team, the NEST team. So, a good example is with the clinical training. So, when it all started there was a lot of efforts to involve paediatricians at the hospital, but also residents or doctors who were in paediatric training, to learn the skills, the materials, the training, and all that. So that created a bigger pool of people to draw on. And therefore, even if the team leading the clinical education mentorship team was maybe three people, they had a big team to draw on. And those were not linked to any institution but were mainly ministry, government. People who were not affected by these institutional changes. So, if you move from one institution to another it's not now you couldn't use this group of people it's telling them now come for the training. But this the organisation that paid you last time will not pay you, you'll be paid by organisation B.” (FI 32)* |
| **5S** | When there is a change of leadership within the network and new network leadership puts in efforts to maintain network functioning (context), network members feel that it is still worthwhile to be part of the network (outcome) because they feel the leaders value the network (mechanism) |
|  | *“NEST360 Kenya completed the health facilities progress review and introduction of data for action visits at Thika on September 7, Machakos on September 8, Kerugoya on September 13, and Nyeri and Embu on September 14. Facilities were receptive to the new NEST360 Kenya leadership and discussions around data led to action points to help the facilities tackle challenges and promote success.” (documents – Q3-Q4 2022 NEST360 quarterly newsletter)* |
| **5T** | When hospital/facility leadership sees the effort that the network is making and the changes that have resulted (context), this encourages them to take action to work towards the collective vision (outcome) because they understand the value of the work (mechanism) |
|  | *“And even the hospital management has, after seeing that the NEST has come in, is supporting many things these days. If you ask for things, even from the hospital store, the supplies are limited. But you find even from the administration they try. They try to make sure that they provide whatever we need. It's not always enough, but you can see goodwill from the administration, which was not there initially. It's as if they are saying if these others have come in and have done such a good work, how can how can we fail them? Or how can we fail those a newborns or even the nurses who are in the or the workers in the newborn unit? So, you'll find even from the administration that they're really trying whenever we ask them for some things, you find they might not have the resources... But you can see their efforts.” (FI 22)*  *“I think NEST has shown that you can actually make a difference. It has shown that it doesn't have to be the very expensive machines, you need to make a difference. So, I remember initially, the discussion we need a NICU here to make a difference. But then we should just need simple machines, you don't even require ventilators to make a difference, remember the management, I think they have shifted, they have realised you don't require very expensive things to make a difference. And they have seen what NEST has done.” (FI 23)* |

### Champions

| **11A** | When a network provides a platform for members who are passionate and believe in the collective vision (context), they are willing to be champions (outcome) because doing so enables them to initiate change (mechanism) |
| --- | --- |
|  | *“I can say the team leader, actually, who is our Med Sup is like the role model when it comes to all this…And it's like the champion, right, rather than the patron of the whole issue.” (FI 09)*  *“I think the last bit is could be a bit controversial is also networks and and teams really depend on champions. And the success of a lot of this implementation work and and how thing take up and taken up and successful in some of the environments is who the champions are on the ground. I think part of the success for NEST to some degree is because of the champions that NEST had when it started, there were passionate people, there were people who were respected had a good knowledge of the terrain, engaged very well with people at the hospital level. And that played a very huge role in creating this collegial partnership and network of people who were willing to work together. And I think that's something maybe is as can't be quantified or can’t be it's not tangible, per se, but it's it's a software component that we might overlook that sort of played quite a role because of the champions behind CIN the champions so to come in NEST initially and created all these other and mentored other people to actually be champions and advocates for NEST.” (FI 32)* |
| **11B** | When network leaders identify and nurture influential and capable network members and support their leadership capabilities (context), this creates network champions (outcome) because they have the support to enact their skills and passions (mechanism) |
|  | *“And so far, we've been able to train a number of biomed engineers and a number of clinical staff and they were also taken through what we call Generic Instructors Course. So, we have GICs, yeah, they're instructors who are able now from the Ministry of Health, they are from those facilities and expose that we we involve all these levels from from national to county level to facility level. And, you know, we have* *all these people who are now champions and are able to they're well equipped to be able to now even continue training.” (FI 20)*  *“We were able to identify some key, my opinion, people who would carry forth that training. So, in Nyeri, we I identified one person, the ward in-charge was not very wanting to teach. So, I had to identify somebody else who I felt would be left behind to teach. So that happened in Nyeri and actually it was a difficult time for me because the first baby we put on CPAP I think we we chose the baby had other complications but we still put the baby on CPAP and then the baby died. So now people are like ahhh I'm not so sure about this CPAP thing. And also, for me, it was very heart-breaking. Because I'm like I know this thing works. Probably we didn't choose the right candidate. So, what we did to help correct that we had a specialised nurse in Nyeri because I had to move on to Mama Lucy. So, she was one of my students. So, I trusted her and she knows about CPAP and what it can do. So, we left her there for a whole week for the for and she was able to be present, work with the people, and put a baby on CPAP and see the baby actually thrive. So at least that really, really helped. That neonatal nurse actually really helped. And she was able to now quote unquote mentor. That side because she I left her behind for a week. And then after she stayed away for a week, then she came back again for another week. And now several, like six or seven people are comfortable and then now we left them for some time, after a month, we sent her back again, like that. And they were, I think now they were okay to start CPAP and their own newborn champions, were able not to oh these things this thing can do this. So, all those lessons we took to Mama Lucy.” (FI 29)* |
| **11C** | When network champions are capacitated and willing to take on additional responsibilities within the network (context), they will train and mentor network members (outcome) because they see the value and have the skills and passion to do so (mechanism) |
|  | *“It is very difficult for me to dissociate myself from NEST because I think I am NEST. As in when NEST started we were the first people to be trained to become the trainer of trainees you see. I have trained about five county personnel on the NEST bundle, the ETAT+... Kerugoya, Nyeri. I've done those ones Machakos and then Western. There's Kisumu, so I've trained those ones. Now, when we go to train, you know, you train your colleagues. You participate in the training of the biomedical engineering, the mentorship processes, you know, you visit, go see what are they doing there. They also visit, you see what you are doing here. So, for us NEST is not foreign. In Kakamega County, we are part of NEST. Yes, yeah. So, whenever we have visitors coming to Kakamega, it's very difficult as a medical superintendent to say we have visitors simply say our team is here. Yeah, because we have worked with them. And we have seen the improvements in terms of the knowledge base among my medical officer interns clinical officer interns, my nurses, and nurses interns, and all that we have really worked with them. So, we feel like we are part of the programme for NEST.” (FI 04)*  *“And so far, we've been able to train a number of biomed engineers and a number of clinical staff and they were also taken through what we call Generic Instructors Course. So, we have GICs, yeah, they're instructors, who are able now from the Ministry of Health, they are from those facilities and expose that we we involve all these levels, from from national to county level to facility level. And, you know, we have all these people who are now champions and are able to they're well equipped to be able to now even continue training.” (FI 20)* |
| **11D** | When network champions are capacitated and willing to take on additional responsibilities within the network (context), they will help change practices (outcome) because they see the value and have the skills and passion to do so (mechanism) |
|  | *“So that is what we have been working on and then having a champion champion who will bring whatever you want. She is always there keeping the records telling me no [name] this is what happened. It has really helped Nakuru to achieve a lot on CPAP.” (FI 05)*  *“For example, one of the challenges we have had is the issue of we have not been doing very well with the CPAP uptake, despite the fact that we got the machines. So, one of the meetings or in some of the meetings, we've tried to compare with other facilities which are doing better and so, we learned that some facilities were doing what they really invested in CPAP champions. So, these are people who are the ones who are running that idea. And because what we noticed is that after the initial training, over time, the confidence levels went down. But when we got now those sharings and the issue of champions, you know, we've tried to get one champion here. And we have seen that that has helped us quite quite a lot. Unfortunately, one of our champions has also been transferred to another facility, that was our medical officer, but we still have one nurse who is still working as a key champion especially CPAP.” (FI 25)* |
| **11E** | When network champions have pre-existing relationships that they bring to the network (context), this helps the network take action (outcome) because these relationships bring with them extra resources (e.g. access to people, lending legitimacy to the network) (mechanism) |
|  | *“The champions also bring something that's easily visible, like relationships they have with people in Ministry, relationships they have with people at hospital. So those relationships, despite, you know, the mentorship to other people, those relationships that champions have to make things happen cannot be passed on because it was individual things it was something created.” (FI 32)* |
| **11F** | When network champions leave the network (context), this may result in a loss of relationships important to the network (outcome) because these are personal to the champion (mechanism) |
|  | *“I wouldn't say the Kenyan one is perfect but I think there was a lot of effort when NEST started that the champion mentored very many a couple of other people around them that even when they reduced their time and contribution towards NEST, it pretty much hasn't fallen apart. There are bits and pieces that are not as enthusiastic as they would be but it's still holding together. And it's one piece and I wouldn't say it would fall apart if they left but it would be great to still have them. And and the champions also bring something that's easily visible, like relationships they have with people in Ministry, relationships they have with people at hospital. So those relationships, despite, you know, the mentorship to other people, those relationships that champions have to make things happen cannot be passed on because it was individual things it was something created.” (FI 32)* |
| **11G** | When network champions leave the network (context), this may result in a loss of energy to take action in the network (outcome) because network members feel less motivated and encouraged (mechanism) |
|  | *“Like I said previously, when when Prof Irimu was handling this, I think she was more how do you call it, she was she was more hands on, which she was more involved. And so, it's made you feel better. And I think when when her influenced stopped, I don't know what exactly happened, I don't so now, it's a bit like the touch somehow, the energy, like it went away, don't necessarily know what happened when the energy went away something from so now I think the activities are just been done without even then used to have I think the clinical services manager that is Dolphine will come around.” (FI 10)*  *“I wouldn't say the Kenyan one is perfect but I think there was a lot of effort when NEST started that the champion mentored very many a couple of other people around them that even when they reduced their time and contribution towards NEST, it pretty much hasn't fallen apart. There are bits and pieces that are not as enthusiastic as they would be but it's still holding together. And it's one piece and I wouldn't say it would fall apart if they left but it would be great to still have them. And and the champions also bring something that's easily visible, like relationships they have with people in Ministry, relationships they have with people at hospital. So those relationships, despite, you know, the mentorship to other people, those relationships that champions have to make things happen cannot be passed on because it was individual things it was something created.” (FI 32)* |

### Adaptability

| **15A** | When passionate, innovative, and committed network members face circumstances in the network’s operating environment that are out of the network’s control that affect the network forming (context), the network adapts its activities to be able to continue to work towards the collective vision (outcome) because the network members believe in the value of the network and its collective vision (mechanism) |
| --- | --- |
|  | *“I think maybe as it comes or maybe how it was conceived is not how it turned out. Cause of course, it was the plan for NEST was there just before COVID. Then COVID came in, of course, a lot of dynamics changed. Starting from how training was envisioned because they had planned that we'll have five-day physical training on the sites. We know that then now with COVID, people had to shift. Of course, there were no plans for webinars, but webinars came. There were no plans for hybrid trainings, hybrid trainings had to come in. Then, of course, other things that were not planned for. So that to facilitate the like the hybrid trainings, the hospitals actually got television sets just to facilitate the hybrid training, because now you'd have to put the people in a room and then have the lectures presented. There was only say like one facilitator just to coordinate the team on the ground and then have the training running.” (FI 14)*  *“I'm grateful because I was a teacher. And so, I use my my students and I knew where they were and I knew them and I had their contacts. And then I literally went back to my record and looked for everybody's phone number because I knew wherever they were, they were like an in-charge or somebody who is respected, so people listen to them. I literally called them one by one and…have you had have you heard about Zoom and they’re like, ‘what's,’ they were like. So, I realised, people have no idea about Zoom. So, I had the brilliant idea of creating a WhatsApp group. And I had to write down step-by-step, how do you download Zoom. And then I realised, you need an email address to use Zoom. Then I realised nurses don't have email addresses. So now let's start how to how to create an email address. So that's how it started. And I did steps step one, step... because...I already had experience with creating the SOPs for using the machines, so I used same concept. So, break it down, how you prepare, how you create an email, how you download on on your phone. When you download it on your phone, how to join a meeting...next step. When you're in a meeting, this is what you do. Yeah. And for two weeks, that's all I did. Called Everyone sent everybody. Then I also learned about WhatsApp broadcasts, not just groups, you can create a broadcast list with everyone. And you can send them a message as long as they have your contact on their phone, send them a message at once. So, I would have like 300 contacts and my request to them, I would call them and request, please save my number on your phone. So that took so that meant I had to buy the the programme had to buy me a phone, fill it with airtime, so they could save my phone number. So that I could do I didn't tell them about why I needed them to save it. But I was doing that so I could have a broadcast list. That meant I could be able on a click of a button to send to 1000 people a Zoom link, right sharing the code. And that's how we had an overflow. And people now started to get comfortable in using Zoom. And then when we finished the COVID lectures, we thought we could do the newborn series. So again, went back, redid all the lectures, so that they were uniform, again, worked with the postgraduate students. And we would teach we would do the deep scientific dive to the postgraduates like on a Monday and was it Monday or Tuesday, early in the morning. Friday evening we would do the whole country. So, we would test the material with the postgraduates. And then we see how it's flowing. Correct anything any science that is wrong, and then on Friday teach the whole country. So, we started by having the Zoom link which has which was basic, which would get up 100 people. And then we’d lock people out. Then we went to 250 locked people out, we went to 500 we were locking people out, we had to pay for the 1000, and we still were locking people out. And so, it became a thing. Yeah, for like 12, 14 weeks. Every Friday. We had discussions would have a lecture presented the same way. Nurse... a doctor the the science nurse to the practice. And now we started recruiting people to help because now it was too much for me. So now I started getting the neonatal...requirements we had three so they would alternate so we don't get too tired. The postgraduates also they alternate so we don't get too tired. So, Friday we had something going on those 14 weeks. I don't think I slept at the end of it one every hour of lack of sleep, I think. And we were able now to do teach everything the whole content or the priority areas. And then in October so we did that Between April all the way to October and then still COVID was there, we couldn't teach. So, we figured, now we've become masters of Zoom. Let's try, can we get teachers to teach and then just two days and practicals. And we approached, we were in a CIN meeting online, oh yea we also started having online monthly meetings with the CIN sites. And we pitched about NEST and what we want to do and people would give us ideas, people would attend, people people apparently it was a real need and people would listen. And then we finally because everybody was so engaged, always if it's not a Zoom meeting or webinar or something it was a meeting about something, everybody was always informed about what's happening. And because I already had this broadcast, it was very easy to share information, share the slides, share videos, you know. So, it made it so much easier to do that. So, we had all that going on. Again, we figured we can run the course on Zoom. But we need video conference. So, we said ‘who's willing, anybody willing to be a guinea pig?’ And then Kakamega team the nurse-in-charge, Dolphine, , she was the one in charge then. She said, yeah, come try with us. And try we did. And we had 12 lectures with demonstrations video on, we taught. And we did the two days of mentorship. No two days of, we did three days, three days online, two days hands on, onsite. And when we did the three days online, I was present in Kakamega, so that if anybody had an issue, I could read the room and see people are not understanding or if they're not, if they're feeling sleepy, then I'll tell the guys online maybe we need an energiser or please repeat that topic again, we didn't hear you. So, it made it easier. And by the time when we were done. We were done with the three days, two days people came for hands-on. So, we just had stations. And we use the same model neonatologist was the head of the training but we had neonatal nurses and we had paediatricians. And then they would demonstrate the skill use the first stage approach the GIC, you know, so we did all that. And it really helped. When we finished Kakamega, we did Bungoma, we improved.” (FI 29)* |
| **15B** | When network members are reflexive and seek out input from others on network activity implementation (context), the network can adapt its functioning (outcome) because the network members develop a better understanding of the actions they need to take to work towards solving the identified problem (mechanism) |
|  | *“I think the major changes is modification of the working process in terms of implementation. Right now, I think there is a lot of monitoring and evaluation of what we are doing. We are not doing things as routine, so I think there are those, we are evaluating what we are doing, you can see QI we are modifying. You look at the mentorship for clinical, it is being modified. You look at the technical team; they are thinking of coming up with webinars. So, I think there is a lot of modification in terms of the implementation of the programme and trying to see. We are not doing everything across, like maybe you want to implement something, let’s say we will do it in 13 facilities, you know? I think it is being done at context specific, like this facility is struggling with this, how do we go about this? This facility has these issues, how do we go by? So, it is not a one-man thing but it is very specific in terms of what are our facilities experiencing. And then that now informs the interventions that we are supposed to do at the facility level.” (FI 16)*  *“And even the facilities, the staff that we are working with taking into account their feedback, the feedback that they share with us upon, you know, once we do a few … it was just a matter of doing it. It's a matter of how like when we come up as a team from the hospital and NEST then we discuss. We will have a debrief after the visit then of course they share their their aspect of some of the things that should be probably omitted or should be improved or should be added things like that. And with that we've been able to improve a lot by now from the time we started.” (FI 20)* |
| **15C** | When skilled and capacitated network members leave network facilities (e.g. moved to non-network facilities) (context), network members work to restore those same competencies within the network (outcome) because they recognise the value and importance of those skills in the network (mechanism) |
|  | *“Now they we have only been able to do on-job training so that these onsite mentors now are the ones that train them. Because again, I think, as part of the plan may be, in fact, as part of the MoU it was that people who had been trained were not supposed to be transferred from the newborn unit, for at least I think was it two or three years, without unless it was on their requests. But that has not been honoured. Because hospitals will tell you, sometimes, facilities have been opened, governments are not employing, and we need staff, so we have to pinch for here and there or sometimes, yeah. Others just see it is routine for them. But actually, we were able to establish, even from the nursing council that there is no rule that requires that nurses are rotated and all that. So, there are some who have not rotated. But for those ones who have been rotated again, we have to work with what we have. So unfortunately, we didn't have budgets for the training. We only just had to we've only just had to use the onsite mentors just to trains these ones who come in.” (FI 14)*  *“What we try to do and what to try to hope that happens in the field, should there be such a case that some mentorship needs to be done, we hope that amongst the team in the field that already attended the skills training, at least there should be one or two that are still there, because, again, within our system, their county health system, rotations and transfers are always there. So, you'll find that in most cases, and currently, we will face to be the one that ideally all NEST, all five NEST trained biomeds, all got transferred. So, a new team was brought all of them. And the new team came in and then we were like, ‘Hey, okay, so we are starting from square one.’ So, we normally hope that amongst those that we will be transferred, at least one or two should be left behind. So that this one or two, I am able to organise for some sort of tele-mentorship that you could either do on the phone or in the during our webinars. It makes it easier for them because already they have a clue of what we'll be talking about they'd already attended the physical training the onsite training.” (FI 15)* |
| **15D** | When there are changes in network organisers or leadership that influence network activity operations and financing (context), network members will adapt to continue implementing network activities (outcome) because of their resilience and commitment to the network (mechanism) |
|  | *“Let me say, it has had an impact on the programme as such that the implementation slowed down at some point. Yeah. As a programme manager, I think I can say for any programme manager something that can be frustrating is when you lack systems to do something to to implement, to be able to implement, you know. So like, for example, when we move from CPHD, of course, there is a way, there was a system on how, you know, you are running a programme, and then move to the next, let me say stage, where we are out of CPHD and we have to continuously maintain the programme. And then just as when we are getting the momentum and coming up with systems to be able to implement smoothly, then another transitioning comes onboard where we moved now to, I don't know Strathmore University. And then of course, then they have their own policies, they have, you know, everywhere you go with a partner, they have their policies and system on how they're doing things. So, when you have a programme that is running, that has to continue running, and on the other side, you have to start abiding afresh to the other partners, policies, and system of how they're doing things you're used to doing. You know, it's not an emergency programme, yes. But it's that kind of a programme where we are managing technologies and if a technology breaks down today, we have to wait for its up time. Yeah, from down time. So, you see like maybe, initially, maybe, let me say now after CPHD, we are transition to our own kind of a system with Rice, where we were doing ... And then now once we had established that and we were having a momentum, it's when now we moved to Strathmore. So, you realise, when when you're having that momentum, where there's flexibility on how you're implementing something, you have just established that there is a downtime of equipment. And the biomed engineer, the NEST biomed engineer needs to rush the facility and ensure that because he has received a call from the hospital biomed engineers that are then unable to repair the equipment. So, within that 24 hours at least, we have to send out the biomed, the NEST biomed engineer, to go because remember these babies are relying on these technologies for their life support. So, and ours is to ensure the survival. And for these babies to also try as a programme. So you realise, we have established a mechanism where and that flexibility where the NEST biomed can quickly rush to the facility and ensure the uptime. But now, when you move when we moved to, I'm just giving specific examples, this journey just to show how transition can actually affect a programme. Yeah. So then when you when you move, when you move now to Strathmore, they have their own system of how they do things. So, if someone had has to travel, you have to give a notice, have about 10 days for that to be planned and that to happen. So, it becomes frustrating, because now we can't give 10 days in the baby cannot wait for 10 days. I mean, that's a mortality yeah. For you to be able to give a go ahead and organise transport for biomedical engineer to travel and, you know, prepare the equipment. So those are the kinds of frustrations that were coming along with implementation of this programme. So as a programme manager, I need to coordinate, I have a work plan in place with the team and the country director. And we know very well, from the work plan in the month of say January, we have these four activities that needs to happen. So in between the activities, if anything comes up with any programme implementation there has to the flexibility. So, you realize when something comes in between the planned activities in that particular month, now, having moved to having joined another partnership here, it becomes difficult to manage anything else that comes in between. So that was quite frustrating, especially the one that you're given restriction when it comes to timelines, you cannot do this at this time, we are time bound we cannot organise a training. In for instance, a training that maybe we could have organised within a span of a week and well-coordinated. No, I'm not talking about planning in terms of I'm talking about now, executing the training. Planning of course has to happen probably a month back, but now you want to execute it and because you've moved to the new partnership, and they feel like no we are time bound we are not able to book a conference space. For you, we are not able to organise transport for you, I mean it becomes frustrating. Then just as we are settling, again, another transitioning happens now from Strathmore to now having to partner with a consultant, you know, to support us with the finance systems, the operation system, basically when it comes to logistics and you know finance, and then just as we are also picking up that momentum, now we have to transition again. Now we are currently in Aga Khan. I mean, it can be frustrating for any programme implementation because even now at Aga Khan they now have, of course, even that consultant had his own system and ways of doing things. This affects a lot the flexibility on how they programme activities can be implemented. And of course, now getting into Aga Khan. Now fully transitioning to Aga Khan, where now finance has to be, of course, operated by them, logistics and when you want something to be to be done, there's a lot of bureaucracy of how things are going to be improved, approved. Sorry. And so that for that particular activity to take place. I mean, it's when you start settling down, you have to start over again. I mean, it's that kind of a cycle. So, we've been oscillating around transitioning more than how focusing on, you know, programming implementation and ensuring quality of what we desire to achieve. Yeah, so I think that has also affected a little bit of the quality, but even so with that I can say, I can tap our backs. I mean, as the NEST Kenya team that we've actually managed to push through a lot of activities. I mean, just just becoming flexible ourselves, putting the frustrations aside, and, you know, trying to keep that focus and, and yeah, moving on. And now I think we actually transitioned fully including the staff like, you know, now settling in under Aga Khan completely. So, I think after we settled in Aga Khan just a few months, I went on my maternity leave so. Yeah, now that I'm back. I'm sure the staff might my fellow, I mean, Kenyan team, I think, then they've now been able to, they're sort of getting a momentum. Rather, they're settling down in Aga Khan, and be able to continue with this implementation of the program. Yeah. So basically, the transitioning has had an effect big an impact big time on programme implementation. Yeah. And it can be very frustrating.” (FI 20)* |
| **15E** | As the network becomes more established and responsibilities shift to different network members (context), this helps the network adapt to support network sustainability (outcome) because it creates network resilience (mechanism) |
|  | *“We can also have those exchange, I call them exchange lessons and all that. And of course, because there is high turnover of staff, we are thinking of a coaching model. Like how do we come up with not a full training kind of but just have coachers at the regional level. Supporting them with transport may not be a big deal even for the programme and even at the county level. But see calling out people training now and then will not make sense. So, we are thinking of a coaching model where we have coachers for the QI at regional levels. Then they can, and you know the good thing with this is, even those facilities that refer babies, these coachers will go there. So, it is not a one man show of NEST sites, but it is all over. So, if we have county QI coordinator, he is overseer of the whole county, he can be our coacher, we capacity build. He can identify who has been moved to where, who do I need to coach, and all that especially for facilities that struggle with QI and those that are, don’t have active QI teams, they can have the coachers.” (FI 16)*  *“The other thing that we've done is around making sure that we have a large pool of trainers, a large pool of trainers. In phase two, we are going to set up regional teams. So, as we scale up in the new counties, when it's not going to be my team running around to do the training. It's going to be looking into our database and saying that we have trained three or four people in Kakamega, Kisumu, can they go and facilitate the training?” (FI 18)* |

### Teamwork

| **12A** | When network members have opportunities to learn and work together (context), this improves multidisciplinary teamwork (outcome) because they get to know each other better (mechanism) |
| --- | --- |
|  | *“Teamwork has improved. And maybe largely because most of the meetings when we meet for reviews, they they are multidisciplinary. So that helps. So is not a case, where for example, is only the clinician who's attending a meeting, you will … see a meeting, where clinician is there, a nurse is there, a biomed is there, a manager there. So that kind of teamwork, I mean, that kind of multidisciplinary discussions and meetings, they have promoted that whole issue of teamwork within here. And yes, we can confirm that now we are more working more as a team than maybe before.” (FI 25)*  *“So, you know, where people sit around a table during a group work meeting and people actually appreciate that they they know. The hierarchical structures between cadres do not exist in all these other places. So, these these are a lot of teamwork. So, I think that is something that people can quickly learn and benefit from and the spirit of partnership and learning from one another. Because I think there's a lot of that where people have come to recognise that there's something to learn from another hospital, be it how they navigate the administrate the administration to get things done in the newborn unit, be it how they crack the whip on students who are probably not doing their end of the bargain. Yeah, so yeah, so there is always something to learn from that.” (FI 32)* |
| **12B** | When the training provided to network members is multidisciplinary (context), this enables interprofessional teamwork among network members (outcome) because they understand each other’s roles and capabilities (mechanism) |
|  | *“I normally interact with the focal person, the nurse in-charge of the unit. Yeah. Because in the first place, I’ve given him my programme, my schedule for service. So, in the event the event there is any need for for service and maybe I might have forgotten she’s there to remind me. And whenever there is breakdown of equipment, she instantly would call me because she has my contact, she will call me whenever there is any emergencies, we’ll be in touch each and every time. And whenever even I when I do a repair or a service. I normally I normally present after doing the documentation, there is a part where she’s supposed to sign to sign yes after the work is completed.” (FI 13)*  *“I wouldn’t call it a change, let me call it a transformation because it’s a very big change about the the nurses and the biomeds and the clinicians working together for a patient. That’s when you have maybe if they want to fix a patient, they are not very sure of the settings or how we are being, told they include you there. We want to fix this patient. We want this is not coming on the way, we want assistance, that big, big collaboration. And that one, maybe on the department we are seeing of it. But in NBU it’s a big, big transformation there. Yes. Yes. And I think maybe if the Minister or in other partners, maybe they upgrade that cooperation, it works very well, even assisting with the patient. And then machine usage. You minimize those minor breakdowns. Yes. When the nurses and the clinician are that confidence about the machine.” (FI 24)* |
| **12C** | When network members feel they are part of a well-functioning team (context), they support each other and do not feel alone in their job (outcome) because they feel responsible and accountable to each other (mechanism) |
|  | *“And there is need for teamwork. So, this teamwork, everyone feels obliged to contribute in one way or the other to the overall best outcome. So, then you find the biomedical engineers coming in strongly. You find the nurses coming in strongly. You find that administration, the management weighing in strongly, ‘How can we help? Where is the problem?’… So, for me, teamwork has just been the best.” (FI 04)*  *“Yeah, I think it's part of is a very positive programme. And one of the things I like about NEST is the teamwork spirit. Because when you look at how it has made us involved, I know we have started before we had other partners. We have I have been involved in several newborn studies in the unit. Initially, we had a Rotary Rotary grant that we were doing on CPAP. At the same time, we also had the ACTION trial the … ACTION trial, which is looking at steroids and newborn outcomes. So, the way NEST works is different. It is more of teamwork. So, you feel all of you, the nurses, the biomed, even including the cleaner, you have a team so that now you're able to call somebody and say no, this is what I think we should do. So even when you are sitting down and making a decision it will become much easier. Then when like you are dictating what to be done, everyone has a knowledge, they know what is expected. So, they are able to listen to any of us. So, to me that you think that was the most positive thing about what NEST is doing.” (FI 05)* |
| **12D** | When network members feel they are part of a well-functioning team (context), network members feel that they can take the initiative to provide care (outcome) because they feel empowered and understand each other’s roles and capabilities (mechanism) |
|  | *“The teamwork here is so amazing. Starting with the paediatrician, when the doctor says students, biomed there is no specific job or specific person you do everything teamly. You don't wait for that this work conditions for for doctari. No, we also canulate, take samples, yes even calculate the fields. Even if a baby comes twitching, don't have to wait for a doctor to come and collect clinical it within and administer the carrier, the loading those administer. When the doctor come now we can prescribe but at times also allowed to prescribe by our consultant. Yes, you don't. But now that we've been in the drug and the doctor is maybe attending to another question exclusively, like at night we only have one doctor who works in two wards. So do not wait wait wait to prescribe and give the drug.” (FI 06)*  *“It's the teamwork before you find a nurse will wait until the clinician assesses the patient but this is different. Where a patient gets to the newborn, even the nurse is able to assess and assesses decides this patient maybe has suspected this just requires oxygen or requires CPAP. They don't have to wait for the clinician, they actually initiate, they will say this baby needs a random blood sugar, you need to be the one to do it. And then they'll give a give it a plasma, they will even manage before, they then wait for the clinician, presumably because they have been empowered, before they didn't know what to do. Now they know what to do. So doing that improved outcome because now if you've got to wait for that means for the clinician to come maybe the outcome of this baby would have been different. So I've seen a lot of teamwork.” (FI 23)* |
| **12E** | When network members feel they are part of a well-functioning team (context), network members feel that they can take action to change practices (outcome) because they feel responsible and accountable to each other (mechanism) |
|  | *“It is the the same I know even the other year our unit was get the cleanest and the best organised and it's amazing because one you go to the other units you find the doctor will just write and go away. But here there is a lot of teamwork. For all of us the cleaners, the nurses, the nutritionist because actually one of the other things that is...being that teamwork has really helped us to identify the problem and be able to ask yourself, ‘Who should solve this problem? How can we solve as a team?’ So, like one of the problem, one time we discovered was our babies were not gaining weight and they were taking very long in the unit. We did a a small study. And the next question was, who should we include in this? So, when we sat down with our team, we discovered we only have two nurses in the unit per shift. Sometime this shift has 75 babies. So you've got imagine if there are 75, you may be having 20 or 15 that are critical in CPAP or...so when we looked at it, we were like, no, we need more staff. So, then we asked ourselves, with ... with a team. We have a WhatsApp group now for the newborn unit. So, in the WhatsApp, we asked ourselves, who do you think we can add in this group? And we decided we need a nutritionist. So, we work with the administration and we are given a nutritionist, just for the newborn who has been very instrumental in helping mothers helping calculate feeds, observing babies who are not feeding, gaining weight well, which has also really helped us.” (FI 05)*  *“So, you will find that even the way people talk, you know, that teamwork. Yeah, has brought in the the relationship that the way they communicate when you go to that unit and just stand somewhere and see how people are relating with each other.” (FI 09)* |
| **12F** | In an externally initiated network, when there is teamwork among network organisers (context), this will enable better network activity implementation (outcome) because they support each other in their work (mechanism) |
|  | *“And then the other thing is teamwork. I think there is more stronger teamwork unlike before. We didn’t have an office clinical, biomed, QI, and he is the Covid person. I think right now there is that coordination, there is that teamwork, like what am doing is building on what Dolphine is doing and what Dolphine is doing is building on what I am doing. I can tell Vincent what is happening and he can tell me ‘Hannah I need your assistance here or we need to work on this.’ Yeah. So, it is a matter of a lot of teamwork and coordination which again I think leads to better implementation of the program.” (FI 16)*  *“But there were other areas which hadn't been as successful, most of them is like, you know, the the relation, the teamwork, so to speak, within the country of the different players. Because you had the roles can be a little bit conflicting. And getting to know, you know, where do you draw the line of who does what, who doesn’t do what, I think there's a bit of people may be feeling like, you know, their roles and budgets weren't very clear that stage. So, when I came in that was at least, you know, one of the things that we needed to do like streamline, you know, those things. Like there were situations before that like the trainings on biomeds and the clinical teams would happen separately with very different schedules for no good reason yet. We are supposed to be promoting as NEST interprofessional working and trainings. So, we had to get at least those ones streamline. Then we said there is no need of having a parallel planning and trainings. So that was a call to streamline that we got the teams working on those to work* *at the unit,* *run logistics together and deliver the trainings jointly and that was useful in getting teamwork built…Team, you know, teamwork issue, and that means working in facilities together with the Ministry of Health with counties. So that's what was our priority.” (FI 28)* |

### Psychological Safety

| **9A** | When network leadership/organisers are available and approachable, invites input and feedback, and models openness, fallibility, and non-judgmental behaviour (context), then this promotes a network’s psychological safety (outcome) because network members feel empowered and not threatened when they speak up or make a mistake (mechanism) |
| --- | --- |
|  | *No supporting data* |
| **9B** | When network members form trusting and respectful horizontal relationships (leading to flattened hierarchy) (context), then this promotes a network’s psychological safety (outcome) because network members feel equal (mechanism) |
|  | *“So, you know, where people sit around a table during a group work meeting, and people actually appreciate that they they know, the hierarchical structures between cadres do not exist in all these other places. So, these these are a lot of teamwork. So, I think that is something that people can quickly learn and benefit from and the spirit of partnership and learning from one another. Because I think there's a lot of that where people have come to recognise that there's something to learn from another hospital, be it how they navigate the administrate the administration to get things done in the newborn unit, be it how they crack the whip on students who are probably not doing their end of the bargain. Yeah, so yeah, so there is always something to learn from that.” (FI 32)* |
| **9C** | When a network has a shared network identity and culture that promotes a psychologically safe space (context), then network members are more likely able to learn, improve, and seek feedback (outcome) because they feel empowered and a reduced fear of negative consequences (mechanism) |
|  | *“What we do for other hospitals is when we are doing our mortality audit and it comes up that we get quite a few bad outcomes or poor outcomes from a particular hospital, we try and invite them for some of those mortality audits, not as not in a vindictive way, but to understand what they’re working with. And we’ve learnt a lot, we’ve learned that some centres, the staff are doing what they can, but they’re not equipped or trained. And when you create that safe environment, like they can be tell you some of the things they do like what they do for resuscitation and then that becomes like a point of, okay, we probably need to reach out to them. So, when we have trainings, we make a point of trying to invite some of those centres to come in or to also tell them what they need to advocate for from the hospital administration, some lack things as basic as oxygen. And he’s just sent in an empty van at the back. So yeah, it helps to contextualise some of those things. But going down, that’s like part of the plan, that we have like to actually go there and see what they have, instead of blaming them for poor outcomes to go and see what they have, what are they dealing with, and how we can help them.” (FI 03)*  *“Of course, how can I leave an action plan that I was told in March to leave it up to December it can't happen. So, for them when they come you just put on toes to add on whatever you are given. And it has really brought change. You know at first when we were told of the QI visits, we were we were thinking of it was something like fault finding, yeah. But when we when we get to where when I got used to them, I've just seen that the something that just brings change, it's not about about faults. It's how you actually it's just, it helps you to understand how things are done and how they're done based not just doing them.” (FI 11)* |
| **9D** | When a network is a psychological safe space for network members (context), it enables members to openly raise concerns or problems (outcome) because they know they will be supported and there will not be negative repercussions (mechanism) |
|  | *“In the beginning, you can you can tell it's the they might not be but we really try to moderate the tone of the meeting. So that it's not fault-finding, about blaming, we just want to understand the process they went through. And we've seen, like some cases, we thought were cut and dried that this maybe it wasn't so easy. And they you realise that the staff might be trained and experienced. But what's available in terms of equipment and monitoring for them to use is...by inviting like, there's a case where we realised we didn't have the answers, even after talking to them because the person who came was an experienced midwife. And we felt if we had post-mortems, for example, for some of these babies, if it's acceptable to the family, which is a different discussion, we might have learnt a bit more about why they presented the way they did. So, we try to make the tone of the meeting one where we are meeting as colleagues to discuss our our the things that we are grappling with and what we also try to inverse it. So, when they tell us their challenges, we tell them our challenges, too. We say like see, we have issues with documentation, we also did not do this. And we asked for their feedback. Like how were you handled when you came? Did you feel victimised? So, that we can go back to the team and tell them to be careful how we handled them. Because we're one team and we need to make people comfortable talking, voicing their concerns.” (FI 03)*  *“They will be defensive initially, ‘oh no, we have we have shortage of staff.’ We told them it's not about shortage of staff it's about how do you reorganise your work to do to ensure, yes. So, they they took it.... So they were a bit ambivalent but and we told them but we used to tell them we have come here to do a soul searching for you to help you grow before somebody else externally comes, identify your gaps, before something wrong goes goes up, comes in comes out, and then you are exposed to media before so we'd rather you know it now. And you will say this is what we are doing. Yes, we have a shortage of staff. But this may have happened because of an oversight of this. And you can, you can link it to a root cause which you are you have already been communicating with the county, we've been telling you about this one. So, it helps in them knowing things in advance, be able to escalate it to their senior. So, their seniors when things go wrong, their seniors are able to say ‘no no, but I've been told of this.’ So, they'll take things seriously. So that's what I can say on my participation on governance. On clinical teams, I don't know whether they have been maybe you and get people who have. But but but there have been receptive over time, they have accepted and since after every they've seen they saw that there was nothing punitive. And it was helping them do their work better. So, I can say when I go, when we go to visit to now the there is like, we are friends now they don't. They're not defensive. They accept and they say what, then they say what they will do.” (FI 27)* |
| **9E** | When a network is a psychological safe space for network members (context), it encourages innovation and innovative behaviour (outcome) because they know they will be supported and there won’t be negative repercussions (mechanism) |
|  | *“And I think the net should I say, the network that we have that involves bringing people together in the programme review meetings, the quality improvement initiatives that we have, the mentorship that they have, those are things that are very unique. And to give you an example, in Kakamega County recently invited us to their QI Technical Working Group. And they really love their QI model, one approach that is used by NEST and the team that visited of course they made a presentation. So, they've requested that we support them to develop their team, the QI county specific QI that is just not specific to the newborn, but cuts across the entire the entire hospital. The same thing we are hearing from the Ministry, so the Directorate of Quality Standards, they recognise that the approach that we're using for QI is something that is also very unique. And the the the process of integrating or trying to embed that approach. Because they've been leading our QI into the national you know within the routine system. So I think I would say yes, that is something you people people know that NEST it's a programme, it has its unique characteristics. Of course, some are good, some might not be good. But they do know that. Yeah, that this is something that is definitely their part of, should I say, a programme or a project or an organisation that is really keen on improving quality of care.” (FI 18)*  *“I know there was an improvement when it comes to management of neonatal jaundice and when it comes to CPAP use, so looking at neonatal jaundice, the staff were well trained and after the training, they continued with on-job trainings within the unit. And of course, and of course also with the unit that is referring to them, but that's now the maternity. And this way other than jaundice, they were able to also improve on hypothermia. Right from the time the baby is being transferred to the newborn unit from maternity, they from the training, it helps them even to become more innovative around keeping that warm chain of the … babies especially from referred to from maternity, all the way to the newborn unit. Some of them came up with you know, the wrapping, they using the wrapping bags, and you know, ensuring that they warm chain is kept until the baby is admitted in the newborn unit and is in the unit newborn unit, neonatal unit staff hands.” (FI 20)* |
| **9F** | When a network creates a psychological safe space for network members (context), it enables them to more easily communicate and collaborate across the network’s facilities, levels, and sectors of care (outcome) because they already have a common ground and understanding (mechanism) |
|  | *“It has been very positive because it is not really it depends with how you do the audits. If you do an audit like a blame, you may not achieve much. But if you do an audit, ask ourself where did we go wrong as a team, so it will become much easier for them to be receptive. So, they have been fairly receptive. Even when they refer, I know the other week one referred and I told her, you will have to bag this baby until we stabilise, and that is what we did. We bagged with her and we tell her no, that is not how you hold the … bag you do it like this. They were very they are very it depends on the communication you communicate to them.” (FI 05)*  *“And then yes, and then communication in advance. Yes... when they see us national team when they are seen on the ground, they tend to panic what did they come to do. We have a senior officer coming from the Ministry. Could there be something wrong? Something like that? Yeah. So so that there was that so so maybe it's a communication so but now communication later on went when they were all brought to the other centre with 13 hospitals that all the bosses saw us. And you are the ones also facilitating they now even when we go there, we don't need that a letter from the ministry that you have now. Yes... like now if I go to the county, like now I'm going to for that county I had to show them a letter. And we had to communicate them in advance. But for NEST now, you can come now that we have bonded we don't we will just say we're coming to your organisation. This one what I need is only a release letter from the Ministry.” (FI 27)* |

### Commitment

| **7A** | When network members identify with the network’s collective vision, identity, and culture (context), they are more likely to be committed to the network (outcome), because they believe in and value the vision, identity, and culture (mechanism) |
| --- | --- |
|  | *“It's just, it's an important initiative. I think it's a very it's it's it's made a difference. And I wish that difference that it has made can be highlighted. So that it's important to see. And it's not discussed as at policy levels as something that might be done. It's essential. Like what we were doing before, it's not safe. We can only wish that it will scale up and go farther, maybe in the future, if they're thinking about innovation for other types of equipment. That would be something exciting would be like to hear and contribute to things. There are still things that we grappled with things like monitoring, continuous monitoring, things that we would like to have and a technology to allow that would be good.” (FI 03)*  *“It is very difficult for me to dissociate myself from NEST because I think I am NEST as in when NEST started we were the first people to be trained to become the trainer of trainees you see. I have trained about five county personnel on the NEST bundle, the ETAT+... Kerugoya, Nyeri. I've done those ones Machakos and then Western. There's Kisumu, so I've trained those ones. Now when we go to train, you know, you train your colleagues. You participate in the training of the biomedical engineering, the mentorship processes, you know, you visit, go see what are they doing there. They also visit you see what you are doing here. So, for us NEST is not foreign. In Kakamega County, we are part of NEST. Yes, yeah. So, whenever we have visitors coming to Kakamega, it's very difficult as a medical superintendent to say we have visitors simply say our team is here. Yeah, because we have worked with them. And we have seen the improvements in terms of the knowledge base among my medical officer interns, clinical officer interns, my nurses, and nurses interns and all that we have really worked with them. So, we feel like we are part of the programme for NEST.” (FI 04)* |
| **7B** | When network members’ professional identity/calling align with the network vision (context), they are more likely to be committed to the network and enact affective commitment (outcome) because they professionally value/find importance in the network vision (mechanism) |
|  | *“The nurses that come for neonatal training they're passionate, really into newborn care. And it really helps because at that point, I'm not pushing anybody just working with people who want to work and want to be there. Yes, and even our registrar's who come in not necessarily, want be wanting to wanting to be a part of newborn care, they inevitably catch on and even they go out there and make changes to some extent. So, I suppose those neonates change you, neonates have a way of changing.” (FI 02)* |
| **7C** | When networks members have support from network leadership/organisers (context), they are more likely to be committed to the network (outcome), because they feel valued (mechanism) |
|  | *“And I can say that one of the things that I've learned, because we've had quite a number of programmes are running across the health sector, with the NEST is a very unique programme. Why could, why should I say it's unique? With NEST actually, it goes back to the users or the clients or to a point the facility. And it narrows down not just at the facility management, because some programmes will work with the managers, then you're just given these what you're supposed to do and all that. But with NEST it's narrowed down to the people that are hands on, for our case, the devices, the people that are really with the devices on a day-to-day basis. And with that, we feel we are part of the programme because you're able to contact a technical person in the programme that is readily available to assist in terms of selling as well as in terms of assisting even troubleshooting in terms of diagnosis in terms of repairing, and even at some point they can even be able to come and physically support.” (FI 08)* |
| **7D** | When networks enable members to achieve professional norms (that are part of their professional identity) (context), they will be committed to the network (outcome) because of affective commitment (i.e. the alignment between their professional and the network’s norms) (mechanism) |
|  | *“We have also seen people now interested in practicing neonatal medicine or going back to school to study to specialise. Whether it be it's nurses, be it doctors, and no one now is afraid in terms of taking care of a neonate. I think we better placed.” (FI 12)* |
| **7E** | When a network member gets ‘emotional’ benefits (positive feelings) or feel a sense of purpose from being part of the network (context), they are likely to be highly committed (outcome) because it is fulfilling for them (mechanism) |
|  | *“I’m actually proud of NEST. Why am I saying like that is because when they came on board, they actually involved the the that management of the hospital. I’m one of the people, I’m the nursing manager, and I was involved in the training, they trained me. And, you know, once the training was done, most of the things, whatever is happening on the ground, I am actually part of the that activities that are being implemented on the ground. And the thing that actually motivates me more is that I’m able to look at this journey, how we started and where we are heading to. Or that’s just a motivating factor. Because, okay, initially, we had a small room, and with so many patients, and you know, we didn’t know how to go about it. Yeah, there is, there are so many patients, we are few nurses there, we don't have resources to use. So, most of those babies were dying, and it was very demotivating. So, looking at this journey, it's a motivating factor, because now we can look back and see where we've come from.” (FI 09)*  *“So, I'm really happy for this programme, it has made us to be up to the task of just not doing things, you are doing things knowing that at the end of this month, I'm being monitored, what have I done right? What have I done wrong? You see and it was good to have… know when you go to meetings and you see that glucose monitoring Bungoma you at number 10 here, but Kakamega is number one. You want to see I'm going to Kakamega that the nurse there but that you're doing right so that I can reach you on. NEST has opened up our minds as a programme.” (FI 11)* |
| **7F** | When a network shows network hospital/facility leadership it can improve care practices (context), this can generate commitment from them (outcome) because they see its value (mechanism) |
|  | *“Also, something else, so that I feel have impacted on the changes is the goodwill of the management. Yeah, because the NEST programme, we able to track our performance every quarter, because they're able to generate our reports, every quarter. And through these reports, we are able to tell where our challenges are. And once this is shared with the management, the management now becomes supportive, because we are able to make decisions based on data. That now decision is quite informative. And we feel like if there is a problem with the usage of CPAP, ‘Do you need more training? Is the equipment the problem?’ And we're able now to address those specific gaps. So, I feel the main use of data is quite quite important, and has really impacted on some of these changes that you're seeing within the facility.” (FI 08)*  *“The newborns, the premature babies, yes, there was really a problem, because we didn't have the CPAP machines. And so, we used to lose many of them, we use to lose many. But at least with the the CPAP machines, we can see that we are saving many. Yeah, and it's not a procedure for a specific person to do. Anyone with a baby will always admitted respiratory distress syndrome, the nurse that is found there, it just starts CPAP. So, the newborn care has actually improve. And even the hospital management has, after seeing that the NEST has come in, is supporting many things. These days, if you ask for things, even from the hospital store, the supplies are limited. But you find even from the administration they try. They try to make sure that they provide whatever we need. It's not always enough, but you can see goodwill from the administration, which was not there initially. It's as if they are saying if these others have come in and have done such a good work, how can how can we fail them? Or how can we fail those a newborns or even the nurses who are in the or the workers in the newborn unit? So, you'll find even from the administration that they're really trying whenever we ask them for some things, you find they might not have the resources... But you can see their efforts.” (FI 22)* |
| **7G** | In an externally initiated network, when the network initiators promote the efforts and accomplishments of the network to donors and the interested global community (context), this increases network member commitment to the network (outcome) because network members feel their efforts are acknowledged and valued (mechanism) |
|  | *“Significant progress was made, despite the challenges of a global pandemic. In 2020, NEST360° faced the challenge of confronting and responding to a global pandemic, which demonstrated the resilience and commitment of the whole NEST team — a network of doers with enthusiasm and zest, who show up to do the work each day.” (document - annual impact report 2020)*  *“NEST360 continued to exceed expectations in Q1 of 2022! NEST is installed in 64 facilities across Malawi, Kenya, Nigeria, and Tanzania. We offer our sincere gratitude to all the NEST360 partners, Ministries of Health, clinicians, nurses, biomedical technicians, mothers, and babies who are part of this successful milestone. We are excited to share our First Quarter 2022 accomplishments!*  *Country-led change is underway in all four NEST implementing countries. The teams have accomplished incredible work that will support progress towards sustainability and institutionalization efforts.” (document – Q1 2022 newsletter)* |
| **7H** | If a network has committed and proud members (context), then they are more likely to act on the identified problem and collective vision (outcome) because members are willing to put in the energy, effort, and passion (mechanism) |
|  | *“I do and they see it. I see it in our nurses like they do things that it's above and beyond she doesn't have to do it, she can do her core duties like my my charge nurse or my nurse in-charge of NBU. They really do a lot of extra to to to improve how the services are being done, you know, like advocacy or even just getting a bit of data. Some maybe, like we she we compile our own monthly reports. It's a bit different from what we get with CIN because it's for inpatient. Talking about the things we need, what what we're missing, we get a lot of support also from our obstetric department, we meet in the MPDSRs, like the head of department herself will come or one of the other consultants. So, I do feel people are passionate because those children in that NBU, even if it is in Eastlands, which I know is a low economic status area, they deserve as good a chance as anybody to survive intact not just survive, because some survival there are issues, they have issues that will be lifelong or long term. They deserve a chance to survive intact and pursue their dreams and their lives. This you don't know what they will become. And I think it's a really fundamental thing for a country to protect its weakest. And its most vulnerable. And for me, those children represent that to most families represent that. And I think most of us we look at it that way, we want to tide them over that that period so that we can they can grow and thrive. So, I feel it.” (FI 03)*  *“Actually, you can feel it, I think that one of the challenges we have would be, first of all, let's talk about the passion. You know, passion is there, you find people come report to us very early, they will leave very late, you know, we have that shortage by and large. Our NBU you know sometimes it goes up to 65, 70 neonates, but then that's on some shifts actually can be against three nurses. But then it becomes really difficult you know, but you will see them work. And, you know, sometimes you sit down and say, ‘God, this is just great.’ If you don't have that push that commitment, believe me, you will not, you will give up. And they're so passionate and they feel bad. You say doctor, we've lost this baby. We did our best, but it is very sad. You know, you can read it. Yeah. So that passion is there. The passion is there. We just need now at the county level to motivate these people. Motivating means, for example, increase the number of workforce, yes, we need a fairly good ratio between the healthcare workers and the patients to have that one on one attention. Yes, as I say, you can see them you can read a bit of frustration because if you're in a room where you have 25 neonates and like some of the rooms, the acute rooms and you will find three or four babies are on CPAP. You know, CPAP really needs close monitoring, like initially, you know, quarter hourly and all that. So, you have like three or four on a CPAP. There are others on oxygen, they need the* *SPO monitoring that these ones who need fluids adjustment, they need that and that. So, they become a bit overwhelmed. So, if it were not for the passion, they would just give up.” (FI 04)* |
| **7I** | When committed network members take action to disseminate knowledge to network members and others outside the network (context), this extends network benefits to network members and others outside the network (outcome) because of a greater availability of knowledge and skills (mechanism) |
|  | *“The clinical training team did not give up and went on a campaign to train everyone (especially nurses) how to download and use Zoom. Three nurses played a key role, using existing WhatsApp groups. They took time to list out the steps to download Zoom and then and explain how to use the software. This extra effort bore so much fruit, and it was evident with the number of people who wanted to join in the webinars. The clinical team had to increase their Zoom account maximum capacity numbers from 100, to 300, then 500. Registrations for the webinars would go up to 2,000 sometimes. In addition, the audience evolved to not only Kenyan, but also health workers from Uganda, Nigeria, Cameroon, Ethiopia, Rwanda, and Mauritius. The webinars were held from April to July 2020 every Friday evening and during this time, the clinical training team became masters of using Zoom to run trainings. Building on this experience, the training team was able to transform the face-to-face newborn care trainings into a three-day virtual training for specific facilities, and sometimes they were able to combine up to three facilities at the same time.” (documents – annual impact report 2020)*  *“And then when we finished the COVID Lectures, we thought we could do the newborn series. So again, went back, redid all the lectures, so that they were uniform, again, worked with the postgraduate students. And we would teach we would do the deep scientific dive to the postgraduates like on a Monday, and was it Monday or Tuesday, early in the morning, Friday evening we would do the whole country. So, we would test the material with the postgraduates. And then we see how it's flowing. Correct anything any science that is wrong, and then on Friday teach the whole country. So, we started by having the Zoom link which has which was basic which would get up 100 people. And then we'd lock people out. Then we went to 250 locked people out we went to 500 we were locking people out we had to pay for the 1000 and we still were locking people out. And so, it became a thing. Yeah, for like 12, 14 weeks every Friday. We had discussions would have a lecture presented the same way. Nurse... a doctor the the science nurse to the practice. And now we started recruiting people to help because now it was too much for me. So now I started getting the neonatal…requirements. We had three so they would alternate so we don't get too tired. The postgraduates also they alternate so we don't get too tired. So, Friday we had something going on those 14 weeks I don't think I slept. At the end of it one every hour of lack of sleep, I think and we were able now to do teach everything the whole content or the priority areas.” (FI 29)* |
| **7J** | When influential outside stakeholders are actively engaged in network processes (context), then they are more likely to be committed to the network (outcome) because they can be helped to understand the collective vision of the network (mechanism) |
|  | *See CMOCs 16B and 16E* |
| **7K** | When influential outside stakeholders (often specific individuals within an organisation) are committed to the network and actively engaged in participating (context), then it may be easier for the network to achieve its aims (outcome) because network members feel empowered (mechanism) |
|  | *“If I'm honest, I don't see that sort of commitment and things in big organisations, but I see it in the people who work for it. So, you know, the big organisation like USAID may, I mean, it’s sort of a nebulous, big, as far as most people consider money providing donor. But within it, there are some very highly motivated people. And I think it’s those people that are attracted to what NEST is trying to do. And those are the people who can drive their own donor units. So yeah, I mean, some of the big donors can just appear a bit tired, can’t they? But that’s, that’s the overall image, I think you need to look at the individuals who work in it. And they differ in different countries.” (FI 01)*  *“We have got our division of Paediatrics called a Child Paediatric and Child Health, so we haven't involve them a 100%. So, they're still especially on QI. So, I would loved to have them. That's one. And then when you would have loved to use their officers, NEST what they did is they asked people to apply, can you apply to be a quality improvement facilitator. So, we have got quality improvement facilitators from different places, who may not necessarily be government, they are in other counties, but they may not necessarily be staff, who will ensure sustainability. On my part, I remember I had three officers, but later on, they became busy and I became the only one supporting. Again, as a result of the issue of you have you had to apply. Right? So that is, so so recent. So, I do I would want so so that that was a challenge.” (FI 27)* |

### Engaged and motivated network members

| **8A** | When network leadership provides opportunities for network members to be supported, recognised, and learn, through training, equipment provision and support, and facility renovations (context), this creates engaged and motivated network members (outcome) because they derive direct benefits and feel like they belong to the network (mechanism) |
| --- | --- |
|  | *“And I can say that one of the things that I've learned because we've had quite a number of programmes are running across the health sector with the NEST is a very unique programme. Why could why should I say it's unique with NEST actually, it goes back to the users or the clients or to appoint the facility. And it narrows down not just at the facility management, because some programmes will work with the managers, then you're just given these what you're supposed to do and all that. But with NEST it's narrowed down to the people that are hands on, for our case, the devices, the people that are really with the devices on a day-to-day basis. And with that, we feel we are part of the programme because you're able to contact a technical person in the programme that is readily available to assist in terms of selling as well as in terms of assisting, even troubleshooting in terms of diagnosis, in terms of repairing, and even at some point they can even be able to come and physically support. Then in terms of training again, the kinds of training that NEST has been offering is quite different from so many trainings that have been able to attend because the kind of training that NEST is offering it's not just a training to understand the equipment but they're gauging ‘Do you have the necessary skills to handle this piece of device?’ I speak that because I'm also a trainer with them. So, I'm also a trainer with them. And this was really assisted us to feel to be part of NEST. And also, we're able to give those monthly reports to them. So, every time we share the reports, they're able to gauge the weakest points we are having. And from there they are now to support.” (FI 08)*  *“I do feel do feel because given the technology and the technology and the training I've gone through now, but with some of the equipment, we never had a good idea about. Actually, my interests I've really been on the CPAP...... This is a machine, I never learned it in other training. I got it the first time when I was introduced to it by NEST through that I'm now in a position now even to train others on how to use and how to how to operate, how to handle, how to repair in the event of a breakdown. So, I really feel to be part and parcel of NEST because of this. Being involved and being given a free a free will to handle the the equipment in the under NEST program. So, I feel really part and parcel of the programme.” (FI 13)* |
| **8B** | When network members’ personal identity strongly aligns with a network’s identity and culture (context), then this will result in engaged and motivated network members (outcome) because they feel they belong (mechanism) |
|  | *“Actually, you can feel it, I think that one of the challenges we have would be, first of all, let's talk about the passion. You know, passion is there, you find people come report to us very early, they will leave very late, you know. We have that shortage by and large our NBU you know sometimes it goes up to 65, 70 neonates but then that's on some shifts actually can be against three nurses. But then it becomes really difficult you know, but you will see them work. And, you know, sometimes you sit down and say, ‘God, this is just great.’ If you don't have that push that commitment, believe me, you will not you will give up and they're so passionate and they feel bad. You say doctor, we've lost this baby. We did our best, but it is* *very sad. You know, you can read it. Yeah. So that passion is there. The passion is there. We just need now at the county level, to motivate these people. Motivating means, for example, increase the number of workforce. Yes, we need a fairly good ratio between the healthcare workers and the patients to have that one on one attention. Yes, as I say, you can see them you can read a bit of frustration because if you're in a room where you have 25 neonates and like some of the rooms, the acute rooms, and you will find three or four babies are on CPAP a, you know, CPAP really needs close monitoring, like initially, you know, quarter hourly and all that. So, you have like three or four on a CPAP. There are others on oxygen, they need the SPO monitoring that these ones who need fluids adjustment, they need that and that. So, they become a bit overwhelmed. So, if it were not for the passion, they would just give up.” (FI 04)*  *“I think there's just something special about people who work in maternal and newborn care. Most of them, it's it's from the heart, sometimes somebody will tell you, I was just, maybe they might just be a nurse. They're not as specialists, they're not a neonatal nurse or whatever. But the moment they get into that space, somehow, it's just some positive energy that comes with it, yeah I think. I think I have not seen somebody who didn't like working with newborns. I haven't seen one. You never know, there could be. I haven't met one. Everybody it was either, ‘Oh, I was so scared.’ ‘Or I didn't know I don't think I was I'm passionate about kids.’ But when I got there or my all these changes.” (FI 14)* |
| **8C** | When network members actively participate in network change practices that align with the professional values they live by (context), this creates engaged and motivated network members (outcome) because it helps them to fulfil their moral obligation or vocational calling (mechanism) |
|  | *“Because when they come, they're telling you this week this month we are doing mentorship. Next month, we are doing quality assurance, so whatever you are giving you up to the standards, right, right, right. So, I'm really happy for this programme, it has made us to be up to the task of just not doing things, you are doing things knowing that at the end of this month, I'm being monitored, what have I done right? What have I done wrong? You see and it was good to have … know when you go to meetings, and you see that* *glucose monitoring Bungoma you at number 10 here, but Kakamega is number one, you want to see I'm going to Kakamega that the nurse there but that you're doing right so that I can reach you on NEST as opened up our minds as a programme.” (FI 11)*  *“A good part of that because when you have something wishes streamlined has a focus as a progress. It's easier. And you see, you are part of that. NBUs it's NBUs, this machine is not working. You see in you you are part and parcel of that. And when everything goes well, you know that everything is okay, today we are okay. You feel good. Because you know that newborn that premature baby will be there maybe for one week, two weeks, and if they are, okay, now with that. You see now, in your profession, you are something you are proud proud of.” (FI 24)* |
| **8D** | When a network can show its members that it can affect some change (context), members are more likely to continue their engagement with it (outcome) because they can see its value and a sense of satisfaction with the positive changes (mechanism) |
|  | *“Because when you see that when they come here, they’re just saying that they have that attitude, they change when they meant for nurses how are most aged, you see that was the perspective. But now you see now the young nurses are coming out. And if this if these babies can, if someone nurse these babies, tiny babies and go home I can also manage so people are not isolated in the NBU. Now if you talk to people they are now willing to come to work in the NBU, unlike the past, when they could only see the aged who are working in NBU. So, it has really brought up a very big change. And when you see when people from periphery come and see how you’re handling these machines, how you’re handling patients, they feel the change.” (FI 11)*  *“But once I think from the onset of the programme might have caused some of these changes is one the attitude. Because before, even from our end, I think we need to have that really positive attitude towards the newborn unit. Yeah, because you're categorising it just as the larger the maternal unit. Yeah. But today, I think of that we feel like that's preterm is equally a human being is equally important and needs to live. And with that, now, it changes our attitude. And we feel like we really need to take care of these devices, the newborn unit and have them functional.” (FI 08)* |
| **8E** | If a network can leverage the affective or normative professional commitment of its members to work towards achieving network goals (context), then they can become more engaged with the network (outcome) because of a sense of shared purpose (mechanism) |
|  | *No supporting data* |
| **8F** | When engaged and motivated network members are provided with the resources and opportunity to act (context), then they will attempt to enact changes in practices (outcome) because they feel empowered to change practice or work towards solving the problem (mechanism) |
|  | *“And also showing us whether we’re using our equipment because they monitor how machines are used. So, when we talk about like we have unmet needs for some things, like getting data to support that, this machine has this number of hours of use, it’s dramatic because before like the basic things, some of them you bring them in yourself, like buy your own pulse oximeter is not necessarily for newborns to finding this equipment or CPAP. There was a form of CPAP that we’d used but we’d use a bottle and put like measure the for the amount of the water level that you wanted to put in there. We couldn’t really calculate your FI02. So to find all these technologies that can take the kind of use you’re putting them through, it was a game changer, it really was. CPAP has given us like another layer of management before calling, like some of the Level 6 referral hospitals to give the babies a chance. Like with the non-invasive form of ventilation, which is what the world is moving towards, like if you can avoid invasive ventilation, that is what is behind it. So, to have that, as a resource, I think has been a really... And even other hospitals are hearing about some of our referrals are sent so that they say they have come for CPAP. So, it’s I think it has changed a lot of things, you’re doing more responsive medicine from data, you have like things you have collected physiologic data you’re collecting from the patient, so you can tailor make your management to that patient. And you’re wondering how you were doing it before, without some... like even another example, a recent one, which found me there is just a Bilirubinometer. Because there are times our lab does not cannot process those samples, either the machine has broken down or we don’t* *have the reagents. So, you keep babies for days longer than they should have stayed because you’re trying to be careful. And they get phototherapy they don’t need, then the numbers go up because you’re still admitting. And now now that we can measure it at the bedside, you can make a medical decision for a baby immediately that is sound and supported by something. So that has also really helped a lot.” (FI 03)*  *“See, we were able to able to renovate the newborn unit and repainting. We’re proud of that. Okay, it is been NEST who did for us. And you think that the repainting and the renovations…when you’re working in a clean environment in a beautiful place It motivates you to work... in a clean environment, beautiful place it motivates you to work.” (FI 26)* |
| **8G** | When network members are committed to the collective vision, that is strongly associated with their professional identity, are faced with issues that are beyond their control (context), they will not be deterred from continuing to engage with the network (outcome) because they believe what they are doing is worthwhile and feel accountable to other network members (mechanism) |
|  | *“So, I think it's what I said that at the hospital level, because of, you know, this teamwork that had started, you know, way before NEST came in at the hospital level, there was less of disruptions. It's more at a more senior much more higher administrative logistics issue to get things done. But having said that, there's the, I would say, the boots on the ground didn't change in a big way. So even when we had a lot of administrative changes, the guys who were responsible for making things run, remained relatively the same. So, you the learning curve was not issue because these people knew what needed to be done. And had, you know, where part of the original NEST plans, were consistently updated through them meetings, both national and international meeting. So, they were pretty familiar with the implementation processes. So, I would say it’s the administrative logistic hiccups that would end up slowing things down, rather than the this team, this middle level team that knew what they needed to do that changed. And I think that played a very huge role to make to still get NEST to where it is. And there was a lot of dedication and commitment from that team. That you know you cannot take away that I I it's a pretty slim team, looking at the amount of work that needed to be done. And they did a fantastic job.” (FI 32)* |
| **8H** | When network members are transferred to facilities out of the network or out of the network unit (context), this leaves a gap in trained, engaged, and motivated network members (outcome) because their skills and importance are lost (mechanism) |
|  | *“The only challenge now we've been having is the fact that those who have been trained again, sometimes because of political reasons, they're moved to other units. So, you find that it's maybe have an issue, maybe it's even a personnel issue. So instead of me looking at it like you you've trained this nurse in the newborn unit which knows everything about newborn care, but since I have an issue with you, I take you to maternity or I take you to surgical units and bring in a new nurse who's not trained. So, it becomes like, lost lost resource. This person is highly knowledgeable, they know what to do. But the new one is coming in again, you have to start again afresh.” (FI 10)*  *“One of our biggest problems has been staff rotations. We make every effort to make sure we train you, one month later. Yeah. You're not there. Yeah. So, we the that has not gone well. We've struggled and I don't know whether it's us or it's the system, right? Or what we should do about it? How do we keep people in the unit? So, we've had that problem in all the countries. Okay, all the countries we're struggling with keeping people in the unit. Right, right. Yeah. No matter how much you talk with management, management is still moving people. Even when you … they're still moving people, so that has not gone well. I don't know. Actually, we don't know how to do that. So, we've tried to come up with ways to solve it with simple orientation programs. Still lobbying with the management, please stop moving people. We've we've now realised that if we get people specialised, people will not be moved. Yeah. So, if we can have like neonatal nurse in training, right? neonatologist...Mostly nurses are the ones who are moved. So, if you can have many of these, whoever wants if they can go and do the course. Yeah. And we didn't have any funding for that. Right. So, we are not able to take people for those courses. But their while we … up there who are willing to, so we are encouraging people if you can do neonatal nursing go and do, this is where you can go and also participating in creating curriculums for different programmes. So, we've done that separately. I've been involved with so many like last week, all this in an effort to keep it going in the unit as much as possible and not give them the skill but keep them right right there where we need them.” (FI 29)* |
| **8I** | When networks engage with non-network stakeholders in network activities (e.g. quality improvement) and deliberately seek out feedback and advice (context), this can improve network activity implementation (outcome) because they provide a broader understanding of relevant issues (mechanism) |
|  | *“For the QI facilitators, mostly clinical and biomed are from NEST sites and even HRIOs. The gap is the ones that are from non-NEST sites, its governance. So, you find these people from governance they are not necessarily clinicians or biomed, it can be somebody in an NGO. We have one, I think it is only one who is a clinician. The other one is working in an NGO. So, yes, what they tell us is it has impacted them as individuals. Especially the clinicians in terms of what they do at their facilities. But you can’t really measure in terms of has the process influenced you as an individual and what you are doing at wherever you are working. But I think the major is, we have learnt a lot, the training, and then we give them certificates. So, like last year if we did two rounds of QI. We gave them certificates mentioning the rounds of QI. Then this year, of course now with the transition and trying to pick the new model, of course we will now remove all those that are from non-NEST and we give them certificates for one round that they have done. So, I think they also appreciate that bit that ‘I was in NEST.’ And then they also mention a lot of impact in terms of the programme. The one thing, one good thing with people from non-NEST, they give feedback without bias. ‘We went to Mama Lucy last QI and this is what we were told and this is not working, you should do this.’ So unlike people who are from NEST side, they might only give the positive, because you know they are also being supported. But people form non-NEST are not attached to a facility, so it’s no biasness in terms of the debrief. So, we always have a debrief when, maybe after two weeks virtually just to share their thoughts including the QI. So, for the county coordinators, they had an observation form and you will also notice the gap of data. They were saying “these people were not able to access data” So, you see it is a problem out there. So, we usually have a debrief and they say, most of the feedback is impact of the programmes to the facilities rather than to the individuals but as individuals they are usually happy like even through the WhatsApp chats and all that you could realise they are also impacted especially because of the trainings and the certificates.” (FI 16)*  However, because they are not part of the network, they may be less engaged.  *“We had on my part, I would say that we have division of we have got our division of Paediatrics called a Child Paediatric and Child Health. So, we haven't involve them a 100%. So, they're still, especially on QI. So, I would loved to have them. That's one. And then when you would have loved to use their officers NEST, what they did is they asked people to apply, can you apply to be a quality improvement facilitator, so we have got quality improvement facilitators from different places, who may not necessarily be government, they are in other counties, but they may not necessarily be staff, who will ensure sustainability. On my part, I remember I had three officers, but later on, they became busy and I became the only one supporting again, as a result of the issue of, you have you had to apply.” (FI 27)* |

### Empowered and confident network members

| **10A** | When a network acknowledges the capabilities of network members by selecting them to be network trainers and mentors (context), they become confident and empowered in their skills (outcome) because they develop the capacity to teach them (mechanism) |
| --- | --- |
|  | *“I think that was quite a bit of a rigorous process but it took time to get to that point. I remember it since the very first training that we all went. The initial training, the refresher training on the devices is out of that. That NEST there are facilitators who are able to point out specific people, according to how they performed in the training and they recommended them to undergo what we call training of trainers, TOT training, of which I attended the TOT training. I was able to attend that training in Nairobi for one week and out of that training again because, you know, you have the pre-test and post-test whereby they’re able to gauge your performance and also during the training you do the presentations, they’re able to gauge how you perform out of that. Again, I was picked up to undergo another training that is called Generic Instructor Course, the GIC, of which I was also able to do the training. I think I performed well because out of that, again, I was picked now as an instructor candidate. Yeah. So, I went through the instructor candidate, of which, that team of instructors, they were able to assess us, they have to now recommend if you really fit or rather if you qualify to get trainer with them. So, it was among those that were picked to be a trainer with them. So out of that now, we are able now to start support in terms of training. So, when they organise any technical trainings, we are called upon out to support the NEST and train our biomeds and all that. And to me, that is a benefit because once I come back home, I’m able to train my biomeds here, I’m able to train the students that are coming on attachment because having now that knowledge, having gone through the training, what it entails, and all that I’m able to conduct all that.” (FI 08)*  *“I’m very very happy because it builds people confidence. For some people, I know one of the nurses that we talk about it with him. He was so quiet teacher. I was saying, Edith if it's not this now taking so much of my time because we had to rehearse the thing four times for him to present something during the webinar. Today, that man is our best trainer. He is so good so and also others who you could feel now, you know, for my students. It's something because they're I'm going to train them. But now when it comes to the other people are spending now this is now yeah. But I learned now to have the patience. Those people, the ones especially the ones who are not very good trainers, today, they're good trainers, because I will tell them, you have not read the reference that's why you don't have confidence. And they go read a lot until they come and even talk things which I tell them now now you are really an expert, you have mastered the subject. Yeah, so that's how we now generated a group of newborn unit people who were very good mentors, because they knew their subject, they were trainers, they have read the references. They had the confidence. Yeah, so right now the people, if you go to these facilities, they talk proudly about how they used to train, how they learned a lot, but they're learned a lot, because they also went a lot to read right, to prepare for training. So, in each facility, we had trainers which facilitate, we had a nurse, a paediatrician who were trained as trainers through the training for trainers. And then now we'd have very many rehearsals with them. So, they're quite polished.” (FI 30)* |
| **10B** | When network members receive support (e.g. training, mentorship) to help them improve and expand their skill set (context), they become more confident in their previously learned and newly acquired skills (outcome) because they develop the capacity to perform them (mechanism) |
|  | *“Also, the use of CPAP, most of this stuff, when we started the programme, most of the clinical staff have been lacking the confidence let me say that to use CPAP. Because to them, it it has been like a complex device to use given the mortality around the use of device, of course, and it sort of brought about some negative attitude around using it. So, with time with the trainings with the mentorship, this builds so much on their capacity and their skills and and boosted their confidence. And speaking of hospital like Kiambu they've been doing well when it comes to use of CPAP and and then in one or two other facilities.” (FI 20)*  *“They're more confidence and not only using the machine even in resuscitation, they are more confident let me say in everything they do unlike before, and I believe the training is has brought has brought all this.” (FI 23)* |
| **10C** | When network members receive support (e.g. training, mentorship) to help them improve and expand their skill set (context), they feel empowered in their role and motivated to proactively apply their skills beyond the network (outcome) because they have greater confidence in their abilities (mechanism) |
|  | *“For our case in terms of biomedical engineers, you feel there's so much that you've learnt out of the NEST programme, you in terms of support, in terms of tools, in terms of how we work that you've been able to carry that to these other departments. Yes, yes, yes. Because today you go to laboratory, we have a file there for devices, the same case, we are having in the the newborn, you have a file there. So that once the biomed visits, anything they do, they're able to record and file there. So today, we are able because you've seen the good things that have come out of this. So, we've been able just to try and adapt the same to these other areas. And also, by assigning the biomedical officers in other areas, today, we are able to try and push for trainings, so that if somebody is in theatre, they can be able to become the best in theatre, attend as many trainings as possible under the theatre category, so that they* *can come back and be the best in that unit. So, we've been able to learn a lot out of these NEST programme.” (FI 08)*  *“So, people never knew what the biomed does, yeah, but NEST has brought the biomed out. And now they are doing what they're supposed to be doing. Right, because I love what one of them said in JOOTRH. The biomed were taught about the device. They were not just taught about clinical use, they were taught like opening the device, right? Like an engineer, like look into them. This is what if it breaks down this is the spare part to look for. So, they know how to repair those machines without NEST. They know they can ask for help. They can do videos, they can do take pictures, and they can be somebody can advise them because we have our biomed experts. So, the biomed role has been improved. Biomeds never used to go to newborn units. Now they go. And I like what one of them said that they know if this machine is not working a baby could die. So, they make it a priority to pass through the newborn unit and make sure all the devices are working the way they should. Then another biomed from JOOTRH said we took these concepts, we learn about newborn technology. And we are doing the same thing in ICU, in medicine. So, the other departments in the hospital are benefiting from the things because the biomed takes care of the whole hospital, right? There are not many, right? So, one or two are located in the newborn unit but they also take care of. So, what we are learning here, they are taking to, they have a path. So that for me, it reassures me that we won't have a graveyard because they will ask questions,* *they will try and look for a solution.” (FI 29)* |
| **10D** | When a network brings in previously neglected cadres/positions into the network and capacitates them to do their role (context), they feel empowered to enact their role in the network (outcome) because they feel their skills and abilities are valued by the network (mechanism) |
|  | *“I think the programme has has trained the biomeds in terms of how to take care of the equipment, the bundle of equipment to make sure that they serve the purpose that has been set out. So, it has created that responsibility on the biomed, so that he doesn't say I'll wait for me to be called. Now, they're able to do PPM, planed preventive maintenance for the unit, they have also been able to train the users on how to use the equipment. And even though the users are more knowledgeable, when they see things are not working, they're able to call up and the biomeds are readily available to offer the service. So, I think it is a it is a lesson that even the other service delivery points can utilise the policymakers, can utilise in terms of knowing that the biomed as important as the people who are offering the end care to the patient.” (FI 12)*  *“Even the other teams because you will find some facilities do have sessions whereby it's quote unquote, some competition interdependent interdepartmental. So, if like this month maybe the NBU won, last time it was the biomeds, or different areas, maybe cleanliness, maybe workspace organisation, maybe service delivery. So especially in the service delivery part. If a problem will be reported on a NEST device that is installed, and that problem the biomeds are able to address it within the quickest time possible, nobody will fail to appreciate them. They'll see these guys are really working, spares have been ordered, they are able to take it up, they're able to escalate it to the NEST team in Nairobi, the NEST team was able to organise proper … to reach us, they do this work the machines back working, they feel it nice when they get appreciated because of the work done.” (FI 15)* |
| **10E** | When network members (e.g. nurses) are given the freedom to use their clinical skills and role (context), they can take action to address problems/provide care without waiting for a more highly trained network member (e.g. doctor) (outcome) because they feel permitted to do so (mechanism) |
|  | *“What we've empowered ourselves with, like now the nursing team, you look at it's very different from how, you know, we started, they are empowered, they are confident, they're able to to to link up with the consultants, the medical officers in a very good way, focusing on quality of services. So that I feel especially the nursing team, we feel proud when we walk down there in the NBU unit, we feel proud of what we are able to do. Yeah, initially, it was like the baby has complicated, call the consultant, call the medical officer, but nowadays, yes, we are calling that consultant or the medical officer, but it's something we are already doing. So that we are able to stabilise the patient, the doctor is coming in and finding us doing something. Yeah, so I feel the NEST team has been good. It has involved all of us. It has not many times you may find a programme just focusing on the doctor only. So yes, we are there supporting them but we don't know exactly what to do, we are waiting to be instructed by so um, so. But here we are put together that class was one. They didn't isolate doctors, consultants in a different corner. We had one class and whatever we were able to be empowered with knowledge, skills, even the machines knowing how to operate them.” (FI 09)*  *“The other change I've seen is that the nurses are able to embrace the new technology...you find that a preterm has come, you're not on duty do you want to know how to take the...to do this the preterm the syringe pump, people really have embraced that technology because it is to use a syringe pump or that Bilidx those. You find a nurse has taken the the … using the Bilidx so you find that the nurse had already started the baby on phototherapy. Previously, you have to had to it for the lab but sometimes the reagents there are no reagents.” (FI 26)* |
| **10F** | When providers in the network are well trained, mentored, or coached (context), this positively changes their attitudes around performing the skills/providing care (outcome) because they feel empowered and confident (mechanism) |
|  | *“Before NEST come in if you can, just starting up from that background, where we are before NEST came in even here as a department, we considered a newborn unit as the larger maternity unit. Yeah, we were not so specific and so much good not giving it so much attention as the newborn neonates but to because we assigned biomedical engineers to every key department within the facility. And we had just assigned a biomedical engineer in maternity unit. And we're not so much concerned or rather, we're not so much on point in the newborn unit. And this now made us just to do the general works, whatever we can do in the labour ward, whatever we can do in the post-natal. That's what we actually also did in the newborn unit. But when NEST now came in, we realised we need to give more attention to the newborn units. And with the trainings that followed, the subsequent trainings, and all of that that's it now engagement, the newborn unit now became even more closer and even more passionate with the newborn units.” (FI 08)*  *“Let's say we feel proud to be part of NEST, we feel we refer ourselves as the NEST hospitals. And because it came and changed how we do things. It's changed how we you know, we perceive things, it's changed our outcomes. So, we are very proud to be part of it. I must say, it's like, yeah, it's like a movement sort of. So first of all, you are always motivated to do the best you can, you're motivated to make a difference for that child. And, you know, you stop doing things just the way you were doing them before because now you know better. So, I think it's more than just adding the group of people but from within, they found it changed in you. And then when you come together as a group, I think it's more of a movement. We want to make a difference for the children. So, I hope that can continue.” (FI 23)* |

### Purposeful relationships, linkages, and partnerships

| **4A** | When network members believe in the network’s collective vision (context), then this can help ensure that network members have purposeful and co-operative working relationships (outcome) because they are more willing to work with and value working with people whom they identify as being likeminded (mechanism) |
| --- | --- |
|  | *No supporting data* |
| **4B** | When network members are open to and able to invest time in developing relationships and linkages through the network (context),then this can help ensure that network members have purposeful and co-operative working relationships (outcome) because they have a better understanding of each other (mechanism) |
|  | *“Now, because, again, I said, what everybody wants to see is that the statistics are improving, you know, perform quality performance within health facilities, we decided our first mission was actually first to coordinate, introduce ourselves with health to the health facilities, and then find out what the issues are. And then we can work with individual health facilities to see how to rectify this to strengthen where things were working well and then overcome barriers where, you know, there were barriers.” (FI 28)*  *“The other thing that we discovered was that was not enough. So, we had to do mentorship. So, after the training, and then the assessment, then two days mentorship. I found that to be better. So, in Nyeri, we did two days. And I did the mentorship myself. And it was interesting I would say because I had to make first of all, I had to make friends with the nurses in the newborn unit. Because not all of them were able to attend the training. So, kind of like do a training for them. So that they don't say oh, that CPAP belongs to the guys went for the training. So, the two days were very useful to …now everybody to oh, this is a CPAP machine, this is what it looks like, this is how it works. And I was there with the biomed. So, it made it easier. And now the people who had been trained also also helped with the ... So that's where I learned that if you want anything to work, it helps if people trust you. It helps when people see that you're one of them. So, I would wear scrubs and just be with them, work with them. And then bring in the issue and for that they didn't know in fact they have been doing wrong, suctioning procedures people have been doing wrong. So, it was time to refine those. We discovered some students who are in that unit also had never seen these things before so also teaching them. And so, in the end, I had to make friends with everyone, and we were able to identify some key, my opinion, people who would carry forth that training.” (FI 29)* |
| **4C** | If network members have strong pre-existing relationships (context), then this helps ensure that network members have purposeful and co-operative working relationships (outcome) because they are already familiar with each other (mechanism) |
|  | *“There is already a network here formed by KEPRECON and KPA, you must have heard about the Clinical Information Network. So, most of those hospitals were already part of you know a community of practice, so to speak. So, they already had that comradery going on. And NEST was more of an addition complementary into what they do. So, for that kind of network, I would see them feeling the value of being part of that network and the strong data that coming out. For others, again, it was more of a mixed results. And they would see just how do I improve things in my own facility but not necessarily as a network, the were efforts made though to try and bring these hospitals together.” (FI 31)*  *“And I think there was also that it was much easier because a lot of the people who ended up within NEST, quite a number of them, were familiar to one another. So, they were part of the broader CIN. So, these are paediatricians, a lot of them either knew each other through CIN or through previous networks or having trained together. And so I wouldn't say there were, the challenges of setting up the network were much easier, in terms of a network of professionals and and people involved in implementing NEST. I think was easier here than in other countries, because they also have some sort of some sort of existing platform.” (FI 32)* |
| **4D** | When network members share a professional identity (context), this supports forming purposeful and co-operative working relationships (outcome) because they share common socialised perspectives (mechanism) |
|  | *No supporting data* |
| **4E** | If there are artefacts in place that outline the roles and responsibilities of network members (context), then this can help ensure that network members have purposeful and co-operative working relationships (outcome) because there is a common understanding among network members about what they are expected to do (mechanism) |
|  | *“So, there is that clarification there is that clarity of each cadres has role in how they should go about things but also help them to plan on may be making arrangements of which devices need to go for maintenance. So that they are kept free for that kind of work to be done. Yeah, so that is those are some meaningful strides that I want to say have happened, again, coming back to the biomedical department.” (FI 15)*  *“Then I think after that, at some point, we signed a memorandum of understanding with the facilities, all these facilities, because it's very key in ensuring that it's just a binding document, because you see that NEST programme is also a programme that is on a research basis. Yeah. So, involving them and all that there has to be a binding document for this.” (FI 20)* |
| **4F** | When a common baseline understanding is created among network members of what is expected of them (context), this helps to develop purposeful relationships (outcome) because the network members have the same level of understanding about each other and their roles and responsibilities (mechanism) |
|  | *No supporting data* |
| **4G** | When a network creates opportunities for relationship building between network members within and across facilities (context), this may help in the creation of purposeful and co-operative working relationships and improve existing working relationships (outcome) because they get to know each other’s personality, skills, ways of working, and motivations better (mechanism) |
|  | *“I would say that that relationship wasn't there initially. In fact, whenever a machine would fail to would develop a problem. Even the nursing team would be like, maybe let it just stay here because if you call them it will just be taken away. So, the biomed team was in another world and the clinical team in other worlds. Now, after this training, the clinical team realised they need the biomedical team to have their babies taken care of very well. So now, like in Kakamega County General Hospital, we have two biomedical engineers stationed in the newborn unit, attached to it. Yes, newborn unit is covered 24/7 by my biomedical engineer, they come in the morning, check the incubators, adjust those fluids and do everything, then in the evening, they will again come and do that. They will be called upon wherever there is any problem. And all that. So, I will say the relationship is quite good. At the moment, they appreciate each other, each other's role. They both some of them will even join my ward round. Yes. And especially if I'm teaching things like phototherapy, and all that they would be in. And I would ask them, yeah, well, what's your take on this and that, so they have just realised all of us have realised our role is to ensure that baby that neonate both receive the best of care. Yeah, so it wasn't there. That relationship was not there before it was bad. I will say, Yeah, but now people appreciate each other strength and the role. Yeah, in the overall outcome of our neonates.” (FI 04)*  *“I think NEST has played a very big role particularly to harmonise our working relationship between the biomeds and the staff. Because whenever they have taken us to be part and parcel of the unit and through that, they have gone further, even to to empower us. So that you have the capacity to support the newborn by giving us the training through that alone, the user or the sub in the newborn unit at the moment, they have confidence in our working ability, as a result of being being quick to be maintained. So, they have confidence we have created confidence in them towards us. Yes, because initially that confidence was not there. But through the empowering of NEST at least, they're able to allow us to get to work on their on the newborn equipment without without having any, any doubt of our ability, maybe to handle them. Yes, and NEST has given us that free role. We have given us free role even to touch on the equipment to see the event maybe I'll start that is when we can conduct today, the main NEST engineer who assistance. So, they are really created a good working harmony between us biomedical engineers, and the user and the newborn unit. Yes. So, we now have got the working relationship and given that has also advanced our frequent interaction with them. Because through our programme, we are each and every day each and every day, there is no day I can't miss going to the newborn unit. Because weekly maintenance I normally do because of the how frequently machines are used to monitor them on a daily basis. So, it does really strengthen our ties with the with the user and the newborn unit.” (FI 13)* |
| **4H** | When the network provides resources that supports the network members’ ways of working (context), this may help in the creation of purposeful and co-operative working relationships and improve existing working relationships across professions (outcome) because they are better able to collaborate and do their job (mechanism) |
|  | *“Actually, it change them greatly. Before before NEST came. We never used to have to have the phototherapy light, so that you make sure that your baby is about on jaundice, when it comes to procurement, sometimes it could take a lot of time. And again, also that pile part of maintenance, it could take a lot of time. But when when when NEST came, actually it improved our services. And because the equipment were very simple to use, you could also find we used to very we used to have very few concentrators, but when the NEST came and brought those bundles even the nurses who were who are in that department in the newborn unit, staying abreast of the technologies and also they became very active because the output was very very within was it was within a very short time. And they could they could become friendly, more friendly to maintenance biomeds, because when they have a breakdown, they call us. We could also network with the NEST personnel in case of any problem and they will be sort with they could be sorted within within a very short time. And that meant the whole thing to become at least enjoyable. And we could we could we could have a kind of good relationship with the nurses.” (FI 21)*  *“Initially before NEST you do it until the machine has broken down, then you're trying to fix it yourself. You're not able, then I'd say let me call the biomed. Sometimes they're not even available. But after NEST, we were supposed to have one of the biomeds like based in the newborn unit we have one, basically in newborn unit. So, because of that, is able to do the servicing of the machine, the ones which are programmed like it doesn't have to attend to the machine has broken down to service the machine. So, because of that we have many more breakdowns of the machine. And then even the ones that tend to break is always available to repair. And because of NEST, again, we're able to get those um those whatever they are call that whatever the parts, we need to repair the machine. So that helps. But the fact that he's available, I think it really has made it possible that our machines are able to function longer and more efficiently, because they're being serviced every other and then before we do that until they're broken down then we realise has never been serviced before. So, I think being this in the newborn unit, and always being available, it has really improved the shelf life of this machines, and us being able to use them. Because there's no point of having a machine if you're not using.” (FI 23)* |
| **4I** | When network leadership creates a psychological safe space that enables cross-learning between network members (context), this may help in the creation of purposeful and co-operative working relationships and improve existing working relationships (outcome) because network members are able to openly share their experiences (mechanism) |
|  | *“I think they they go out of their way to also make you feel like that like with the meetings they they regularly call us for where they I asked us to present or present, like discuss our points of view, and they bring different centres together, and you realise that what I'm grappling with in my centre somebody else in another part of the country is grappling with the same thing or they have approached it in a different way that has worked. So, I feel so, I feel like we are part of a group and just listening to what other hospitals are saying that they they can see it and they want to join NEST also. So, yeah, so I think we do not just because of the innovations, but also the meetings that make you try and think about some of the interventions and how to get better.” (FI 03)*  *“We both have meetings and those meetings are quite, they're quite nice in terms of meet your colleagues, because it's good to meet your colleagues have not seen them for a long time. But again, you tend to learn what they're doing well. Because you have oh, like I remember the last meeting we had some they say they don't have challenge with hypothermia. And I was like they're in Nyeri, Nyeri is very cold it's one of those parts of Kenya, which is very cold, the infant would have, Nyeri is cold. Then they don't have a challenge with hypothermia, as we are here and you're struggling with hypothermia, and you know, then we kept from asking them, so how you doing different and you're able to tell us what they're doing differently. See, when you meet and you exchange information and notes, we're able to learn from one another. So, I think it's actually good for us. Whenever we have these forums, we always learn from one another. And they make them quite regular, which is good.” (FI 23)* |
| **4J** | When network members have established purposeful relationships within and across network facilities (context), this helps to improve communication between network members (outcome) because they are familiar with each other (mechanism) |
|  | *“I think what NEST strengthened within the hospital was biomedical engineering. Like before, I forgot to mention them as well. But I feel since NEST, that interaction has been strengthened, because it was a very technical kind of interaction we used to have before, like when machines have completely broken down. But I feel that has been … since because we do a lot of they do pre-emptive maintenance. And it's now more of a discussion, not just us telling them it's broken down, but also them feeding back how we're using equipment and whether we are using it the right way.” (FI 03)*  *“It is very important, I will say, prior to NEST, Kakamega County and Kisumu County are just about 50 kilometres away. But trust me, I had never even visited that newborn unit. I didn't even know whatever was happening there. Currently, it's very easy, you know, you when you have a case, you can call your colleague say, we have this business or that, have you seen this before? How are you handling this scenario? How are you handling this, this, and that? So, it's very it's very good. It's and I'm even called by the nursing team, nurses from other hospitals, you know, they'll be like, ‘Oh, Doc, we have this scenario. We've done A-B-C-D and probably, we're not getting anywhere, what would be the best thing to do?’ When it comes to consumables the various things, you know, in the lab and all that I'm able to ask my colleagues ‘Anyway, do you have this in excess? Could you give me this for some time?,’ you know, and all that.” (FI 04)* |
| **4K** | When purposeful linkages and strong working relationships are established between network members (context), this improves network members ownership of the network (outcome) because they feel connected to each other and to the vision (mechanism) |
|  | *“We also have learned our lessons in terms of how to provide quality care within our contexts, comparing to other facilities, and also we also have had an opportunity to compare with not just with the facilities within the country, but within also the other participating countries, countries within the the NEST, the NEST programme. So, as our facility, we feel more confident, we feel more connected, even with the interventions that are ongoing with the NEST consortium.” (FI 12)*  *“Well, I think I can say yes, because I think what is very special or what is very unique about how the NEST implementation has been done is that there is always that constant contact to the people and it is not just like any other project where people just come in it's train and donate equipment and that's the end of it. We are in constant touch with these people. Currently, I think we've been able to do like two programme review meetings where we want to get everybody involved, hear from them, listen to what they have to say. They give us their thoughts on how they're implementing this. So, I think there is that there is that ownership that I think is is is unique for in the NEST programme.” (FI 14)* |
| **4L** | When purposeful linkages and strong working relationships are established between network members (context), this helps to build trust among network members (outcome) because network members have confidence in each other (mechanism) |
|  | *“So, after the training and then the assessment, then two days mentorship, I found that to be better. So, in Nyeri, we did two days. And I did the mentorship myself. And it was interesting I would say because I had to make first of all, I had to make friends with the nurses in the newborn unit. Because not all of them were able to attend the training. So, kind of like do a training for them. So that they don't say ‘oh, that CPAP belongs to the guys went for the training.’ So, the two days were very useful to … Now everybody to oh, this is a CPAP machine, this is what it looks like, this is how it works. And I was there with the biomed. So, it made it easier. And now the people who had been trained also also helped with the ... So that's where I learned that if you want anything to work, it helps if people trust you. It helps when people see that you're one of them. So, I would wear scrubs and just be with them, work with them. And then bring in the issue and for that they didn't know in fact they have been doing wrong, suctioning procedures people have been doing wrong. So, it was time to refine those. We discovered some students who are in that unit also had never seen these things before. So also teaching them. And so, in the end, I had to make friends with everyone.” (FI 29)*  Constant change in relationships makes the creation of trust difficult.  *“So, the honest truth is it has had I'm looking for a bet... so I don't want to say it's significant because, you know, NEST is still functional, still goes on, but it actually did have impacts on implementation. Mainly, because transitioning. So, each organisation had its roles and responsibilities and there was very little duplication across those organisations. So, then moving from one organisation to another creates like a vacuum, sort of impact stream of work. So, if say CPHD was responsible for looking after the biomedical side of things, then transitioning that to another organisation or figuring how that will work in the absence of CPHD, then that means a lot and the institutional arrangements, therefore, of how, say, colleagues in KEMRI, would reach out to CPHD to get devices for training, then that has now to go to another partner who has to understand why should we give you devices for training. How does that work? How do we loan them? How can we trust that you take care of them? Because the also at an institutional level, there is some amount of time that takes before you can you can establish like a working relationship, trust, and all that. And that's not just at the beginning, because we transitioned* *from CPHD, then, I think, had a gap where the team in Kenya tried to run the show, then it was quickly realised that there is a lot of logistics that CPHD would run in terms of coordinating trainings and couriering things to sites that needed an institutional base, so we then transition to another institution, which also took a bit of time.” (FI 32)* |
| **4M** | When purposeful linkages and strong working relationships are established between network members (context), this supports the creation of a psychological safe space (outcome) because network members feel they can speak freely without fear of negative consequences (mechanism) |
|  | *“Not officially, what we do for other hospitals is when we are doing our mortality audit, and it comes up that we get quite a few bad outcomes or poor outcomes from a particular hospital, we try and invite them for some of those mortality audits, not as not in a vindictive way, but to understand what they’re working with. And we’ve learnt a lot, we’ve learned that some centres, the staff are doing what they can, but they’re not equipped or trained. And when you create that safe environment, like they can be tell you some of the things they do like what they do for resuscitation, and then that becomes like a point of, okay, we probably need to reach out to them. So, when we have trainings, we make a point of trying to invite some of those centres to come in or to also tell them what they need to advocate for from the hospital administration, some lack things as basic as oxygen and he’s just sent in an empty van at the back. So yeah, it helps to contextualise some of those things. But going down, that’s like part of the plan, that we have like to actually go there and see what they have, instead of blaming them for poor outcomes to go and see what they have, what are they dealing with, and how we can help them at…So we’re trying to identify a few facilities that if they are agreeable with it, but through the county.” (FI 03)*  *“So, you know, where people sit around a table during a group work meeting, and people actually appreciate that they, they know, the hierarchical structures between cadres do not exist in all these other places. So, these these are a lot of teamwork.” (FI 32)* |
| **4N** | When purposeful linkages and strong working relationships are established between network members/organisers (context), this enables network members/organisers to provide support in enacting the collective vision (outcome) because they can develop a mutual understanding of the identified problem and potential solutions (mechanism) |
|  | *“Sometimes we don't have a problem or maybe managing a sick neonate, it's just a matter of making an inquiry with them. I've seen they've really assisted us in so many ways. I can give an example. There was a time, had a problem with CPAP machine. It is not that our CPAP machines were not working, they were working. But there was just a problem because babies are not big which on CPAP. As an in-charge, I really tried, I really tried to talk to my staff, to just to encourage them. So, what I did, I talked to Dolphine and Dolphine came on the ground, and you see when she came on the ground it started working, it started working. So, my assumption was that you see someone you know, someone from outside and someone from in it, you know, someone from outside can convince you much better than someone from there. It works. It really works so well. You know when you have like 15 babies on CPAP in a month, so that is a…Yeah, anytime you come in the morning, all the CPAPs are working and the bed are full. So, there's there's good interaction, there's good communication with the NEST group.” (FI 07)*  *“Can you tell your story and your journey and what do you think and they're they're they're very emotional about it because they said we've never seen anything like the approach that NEST has used. They they say this is the only programme that came over here and it was long term. It wasn't it didn't come in and out. And you know, it has a follow-up when they bring like equipment so they ensure that they they train enough on the equipment not just purchase and do that initial, you know, one day trading and then you know, leave people alone. They said equipment is put in place, you are trained, you are followed up you are given ways on how to monitor performance of the equipment and reporting, how to troubleshoot when there are issues. The NEST people are available to work with you on getting the equipment back to work. The clinical teams are not abandoned. Also he they got your phototherapy machines, your CPAPs there. Then we have shown you how to use them and then we disappear we don't we walk the journey with them. With the trainings with the training of trainers though GIC courses for the teams. And, you know, all those things including mentorship. So so so they really they literally feel like, you know, they shed tears at the imagination that NEST would actually be out of the they say this is this is the best story they've they've ever said they can tell about the programmes that we have encountered, because ours is not about the research, as you know, it's about real, you know, making a real change on the ground.” (FI 28)* |
| **4O** | When purposeful linkages and strong working relationships are established between network members (context), this helps them to be in agreement (outcome) because they can more easily develop a shared conceptualisation of the identified problem and potential solutions (mechanism) |
|  | *“So, it has really interlinked us. The various hospitals say that we feel like we are almost somewhere, yes. We can always consult and compare ourselves with them, learn from each other. So, knowledge flows quite freely. Yes and we always, we are concerned about the service provision in our neighbouring hospitals or counties and this apparently has been quite easy. If we have a baby who needs a specific like surgical intervention through our interactions here, I will be able to know that he's a paediatric surgeon in Kisumu, for example, so that I don't have to refer that patient to Moi Teaching and Referral Hospital, which is probably over 140 kilometres away, you know, when I have Kisumu that is 50 kilometres away. So, it has enabled us to assess the strength of the various hospitals and know how we can then tap into this strengths here and there.” (FI 04)* |
| **4P** | When different professions within the network (e.g. clinical and technical) develop strong working relationships (context), their attitudes toward the other profession changes (outcome) because they understand each other’s value (mechanism) |
|  | *“Now at first, we were used to see them like this is just a person to handle the machines after one all he knows is the machine but when NEST came up, and they send both of us on how to use the machine, you see. So, to give them it gives us power also to see the biomedical people as also as part of the medical team. Because without them our machines will be failing regularly. But when they came up and they even they have a CMEs on how this machine is working for the when it fails when it is faulty, how, what what is done. It has brought us it has been that relationship to really build the relationship.” (FI 11)*  *“My colleagues would be very much willing to go to see how the babies are doing there because of the improved improved equipment or the bundles which we have we are brought to us. So that is that kind of improvement over there is that kind of that kind of positive attitude towards towards visiting the place and like sometimes back when up when when we never used to have that positive. Because when I go there, I will just tell the attendant that ‘oh, the nurse there I'm going to work out your problem or which will take a lot of time.’ Because now, I'm now I'm trained and I'm able to to assist. The person in that department at least have developed a positive attitude towards going to help then, yes. At least that is what I've seen. Yes, that is what I've noted. Yes and also, the nurses in that department are very happy because they used to have a very big, big mortality rate. But when the bundles came, their mortality dropped. Actually, too, it is only left in labour ward. Yes. Whereby I remember there is a month when we never we used to when we are the zero-mortality rate, from from from newborn unit. Yes. So, when the working in that kind of condition, you become very happy.” (FI 21)* |
