# Supplementary material for: Understanding the initiation, formation, functioning, and performing of networks to change practices – Realist evaluation of a programme to improve newborn care in Kenya
Source: SSM Health Syst. 2025 Dec;5:100101. doi: 10.1016/j.ssmhs.2025.100101 (PMC12678620; doi:10.1016/j.ssmhs.2025.100101)
Supplement: Supplementary file 8 — Supplementary material [file mmc8.docx]

## Appendix H. Extended narrative results

#### Network processes

This first section of the programme theory explains the processes that networks undertake to initiate, form, function, perform, and sustain impact and change (see Processes in Figure 1 and Appendix 5). They are underpinned by the refined CMOCs in Tables 1-4. There is one piece of illustrative data for one CMOC in each network process and additional illustrative data supporting the CMOCs is available in Appendix 8.

**Identify a problem**

Building on the results from our Realist Review, the primary data confirmed *identifying a problem* as the initiation of a network. However, the primary data indicated that this is coupled with network initiators seeing an opportunity in the problem and believing that they have the capabilities and resources (or potential to obtain or reallocate resources) to do something about the identified problem. Smesler’s theory of collective behaviour (Appendix 4) remains relevant to this part of the programme theory and the feeling of a strain around a problem, but the primary data adds that seeing an opportunity in the problem and a belief in capabilities and resources needs to be present as well. Depending on the network, these drivers of network initiation can exist within the same or different people. In an externally initiated network, the ability to see that opportunities exist within their capabilities and resources may more likely come from someone external, who feels the strain of the problem less acutely but has a broader perspective on it. These external network initiators need to present the opportunity to potential network members in a way that is attractive for them to commit to the network (see Commitment).

However, in a network that is formed organically or from the bottom-up, individuals may feel the strain of the problem but also see opportunities to change within their abilities and resources. The sense of dissatisfaction and/or frustration about the problem and the ability to see an opportunity will ignite the network initiators to do something about the problem (i.e. forming a network). The ability to see an opportunity in the problem may be latent within potential network initiators and not activated until they have the means to do something about it, such as resources or external support. This is relevant because the composition of network members may be important for the network’s ability to form, function, and perform (CMOCs 1A – 1C).

Network initiators will then recruit other people, particularly potential leaders or champions, into the cause and collectively work to understand what they need to do to solve the *identified problem*. Depending on the network, this may be people who directly feel the strain or who see the magnitude of the problem. Network initiators engage with potential network members to support forming the network and start to generate their commitment to solving the problem through the network (CMOCs 1D – 1G).

Empowerment theory’s approach to community empowerment (Appendix 4) may reflect these additional dimensions to this part of the programme theory.^1^ When potential network members have the capabilities, desires (linked to the strain or understanding of the problem), and resources to engage in change this may lead to network formation. As a network, they can identify needs, develop approaches to tackle them, and take action to improve them, as empowered communities do.

In the case of NEST360, the problem is high preventable newborn morbidity and mortality, which is a well-recognised global problem and one of the SGDs (Target 3.2.2).^2^ NEST360 is an example of an externally initiated network; the initiators at a US-based university understood and saw the magnitude of the problem, but they also knew they had the capabilities and potential access to resources to do something about it. The healthcare workers, biomeds, and hospital administration that feel the strain and frustration of the problem, as in most hospitals in Kenya, lacked access to the medical devices and training to substantially act on the problem.

While networks may be formed in response to the *identification of a problem*, once in existence, well- functioning networks can be employed to identify additional problems and may continually reinvent themselves and stay relevant. Networks use different approaches to continue to identify smaller problems that contribute to the larger problem/collective vision. The *identification of problems* within a network may occur in clinical practice, through learning sessions, during mentoring and coaching visits, using QI tools, or through approaches that search out to identify barriers or challenges to uptake in care or service use (CMOCs 1H – 1J).

The NEST360 network puts in effort to continually *identify problems* and take action to improve them. This includes the QI dashboard and visits, audits, and mentorship visits. Transferring responsibility for these network activities from the programme and over to the national and country governments and hospital administrations will support sustaining impact and change.

| 1A | When potential network members feel a sense of dissatisfaction (misaligned with expectations or values) with an issue (e.g. in clinical care, service delivery organisation, or health system management) (context) they will have the energy, excitement, and motivation to do something about it (outcome) because of their frustration (mechanism) | No supporting data |
| --- | --- | --- |
| 1B | When potential network members feel a sense of dissatisfaction (misaligned with expectations or values) with an issue (e.g. in clinical care, service delivery organisation, or health system management) (context) they will get annoyed/angry/fed up (outcome) because of their frustration (mechanism) | New |
| 1C | In the formation of an externally initiated network, when future network members/leaders/initiators are helped to believe that improvement/change with an issue (e.g. newborn care, morbidity, and mortality) is achievable (context), they will have the energy, excitement, and motivation to do something about it (outcome) because they feel doing so is a worthwhile use of their time and energy (mechanism) | New |
| 1D | When a network is in its formation stage and potential network members reflect together on problems (context) they will recruit others to the cause (outcome) because there is a realisation that they can be part of the solution (mechanism) | Refined |
| 1E | When potential network members share and discuss their collective experiences, emotions, understanding, or perspectives for a commonly felt problem (context) they are better able to understand what needs to be done and find solutions (outcome) because they have more knowledge to drawn on (mechanism) | Confirmed |
| 1F | In a setting with change potential, when network initiators meet and select others who share and amplify their concerns about the identified problem (context) this helps form ties that support network formation (outcome) because the shared concerns about the identified problem are mutually reinforced (mechanism) | New |
| 1G | When the network initiators get input from potential network members on the magnitude and resources needed to begin solving the identified problem (context) this starts to generate commitment from potential network members (outcome) because the potential network members feel their perspective and experiences are respected and valued (mechanism) | New |
| 1H | When members in an established network feel it is safe to critically examine and reflect on existing practices together (context), they are able to identify new problems and potential solutions (outcome) because they feel enabled to challenge the status quo (mechanism) and/or feel in a psychological safe space to be able to challenge the status quo as a team (mechanism)  *“Even the other year our unit was get the cleanest and the best organised and it's amazing because one you go to the other units you find the doctor will just write and go away. But here there is a lot of teamwork. For all of us the cleaners, the nurses, the nutritionist, because actually one of the other things that is...being that teamwork has really helped us to identify the problem and be able to ask yourself, who should solve this problem? How can we solve as a team? So, like one of the problem, one time we discovered was our babies were not gaining weight and they were taking very long in the unit we did a a small study. And the next question was, who should we include in this? So, when we sat down with our team, we discovered we only have two nurses in the unit per shift. Sometime this shift has 75 babies. So, you've got imagine if there are 75, you may be having 20 or 15 that are critical in CPAP or… So, when we looked at it, we were like, No, we need more staff. So, then we asked ourselves, with... with a team, we have a WhatsApp group now for the newborn unit. So, in the WhatsApp, we asked ourselves, who do you think we can add in this group, and we decided we need a nutritionist. So, we work with the administration, and we are given a nutritionist, just for the newborn who has been very instrumental in helping mothers helping calculate feeds, observing babies who are not feeding, gaining weight well, which has also really helped us.” (FI 05)* | Refined |
| 1I | In an externally initiated network, when there is support and partnership from network initiators and organisers (e.g. through quality improvement and mentorship activities) (context) this can help to continue to identify problems that are within the network member’s capacity to change and determine appropriate solutions (outcome) because the network members feel in a psychological safe space to be able to challenge the status quo as a team (mechanism) | New |
| 1J | When the network enables members to regularly identify problems they can work together to address (context) this supports network functioning (outcome) because they can appreciate the value of the network (mechanism) | New |

Table 1: CMOCs for Identify a problem

**Collective vision**

Network members develop a *collective vision* through a process of collective sense making, identifying what they have in common, and sharing common perspectives. When network members have a similar professional or vocational calling, this can facilitate developing a *collective vision*. In a top-down or externally initiated network, the *collective vision* is more likely to come from the external network initiators and so the first three CMOCs (2A-2C) from our Realist Review around how to develop a *collective vision* may be less important, but still relevant. A network will introduce its vision to additional members, particularly when the network is formed from the top-down or initiated by external stakeholders; this new finding was informed by the primary data (CMOC 2D). Once potential network members are introduced to the *collective vision*, they may commit to the network because of shared experiences, pre-existing relationships, and a process of collective sense making. Leadership and resourcing the network play a role in eliciting support for the *collective vision*. This area of the programme theory was further elaborated with primary data and resulted in the formation of five new CMOCs (2D, 2F-2H, 2J) and the refinement of two CMOCs (2E, 2I). The *collective vision* can help link members as a network (CMOC 2K, refined) and the existence of MoUs can support commitment to the *collective vision* and network formation (CMOC 2L, refined). If there is no *collective vision* then the network may be less successful; however there were no primary data to support this claim from our Realist Review (CMOC 2M). Network members may also work to get broader awareness and support to the cause or vision from stakeholders outside of the network (new CMOCs 2N-2P).

The substantive theories from our Realist Review – Smesler’s Theory of Collective Behaviour^3 4^ and the Collective Identity Approach from New Social Movement Theory^5-7^ (Appendix 4) continue to be relevant with the primary data. Tuckman’s Small Group Development Theory’s first three stages (Appendix 4) have greater importance for the revised programme theory.^8^ In terms of forming, the network initiators put in effort to introduce the vision to potential network members and support their commitment to the vision and participation in the network. This is particularly needed when the network is externally initiated or supported. Storming may arise here if the network initiators do not introduce the *collective vision* with care; however, the primary data did not show any indication of storming, so this is speculation. The primary data further supports that norming helps to explain how network members progress from developing a *collective vision* to the vision becoming a norm of the network.

NEST360’s *collective vision* was developed by the external network initiators (NEST360 Global). However, because the vision focused on a global problem (preventable newborn morbidity and mortality) that is particularly acute in Kenya, it resonated with potential network members, which made them more likely to commit to the *collective vision*. Early on in network formation, the network initiators (NEST360 Global) and the network organisers (NEST360 Kenya team) put in effort to introduce the *collective vision* to potential network members (hospitals) to generate commitment to the vision. While the *collective vision* came from the network initiators (NEST360 Global), it was based on a shared understanding and perspective with potential network members of the challenges with small and sick newborn care. Pre-exiting relationships between potential network members in NBUs and network organisers (NEST360 Kenya team) helped to generate commitment to the *collective vision* because there was pre-existing trust. Creating opportunities for potential network members to be heard and discuss the *collective vision* helped them to feel ownership of the vision. The network hospital leaders, medical superintendents, have varying degrees of involvement in the network. However, their commitment to the *collective vision* was generated with engagement from network leaders and champions. When network leadership (NEST360 Global and NEST360 Kenya team) communicated and explained the network vision to potential network members, they focused on the problem that the network tries to solve and the network hospitals are facing. In the early stages of the network, NEST360 provided resources to hospitals to support renovations to NBUs; this also generated commitment to the *collective vision* because network members saw that the network was doing something. MoUs between the network hospitals and network organisers (NEST360 Kenya) helped to solidify commitment to the *collective vision*. The NEST360 network has also been able to create broad awareness and support around its *collective vision* from outside the network, from government and health sector partners, which supports an enabling environment for the network and network activities.

| 2A | When potential network members or network initiators engage in an open process of collective sense-making around a problem (context), they can identify what they share in common (outcome) because they learn about each other’s experiences, emotions, understandings, or perspectives (mechanism) | Refined |
| --- | --- | --- |
| 2B | If potential network members or network initiators identify and articulate commonalities among each other (context), then this enables the development of a collective network vision (outcome) because they understand each other’s perspectives (mechanism) | Refined |
| 2C | When potential network members and/or network initiators have common professional or vocational identities or calling (context), this facilitates the development of a collective network vision (outcome) because they are more likely to have common professional perspectives and values (mechanism) | Refined |
| 2D | When network initiators introduce the idea/intention of the network to potential members when the network is forming and recruiting network members (context), this will generate early commitment to the collective vision (outcome) because potential network members understand the network’s purpose (mechanism) | New |
| 2E | If a network’s collective vision is based on shared experiences, emotions, perspectives, and understanding among the potential network members and network initiators (a similar specific way in which reality is perceived) (context), then this will lead to commitment to the collective vision (outcome) because potential network members feel represented (mechanism) | Refined |
| 2F | If potential network members know each other prior to network formation (context), this can help generate commitment to the collective vision (outcome) because there is existing trust between the potential network members (mechanism) | New |
| 2G | When potential network members engage in an open process of collective sense-making around a problem (context), this generates a sense of ownership around the collective vision (outcome) because potential network members feel heard/represented (mechanism) | New |
| 2H | When network leaders/champions consult and engage hospital/facility leadership (context), this generates commitment to the collective vision from the administrative hierarchy even if they have no leadership role within the network (outcome) because they understand its value (mechanism) | New |
| 2I | If the collective vision of a network is communicated and explained by network leadership in a way that appeals to professional ideals and values shared by potential network members (context), then this will lead to commitment to the collective vision (outcome) because potential network members feel engaged (mechanism) | Refined |
| 2J | When the network provides resources to support making changes to the physical infrastructure of facilities within the network (context), this encourages potential network members to commit to the collective vision (outcome) because potential network members feel that the network is doing something for them (mechanism) | New |
| 2K | When potential network members agree to an existing collective vision (context), they will be more willing to take on activities to enact the collective vision (outcome) because they have an understanding of what is expected of them (mechanism) | Refined |
| 2L | When formal agreements have been negotiated and agreed between network members (context), then the network members will feel solidified in a network and be more likely to follow a collective vision (outcome) because the agreements help network members to understand their roles and responsibilities (mechanism) | Refined |
| 2M | If there is no collective vision or if network members do not follow the network’s collective vision (context), then they will be less successful in implementing network activities (outcome) because network members do not feel committed to the network (mechanism) | No supporting data |
| 2N | When the network makes an effort to disseminate its vision to solve the identified problem beyond the network (context), then non-network stakeholders (e.g. partners, donors) will be more aware of the network (outcome) because network members have advocated for its purpose (mechanism) | New |
| 2O | When the network engages with government officials and takes their inputs into consideration (context), greater support is generated for the network and network activities (outcome) because there is closer alignment of vision between the network and the government, and government officials feel valued and respected (mechanism)  *“I would say it's probably the same things we struggle with within the broader CIN network is to what degree is Ministry involved what degree are professional associations involved. You can only engage Ministry people to a certain degree because the person responsible for child and newborn also have very many other things they're looking after. NEST is not the only newborn the only important thing in the newborn space. So, they have to look after people who do like KMC, all these resuscitation courses, they have to look at guideline development, how to partner with colleagues in child health and maternity so they're also spread pretty thin. And, and you can only engage them to a certain degree. So, there's a lot of effort to make sure that they're part of the meetings. They have a lot of the technical input, there is a lot of effort to ensure people being involved in quality improvement and Ministry people, Ministry sanctioning, pretty much leading being put on the driver's seat when there is like a proper technical meeting happening around NEST. So, it's not a NEST meeting it's a Ministry meeting. But that's I guess that's the most you can do. I can't I have no no magic bullet for it. I think it's just continually and but I think it's also I think one way is to, if there is a way of helping the Ministry get part of the NEST activities funded by government so that there is an activity in their work plan that aligns with NEST, then there is sort of joined at the hip in some way, in some way. So, I don't know what that activity would be. But I think that's if there is a way of advocating to get somethings funded by central government. One is a commitment. It's one is is a sign that the government is interested and sees the value of it and is investing in it. So even people in government see, can read that, and therefore see this as an important deliverable.” (FI 32)* | New |
| 2P | When network leadership/organisers are engaged in government meetings (e.g. technical working groups) (context), this can improve the enabling environment for the identified problem/collective vision (outcome) because more stakeholders are aware of and engaged with the problem/vision (mechanism) | New |

Table 2: CMOCs for Collective vision

**Taking action to solve a problem**

Consistent with our Realist Review, this part of the programme theory begins with the need to recruit others to join the collective vision. In a bottom-up network, members will continue to recruit colleagues to the collective vision, particularly those who are likeminded, capable, and willing to help to support *taking action*. As this evaluation is focused on an externally initiated network, there were no data to support this CMOC (3A, modified based on totality of data), but it may have relevance for the network initiators (NEST360 Global). In an externally initiated network, the energy and effort put in to recruit others ensures a diverse mix of skills and competencies (CMOC 3B, refined). Network members will also seek out those with common experiences or perspectives (CMOC 3C, refined).

The intention to *take action* will be supported by the existence of resources, opportunities for meetings to coordinate efforts, teamwork within and across cadres and organisations, supportive leadership, platforms for communication, and purposeful activities, such as QI and mentoring (CMOC 3D, confirmed, 3E - 3J, new). As the network evolves, members need to continually *take action* to address the identified problem, potentially by tackling smaller problems that contribute to the larger problem causing the strain that initiates the network. The support that network members receive to *take action* builds their capabilities and willingness to solve problems that may support the network as it evolves and adapts overtime (See Adaptability). However, if network members fail to make progress towards solving the problem, this may undermine the relationships and support the network provides and discourage network participation (CMOC 3K, new). When network initiators see the opportunities and resources related to the identified problem (see Identify a problem), this influences their intention and willingness to collectively *take action* on the identified problem and therefore distinguishes doing something about the problem from not getting initiatives off the ground.

The supporting theories from our Realist Review — Collective Identity Approach from New Social Movement Theory,^5-7^ the Theory of Planned Behaviour,^9^ and Self-Efficacy Theory^10^ (Appendix 4) — are still relevant to this part of the refined programme theory.^9^ Ajzen and Bandura’s theories help to explain that if network members believe that collectively they see the opportunity and have the resources to solve the identified problem, they will have more intention to perform the behaviour, (i.e. form the network), and they are more likely to do so.

In the NEST360 network, NEST360 Global recruited a diverse mix of partners with complementary skill sets and expertise to organise the network in Kenya. Within network hospitals, NBUs recruited other hospital staff to support their unit in working towards the collective vision. The resources (equipment, parts, and training) NEST360 provides encouraged network hospitals to *take action* because with the necessary resources, they had more capacity to improve care for small and sick newborns. The feeling of working as a team within and across facilities, NEST360 network meetings, support from NEST360 Kenya for mentoring, QI, and training, and network WhatsApp groups all supported the hospitals in *taking action* towards changing their newborn care practices.

| 3A | In a network formed from the bottom-up when network members realise they alone are unlikely to be able to solve a problem (context), they will recruit likeminded colleagues for their cause (outcome) because they believe collective action is needed (mechanism) | Refined |
| --- | --- | --- |
| 3B | In an externally initiated network when network initiators and organisers realise that they need diverse health system actors to be able to solve the problem/meet the target (context), they will recruit/mandate/encourage colleagues they perceive to be capable of helping their cause (outcome) because they believe a collective approach is needed (mechanism) | Refined |
| 3C | When network members think collective action is needed to solve the identified problem (context), they will seek out other individuals with common experiences or perspectives (outcome), because they believe such people may be willing to help them (mechanism) | Refined |
| 3D | When members in an established network have access to resources that will help them solve the problem they have identified (context), they may be more prepared/more likely to intend to take action (outcome) because they believe they have a more realistic chance of success (mechanism) | Confirmed |
| 3E | When an externally initiated network is forming and opportunities are provided to network members to meet, discuss, and find solutions to problems (context), this will support network members to take action and shape an identity and a sense of belonging (outcome) because they are able to coordinate their work and work together | New |
| 3F | When network members of different roles/cadres/units/organisations work together as a functioning team (context), this enables them to take collective action towards solving the problem (outcome) because they have a common understanding of what needs to be done (mechanism) | New |
| 3G | When network leadership or organisers are supportive of network members taking action (context), network members will be more likely to take practical and concrete action to solve problems (outcome) because they feel their efforts are valued and worthwhile (mechanism) | New |
| 3H | 1. When trained network members are provided with support (e.g. quality improvement and mentoring) that is relevant to the problem they are addressing (context), they are more able to take practical and concrete action to solve problems (outcome) because they feel empowered (mechanism) | New |
|  | 2. When information that makes challenges visible emerges from supportive activities (e.g. quality improvement and mentoring) (context), network members feel a greater need to address the problem (outcome) because they feel accountable to network leaders/organisers (e.g. mentors, quality improvement team) | New |
|  | 3. When network members take practical action as a team (context), this mobilises the network members’ efforts and resources (outcome) because they feel internal accountability towards other network members (mechanism) | New |
| 3I | When network members are provided with a platform for them to easily communicate (e.g. WhatsApp groups) (context), they are more able to take practical and concrete action to solve problems (outcome) because they can access and gain the knowledge and reassurance they need (mechanism)  *“This was part of the initial plans, and I'm happy that it got it kicked off well. After the training, in fact it usually starts before the training, so that we're able to do communication early enough to enable them to prepare well. After the training, we do have a WhatsApp group for ease of quick communication. Sometimes they even learn from themselves in those WhatsApp groups. And the good thing is that we do have biomedical WhatsApp groups, clinical team also has theirs. And then we have common shared groups of the focal the focal persons. These are the these are the to go to people that link the programme team that normally and Dolphine, and even the other directors and the facility teams. So, they're the people who if we needed to get any updates, quickest possible, they're the people that we go to. So, we have also another common WhatsApp group for such kind of teams. So, this has both biomeds and clinical paediatricians. It's a mix of all those people. Then separate from that we have biomedical team aside, clinical team WhatsApp group aside, within the NEST’s much larger team. We also have cross-country. I mean, we have a common group for the cross-country teams. Like the training managers, both clinical and technical, we have one that is common for all the four countries. And of course, led by the country liaison team that’s now Edith and Millicent again separate from that they do have clinical aside for the four countries. And then we have another technical one for the NEST engineers for the four countries together with Hatch team in that composition. So that’s how we we are able to address issues quick as possible. Because sometimes, you will find in some other cases like for example, let me give you an example of a device. We have a device the Billidx Bilirubinometer machine. So far, we’ve experienced two errors that have been encountered in the field, Tanzania, Nigeria, Kenya. Malawi is yet to report. And those two errors do not exist within the user manual. They are not there. So, we whoever experiences it is free to post on WhatsApp, and we try to discuss it there. If it’s something that somebody already encountered and was able to manoeuvre around you can share how that you were able to do that.”* (FI 15) | New |
| 3J | 1. If network leaders/organisers continue to put effort and work into solving the identified problem (context), this supports network members to make progress towards changes in practice/improvements (outcome) because they feel encouraged to do so (mechanism) | New |
|  | 2. When efforts are made to provide consistent support to network members from network leadership/organisers (e.g. through quality improvement and mentorship) (context), it promotes network members to take action (outcome) because network members develop a bond with those providing support (feeling part of a larger team) and feel empowered by and accountability to them (mechanism) | New |
| 3K | If, despite the support provided, network members fail to act or make progress (context), this starts to undermine the relationships between network members and those providing support (outcome) because those providing support feel the network members do not value their efforts (mechanism) | New |

Table 3: CMOCs for Taking action to solve a problem

**Identity and culture**

A network’s identity and culture comes from a feeling of collective identity among network members that is interlinked with identifying with the network’s collective vision. When network members come together to work towards a shared problem, they get a sense of fulfilment (CMOC 6A, confirmed) and potentially belonging; however, the latter was not supported by primary data (CMOC 6B). When network members identify with other network members and its vision, they will develop a *network identity* (CMOC 6C, confirmed). If the network celebrates contributions made by those network members who had previously felt unrecognised in their work environment prior to the formation of the network, this can give them an *identity* *in the network* as important network members (CMOC 6D, new). When the *identity of a network* is linked with network initiators or partners providing resources to the network, this may create a separate and potentially unsustainable *identity,* not integrated into the health system (CMOC 6E, new). The demonstration and reinforcement of the *network identity* by network leadership, champions, and members can help other network members to adopt the *identity* (CMOC 6F, new). A network creates opportunities for members to connect, share experiences, and learn from each other in an open and safe environment, which creates a positive *network culture* and a sense of belonging to the network (CMOC 6G, refined). Network members can also shift negative or unsupportive pre-existing cultures to better align with the *network’s culture* (CMOC 6H, refined).

The three substantive theories from this part of our Realist Review continue to be relevant with the primary data: New Social Movement Theory,^5-7^ Organisation Culture Theory,^11 12^ and Tuckman’s Small Group Development Theory^8^ (Appendix 4). Another view on organisational culture, Culture Typology, developed by Quinn and Cameron, posit there are four different types of culture in organisations: clan, hierarchical, market, and adhocracy ^13^ (Appendix 4). Networks try to create a clan culture, which focuses on collaboration, has high engagement and commitment from members, values relationships and teamwork, promotes shared values and goals, and empowers members to facilitate commitment and loyalty to the organisation.^13^ Networks, however, exist within health systems, which are hierarchical in culture. Hierarchical cultures are procedural, structured, and controlling.^13^ Certain networks, particularly those that are formed from the bottom-up, may have elements of adhocratic culture, which is entrepreneurial with risk-taking leaders.^13^ When networks change pre-existing cultures, this is likely a movement from a hierarchical to clan culture, which can decentralise power and authority to midlevel managers and distribute leadership.

NEST360 aims to create a clan culture in the network, even though it is not something that the network initiators and organisers explicitly set out to do. The creation of *identity and culture* are important to how a network functions and performs and therefore is worthwhile to build. NEST360 created a *network identity and culture* that stems from network members’ passion for newborn care. Network hospitals and members feel part of a *culture* to improve newborn care. The provision of resources, for example training, equipment, and mentoring activities, helped to create a separate *network identity,* but this may make the *identity and culture* less sustainable when these resources are no longer provided to the network hospitals. However, NEST360 creates opportunities across network hospitals for members to connect, which reinforces a sense of belonging to the network and spreads its *culture*. Findings also show that for some network hospitals, a positive pre-existing *culture* that aligns with the network’s vision can positively support the development of a *network identity and culture*.

| 6A | When network members with a collective identity come together to solve a shared problem (context), this makes them feel fulfilled (outcome) because of a sense of shared purpose (mechanism)  *“And you see, you are part of that NBUs. It's NBUs this machine is not working, you see in you you are part and parcel of that. And when everything goes well, you know that everything is okay, today we are okay. You feel good. Because you know that newborn that premature baby will be there maybe for one week, two weeks, and if they are, okay. Now with that you see now, in your profession, you are something you are proud proud of.” (FI 24)* | Confirmed |
| --- | --- | --- |
| 6B | When network members feel fulfilled from working with likeminded people in a network (context), they want to belong to the network (outcome) because they feel it is worthwhile (mechanism) | No supporting data |
| 6C | When network members identify with other network members and the network’s vision (context), they develop a network identity (outcome) because it gives them a sense of purpose (mechanism) | Confirmed |
| 6D | When a network celebrates the contributions made by previously unrecognized/ unacknowledged hospital unit/facility leaders (context), this gives hospital unit/facility leaders an identity in the network as important network members (and in the hospital/facility) (outcome) because their expertise and role is now more valued by others (mechanism) | New |
| 6E | When the identity of a network is linked with network partners/initiators providing resources to the network (context), the network has a separate identity that may make it less sustainable (outcome) because the network partners/initiators have not put in sufficient effort to integrate the network in the health system (mechanism) | New |
| 6F | When network leadership and members consistently and regularly demonstrate and reinforce the network identity (context), this helps other network members adopt the network identity (outcome) because of role modelling (mechanism) | New |
| 6G | When a network creates opportunities for members to connect, share experiences, and learn from each other in an open and safe environment (context), this creates a positive network culture and a feeling of belonging to the network (outcome) because members feel respected, valued, and acknowledged (mechanism) | Refined |
| 6H | When local network leadership and members recognise that the network’s culture is not aligned to existing cultures in their environment (context), then they may attempt to change the pre-existing cultures to make room for the network culture and practices (outcome) because they no longer identify with the pre-existing culture and see value in the network culture (mechanism) | Refined |

Table 4: CMOCs for Identity and culture

#### Network activities

Networks undertake certain activities for Initiation & Forming, Functioning & Performing, and Sustaining Change & Impact (see Activities in Figure 1 and Appendix 5). There may be other activities that networks undertake but based on the primary data and our Scoping and Realist Reviews, the ones described below are likely the most important. Of these activities, only leadership was present in our Realist Review; the other activities emerged from primary data analysis, however, the literature from our Realist Review supports this evolved understanding of the programme theory and the inclusion of these network activities. The concept of Relational Coordination, which is a theory about how stakeholders coordinate by communicating and relating to achieve outcomes^14^ (Appendix 4), lends itself to different aspects of networks, particularly the network activities that we have identified. The underlying CMOCs for these network activities are provided in Tables 5-10 and we have provided one piece of illustrative data for one CMOC in each network activity and additional illustrative data supporting the CMOCs is available in Appendix 8.

**Knowledge and skills dissemination**

Disseminating *knowledge and skills* is one of the main ways networks perform. In our Scoping Review under the framework component ‘Mode of Functioning,’ the most frequent practical characteristic was ‘knowledge/information sharing, education, and learning.’^15^ When a network *disseminates knowledge and skills* by creating training opportunities, this can change and improve practices related to the identified problem (CMOC 14A). Network members may feel more supported and capable and may be more likely to adopt new practices through mentorship activities (CMOC 14B.1, 14B.2). Network members may also *disseminate knowledge and skills* to other departments within the network facility, which improves linkages between departments (CMOC 14C). When network members create links with non-network facilities, this also creates opportunities for sharing *knowledge and skills* (CMOC 14D).

*Disseminating knowledge and improving the skills* of clinicians, nurses, and biomeds is an important aspect of the NEST360 network. One of the first activities the NEST360 Kenya team undertook was to update the Emergency Triage, Assessment, and Treatment (ETAT+) protocols. This was the basis for the in-service and pre-service training for clinicians and nurses and incorporated aspects of NEST360 supported device usage. Pre-service and in-service training materials on NEST360 supported devices were developed for the biomeds. Biomeds were trained on the maintenance and repair of devices as well as usage. The network created a training course to train trainers in teaching skills as well as content (General Instructors Course), while mentors helped to instil the *knowledge and skills* learned through the trainings. NBUs have integrated maternity and postnatal wards into the facility trainings and Continuing Medical Education (CMEs) which has improved their relationships with other departments with which they often liaise. Staff at facilities that refer (often Level 4 hospitals) to the NBUs in the network are often included in CMEs and facility trainings.

| 14A | When the network adequately shares knowledge and skills throughout the network by creating training opportunities (context), this changes/improves practices around the identified problem (outcome) because network members are more capable (mechanism)  *“And then, of course, there has been a lot of change of practice change in practice in terms of like, maybe I'll give an example for jaundice. In every NEST hospital, you will notice jaundice or phototherapy is our game changer. Previously, people actually just thought phototherapy is just putting baby under the light. They didn't know it had to be measured. So, a lot of places actually had lights that were on but we're not giving the required irradiance. So now that people are measuring, people are telling you babies are just within one to two days, they're out. Previously, we'd had babies staying on photo for a week or more and all that. But now they realise, well actually we had to measure this.” (FI 14)* | New |
| --- | --- | --- |
| 14B | 1. When network members disseminate knowledge and skills through mentorship activities (context), network members feel supported and capable (outcome) because they develop the knowledge and skills needed to do their job well (mechanism) | New |
|  | 2. When network members regularly disseminate knowledge and skills through mentorship activities (context), network members are more likely to adopt new practices (outcome) because the mentors continuously reinforce the practices (mechanism) | New |
| 14C | When network members include colleagues in other hospital/facility departments in knowledge sharing opportunities (context), this improves linkages between network members and other departments (outcome) because they respect and value the help provided (mechanism) | New |
| 14D | When network members include providers in non-network facilities in knowledge sharing opportunities (context), this supports their ability to improve practices (outcome) because they develop essential skills and knowledge (mechanism) | New |

Table 5: CMOCs for Knowledge and skills dissemination

**Cross-learning**

A common activity that networks use to perform is *cross-learning* within and across network facilities. Within facilities, networks facilitate inter-professional *cross-learning* by bringing together network members from different professions and departments to share their experiences on problems and solutions (CMOC 13A). When a network makes an effort to connect members from different facilities, this helps them to learn new approaches to solve problems better and facilitates inter-facility learning in the network (CMOC 13B.1, 13B.2). This learning occurs through network meetings, supportive supervision, mentoring, QI activities, webinars, and creating linkages across network facilities. Network meetings are one of the main opportunities for *cross-learning* and can help network members to solve problems better (CMOC 13C). *Cross-learning* can continue remotely through WhatsApp and other communication platforms because of the purposeful relationships that have been established between network members at different facilities through network meetings and activities (CMOC 13D). When networks in different geographies are connected, this provides more opportunities for network members to learn how to solve problems better (CMOC 13E).

*Cross-learning* is an important network performing activity to highlight because it is a common way that practices are changed in networks. When a network brings together members, there are opportunities both for *cross-learning* and developing professional relationships (See Purposeful Relationships, Linkages, and Partnerships). Continued *cross-learning* may be more likely when purposeful relationships or connections have already been established. *Cross-learning* can take different shapes and levels of formality over the evolution of a network.

By providing opportunities to connect network members within and across facilities, the NEST360 network formed purposeful relationships and linkages among network members (See Relationships, Linkages, and Partnerships) and created opportunities for *cross-learning*. Through inter-professional trainings, clinical and biomedical network members have established better working relationships and learnt from each other, which has improved the overall care provided on the NBU. The NBU has established better linkages with the labour and postnatal wards and included those providers in their trainings and CMEs. The NEST360 network has also accomplished *cross-learning* across hospitals in the network through network review meetings, supportive supervision, mentoring, and QI activities, which provides opportunities for network members to exchange with or visit different hospitals in the network. NEST360 has also instituted network webinars, some of which have been open to external participants, providing another avenue for *cross-learning*. Network meetings are a key opportunity for *cross-learning* and provides nurses, clinicians, and hospital administrators opportunities to share experiences, learnings, and challenges with each other. Network members also use WhatsApp groups to continue learning from each other in-between more formal learning opportunities. While this research is only focused on NEST360 in Kenya, *cross-learning* also occurs among the larger four-country network, though less frequently than through the networks within countries.

| 13A | When the network brings network members together from different professions within the same facility to share their experiences on problems and solutions (context), this facilitates intra-facility cross-professional learning (outcome) because they understand the value of different roles in achieving the shared desirable change/outcomes and feel their challenges are acknowledged and inspired by different approaches from their peers (mechanism)  *“And then the educational piece, which is also very heavy. I think it's it's unique in a way. Because the first one we do is to provide a course that brings together the clinicians and the biomeds and that allows cross collaborative learning. And what we've seen happen with that is at the facility then there's a lot of collaboration between the nurses and the biomeds and so the nurses telling us that sometimes they're able to see when a device is not working and they can call the biomeds. So that's something that was not happening before.” (FI 18)* | New |
| --- | --- | --- |
| 13B | 1. When the network makes an effort to connect network members from different facilities in the network (context), this facilitates inter-facility learning (outcome) because they feel their challenges are acknowledged and inspired by different approaches from their peers (mechanism) | New |
|  | 2. When the network makes an effort to connect network members from different facilities in the network (context), this helps them learn new approaches to better solve problems (outcome) because network members can share their experiences and knowledge and see their own work/setting in a new light (mechanism) | New |
| 13C | When the network brings together network members through meetings (context), this helps them to better solve problems (outcome) because they learn from each other (mechanism) | New |
| 13D | When network members have created purposeful relationships through network meetings and activities (context), they are able to continue their cross-learning remotely (e.g. WhatsApp) (outcome) because they have an already established bond (mechanism) | New |
| 13E | When the network makes an effort to connect network members with networks in other geographies (within or across countries) (context), this helps them to better solve problems (outcome) because network members can share their experiences and knowledge (mechanism) | New |

Table 6: CMOCs for Cross-learning

**Resourcing networks**

Networks require different *resources* to be initiated, form, function, perform, and ultimately be sustained or sustain the changes in practice and impact. These resources can be tangible, such as financial resources, human resources, infrastructure, medical devices, and pharmaceutical products or intangible, such as time of network members. Networks will need varying levels of *resourcing* at different times in the network’s evolution. External *resources* may not always be available to a network and a network that relies on external *resources* will likely need alternative sources of these *resources* for the network to continue to perform and sustain the changes in practice. External *resources* may be transitioned out of networks overtime to support a gradual increase in local *resources*.

If the network exists in an under-resourced health system, this may make sustainability of the network and network activities challenging (CMOC 16A). Network sustainability may also be challenging when a network is heavily dependent on external *resources* and not sufficiently integrated into the health system (CMOC 16B) or when the network does not have sufficient human *resources* required to changes practices and sustain the network impact (CMOC 16C). When the network shares the collective vision with stakeholders, such as partner organisations, this can help sustain the network and extend the network’s reach (CMOC 16D). Furthermore, if the network makes an effort to engage local or sub-national government in the network and the collective vision, members may be able to use their skills and *resources* to support network activities and sustainability (CMOC 16E).

The Kenya health system is plagued with financial, pharmaceutical, consumables, equipment, and human *resources* challenges. While the NEST360 network has provided significant financial *resources*, infrastructure upgrades, equipment, parts, consumables, and capacity building activities (e.g. training, mentoring, QI) into the health system, as NEST360 begins to transition direct *resource* support from existing network hospitals, there may be challenges for the health system to match the level of *resource* input provided by NEST360. Furthermore, the network hospitals are understaffed and so existing staff may have challenges in sustaining the changes in practice and network activities. As NEST360 moves to transition direct support from existing network hospitals, the network organisers (NEST360 Kenya team) are engaging with stakeholders and county governments to help sustain network activities and the network’s reach.

| 16A | When the health system is insufficiently resourced (context), transition and sustainability of the network and network activities may be challenging (outcome) because there is limited capacity to sustain the network’s impact (mechanism) | New |
| --- | --- | --- |
| 16B | When an externally initiated network is heavily dependent on external resources and not sufficiently integrated into the health system (context), this will make transition and sustainability of the network and network activities challenging (outcome) because the health system and stakeholders may not be able to prioritise absorbing the required investment (mechanism) | New |
| 16C | When the network does not have sufficient human resources required to change practices and sustain the changes (context), this will make transition and sustainability of the network and network activities challenging (outcome) because the network has limited capacity to institutionalise its impact (mechanism)  *“But you see, now we also think that the biggest challenge because I still believe that figure can come down, if you only have if you only have enough human resource because now so yeah, you have a single nurse. Let's say you have within five, five CPAPs. See remember, the CPAP needs to be monitored. At least every hour. Lactic acid is not kinking of the tubes, there is no secretions. Now you only have one nurse. So even if you have to put five babies on CPAP, most likely monitoring is not possible, and this same same nurse then is supposed to titrate leads.” (FI 10)* | New |
| 16D | When the network shares the collective vision with stakeholders (e.g. partner organisations) outside the network (context), this can help with sustainability and extending the network’s reach (outcome) because they can understand the value of the network (mechanism) | New |
| 16E | When the network makes efforts to engage local/sub-national government in the network and the collective vision (context), members can use their skills and resources to support network activities and sustainability (outcome) because they appreciate the value of the network (mechanism) | New |

Table 7: CMOCs for Resourcing networks

**Leadership**

*Network leaders* have a formal responsibility in network operations and decision making. *Leadership* differs depending on if the network was initiated from the top-down, by external stakeholders, organically, or from the bottom-up. As we collected the primary data on a network initiated by outside global health partners, we revised the CMOCs to take this background into consideration. We were unable to confirm, refute, or refine five of the CMOCs from the initial programme theory from the primary data (5B, 5D, 5G, 5H, 5Q), which may be because these CMOCs are more relevant for networks that are initiated from the bottom-up or form organically. From the primary data, we identified other important aspects of *leadership* in networks that did not emerge from the literature in our Realist Review. These aspects were added in nine new CMOCs (5A, 5N-5P, 5R-5T). The primary data confirmed one CMOC (5I) and refined seven (5C, 5E, 5F, 5J-5M).

In networks that are initiated and/or supported by external organisations, they will recruit *network leaders* they believe have the competencies to contribute to network formation and functioning (CMOC 5A). In order to set up the network and keep it going, network leaders enact *leadership* capabilities and communicate the network’s vision and goals to bring network members around the vision and move forward in the same direction (CMOC 5C). *Network* *leaders* provide support and feedback to network members to support network functioning to take action to solve the identified problem (CMOC 5E). *Network* *leaders* can influence network members to develop greater commitment, engagement, and/or motivation when they get things done (CMOC 5F).

In order to support the network to work towards the collective vision, *network* *leadership* will consistently and regularly support and provide resources to network members (CMOC 5I). *Network leaders* and members can influence other network members to adopt network practices by promoting, encouraging, and championing practices (CMOC 5J). *Leadership* can create a welcoming, supportive, and inclusive network environment, which can support network members to seek support to improve knowledge, skills, or manner of working (CMOC 5K).

A network needs to grow and cultivate *leadership*, which can happen when networks have processes in place to identify and enable those with the necessary skills, motivations, or attitudes to take on *leadership* roles (CMOC 5L). There can be challenges with *network leadership* when a network is highly dependent on a few people or organisations to implement or support network activities, there is misalignment between *network leaders*/organisations, there are *leadership* changes within the network, *leaders*/organisations exit the network, and new *leaders*/organisations introduce new working practices (CMOC 5M – 5O). Changes to non-network stakeholder *leaders*, whose commitment to the network is important for network functioning, can also undermine network functioning (CMOC 5P). Networks can mitigate these *leadership* challenges, such as new network *leadership* putting in effort and network organisers leveraging their influence and effort among others in the network (CMOCs 5R – 5T).

Smelser’s Theory of Collective Behaviour,^3 4^ Tuckman’s Small Group Development Theory,^8^ and Ganz’s theorising on *leadership* in social movements^16 17^ (Appendix 4) continue to be relevant in the revised programme theory. Additionally, networks are relational systems^18^ (Appendix 4) and functioning networks are an example of when relationships are prioritised and reinforced by *leaders*.

There are different layers of *leadership* in NEST360: NEST360 Global (Rice University and partners), NEST360 Kenya (Aga Khan, KWTRP, Kenya Paediatric Research Consortium (KEPRECON)), and *leaders* at the hospitals (medical superintendents) and within the NBU (nurse-in-charge, paediatricians). Each of these *leaders* enact different *leadership* capabilities in different parts of the network that are important for network initiation, formation, functioning, performing, and sustaining change and impact. One participant saw *leadership* as an important success factor: *“so leadership to me was one one big unaccounted for factor in the success of this programme” (FI 31)*. This statement links to one of our overall findings in the evaluation – that there are many other factors that need to occur or be in place (that make up the network) for the central activities that NEST360 has put in place to work. Among the NEST360 Kenya *leadership* (network organisers), there were several changes in partner organisations. This made implementation of network activities challenging, delayed, and frustrating for the team providing support to the hospitals, who remained mostly the same.

| 5A | Networks, initiated and supported by outside organisations/partners (context), will recruit organisations or individuals into leadership positions that they think are competent for network formation and functioning (outcome) because they believe these organisations or individuals understand what the network needs to succeed (mechanism) | New |
| --- | --- | --- |
| 5B | When networks have a member(s) that clearly takes on a leadership role, focuses on building linkages between potential network members and stakeholders, and creates communication channels (context), this helps the network to form (outcome) because it brings people together (mechanism) | No supporting data |
| 5C | When networks have a member(s) that enacts leadership capabilities, communicates the network’s vision, and helps to set collective goals (context), this helps to bring network members around the collective vision and to move forward in the same direction (outcome) because they have the same understanding of the network’s value (mechanism) | Refined |
| 5D | When networks have a member(s) that clearly takes on a leadership role and actively coordinates network members (context), this helps the network to form (outcome) because there is a central figure bringing people together (mechanism) | No supporting data |
| 5E | When networks have a member(s) that enacts leadership capabilities and provides support and feedback to network members (context), this can help the network function and take more appropriate action towards the identified problem (outcome) because network members feel empowered to take action to work towards achieving the vision (mechanism) | Refined |
| 5F | When networks have a member(s) that enacts leadership capabilities and gets things done (context), network members develop greater commitment, engagement, and/or motivation (outcome) because they believe belonging to the network is worthwhile (mechanism) | Refined |
| 5G | When networks have a member(s) that takes on a leadership role and dedicates time to setting up and the functioning of the network (context), network members develop greater commitment, engagement, and/or motivation (outcome) because they believe belonging to the network is worthwhile (mechanism) | No supporting data |
| 5H | When networks have a member(s) that clearly takes on a leadership role and actively engages network members (context), network members develop greater commitment, engagement, and/or motivation (outcome) because network members feel part of the network (mechanism) | No supporting data |
| 5I | When network leadership consistently and regularly supports and provides resources to network members (context), this helps network members act to achieve the network’s collective vision (outcome) because the network members feel empowered (mechanism) | Confirmed |
| 5J | When network leadership and members consistently and regularly promote, encourage, or champion practices or influence network members to take up certain practices (context), this helps other network members to adopt these practices and sustain them in the network/unit (outcome) because of role modelling (mechanism) | Refined |
| 5K | When network leadership creates a welcoming, supportive, and inclusive environment within the network (context), members feel more able to seek support to improve knowledge, skills, or manner of working (outcome) because network members feel that they are in a psychological safe environment (mechanism) | Refined |
| 5L | When networks have processes in place to identify and enable those with the necessary skills, motivations, or attitudes to take on leadership roles from a wider pool of engaged human resources (context), this may support network functioning and may lead to changes happening (outcome), because the network is able to create a distributed form of leadership across the network (mechanism) | Refined |
| 5M | If a network is highly dependent on a few people or leaders to implement network activities or leaders that support their implementation (context), then it is at risk of being unsustainable or functioning poorly (outcome) because the leadership structure is fragile (mechanism) | Refined |
| 5N | When there is misalignment of priorities between network leaders/partner organisations (context), then one leader/partner organisation may feel they need to leave the network (outcome) because they do not feel they are moving in the same direction (mechanism) | New |
| 5O | 1. When there is a leadership/partner organisation change within the network and a new leader/partner organisation comes in (context), this can jeopardise maintaining the network’s collective vision, existing relationships, and functioning (outcome) because the new leader/partner organisation is external and has not assimilated into the network (mechanism) | New |
|  | 2. When a leadership change within the network introduces new working practices in the network (context), this can disrupt network activity implementation and functioning (outcome) because network members need to learn about new practices (mechanism) | New |
|  | 3. When a leadership change within the network introduces new working practices without first discussing and negotiating these with network members (context), network members can become frustrated (outcome) because their experience and expertise in the network is not valued (mechanism)  *“I'll honestly say that it has impacted it has okay let me say it has had an impact on the programme as such that the implementation slowed down at some point, yeah. As a programme manager, I think I can say for any programme manager something that can be frustrating is when you lack… to do something to to implement, to be able to implement, you know. So like, for example, when we move from CPHD, of course, there is a way there was a system on how, you know, you are running a programme, and then move to the next let me say stage where we are out of CPHD, and we have to continuously maintain the programme. And then just as when we are getting the momentum and coming up with systems to be able to implement smoothly, then another transitioning comes on board where we moved now to, I don't know Strathmore University. And then of course, then they have their own policies, they have, you know. Everywhere you go with a partner, they have their policies and system on how they're doing things. So, when you have a programme that is running that has to continue running and on the other side, you have to start abiding afresh, to the other partners, policies and system of how they're doing things you're used to doing… if anything comes up with any programme implementation there has to the flexibility. So, you realise when something comes in between the planned activities in that particular month, now, having moved to having joined another partnership here, it becomes difficult to manage anything else that comes in between. So that was quite frustrating, especially the one that you're given restriction when it comes to timelines, you cannot do this at this time, we are time bound we cannot organise a training... and because you've moved to the new partnership, and they feel like no we are time bound we are not able to book a conference space for you, we are not able to organise transport for you, I mean it becomes frustrating. Then just as we are settling again, another transitioning happens now from Strathmore to now having to partner with a consultant, you know, to support us with the finance systems, the operation system, basically when it comes to logistics and you know finance. And then just as we are also picking up that momentum, now we have to transition again. Now we are currently in Aga Khan. I mean, it can be frustrating for any programme implementation because even now at Aga Khan they now have, of course, even that consultant had his own system and ways of doing things. This affects a lot the flexibility on how they programme activities can be implemented. And of course, now getting into Aga Khan now fully transitioning to Aga Khan, where now finance has to be, of course, operated by them, logistics, and when you want something to be to be done, there's a lot of bureaucracy of how things are going to be improved, approved. Sorry. And so that for that particular activity to take place, I mean, it's when you start settling down, you have to start over again. I mean, it's that kind of a cycle. So, we've been oscillating around transitioning more than how focusing on you know, programming implementation and ensuring quality of what we desire to achieve. Yeah, so I think that has also affected a little bit of the quality, but even so with that I can say, I can tap our backs, I mean, as the NEST Kenya team that we've actually managed to push through a lot of activities. I mean, just just becoming flexible ourselves, putting the frustrations aside, and, you know, trying to keep that focus and and yeah, moving on…transitioning has had an effect big an impact big time on programme implementation. Yeah, and it can be very frustrating.” (FI 20)* | New |
| 5P | When non-network health system leaders, whose buy-in/commitment to the network is important for network functioning, change (context), this can disrupt network activity implementation and functioning (outcome) because it puts at risk relationships essential for network functioning (mechanism) | New |
| 5Q | When network leadership is not able to get the network to develop and agree on a shared vision (context), then it is at risk of not performing well and becoming unsustainable (outcome) because members are unclear as to what the network is for (mechanism) | No supporting data |
| 5R | If network members take appropriate action to mitigate any disruption leadership changes might bring (context), then the network is more likely to keep functioning (outcome) because the impact of any disruptions are minimised (mechanism) | New |
| 5S | When there is a change of leadership within the network and new network leadership puts in efforts to maintain network functioning (context), network members feel that it is still worthwhile to be part of the network (outcome) because they feel the leaders value the network (mechanism) | New |
| 5T | When hospital/facility leadership sees the effort that the network is making and the changes that have resulted (context), this encourages them to take action to work towards the collective vision (outcome) because they understand the value of the work (mechanism) | New |

Table 8: CMOCs for Leadership

**Champions**

*Network champions* are important for initiating a network, its formation, ongoing functioning and performing, and sustaining change and impact. *Champions* may or may not be a formal network leader or have a formal leadership role in their facility/organisation, though they are respected by their colleagues, may regularly provide support, and are advocates for the network cause. *Champions* have the skills, passion, and energy to take action and support and motivate other network members. Their passion aligns with the network’s collective vision and they put extra energy into making things happen. *Champions* may emerge in networks because networks have a clan culture or aim to build one^13^ (Appendix 4). The existence of a collective vision and psychological safe space in functional networks may facilitate the roles of *champions*.

At network formation, network members who are passionate about the problem and believe in the collection vision may emerge as *champions* (CMOC 11A). As the network evolves, *champions* may be identified and nurtured by network leaders (CMOC 11B). When *network champions* are capable and willing to take on additional responsibilities within the network, they play an important role in supporting the network to take action towards the identified problem and cultivating committed, engaged, and motivated network members. They support training, mentoring, and changing practices (CMOCs 11C, 11D). *Champions* may have pre-existing relationships that they bring to the network that help the network take action (CMOC 11E). However, when *champions* leave the network, there can be unintended consequences for these relationships and the network (CMOCs 11F, 11G).

From the network’s initiation, *champions* have played an important role in the NEST360 network. Early NEST360 *network champions* were passionate, respected people who had a good understanding of the context of the network hospitals and engaged well with the hospitals. They were key in forming the initial network relationships. As the network began to function, additional *champions* emerged in the network hospitals, who became trainers and mentors for the network. *Champions* have helped staff in the NBU change their practices of care. Some *champions* have brought with them relationships that are beneficial to network functioning, for example from the national or county ministries of health. Over time, some *champions* have transitioned out and left a void. For example, one of the network hospitals felt a reduction in the energy and enthusiasm around network activities following a *champion’s* departure.

| 11A | When a network provides a platform for members who are passionate and believe in the collective vision (context), they are willing to be champions (outcome) because doing so enables them to initiate change (mechanism) | New |
| --- | --- | --- |
| 11B | When network leaders identify and nurture influential and capable network members and support their leadership capabilities (context), this creates network champions (outcome) because they have the support to enact their skills and passions (mechanism)  *“If you're going to create a network, you're going to create a movement, you need to identify key movement makers, key game changers. People who genuinely care about what is happening and you will find them they are not necessarily the leader. In fact, if you should look beyond the leader. People who have some form of influence in their unit have been very helpful and across the country that's one of the things that has helped me, once I'm able to identify them, then it's easy to work with them.” (FI 29)* | New |
| 11C | When network champions are capable and willing to take on additional responsibilities within the network (context), they will train and mentor network members (outcome) because they see the value and have the skills and passion to do so (mechanism) | New |
| 11D | When network champions are capable and willing to take on additional responsibilities within the network (context), they will help change practices (outcome) because they see the value and have the skills and passion to do so (mechanism) | New |
| 11E | When network champions have pre-existing relationships that they bring to the network (context), this helps the network take action (outcome) because these relationships bring with them extra resources (e.g. access to people, lending legitimacy to the network) (mechanism) | New |
| 11F | When network champions leave the network (context), this may result in a loss of relationships important to the network (outcome) because these are personal to the champion (mechanism) | New |
| 11G | When network champions leave the network (context), this may result in a loss of energy to take action in the network (outcome) because network members feel less motivated and encouraged (mechanism) | New |

Table 9: CMOCs for Champions

**Adaptability**

Networks are initiated, form, function, and perform in complex open systems (i.e. health systems) and environments. Events may occur in a network’s environment, for example, an earthquake or the COVID-19 pandemic, which hinder its formation, functioning, and performance. Networks will need to adapt to form and undertake activities to work towards achieving their collective vision and sustain change and impact they achieve.

When there are unforeseen circumstances that are out of network members’ control that affect network formation, the network must *adapt* to be viable. If network members are passionate, innovative, committed, reflexive, and seek the input of others, this can help the network *adapt* its activities and functioning to continue to work towards the collective vision (CMOCs 15A, 15B). Network members and the skills and competencies that they contribute to the network are essential to network formation, functioning, and performing. When network members are moved to non-network facilities, the remaining network members will have to work to restore those same competencies within the network (CMOC 15C). Networks may also experience changes in network organisers or leadership that can influence network activity operations and financing and network members will have to *adapt* to continue implementing network activities (CMOC 15D). As the network becomes more established and responsibilities shift to different network members, this can help the network *adapt* to support network sustainability (CMOC 15E).

The NEST360 network has had to *adapt* during its formation and throughout its performing. During the network’s formation, the network had to significantly *adapt* its plans and activities because of COVID-19 related restrictions. At the beginning of the pandemic, the network ran a series of webinars on newborn care for the network and others interested. This led to modifying in-person trainings for clinicians and nurses on newborn care and devices to a hybrid format with three-days of virtual lectures followed by two-days of in-person practical training. Training for biomeds was conducted virtually and followed-up with mentorship during device installation and continued remote support. Meetings were moved online and tools such as Zoom and WhatsApp were leveraged to facilitate virtual trainings, meetings, and communication.

NEST360 network members are reflective on how network activities are being undertaken and take into account network hospital feedback to make changes along the way, for example with the QI visits. The network has had to continually *adapt* to changes among trained staff on the NBU and biomeds. Despite pro-active efforts to avoid this, many network hospitals have lost clinical, nursing, and biomedical staff. In response, remaining NBU and biomedical staff and mentors train the replacement staff on-the-job to avoid loss of skills needed to work towards the collective vision. The NEST360 network has also had to *adapt* to changes in the organisations that make up the network organising and leadership team (NEST360 Kenya). These changes have been challenging to network organisers (NEST360 Kenya team), who have largely remained the same, and required them to *adapt* to changing operational and financial processes to support network hospitals and activities. As the network evolves, the NEST360 Kenya team is making an effort to shift responsibilities for certain network activities, for example QI visits, to the counties and national MoH.

| 15A | When passionate, innovative, and committed network members face circumstances in the network’s operating environment that are out of the network’s control and affect the network forming (context), the network adapts its activities to be able to continue to work towards the collective vision (outcome) because the network members believe in the value of the network and its collective vision (mechanism)  *“So the first trainings we did, we piloted that the guidelines, the training in in Nyeri hospital and in Mama Lucy at that time, I'm the one who was doing the clinical part and Edith was doing the the nursing part. And then we had a medical engineer and oh now I remember what happened. So, you are now to scale-up in the other count...in the other facilities and we had COVID. Yes, somehow now, things just couldn't work the way we had planned, although we had planned for our for our training in May for the for the instructors because we wanted people to help us instruct but now but now that that could not happen.*  *And so, when now came to training the NEST programme because we had done very many webinars and even even the webinars, the nurses who do their parts, the engineers who do their part, and the clinical would do their their part. So, we used...three players that are put even in the webinars and demonstrate the use of machines, demonstrating how you do the procedures on the webinars. And then at that time, I thought, I can we can try a training, NEST training just in a webinar because of this contact. And then we did three days and who visited from from from morning to evening to six there and we will … because it was very interactive so that people don't sleep.*  *So, we're able to do virtual trainings, we did the second one, it was successful. And at that time, we were to lose money if we have not done any trainings. That yeah, there was no carry forward of the money in January, right. And I just wondered, I'm going to let Kenya down? No, I can't. And I just organised, we organised three trainings training simultaneously three hospitals, online, each with a team of trainers. So the the the three days we did we did together and that's in each of the facilities and all the sessions the theory sessions will be given by three people from the three different hospitals because when they go to the hospitals for practicals you don't want people to get lost to not have not to have rehearsed. So that training we had about 12 trainers for the plenary session. And then on after three days, they went onsite each each team so we had Kerugoya and we had Machakos and we had Thika they all went at site. And they now conducted the the hands-on experience on on the machine. I think to me that is the greatest things I'll always remember where I took a risk. And, that time what happened, we hadn't prepared the guidelines and I we said ‘No, we can they can look at the guidelines for the from the from the phone PDF guidelines.’” (FI 30)* | New |
| --- | --- | --- |
| 15B | When network members are reflexive and seek out input from others on network activity implementation (context), the network can adapt its functioning (outcome) because the network members develop a better understanding of the actions they need to take to work towards solving the identified problem (mechanism) | New |
| 15C | When skilled and capable network members leave network facilities (e.g. move to non-network facilities) (context), network members work to restore those same competencies within the network (outcome) because they recognise the value and importance of those skills in the network (mechanism) | New |
| 15D | When there are changes in network organisers or leadership that influence network activity operations and financing (context), network members will adapt to continue implementing network activities (outcome) because of their resilience and commitment to the network (mechanism) | New |
| 15E | As the network becomes more established and responsibilities shift to different network members (context), this helps the network adapt to support network sustainability (outcome) because it creates network resilience (mechanism) | New |

Table 10: CMOCs for Adaptability

#### Network foundations

Network processes and activities are underpinned by teamwork; a psychological safe space; committed, engaged, motivated, empowered, and confident network members; and purposeful relationships, linkages, and partnerships (see Foundations in Figures 1 and Appendix 5). Purposeful relationships, linkages, and partnerships; committed, engaged, and motivated network members; and a psychological safe space are part of the programme theory from our Realist Review; while teamwork and empowered and confident network members have emerged from the primary data. The CMOCs underpinning this part of the programme theory are in Tables 11– 16. I have provided one piece of illustrative data for one CMOC in each network foundation and additional illustrative data supporting the CMOCs is available in Appendix 8.

**Teamwork**

Networks can improve *teamwork* among network members within and across network facilities. Existing levels of *teamwork* within networks can help the network to form and function. When network members have opportunities, such as multidisciplinary team meetings, trainings, and inter/intra-facility QI and mentoring activities to learn and work together, this can improve multidisciplinary *teamwork* (CMOC 12A). *Teamwork* between different types of professions or specialities can improve in the network through interprofessional training opportunities (CMOC 12B). When there is improved *teamwork* in the network, network members can better support each other and do not feel alone in their job (CMOC 12C). Network members who feel part of a well-functioning team feel they can take the initiative to provide care and take action to change practices (CMOCs 12D, 12E). In an externally initiated network, *teamwork* between the network organisers or between network organisers and members can result in better implementation of network activities because they support each other (CMOC 12F).

In a network, *teamwork* emerges in the latter two phases of Tuckman’s small group development theory^8^ (Appendix 4) — norming and performing — when network members set out processes and (new) ways of working. For the network to optimally function and perform, *teamwork* is needed among network members and potentially with those outside the network who may influence its functioning as a way to mitigate potential blockages (i.e. Smesler’s social control mechanisms,^3 4^ Appendix 4). The existence of psychological safety^19^ (Appendix 4), which can be created by a network, helps to promote *teamwork*. Pre-existing *teamwork* in a network may also help to build psychological safety.

There are many different models of *teamwork* and the *teamwork* literature is vast. One model that is particularly applicable to networks is the LaFasto and Larson model (2001)^20^ (Appendix 4). Their characteristics of *teamwork* and collaboration are reflected in functional and performing networks. Networks enable members to reinforce these characteristics to ensure the presence of *teamwork*. For example, networks provide training and mentoring opportunities to improve the skills and competencies of network members. Strong relationships, commitment to the collective vision, positive culture, and supportive leaderships are key in networks and can help build *teamwork* in the network.

The NEST360 network put in significant effort to establish and strengthen *teamwork* within and across network facilities. It was able to accomplish this through multidisciplinary meetings that included clinicians, nurses, biomeds, and hospital administrators, trainings across the network and within facilities, and intra-and inter facility QI and mentoring activities. *Teamwork* was built and strengthened between clinical network members and the biomeds. Their relationships and ability to work together is one of the noteworthy changes in practice in the network. The network has also improved *teamwork* between the maternity and newborn units. *Teamwork* has enabled care practices to improve on NBUs because network members feel empowered to enact their role (also see CMOC 10E). Strong *teamwork* among the network organisers and *teamwork* between them and network hospitals has enabled them to better implement and support network activities.

| 12A | When network members have opportunities to learn and work together (context), this improves multidisciplinary teamwork (outcome) because they get to know each other better (mechanism) | New |
| --- | --- | --- |
| 12B | When the training provided to network members is multidisciplinary (context), this enables interprofessional teamwork among network members (outcome) because they understand each other’s roles and capabilities (mechanism)  *“So, for the good things I would say that right now NEST has is one of the programmes that has really promoted collaboration between the clinicians and the biomedical team. Initially before NEST came onboard there was that bit of there was no, you know, teamwork. And there was quite a bit of do I call it miscommunication or something. So, NEST has through these trainings that we do together, NEST has been able to bring together both the clinicians and the technical team to work together. And you see once a facility has brought in two or three people from working on the same things, then there is that bond that is created. And when they go back, they kind of work together because of what they have been trained from NEST.” (FI 17)* | New |
| 12C | When network members feel they are part of a well-functioning team (context), they support each other and do not feel alone in their job (outcome) because they feel responsible and accountable to each other (mechanism) | New |
| 12D | When network members feel they are part of a well-functioning team (context), network members feel that they can take the initiative to provide care (outcome) because they feel empowered and understand each other’s roles and capabilities (mechanism) | New |
| 12E | When network members feel they are part of a well-functioning team (context), network members feel that they can take action to change practices (outcome) because they feel responsible and accountable to each other (mechanism) | New |
| 12F | In an externally initiated network when there is teamwork among network organisers (context), this will enable better network activity implementation (outcome) because they support each other in their work (mechanism) | New |

Table 11: CMOCs for Teamwork

**Psychological safe space**

Primary data collection confirmed (CMOCs 9B-9E) or refined (CMOCs 9A (refined based on totality of data), 9F) all CMOCs, but did not add any additional dimensions to this part of the programme theory. The primary data reinforces that creating a *psychological safe space* is an important part of a network. A *psychological safe space* is formed and promoted when network members form trusting and respectful horizontal relationships (CMOC 9B). Once a network has created a *psychological safe space*, network members are more likely to learn, improve, seek feedback, and openly raise concerns and problems (CMOCs 9C, 9D). A *psychological safe space* encourages innovative behaviour and innovation in the network (CMOC 9E). It enables network members to more easily communicate and collaborate across facilities, levels of the health system, and sectors of care (CMOC 9F).

Our Realist Review suggests that *psychological safety*^19^ (Appendix 4) is more likely to be created in a network that formed from the bottom-up because there is more natural commitment to the collective vision and likely less hierarchy or imposed leadership. A more hierarchical culture in the workplace and society at large may be a hinderance to establishing *psychological safety.^21 22^* However, in networks that are formed from the top-down or externally initiated, there are approaches that network initiators, leadership, and organisers can take so that a *psychological safe space* is created in the network and therefore shift the hierarchical culture. These approaches include being available, supportive, and non-judgemental. The qualities of relational coordination^14^ (Appendix 4) are akin to the existence of a *psychological safe space*.

NEST360 has put in effort to create *psychological safe spaces* within the network. Inter-and intra-facility network meetings have helped to create teamwork and flatten hierarchies. This has enabled network members to share openly and freely experiences and challenges and identify ways to change practices. The network has been able to create an environment where network members do not feel a sense of fault finding but of improvement. Network facilities can be innovative and share what they have learned with other facilities in the network, for example different models of QI. Communication has also improved between network facilities and the lower-level facilities that refer to them and between the MoH and network hospitals.

| 9A | When network leadership/organisers are available and approachable, invite input and feedback, and model openness, fallibility, and non-judgmental behaviour (context), then this promotes a network’s psychological safety (outcome) because network members feel empowered and not threatened when they speak up or make a mistake (mechanism) | Refined |
| --- | --- | --- |
| 9B | When network members form trusting and respectful horizontal relationships (leading to flattened hierarchy) (context), then this promotes a network’s psychological safety (outcome) because network members feel equal (mechanism) | Confirmed |
| 9C | When a network has a shared network identity and culture that promotes a psychological safe space (context), then network members are more likely able to learn, improve, and seek feedback (outcome) because they feel empowered and a reduced fear of negative consequences (mechanism) | Confirmed |
| 9D | When a network is a psychological safe space for network members (context), it enables members to openly raise concerns or problems (outcome) because they know they will be supported and there won’t be negative repercussions (mechanism) | Confirmed |
| 9E | When a network is a psychological safe space for network members (context), it encourages innovative behaviour and innovation (outcome) because members know they will be supported and there won’t be negative repercussions (mechanism) | Confirmed |
| 9F | When a network creates a psychological safe space for network members (context), it enables them to more easily communicate and collaborate across the network’s facilities, levels, and sectors of care (outcome) because they already have a common ground and understanding (mechanism)  *“Hospitals that we've done mentorship programme…so that we have direct communication with them, direct communication, that is from the sub-county to the newborn unit. So that is one thing that shows that the referral facilities know they know what they're doing. And sometimes they can just call us for, you know for what we do. We've just delivered that baby with birth asphyxia or a baby with congenital abnormalities. What do we do and know, as mentors as mentors were able to give them information, we're able to to sometimes we correct them, and sometimes to encourage them in terms of so many things. So, one thing that they're receptive, very receptive.” (FI 07)* | Refined |

Table 12: CMOCs for Psychological safe space

**Commitment**

A network must work to get *commitment* from network members and outside stakeholders. The primary data added additional explanations for how networks cultivate *commitment* from network members, supported by five new CMOCs (7B, 7D, 7F, 7G, 7I, 7J), and while we had no primary data to support CMOC 7J, it made sense within the programme theory. Two CMOCs were confirmed (7A, 7E) and three refined (7C, 7H, 7K) by the primary data.

*Commitment* to a network is generated from network members when they identify with the network’s collective vision, identity, and culture (CMOC 7A); when their professional identity/calling aligns with the network vision (CMOC 7B); when they have support from leadership/administration/stakeholders (CMOC 7C); when the network enables members to achieve professional norms (CMOC 7D); and when network members feel they get emotional benefits or a sense of purpose from the network (CMOC 7E). When a network shows results from dedicated network members’ efforts, this can generate *commitment* from network hospital leadership (CMOC 7F). If network initiators or organisers promote the network’s efforts to the global community, this can also increase *commitment* from network members (CMOC 7G). Networks with *committed* members are more likely to act on their identified problem and collective vision (CMOC 7H). *Committed* network members may take action to disseminate knowledge to other members and those external to the network, which extends network benefits within and beyond the network (CMOC 7I). When influential outside stakeholders are actively engaged in network processes then they are more likely to be *committed* to the network (CMOC 7J) and it may be easier for the network to achieve its aims (CMOC 7K).

The four substantive theories that support this part of the programme theory from our Realist Review, Organisational Commitment Theory,^23 24^ Organisational Culture theory,^11 12^ Tuckman’s Small Group Development Theory,^8^ and Smesler’s Theory of Collective Behaviour^3 4^ (Appendix 4) are consistent with the revised CMOCs from the primary data. Our Realist Review highlighted that Tuckman’s stages of ‘forming’ and ‘storming’ may be important for generating *commitment,* but the primary data also points to the importance of the ‘performing’ stage. For example, when the network shows results to hospital leadership, this can generate the leaders’ *commitment* to the network because they see the value in what the network is doing. *Commitment* from non-network stakeholders in the network environment continues to be important to ease threats of ‘social control mechanisms’^3 4^ (Theory of Collective Behaviour, Appendix 4) that may impact network forming, functioning, and performing.

NEST360 has worked to cultivate *commitment* from the NBUs, hospital leadership, and national and county MoHs. Network members feel proud and positive about being part of the NEST360 network. The support provided by the NEST360 Kenya team in terms of training, mentorship, and ad hoc support to the network members helps them feel a sense of belonging. With the *commitment* that network members feel towards the network, they are motivated to act on the collective vision. At the start of the NEST360 network, the network initiators and organisers did not put in sufficient effort to engage the county government in network processes, which led to mixed interest and *commitment* in the network. The effects of this lack of engagement are now starting to be seen as NEST360 begins to reduce the level of support to the initial 13 hospitals in the network. For example, MoUs were put in place with the network hospitals instead of the counties and so as county leadership changes, they are often unaware of the network. NEST360 has however, engaged the MoH in network activities, particularly QI, which the MoH has taken a notable leadership role in.

| 7A | When network members identify with the network’s collective vision, identity, and culture (context), they are more likely to be committed to the network (outcome) because they believe in and value these (mechanism) | Confirmed |
| --- | --- | --- |
| 7B | When network members’ professional identity/calling align with the network vision (context), they are more likely to be committed to the network and enact affective commitment (outcome) because they professionally value/find importance in the network vision (mechanism) | New |
| 7C | When networks members have support from network leadership/organisers (context), they are more likely to be committed to the network (outcome) because they feel valued (mechanism) | Refined |
| 7D | When networks enable members to achieve professional norms (that are part of their professional identity) (context), they will be committed to the network (outcome) because of affective commitment (i.e. the alignment between their professional and the network’s norms) (mechanism) | New |
| 7E | When a network member gets ‘emotional’ benefits (positive feelings) or feel a sense of purpose from being part of the network (context), they are likely to be highly committed (outcome) because it is fulfilling for them (mechanism)  *“Let's say we feel proud to be part of NEST, we feel we refer ourselves as the NEST hospitals. And because it came and changed how we do things. It's changed how we, you know, we perceive things, it's changed our outcomes. So, we are very proud to be part of it. I must say, it's like, yeah, it's like a movement sort of. So, first of all, you are always motivated to do the best you can, you're motivated to make a difference for that child. And, you know, you stop doing things just the way you were doing them before, because now you know, better. So, I think it's more than just adding the group of people but from within, they found it changed in you. And then when you come together as a group, I think it's more of a movement. We want to make a difference for the children. So, I hope that can continue.” (FI 23)* | Confirmed |
| 7F | When a network shows network hospital/facility leadership it can improve care practices (context), this can generate commitment from leadership (outcome) because they see the network’s value (mechanism) | New |
| 7G | In an externally initiated network, when the network initiators promote the efforts and accomplishments of the network to donors and the interested global community (context), this increases network member commitment to the network (outcome) because network members feel their efforts are acknowledged and valued (mechanism) | New |
| 7H | If a network has committed and proud members (context), then they are more likely to act on the identified problem and collective vision (outcome) because members are willing to put in the energy, effort, and passion (mechanism) (modified) | Refined |
| 7I | When committed network members take action to disseminate knowledge to network members and others outside the network (context), this extends network benefits to network members and others outside the network (outcome) because of a greater availability of knowledge and skills (mechanism) | New |
| 7J | When influential outside stakeholders are actively engaged in network processes (context), then they are more likely to be committed to the network (outcome) because they can better understand the collective vision of the network (mechanism) | No supporting data |
| 7K | When influential outside stakeholders (often specific individuals within an organisation) are committed to the network and actively engaged in participating (context), then it may be easier for the network to achieve its aims (outcome) because network members feel empowered (mechanism) | Refined |

Table 13: CMOCs for Commitment

**Engaged and motivated network members**

*Engaged and motivated network members* are integral to ensure a network functions and works towards its collective vision. Primary data collection confirmed (CMOCs 8B-8D, 8F) and refined (CMOC 8A) the first section of this part of the programme theory on how a network develops *engaged and motivated network members*. In the second section of this part of the programme theory, the primary data contributed four new CMOCs (8E, 8G-8I) to understanding how *engaged and motivated network members* and engaged stakeholders contribute to the networks’ goals.

When network leadership provides opportunities for network members to be supported, recognised, and learn, this creates *engaged and motivated network members* (CMOC 8A). If personal identity strongly align with network identity and culture or network members actively participate in network change practices that aligns with their professional value, this can also develop *engaged and motivated network members* (CMOC 8B, 8C). When a network shows that it can affect change, network members are more likely to continue to engage with the network (CMOC 8D). Networks that leverage affective or normative professional commitment to work toward the networks’ goals will likely have more engagement from network members (CMOC 8E).

*Engaged and motivated network members* contribute to network goals. When *engaged and motivated network members* are provided with resources and the opportunity to act, they will attempt to enact changes in practices (CMOC 8F). If *engaged and motivated network members* that are committed to the network’s collective vision are faced with issues beyond their control, they will not be deterred from continuing to engage in the network (CMOC 8G). When *engaged and motivated network members* are moved out of the network, this will leave a gap in the network (CMOC 8H). The network may also engage non-network stakeholders to improve network activity implementation (CMOC 8I).

The substantive theories from our Realist Review, Organisational Commitment Theory and the Collective Identity Approach from New Social Movement theory^6 7^ (Appendix 4) continue to apply to this revised part of the programme theory. Network members’ type of commitment (affective, normative, continuance) to a network is an indication of the extent of their motivation and engagement^23-25^ (Appendix 4). These types of commitment align to Action Theory’s explanation of relationships between organisation members and the organisation’s goals^26^ (Appendix 4). Network members may commit solely to the collective vision (goals) as in affective or normative commitment, or they may accept the collective vision but have other objectives for being part of the network, as in continuance commitment.

Network members in NEST360 are *engaged and motivated* for different reasons that span affective, normative, and continuance commitment. NEST360 has created affective commitment to the network through the types of people they have recruited. Many NEST360 network members have affective commitment to the network because they have a professional calling or identity towards newborn care that has led them to be passionate network members. These network members – particularly when they are champions — can spread a sense of affective commitment. Some network members may have normative commitment to the NBU and network based on feelings of a moral obligation; however, this was challenging to differentiate from affective commitment.^23^ NEST360 has brought significant resources to the hospitals in the network and so there are likely network members who have continuance commitment to the network. As a result of these different types of commitment, it has *engaged and motivated network members* to change practices around newborn care. However, when these *engaged and motivated network members* are transferred to other hospital departments or out of the hospital, gaps are felt in the NBU. NEST360 also engages with non-network stakeholders to support activity implementation.

| 8A | When network leadership provides opportunities for network members to be supported, recognised, and learn through training, equipment provision and support, and facility renovations (context), this creates engaged and motivated network members (outcome) because they derive direct benefits and feel like they belong to the network (mechanism)  *“I'd say equipping the unit is is a big improvement. Because without the NEST programme probably, even now we will not be having CPAP equipment, people will not be knowing how to utilise the equipment. There’s also been a lot of training in terms of our staff being capacity built to know how to take care of a neonate, to know how to take care of premature. And the thing about monitoring the different pathways in terms of achievements, setting targets, also has made sure that we are kept on our toes in terms of ‘Do you know the vital signs for this patient?’ Now that you need to document ‘Do you know, the sugar levels for this patient at admission? That this child has sepsis?’ So, these are a lot of responsibility. Amongst our staff, there's also the need to maintain the standard for working within some standards to make sure that they achieve the goals that have been set by the programme. So definitely, one of the biggest changes has beneficiary has been the patient who is the ultimate recipient in terms of improvement of quality of care. That thing also has been to the staff who have been given a better environment to work in terms of equipping in terms of ensuring that supplies are available in terms of also ensuring that they have the requisite knowledge and skills to participate. And also, knowing that they have to continuously improve. I think it's also been of great benefit.” (FI 12)* | Refined |
| --- | --- | --- |
| 8B | When network members’ personal identity strongly aligns with a network’s identity and culture (context), then this will result in engaged and motivated network members (outcome) because they feel they belong (mechanism) | Confirmed |
| 8C | When network members actively participate in network change practices that align with the professional values they live by (context), this creates engaged and motivated network members (outcome) because it helps them to fulfil their moral obligation or vocational calling (mechanism) | Confirmed |
| 8D | When a network can show its members that it can affect some change (context), members are more likely to continue their engagement with it (outcome) because they can see its value and a sense of satisfaction with the positive changes (mechanism) | Confirmed |
| 8E | If a network can leverage the affective or normative professional commitment of its members to work towards achieving network goals (context), then they can become more engaged with the network (outcome) because of a sense of shared purpose (mechanism) | New |
| 8F | When engaged and motivated network members are provided with the resources and opportunity to act (context), then they will attempt to enact changes in practices (outcome) because they feel empowered to change practice/work towards solving the problem (mechanism) | Confirmed |
| 8G | When network members are committed to the collective vision that is strongly associated with their professional identity and are faced with issues that are beyond their control (context), they will not be deterred from continuing to engage with the network (outcome) because they believe what they are doing is worthwhile and feel accountable to other network members (mechanism) | New |
| 8H | When network members are transferred to facilities out of the network or out of the network unit (context), this leaves a gap in trained, engaged, and motivated network members (outcome) because their skills and knowledge are lost (mechanism) | New |
| 8I | When networks engage with non-network stakeholders in network activities (e.g. quality improvement) and deliberately seek out feedback and advice (context), this can improve network activity implementation (outcome) because the non-network stakeholders provide a broader understanding of relevant issues (mechanism) | New |

Table 14: CMOCs for Engaged and motivated network members

**Empowered and confident network members**

As network members become committed to and engaged with the network, their *confidence* in their skills may improve and they may feel more *empowered* to enact these skills and their role within and beyond the network. A network may acknowledge the capabilities of network members by selecting them to be network trainers and mentors, which makes them feel *confident and empowered* in their skills (CMOC 10A). When network members receive support to help improve and expand their skill set, this can improve their *confidence* in their new and existing skills. This often happens through activities, such as training and mentorship (CMOC 10B). Support can help network members feel *empowered* in their role and proactive in applying their skills beyond the network, for example to other departments within the network hospital that are not formally part of the network (CMOC 10C). When a network brings in previously neglected cadres/positions and enables them in their role, they can feel *empowered* and enact their role in the network (CMOC 10D). When network members feel *empowered* and are given freedom to use their clinical skills and enact their role, they are more likely to independently take action to address problems or provide care without waiting on someone more senior (CMOC 10E). Attitudes towards providing care will also change because network members are *empowered and confident* (CMOC 10F).

In a network, *empowerment* exists at the individual, organisation, and community level^1^ (community level see – Identify a problem) (Appendix 4). Engagement in a network can help members believe in their competencies, which can *empower* them. Processes such as decision making, managing resources, and collaborating with others on a shared goal may be *empowering* and are all processes that go on within a well-functioning network. Empowerment theory links with Bandura’s Self-Efficacy theory^10^ and Ajzen’s Theory of Planned Behaviour^9^ (Appendix 4). In a network, if members feel *empowered*, they will have belief in their ability to enact behaviours to influence certain outcomes. Furthermore, in line with Ajzen’s theory, when network members think they have the resources and opportunities to enact a behaviour, they will have more perceived control over enacting the behaviour, which will make them feel *empowered*. Empowerment theory also links with Quinn and Cameron’s Culture Typology^13^ (Appendix 4). When a network creates opportunities for shared responsibilities, social exchanges, and an enabling environment, all qualities of a clan culture, it is more likely to be *empowering*.

The NEST360 network created *confident and empowered* *network members* through its relationship building and network activities, such as training and mentoring. NEST360 selected clinicians and biomeds from the network hospitals to act as trainers and mentors. The selection process and training in these roles helped them to feel *confident and empowered* in their clinical and biomedical skills. Training of nurses and clinicians in the NBU and biomeds on clinical skills and devices led them to be more *confident*. The biomeds were able to take the skills they learned as part of the NEST360 network to other departments within the hospital. Prior to the NEST360 network, biomedical engineering was often a neglected hospital department and by bringing biomeds into the network, they have been *empowered* to enact their roles, which has enabled changes in practice in the NBUs. NEST360 also helped the nurses in the NBU to feel *confident and empowered* in their skills and roles, that they were able to address problems and provide care without waiting for their more senior colleagues.

| 10A | When a network acknowledges the capabilities of network members by selecting them to be network trainers and mentors (context), they become confident and empowered in their skills (outcome) because they develop the capacity to teach them (mechanism) | New |
| --- | --- | --- |
| 10B | When network members receive support (e.g. training, mentorship) to help them improve and expand their skill set (context), they become more confident in their previously learned and newly acquired skills (outcome) because they develop the capacity to perform them (mechanism) | New |
| 10C | When network members receive support (e.g. training, mentorship) to help them improve and expand their skill set (context), they feel empowered in their role and motivated to proactively apply their skills beyond the network (outcome) because they have greater confidence in their abilities (mechanism) | New |
| 10D | When a network brings in previously neglected cadres/positions into the network and enables them to do their role (context), they feel empowered to enact their role in the network (outcome) because they feel their skills and abilities are valued by the network (mechanism) | New |
| 10E | When network members (e.g. nurses) are given the freedom to use their clinical skills and role (context), they can take action to address problems/provide care without waiting for a more highly trained network member (e.g. doctor) (outcome) because they feel permitted to do so (mechanism)  *“And another thing they did was to to embrace teamwork. Yeah, initially it was like mine is nothing come and wait for the doctor to come and do whatever review and I wait to be no follow the instructions on the orders as indicated. The NEST approach was slightly different. All of us are involved. Like in the use of reviews of the babies, so we need to understand the management they took us through that knowledge. You know, the the trainings, empower the nurses with the relevant knowledge concerning various conditions. So, when the there's a problem or the condition is worsening, you need to escalate to the next level. But as you do that, there's something we're doing with the use of the machines we are using it to. By the time the doctor is coming to review, there's something already happening.” (FI 09)* | New |
| 10F | When providers in the network are well trained, mentored, or coached (context), this positively changes their attitudes around performing the skills/providing care (outcome) because they feel empowered and confident (mechanism) | New |

Table 15: CMOCs for Empowered and confident network members

**Purposeful relationships, linkages, and partnerships**

This part of the programme theory explains how *purposeful relationships, linkages, and partnerships* form within the network. *Relationships* are an essential part of a network and the intention and strength of these *relationships* set them apart from standard health system connections. There are three main sections to this part of the programme theory: how the network builds *relationships*, the opportunities for building *relationships*, and what the existence of these *relationships* enable. The primary data confirmed three CMOCs (4B, 4C, 4E), refined three (4G, 4J, 4O), added nine (4D, 4F, 4H, 4I, 4K-4N, 4P), and did not provide additional data to assess 4A.

Networks form purposeful and co-operative working *relationship, linkages, and partnerships* within and across facilities and professions in several ways. *Relationships* can form when there is belief in the network’s collective vision or when network members are open to and able to invest time in developing *relationships and linkages* through the network (CMOC 4A, 4B). The existence of strong pre-existing *relationships* between network members, a shared a professional identity, and a common baseline understanding between network members and what is expected of them can help to build *relationships* (CMOCs 4C, 4D, 4F). Artefacts that outline the roles and responsibilities of network members are also important in building *relationships* (CMOC 4E). Networks create opportunities for *relationship* building between network members’ within and across facilities (CMOC 4G). The provision of resources that supports the network members ways of working provides an opportunity for *relationship* building in the network, as does when network leadership creates a psychological safe space that enables cross learning between network members (CMOC 4H, 4I). Once purposeful and co-operative *relationships* are established, communication is improved between network members, ownership of the network may be strengthened, and understanding and trust between network members is built (CMOC 4J – 4M). Network members are better able to provide support to work towards the identified problem and create a psychological safe space (CMOC 4N, 4O). If the network has members of different professions, their attitudes towards each other may change as well (CMOC 4P).

Tuckman’s Small Group Development Theory,^8^ New Social Movement Theory’s Collective Identity Approach,^7^ and Organisational Culture Theory^11 12 27^ (Appendix 4) all remain relevant to the revised programme theory. Bartel and Rockmann’s work on relational systems and organisational resilience (Appendix 4) finds that when there is positive attention to *relationships* in an organisation, the value of interpersonal *relationships* to the organisation is recognised and resources are allocated to cultivate *relationships*.^18^ As explained in our Realist Review and above, *relationships* are key throughout a network’s evolution and the network provides different opportunities to build these key *relationships*.

The NEST360 network had the benefit of pre-existing *relationships* from the Clinical Information Network. However, despite these pre-existing *relationships* the NEST360 Kenya team and network members still needed to put in effort to strengthen these existing *relationships* and build new ones. For example, when the trainers and mentors made efforts to get to know NBU staff first, this supported mentorship activities. When there were changes within people or network organisations, this undermined network functioning because new *relationships* needed to be formed. MoUs were put in place between the hospitals and the network organisers (NEST360 Kenya). The NEST360 network created different opportunities to develop and strengthen *relationships*, including cross-professional trainings with clinicians, nurses, and biomeds and trainings across levels of facilities (i.e. Level 5 hospitals in the network training Level 4 hospitals that refer to them). Opportunities for cross-learning at inter-hospital network meetings helped to form *relationships* amongst hospital administrators, nurses, and paediatricians at different hospitals in the network. *Relationships* in the NEST360 network have improved communication between NBU nurses and biomeds, generated a feeling of ownership of the network activities on the part of the hospitals, enabled support in changing and improving clinical and biomedical practices, and created a feeling of psychological safety within and among the NBUs in the network. While trust emerged in the *relationships* between the network organisers (NEST360 Kenya) and network hospitals, changes to the organisations that make up the NEST360 Kenya team weakened this trust.

| 4A | When network members believe in the network’s collective vision (context), then this can help ensure that network members have purposeful and co-operative working relationships (outcome) because they are more willing to work and value working with people whom they identify as being likeminded (mechanism) | No supporting data |
| --- | --- | --- |
| 4B | When network members are open to and able to invest time in developing relationships and linkages through the network (context), then this can help ensure that network members have purposeful and co-operative working relationships (outcome) because they have a better understanding of each other (mechanism) | Confirmed |
| 4C | If network members have strong pre-existing relationships (context), then this helps ensure that network members have purposeful and co-operative working relationships (outcome) because they are already familiar with each other (mechanism) | Confirmed |
| 4D | When network members share a professional identity (context), this supports forming purposeful and co-operative working relationships (outcome) because they share common socialised perspectives (mechanism) | New |
| 4E | If there are artefacts in place that outline the roles and responsibilities of network members (context), then this can help ensure that network members have purposeful and co-operative working relationships (outcome) because there is a common understanding among network members about what they are expected to do (mechanism) | Confirmed |
| 4F | When a common baseline understanding is created among network members of what is expected of them (context), this helps to develop purposeful relationships (outcome) because the network members have the same level of understanding about each other and their roles and responsibilities (mechanism) | New |
| 4G | When a network creates opportunities for relationship building between network members within and across facilities (context), this may help in the creation of purposeful and co-operative working relationships and improve existing working relationships (outcome) because they get to know each other’s personality, skills, ways of working, and motivations better (mechanism)  *“Actually feel the major thing that helped create the relationship was ultimately training, one for the nurses to accept and recognise they need the biomeds to support them in terms of equipment functionality. And other hand for the biomeds to recognise and accept for optimal use of this equipment, they need regular checks. Yes. So that alone now builds the confidence. For the two teams for the clinical and the biomed and they realised we need to work together. Yeah, not like, initial whereby we're like, just the equipment is okay, then that's all. But today, you're like, we have to work together. And through that the nurses saw or rather felt, the biomed has been trained specifically on this newborn equipment, then the level of confidence went up. And they realise that now these people are okay now to use or rather to maintain these devices. And with that now followed, now, the assigning of a focal person in the unit, the body is a person that has been trained. And that's now built even more confidence with the clinical team, that now they could now create the rapport and even directly contact this person to come and check on their devices. And with that, now being on routinely now it has created now that's good relationship between the biomed and the clinical team.” (FI 08)* | Refined |
| 4H | When the network provides resources that support the network members’ ways of working (context), this may help in the creation of purposeful and co-operative working relationships and improve existing working relationships across professions (outcome) because they are better able to collaborate and do their job (mechanism) | New |
| 4I | When network leadership creates a psychological safe space that enables cross-learning between network members (context), this may help in the creation of purposeful and co-operative working relationships and improve existing working relationships (outcome) because network members are able to openly share their experiences (mechanism) | New |
| 4J | When network members have established purposeful relationships within and across network facilities (context), this helps to improve communication between network members (outcome) because they are familiar with each other (mechanism) | Refined |
| 4K | When purposeful linkages and strong working relationships are established between network members (context), this improves network members ownership of the network (outcome) because they feel connected to each other and to the vision (mechanism) | New |
| 4L | When purposeful linkages and strong working relationships are established between network members (context), this helps to build trust among network members (outcome) because network members have confidence in each other (mechanism) | New |
| 4M | When purposeful linkages and strong working relationships are established between network members (context), this supports the creation of a psychological safe space (outcome) because network members feel they can speak freely without fear of negative consequences (mechanism) | New |
| 4N | When purposeful linkages and strong working relationships are established between network members/organisers (context), this enables network members/organisers to provide support in enacting the collective vision (outcome) because they can develop a mutual understanding of the identified problem and potential solutions (mechanism) | New |
| 4O | When purposeful linkages and strong working relationships are established between network members (context), this helps them to be in agreement (outcome) because they can more easily develop a shared conceptualisation of the identified problem and potential solutions (mechanism) | Refined |
| 4P | When different professions within the network (e.g. clinical and technical) develop strong working relationships (context), their attitudes toward the other profession changes (outcome) because they understand each other’s value (mechanism) | New |

Table 16: CMOCs for Purposeful relationships, linkages, and partnerships

#### Network cross-cutting strategies

The network cross-cutting factors, *communication, trust,* and *energy, effort, and passion*, (see Cross-cutting arrows in Figures 1 and Appendix 5) are not supported by separate sections of CMOCs and data but they appear across the network processes, activities, and foundations. *Communication* is required for processes, activities, and foundations in network initiation, but it also improves among network members as the network evolves (CMOCs 2I, 3I, 4J, 5B, 5C, 9F). *Trust* emerges from the network activities, foundations, and processes but is also pre-existing to a degree for network initiation to occur (CMOCs 2F, 4L, 9B). The formation of purposeful relationships, linkages, and partnerships can help to build *trust* within the network. *Trust* is important in the formation and functioning of a network,^15^ however it is something that did not emerge strongly in our Realist Review or Evaluation. *Energy, effort, and passion* are needed from a dedicated group of network members for the network to form and function, but as the network evolves they become more distributed across the network (CMOCs 1A, 1C, 2N, 3G, 3H.3, 3J.1-3K, 5S, 5T, 7H, 7G, 11A-11D, 11G, 13B.1, 13B.2, 13E, 15A, 16E).

### References

1. Zimmerman MA. Empowerment Theory: Psychological, Organizational and Community Levels of Analysis. In: Rappaport J, Seidman E, eds. Handbook of Community Psychology. New York: Kluwer Academic/Plenum Publishers 2000.

2. UN. Goal 3: Ensure healthy lives and promote well-being for all at all ages: United Nations Department of Economic and Social Affairs Sustainable Development; [Available from: <https://sdgs.un.org/goals/goal3#targets_and_indicators> accessed February 26 2024.

3. Weeber S, Rodeheaver D. Militias at the Millennium: A test of Smelser’s Theory of Collective Behavior. *The Sociological Quarterly* 2003;44(2):181-204. doi: 10.1111/j.1533-8525.2003.tb00554.x

4. Ormrod JS. Smelser’s Theory of Collective Behaviour. Fantasy and Social Movements UK: Palgrave Macmillan 2014.

5. Engles B, Muller M. Northern theories southern movements?: contentious politics in Africa through the lens of social movement theory *Journal of Contemporary African Studies* 2019;37(1):72-92. doi: 10.1080/02589001.2019.1607967

6. Fominaya CF. Collective Identity in Social Movements: Central concepts and debates. *Sociology Compass* 2010;4/6(393-404) doi: 10.1111/j.1751-9020.2010.00287.x.

7. Polletta F, Jasper J. Collective Identity and Social Movements. *Annual Review of Sociology* 2001;27:283-305.

8. Tuckman B. Developmental sequence in small groups. *Psychological Bulletin* 1965;63(6):384-99.

9. Fishbein M, Ajzen I. Belief, attitude, intention, and behavior: an introduction to theory and research. Reading, Mass; London: Addison-Wesley 1975.

10. Bandura A. Self-efficacy: Towards a unifying theory of behavioral change *Psychological Review* 1977;84(2):191-215.

11. Ravasi D, Schultz M. Responding to organizational identity threats: exploring the role of organizational culture. *Academy of Management Journal* 2006;49(3):433-58.

12. Schein EH. Organizational Culture. *American Psychologist* 1990;45(2):109-19.

13. Cameron KS, Quinn RE. The Competing Values Framework. Diagnosing and Changing Organizational Culture San Francisco: Jossey-Bass 2011.

14. Bolton R, Logan C, Gittell JH. Revisiting Relational Coordination: A systematic review. *The Journal of Applied Behavioral Science* 2021;57(3):290-322. doi: 10.1177/0021886321991597

15. Kalaris K, Wong G, English M. Understanding networks in low-and middle-income countries’ health systems: A scoping review. *PLOS Global Public Health* 2023;3(1) doi: 10.1371/journal.pgph.0001387

16. Ganz M. Leading Change: Leadership, Organization, and Social Movements. In: Nohria N, Khurana R, eds. Handbook of Leadership Theory and Practice: A Harvard Business School Centennial Colloquium. Boston, MA Harvard Business Press 2010:527-68.

17. Ganz M, McKenna E. Bringing Leadership Back In In: Snow DA, Soule SA, Kriesi H, et al., eds. The Wiley Blackwell Companion to Social Movements Second ed: John Wiley & Sons Ltd 2019.

18. Bartel CA, Rockmann K. The disease of indifference: How relational systems provide the attentional infrastructure for organizational resilience. *Strategic Organization* 2024;22(1) doi: 10.1177/14761270231183441

19. Edmondson A. Psychological Safety, Trust, and Learning in Organizations: A Group-Level Lens. In: Kramer RM CK, ed. Trust and Distrust in Organizations: Dilemmas and Approaches. New York: Russell Sage Foundation 2004.

20. LaFasto F, Larson C. When teams work best: Sage Publications 2001.

21. Nembhard IM, Edmondson AC. Making it safe: The effects of leader inclusiveness and professional status on psychological safety and improvement efforts in health care teams. *Journal of Organizational Behaviour* 2006;27:941-66. doi: 10.1002/job.413

22. Edmondson AC, Higgins M, Singer S, et al. Understanding psychological safety in health care and education organizations: A comparative perspective. *Research in Human Development* 2016;13(1):65-83. doi: 10.1080/15427609.2016.1141280

23. Meyer J, Allen N. A three-component conceptualization of organizational commitment *Human Resources Management Review* 1991;1(1):61-89.

24. Porter L, Steers R, Mowday R, et al. Organizational commitment, job satisfaction, and turnover among psychiatric technicians. *Journal of Applied Psychology* 1974;59(5):603-09.

25. Manetje O. The impact of organisational culture on organisational commitment. University of South Africa, 2009.

26. Bowey AM. Approaches to organisation theory. *Soc Sci Inform* 1972;11(6):109-28.

27. Allaire Y, Firsirotu ME. Theories of Organisational Culture. *Organisation Studies* 1984;5/3(193-226)
